# Massive transcriptome sequencing of human spinal cord tissues provides new insights into motor neuron degeneration in ALS

**DOI:** 10.1038/s41598-017-10488-7

**Published:** 2017-08-30

**Authors:** Anna Maria D’Erchia, Angela Gallo, Caterina Manzari, Susanna Raho, David S. Horner, Matteo Chiara, Alessio Valletti, Italia Aiello, Francesca Mastropasqua, Loredana Ciaccia, Franco Locatelli, Francesco Pisani, Grazia Paola Nicchia, Maria Svelto, Graziano Pesole, Ernesto Picardi

**Affiliations:** 1 Department of Biosciences, Biotechnology and Biopharmaceutics, University of Bari, Via Orabona 4, 70126 Bari, Italy; 2 Institute of Biomembranes, Bioenergetics and Molecular Biotechnologies (IBIOM), National Research Council, Via Amendola 165/A, 70126 Bari, Italy; 3 Department of Pediatric Oncohaematology, Bambino Gesù Children’s Hospital IRCCS, Piazza S. Onofrio 4, 00165 Rome, Italy; 4 National Institute of Biostructures and Biosystems (INBB), Viale Medaglie D’Oro 305, 00136 Rome, Italy; 5 Department of Biosciences, University of Milan, Via Celoria 26, 20133 Milan, Italy; 6 Center of Excellence in Comparative Genomics, University of Bari, Piazza Umberto I, 70121 Bari, Italy

**Keywords:** Gene expression profiling, Amyotrophic lateral sclerosis

## Abstract

ALS is a devastating and debilitating human disease characterized by the progressive death of upper and lower motor neurons. Although much effort has been made to elucidate molecular determinants underlying the onset and progression of the disorder, the causes of ALS remain largely unknown. In the present work, we have deeply sequenced whole transcriptome from spinal cord ventral horns of post-mortem ALS human donors affected by the sporadic form of the disease (which comprises ~90% of the cases but which is less investigated than the inherited form of the disease). We observe 1160 deregulated genes including 18 miRNAs and show that down regulated genes are mainly of neuronal derivation while up regulated genes have glial origin and tend to be involved in neuroinflammation or cell death. Remarkably, we find strong deregulation of SNAP25 and STX1B at both mRNA and protein levels suggesting impaired synaptic function through SNAP25 reduction as a possible cause of calcium elevation and glutamate excitotoxicity. We also note aberrant alternative splicing but not disrupted RNA editing.

## Introduction

Amyotrophic lateral sclerosis (ALS) is a fatal and devastating neurodegenerative disorder that causes the progressive death of upper and lower motor neurons^1^. Symptoms and clinical signs, including muscle weakness and atrophy, fasciculations, hyperreflexia, dysarthria and dysphagia emerge when axonal connections fail, leading to denervation^1^. The worldwide incidence of ALS is approximately two per 100,000 individuals and the mean age of onset is 55–60 years, with a higher incidence among men than women^1^.

The vast majority of documented cases are sporadic (~90%), without any associated family history. Sporadic and inherited (usually through an autosomal dominant pattern) forms of ALS show common clinical and pathological traits, suggesting equivalent pathogenic mechanisms^1^.

Although much effort has been done in elucidating molecular factors underlying the onset and progression of the disorder, the causes of ALS remain unknown^2^. However, numerous investigations in humans and animal models have associated nucleotide variants in a small group of genes (including SOD1, UBQLN2, VCP, OPTN, TDP43, FUS, MATR3 and a hexanucleotide repeat expansion within c9orf72) with the familial form of ALS^2^.

Microarray-based transcriptome studies have revealed multiple perturbations of motor neuron function in both forms of ALS^3, 4^, consistent with the hypothesis that various cellular events, including mitochondrial dysfunction, enhanced apoptosis, glutamate-mediated excitotoxicity, free radical injury, protein misfolding, abnormal calcium metabolism, altered axonal transport and activation of proteases and nucleases contribute to the pathobiology of the disease^5^.

Since motor neurons cannot be sampled during life, investigations into ALS neurodegenerative process are carried out in neuronal-like cells in culture, animal models carrying mutant transgenes or post-mortem material from patients. In the last case, the spinal cord represents the elective tissue and a pivotal source of RNA for gene expression profiling in ALS. While mixed cell populations in which astrocytes, microglia and oligodendrocytes may obscure the distinctive expression profile of motor neurons, which are the most affected in ALS, the co-culture of motor neurons and glial cells has shown an intricate interplay, suggesting that non-neuronal cells play an active role in neurodegeneration^6^. Accordingly, transcriptomic studies using high throughput sequencing approaches (RNA-Seq) to target specific tissues, such as ventral horns of the spinal cord, may provide new insights into genes and pathways involved in the degeneration of motor neurons. Indeed, RNA-Seq allows more accurate and complete expression profiling than classical microarrays, overcoming their reliance on pre-designed probes. Recently, two studies exploring transcriptome alterations in ALS by the RNAseq technology have been reported^7, 8^. The first one investigated transcriptome changes in cerebellum and frontal cortex from familiar (fALS) (with mutated c9orf72 gene) and sporadic ALS (sALS) donors, showing extensive dysregulation of alternative splicing and alternative polyadenylation in both ALS groups^7^. The second one, based on RNAseq technology, investigated transcriptome changes in cervical spinal cord tissues from sALS donors showing that neuroinflammation mediated by TNF (tumor necrosis factor) is the main transcriptome abnormality in sALS^8^.

In the current work, we also report the analysis of whole transcriptome sequencing data from matched control and sALS post-mortem donors but, differently from previous studies, we focus on the detection and characterization of significant changes in ventral horns of the human lumbar spinal cord, a tissue never investigated at single nucleotide resolution. While massive sequencing of the low molecular weight RNA fraction showed 18 dysregulated miRNAs involved in TGF-beta signaling, axon survival and outgrowth, RNA-Seq data revealed altered expression of 1142 genes belonging to a restricted range of ALS deregulated molecular pathways including an impaired synaptic function. In particular, we focused our attention on two downregulated neuronal t-SNARE proteins, SNAP25 and Syntaxin-1B (STX1B), as they form with the v-SNARE protein synaptobrevin, a ternary complex mediating the neurotransmitter release at the presynaptic membrane. Notably, SNAP25 negatively regulates native voltage-gated calcium channels (VGCCs) in glutamatergic neurons^9^, and its reduced expression enhances glutamatergic neurotransmission^10^ and causes neurodegeneration^11^.

Taken together, our results may represent a remarkable breakthrough to unveil sALS, supporting a primary role of t-SNARE protein expression as possible cause of intracellular calcium elevation and glutamate excitotoxicity^12^ and suggesting SNAP25 and related SNARE proteins as candidate novel indicators and signatures of motor neuron death to be monitored in ALS patients.

## Results

### RNA-Seq data analysis

Our study includes ventral horns of the lumbar spinal cord from six human donors affected by sporadic ALS and five age, sex and ethnicity matched controls (Table 1). ALS and control transcriptomes were sequenced employing the Illumina technology on the NextSeq 500 platform. We generated 1.9 billion 100 bp strand-specific paired end reads with, on average, 179 million pairs of reads per sample (Table 2). About 73% of these reads were uniquely aligned to the reference human genome (hg19 assembly) after a cleaning step to remove low quality regions (Table 2). Using RefSeq annotations we verified that about 98% of mapped reads maintained the correct strand orientation and, on average, 46% of mapped bases corresponded to UTR and coding regions of known mRNA transcripts (Table 2).

**Table 1:** Control and ALS samples used in this study.

Bank	ID	BID	Age	Sex	Race	Diagnosis	PMI	RNA-Seq	miRNA-Seq	qRT-PCR	WB	IF
NICHD	A1	5388	61	M	Ca	sALS	8			✓		
NICHD	A2	925	54	M	Ca	sALS	3			✓		
NICHD	A3	4762	59	M	Ca	sALS	6			✓		
NICHD	A4	5595	56	M	Ca	sALS	16	✓	✓	✓	✓	
NICHD	A5	1292	45	M	Ca	sALS	15	✓	✓	✓	✓	✓
HBSFRC	A6	5215	59	M	Ca	sALS	12,5	✓	✓	✓	✓	
HBSFRC	A7	5187	69	M	Ca	sALS	13,1	✓	✓	✓	✓	
HBSFRC	A8	5165	55	M	Ca	sALS	18,3	✓	✓	✓	✓	✓
HBSFRC	A9	5223	47	M	Ca	sALS	16	✓	✓			✓
HBSFRC	A10	5216	53	M	Ca	sALS	16,8			✓	✓	✓
NICHD	A11	5678	55	M	Ca	sALS	6					✓
NICHD	H1	5456	57	M	Ca	ACD	16	✓	✓	✓	✓	✓
NICHD	H2	5663	42	M	Ca	UK	16	✓	✓	✓	✓	
NICHD	H3	5458	64	M	Ca	MI	13	✓	✓	✓	✓	
NICHD	H4	5361	42	M	Ca	CT	19	✓	✓	✓	✓	✓
NICHD	H5	5617	59	M	Ca	MI	10	✓	✓	✓	✓	✓
NICHD	H6	5393	56	M	Ca	ACD	22			✓		
NICHD	H7	5353	43	M	Ca	CD	9			✓	✓	✓

For each sample we report: Bank (Tissue bank: NICHD or HBSFRC), ID (Sample ID used in the manuscript, Figures and Tables), BID (unique Bank ID from tissue bank), age, sex, race (Ca = Caucasian), main diagnosis (sALS = Sporadic ALS; ACD = Atherosclerosis Cardiovascular Disease; HEM = Hemopericardium; UK = Unknown; CD = Combined Drugs; MI = Multiple injuries; CT = Cardiac tamponade), post-mortem delay (in hours), flags indicating if the sample has been assayed for RNA-Seq, miRNA-Seq, qRT-PCR, immunoblotting (WB) or immunofluorescence (IF).

**Table 2:** Main statistics for RNA sequencing.

ID	#FR	#CFR	#AFR	% AFR	% RB	% COD	% UTR	% INTR	% INTER	% MRNA	% COR_STR
A4	54680251	45068732	40934057	90,83	1,00E-06	0,153	0,135	0,433	0,279	0,288	0,972
A5	85144930	66912832	58474545	87,39	2,00E-06	0,217	0,162	0,440	0,182	0,378	0,984
A6	76440384	61373970	53373122	86,96	2,00E-06	0,277	0,210	0,386	0,127	0,487	0,992
A7	83340776	74094106	64311655	86,80	2,00E-06	0,292	0,226	0,347	0,134	0,518	0,993
A8	74664234	55858789	48680941	87,15	2,00E-06	0,299	0,219	0,358	0,123	0,519	0,993
A9	96273069	85995872	75818167	88,16	2,00E-06	0,234	0,203	0,428	0,135	0,437	0,990
H1	109502733	88454014	77268812	87,35	2,00E-06	0,237	0,197	0,432	0,133	0,434	0,990
H2	106819322	91146575	80112958	87,89	2,00E-06	0,243	0,193	0,423	0,142	0,435	0,989
H3	103962426	88206006	77528076	87,89	2,00E-06	0,302	0,233	0,342	0,122	0,536	0,993
H4	92670547	81318295	73329651	90,18	1,00E-06	0,297	0,230	0,338	0,134	0,528	0,992
H5	101742727	79106426	69660162	88,06	2,00E-06	0,267	0,216	0,384	0,133	0,483	0,992
Mean	**89567400**	**74321420**	**65408377**	**88,06**	**1,82E-06**	**0,256**	**0,202**	**0,392**	**0,149**	**0,459**	**0,989**

Here we report main statistics about RNA sequencing calculated by in house python scripts and Picard tools. RefSeq gene annotations were downloaded from UCSC genome browser. Column headers are: ID (Unique Sample ID); #FR (Number of sequenced Fragments); #CFR (Number of cleaned Fragments); #AFR (Number of uniquely aligned Fragments); % AFR (Fraction of uniquely aligned Fragments); % RB (Fraction of Ribosomal Bases); % COD (Fraction of Protein Coding Bases); % UTR (Fraction of UTR Bases); % INTR (Fraction of Intronic Bases); % INTER (Fraction of Intergenic Bases); % MRNA (Fraction of mRNA Bases); % COR_STR (Fraction of Fragments in the correct orientation). The effective number of sequenced read is equal to the double of the number of fragments.

To confirm the sporadic disease nature of our samples, we checked for the presence of known ALS mutations in aligned RNAseq data using REDItools^13^ and did not find any potentially fALS- associated variant in the causative genes annotated in the ALS Online Genetic Database (ALSoD http://alsod.iop.kcl.ac.uk/)^14^.

**Figure 1:**
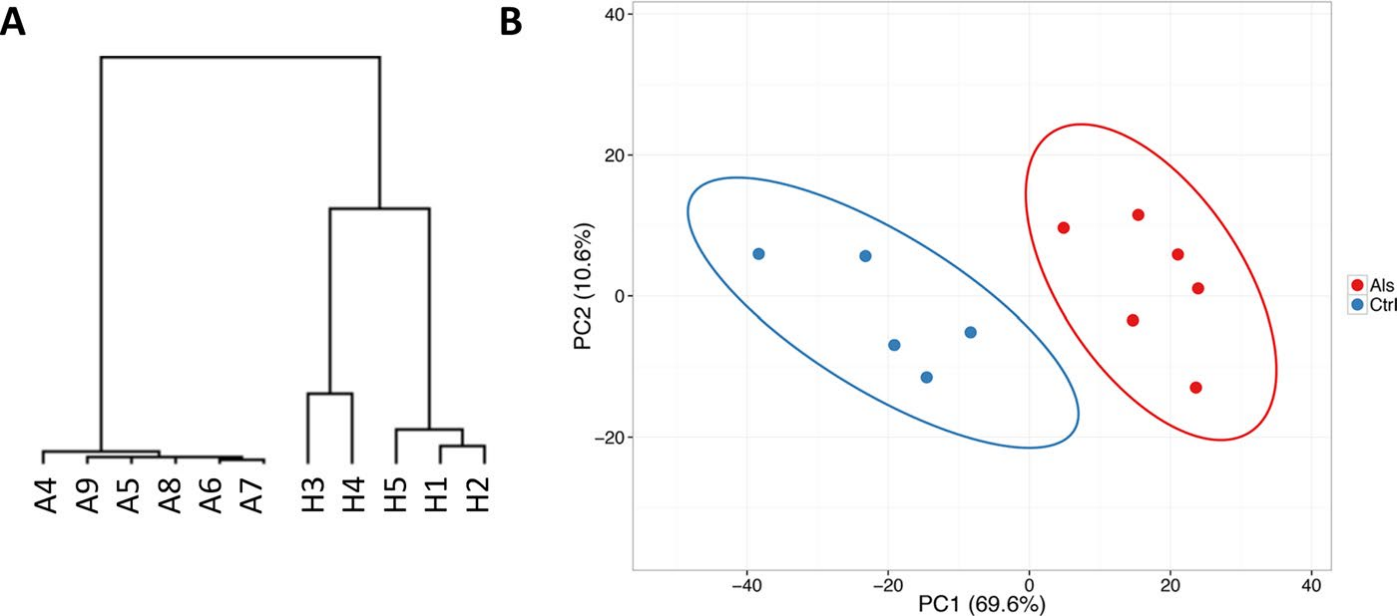
Hierarchical clustering and PCA analysis of RNAseq data. (**A**) Hierarchical clustering conducted on CuffDiff2 expression values (in FPKM). ALS and control samples are clearly separated in two distinct groups. (**B**) PCA analysis of variant genes in which input samples are clustered in diseased (red circles) and normal (blue circles) groups.

We calculated RNAseq based gene expression levels in FPKM and performed unsupervised hierarchical clustering to detect similarities in gene expression profiles between ALS and control samples. We observed a clear separation between diseased and normal groups, suggesting specific transcriptome signatures for ALS (Fig. 1A). Principal component analysis (PCA) of variant genes re-marked such separation (Fig. 1B).

**Figure 2:**
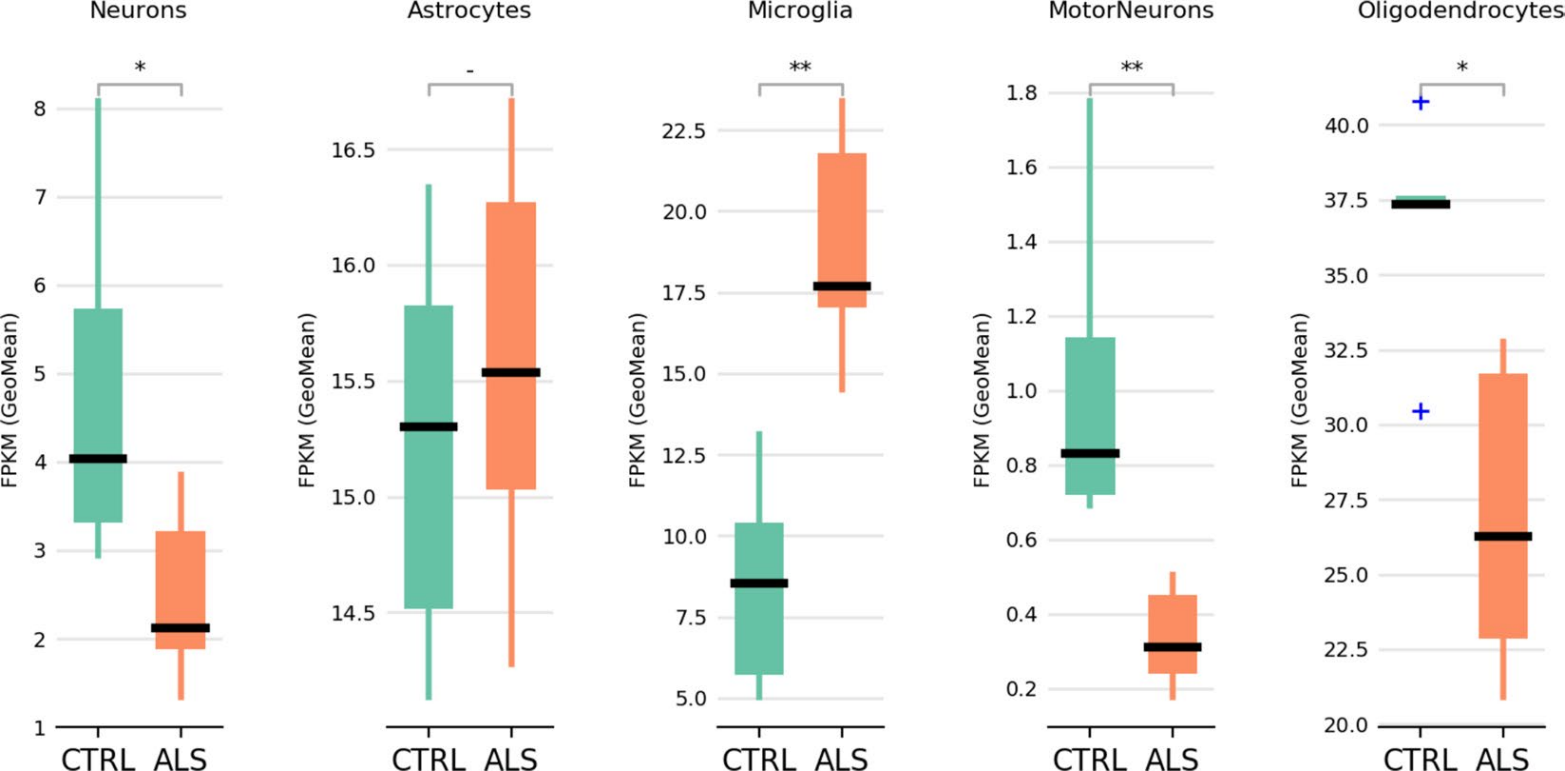
Cell type composition analysis of human spinal cord samples. For each cell type we show the geometric mean of the FPKM values of the cell type marker genes (Supplementary Table 1). Statistically different cell type composition is calculated by Mann-Whitney U test (two-tails). ^*^P < 0.05; ^**^P < 0.01; ^−^Not significant.

Using cell type specific genes for neurons, motorneurons, astrocytes, oligodendrocytes and microglia collected from the literature^6, 15^, we verified the cell type composition of our spinal cord samples (Fig. 2 and Supplementary Table 1). Interestingly, we found statistically significant changes in cell populations composed by neurons, motorneurons, oligodendrocytes and microglia. No substantial differences were detected in the astrocyte population (Fig. 2). While neuron, motorneuron and oligodendrocyte populations appeared depleted in ALS samples, microglial population was over-represented suggesting neuroinflammation, that is thought to be critically important in the neurodegenerative process^16^ (Fig. 2).

### Differentially expressed genes in ALS and controls

**Figure 3:**
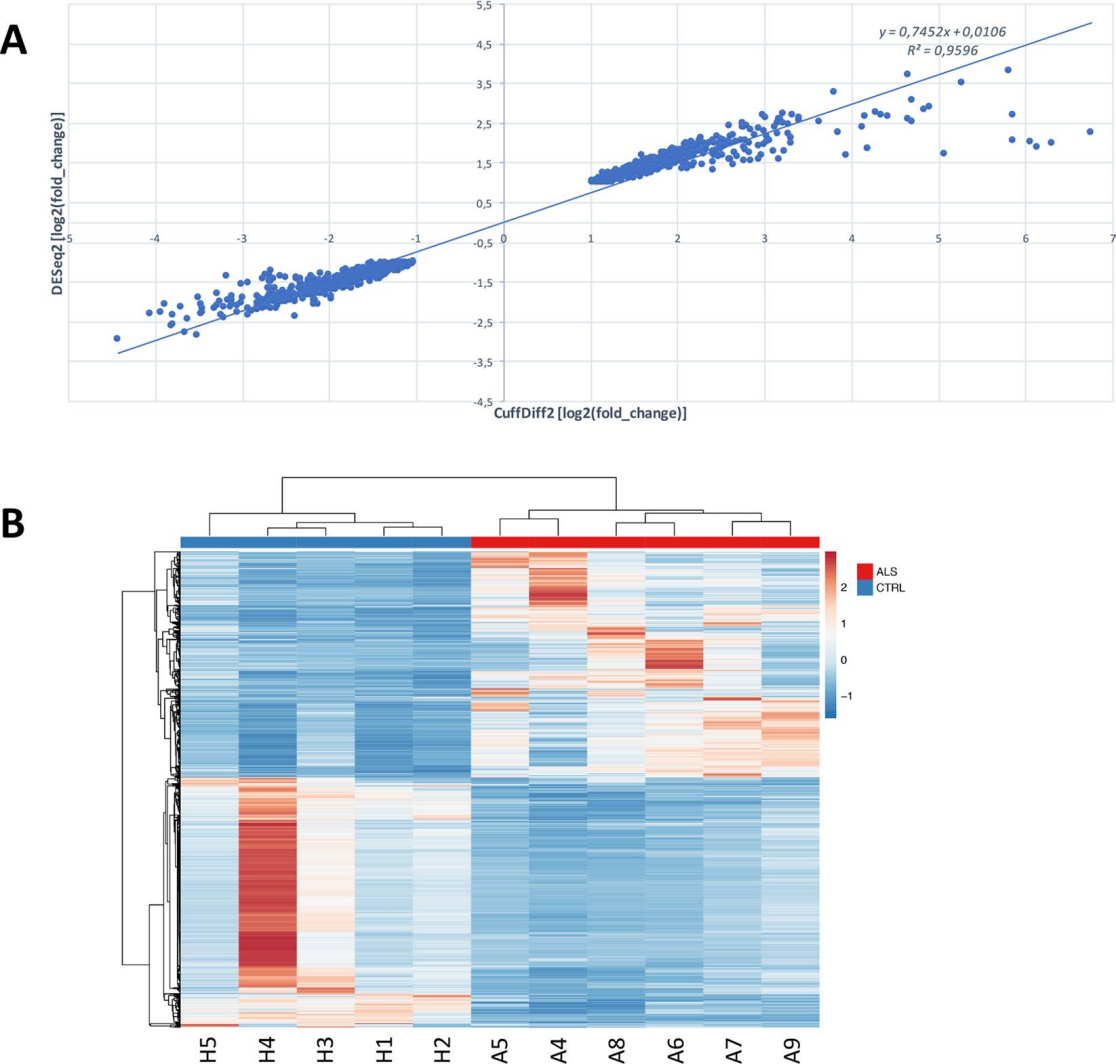
Gene expression analysis of ALS and controls samples using RNAseq. (**A**) Correlation between log2(FC) values of differently expressed genes detected by CuffDiff2 and DEseq2 (Pearson r = 0.98). (**B**) Heat map of 1142 genes differentially expressed between ALS and control samples. Color bars above the heat map indicate the disease status: red, ALS; blue, control. Each row of the heat map represents the Z-score transformed FPKM values of one differentially expressed gene across all samples.

**Figure 4:**
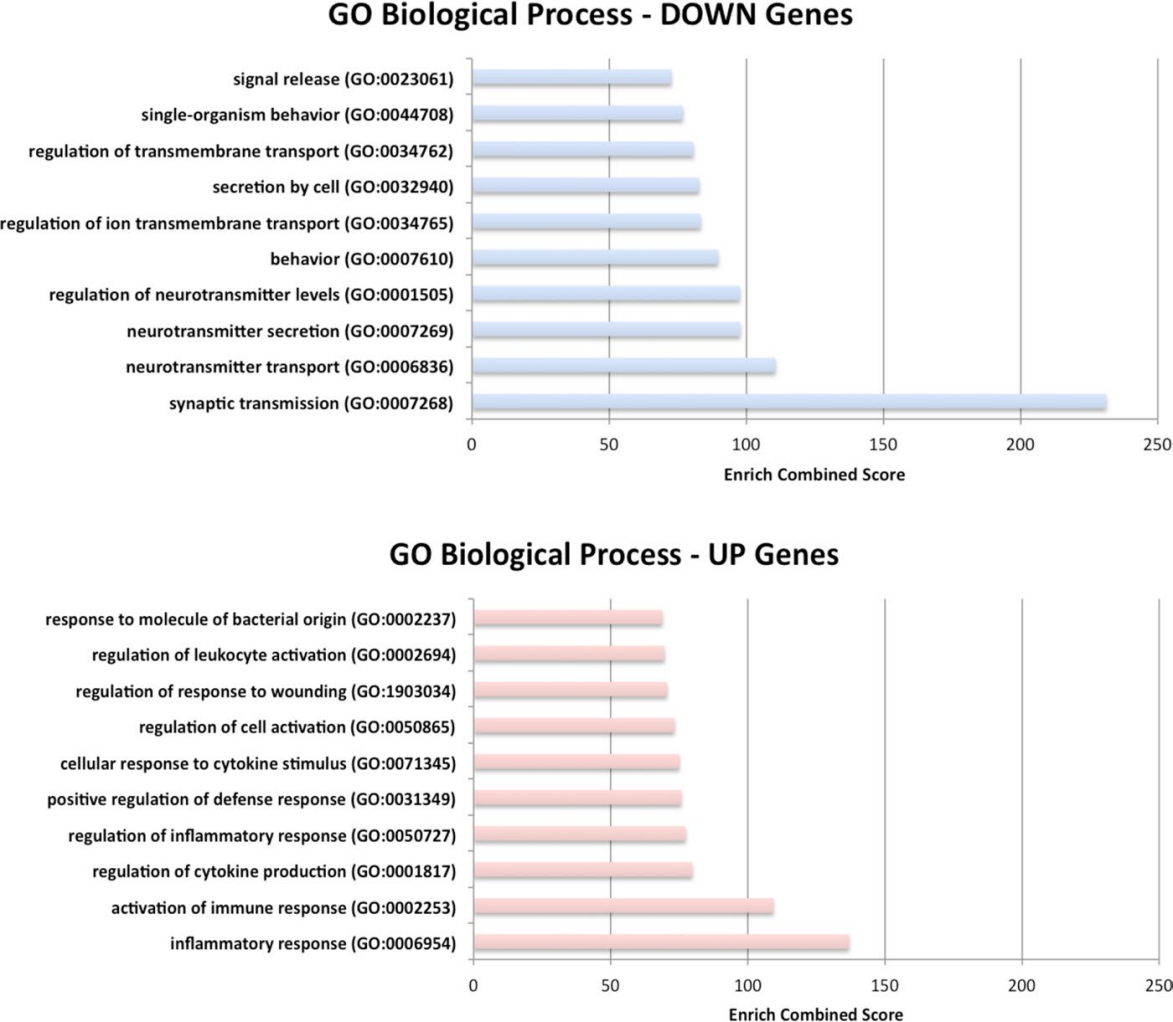
Gene Ontology (GO) analysis of differentially expressed genes. Diagram of GO (Biological Process) terms that are significantly enriched in differentially expressed genes. Categories in the upper part of the figure are enriched in down regulated genes in ALS. Categories in lower part of the figure are enriched in up regulated genes in ALS. The Enrich combined score is reported for each category^66^.

Differential gene expression between ALS and control samples was performed using only unique and concordant RNA-Seq reads. To improve the computational detection of differentially expressed (DE) genes, we applied two independent tools: CuffDiff2 based on FPKM metric that accounts for both uncertainty resulting from read mapping ambiguity and cross-replicate variability^17^, and DESeq2 based on counts of reads in genes^18^. The final list of DE genes was selected by the intersection between CuffDiff2 and DEseq2 lists of significant DE genes and taking into account only genes with |log2(FC)| higher than 1. On the whole, we identified 1142 deregulated genes, for some of them a specific cellular origin is known from the literature (Supplementary Table 2), with log2(FC) values highly correlated between CuffDiff2 and DEseq2 (Pearson r = 0.98) (Fig. 3 and Supplementary Table 2). Of these, 559 (49%) were upregulated in ALS samples and mainly involved in neuroinflammation or activation of immune response (Fig. 4, Supplementary Table 3). Fifty-seven upregulated genes were also found deregulated in a previous study based on peripheral blood of ALS patients^19^ and, thus, they may represent a precious source of potential biomarkers.

Downregulated genes account for the 51% (583) of all DE genes and were significantly enriched (P ≪ 10^−4^ hypergeometric test) in neuronal-specific genes (Fig. 4 and Supplementary Tables 4 and 5). Consistent with past investigations, down expressed genes were involved in several biological processes of nervous system as impulse transmission (SLC1A2, CHAT, SLC12A5, HTR2C) or synapse function (SNPH, SYT4, SNAP25, STX1B) or calcium metabolism (GRIN1, GRIN2A, CACNA1G) (Fig. 4 and Supplementary Tables 2 and 4). RNA-Seq analysis revealed also downregulation of key enzymes in cholesterol biosynthesis including HMGCR (3-hydroxy-3-methylglutaryl-CoA reductase), HMGCS1 (3-hydroxy-3-methylglutaryl-CoA synthase 1), MSMO1 (methylsterol monooxygenase 1) and SQLE (squalene epoxidase), which may affect the organization of cellular membranes and, in turn, modify axon guidance and synaptic transmission (Fig. 4 and Supplementary Tables 2 and 4).

Within the group of down-regulated genes we found a set of 15 synaptic proteins related to the synaptic vesicle cycle (P ≪ 0.0001 hypergeometric test) that consists of docking and fusion of the vesicles, and subsequent exocytosis and neurotransmission (Table 3). Changes in synaptic transmission have been investigated in Alzheimer’s disease^20^ and may have a primary role in ALS for which little is known. Our results indicate deregulation of key genes for synaptic function including the two neuronal t-SNARE proteins, SNAP25 and STX1B. Recent evidence suggests novel functions for SNAP25 in controlling calcium dynamics and enhancing glutamate release in neurons^9, 10^, consistent with a role for SNAP25 in ALS pathogenesis.

**Table 3:** DE genes linked to synaptic function.

Gene	CuffDiff2					DESeq2		
	CTRL	ALS	log2(fc)	pvalue	padj	log2(fc)	pvalue	padj
CACNA1B	4,755	2,003	−1,247	5E-05	0,00071	−1,070	0,00013	0,00175
CPLX1	33,535	5,542	−2,597	5E-05	0,00071	−2,111	0,00000	0,00000
CPLX2	26,962	6,223	−2,115	5E-05	0,00071	−1,528	0,00064	0,00611
DNM1	32,800	7,355	−2,157	7E-04	0,00650	−1,614	0,00007	0,00116
NSF	26,828	8,603	−1,641	5E-05	0,00071	−1,379	0,00016	0,00208
RAB3A	33,164	6,083	−2,447	5E-05	0,00071	−1,849	0,00001	0,00027
RIMS1	11,579	3,910	−1,566	5E-05	0,00071	−1,408	0,00000	0,00000
SLC17A6	4,820	1,057	−2,190	5E-05	0,00071	−1,432	0,00261	0,01776
SLC18A3	17,019	1,829	−3,218	5E-05	0,00071	−2,409	0,00000	0,00000
SLC32A1	6,263	1,280	−2,290	5E-05	0,00071	−1,674	0,00015	0,00197
SNAP25	232,315	36,663	−2,664	5E-05	0,00071	−1,741	0,00031	0,00352
STX1B	20,025	4,881	−2,037	5E-05	0,00071	−1,665	0,00002	0,00032
STXBP1	42,523	16,587	−1,358	5E-05	0,00071	−1,225	0,00001	0,00025
UNC13A	3,875	1,350	−1,521	5E-05	0,00071	−1,318	0,00000	0,00002
UNC13C	5,286	2,190	−1,271	5E-05	0,00071	−1,187	0,00000	0,00012

List of DE genes linked to synaptic function. Per each gene we report: gene name, mean expression levels (in FPKM) in control and ALS groups (from CuffDiff2), log2(fc), P-value and corrected P-value calculated by CuffDiff2 and log2(fc), P-value and corrected P-value calculated by DESeq2.

SLC1A2 which encodes a glutamate transporter essential for terminating the postsynaptic action through the glutamate clearance, appeared negatively regulated in ALS. This finding was also consistent with previous investigations showing down regulation of human SLC1A2 protein in ALS spinal cord^21^.

Fourteen out of 126 genes associated with ALS (mostly by mutation or GWAS studies) from the specialized ALSoD database^14^ showed differential expression in our ALS samples, 10 were down-regulated (including PRPH, UNC13A, SLC1A2, SNCG and NEFH) and 4 were up-regulated (SOD2, APOE, LOX and RNASE2).

Mitochondrial dysfunction is common in ALS pathogenesis since mitochondria have a central role in intracellular energy production, calcium homeostasis and control of apoptosis^22^. Indeed, we found several differentially expressed genes correlated with mitochondrial functions. However, the expression levels of genes transcribed by the mitochondrial genome were unchanged, indicating that mitochondrial transcription may not be impaired in ALS (Supplementary Table 6). Nonetheless, mutations in the mitochondrial genome may occur due to oxidative stress - a hallmark of motor neuron injury^22^.

### Pathway analyses

Differentially expressed genes were further analyzed through the IPA tool (Ingenuity^®^ Pathway Analysis, www.ingenuity.com) in order to identify the most significantly up-regulated and down-regulated canonical pathways associated to neurodegeneration in ALS.

**Figure 5:**
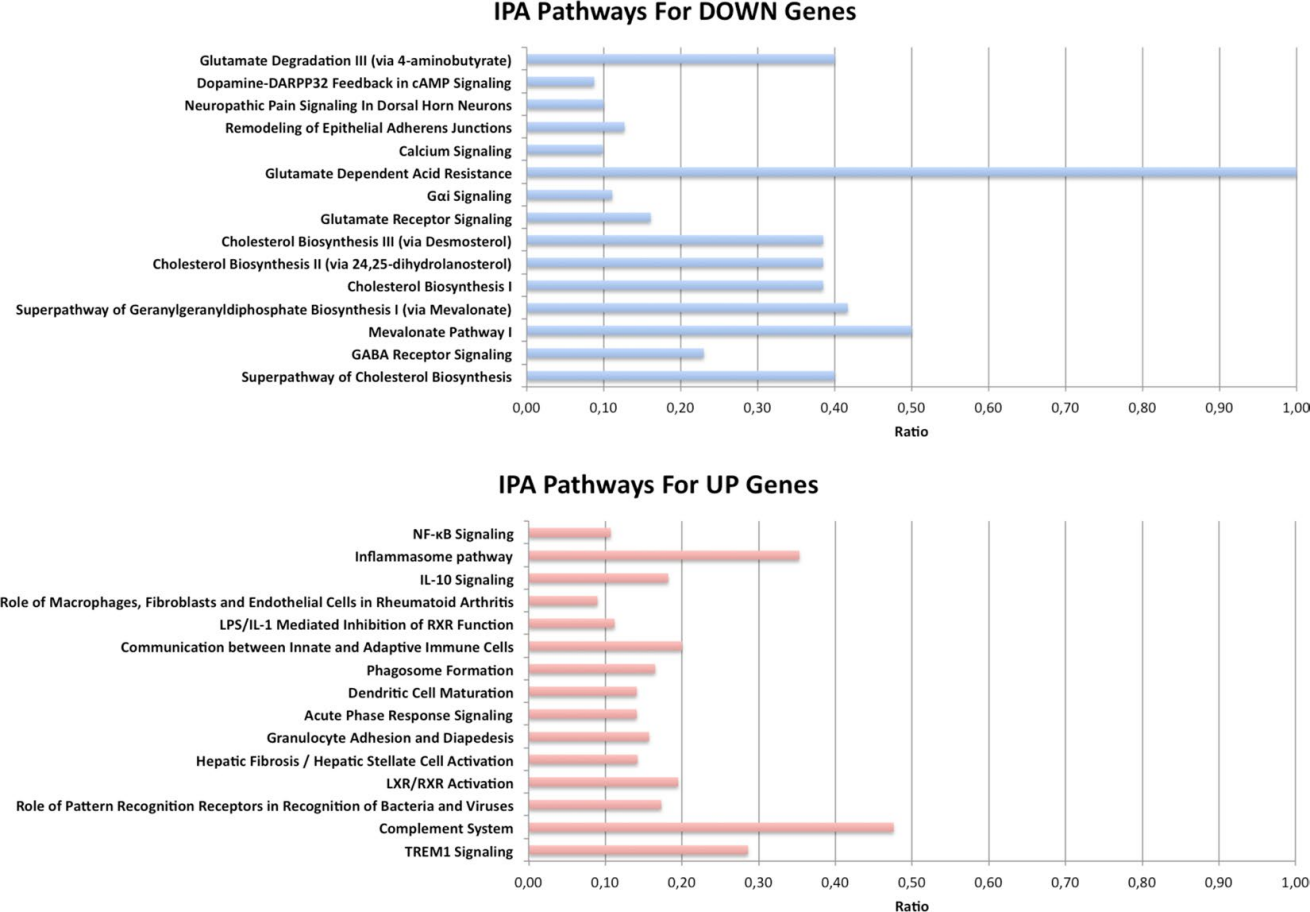
Pathway analysis of differentially expressed genes. IPA pathways that are significantly enriched in differentially expressed genes. Pathways in the upper part of the figure are enriched in down regulated genes in ALS. Pathways in the lower part of the figure are enriched in up regulated genes in ALS. The ratio between the number of differentially expressed genes and the number of genes in the specific pathway is reported on x axis.

Consistent with other transcriptome investigations in transgenic SOD1 mice and human samples, the most highly activated pathways were related to neuroinflammation and immune response (Fig. 5 and Supplementary Table 7). Indeed, our dataset included many up-regulated genes encoding inflammatory mediators such as chemokines and interleukins and several components of the complement system, oncostatin M signaling and STAT3 pathways (Supplementary Table 7).

Significantly down-regulated pathways, instead, included cholesterol biosynthesis, calcium signaling, glutamate receptor signaling and GABA receptor signaling (Fig. 5 and Supplementary Table 7). In addition, several under expressed genes in ALS samples were associated with neurological and neurodegenerative human disorders such as bipolar disorder, schizophrenia, Huntington’s disease or Alzheimer’s disease (Supplementary Table 8). IPA analysis revealed also a significant enrichment for amyotrophic lateral sclerosis signaling, involving nine genes encoding mainly for calcium channels and glutamate receptors (Supplementary Table 8).

Our results were further corroborated by GO-PCA software implementing an unsupervised method to explore gene expression data using prior knowledge in the form of gene ontology (GO) annotations^23^. GO-PCA is able to detect specific signatures, consisting of small set of genes that are strongly correlated by their expression as well as functionally related by GO annotations^23^.

We found 10 significant signatures (P ≪ 0.001) containing between 5 and 10 genes (Table 4). Five signatures, including down-regulated genes, were derived from GO terms related to cholesterol biosynthesis, glutamate secretion, motor activity or axon functionality (Table 4). Remaining five signatures, instead, were associated to inflammation and comprised up-regulated genes (Table 4).

**Table 4:** GO-PCA analysis.

Label	Status	Gene set ID	P-value	Median correlation	Genes
MF: motor activity [5/5]	UP	GO:0003774	0,0000012	0,98	DYNC1I1, KIF3A, KIF3C, KIF5A, KIF5C
CC: microtubule [7/10]	UP	GO:0005874	0,0000073	0,99	KCNAB2, KIF3A, MAPRE3, REEP2, TUBA4A, TUBB2A, TUBB3
BP: glutamate secretion [5/8]	UP	GO:0014047	0,00016	0,96	CPLX1, RAB3A, SLC1A2, SNAP25, STXBP1
BP: cholesterol biosynthetic process [7/10]	UP	GO:0006695	0,000026	0,92	ACLY, CYP51A1, DHCR24, HMGCS1, IDI1, MSMO1, SQLE
CC: axon [10/21]	UP	GO:0030424	0,0001	0,99	CPNE6, EPB41L3, KCNA1, KCNAB2, NEFH, NEFL, NEFM, PTPRN, RAB3A, SCN1B
BP: neg. regulation of viral process [5/8]	DW	GO:0048525	0,0000011	0,43	FBLN1, IFITM1, IFITM2, IFITM3, SLPI
CC: blood microparticle [8/16]	DW	GO:0072562	3,9E-09	0,7	ANGPTL4, APOE, C1QB, C1QC, C3, CP, SERPINA3, STOM
BP: reactive oxygen species metabolic process [6/9]	DW	GO:0072593	0,000013	0,49	CYBA, CYBB, GPX1, GPX3, PDK4, SOD2
BP: receptor-mediated endocytosis [5/17]	DW	GO:0006898	0,00042	0,82	APOE, CD163, FCER1G, ITGB2, MSR1
BP: pos. regulation of inflammatory response [6/10]	DW	GO:0050729	0,00061	0,7	HYAL2, OSMR, S100A9, TLR2, TNFRSF1A, VAMP8

Significant GO signatures detected by GO-PCA tool.

### qRT-PCR validation

We validated RNA-Seq results performing quantitative Reverse Transcription PCR (qRT-PCR) assay using RNA extracted from the ventral horns of the lumbar spinal cord in an enlarged sample group (Table 1). We included donors assayed by RNA-Seq (5 controls and 6 ALSs) and an independent group of 2 controls and 3 ALSs (Table 1). From the list of differentially expressed genes, we selected 16 candidates including genes known to be deregulated in ALS such as INA, HECW1 and SLC1A2 (representing a sort of internal control) and genes not yet described as altered in ALS but associated to significantly disrupted pathways such as the cholesterol biosynthesis, the complement system or the synaptic transmission (Table 5). All genes belonging to deregulated pathways were randomly selected (with |log_2_FC| ≥ 1), except for SNAP25 and STX1B involved in the t-SNARE complex mediating the neurotransmitter release at the presynaptic membrane.

**Table 5:** List of qRT-PCR validated genes and TaqMan Assay IDs.

Gene Symbol	Description	Assay ID
APOC1	apolipoprotein C-I	Hs00155790_m1
ATP2B3	ATPase, Ca++ transporting, plasma membrane 3	Hs00222625_m1
CHD5	chromodomain helicase DNA binding protein 5	Hs01086457_m1
MYT1L	myelin transcription factor 1-like	Hs00903951_m1
STX1B	syntaxin 1B	Hs01041315_m1
SNAP25	synaptosomal-associated protein, 25 kDa	Hs00938962_m1
CTSS	cathepsin S	Hs00175407_m1
SOD2	superoxide dismutase 2, mitochondrial	Hs00167309_m1
SLC1A2	solute carrier family 1 (glial high affinity glutamate transporter), member 2	Hs01102423_m1
GPX3	glutathione peroxidase 3	Hs01078668_m1
HMGCR	3-hydroxy-3-methylglutaryl-CoA reductase	Hs00168352_m1
HMGCS1	3-hydroxy-3-methylglutaryl-CoA synthase 1 (soluble)	Hs00940429_m1
INA	internexin neuronal intermediate filament protein, alpha	Hs00190771_m1
SERPINA3	serpin peptidase inhibitor, clade A (alpha-1 antiproteinase, antitrypsin), member 3	Hs00153674_m1
HECW1	HECT, C2 and WW domain containing E3 ubiquitin protein ligase 1	Hs00389648_m1
CD48	CD48 molecule	Hs00914738_m1
GAPDH*	glyceraldehyde-3-phosphate dehydrogenase	Hs99999905_m1
HPRT1*	hypoxanthine phosphoribosyltransferase 1	Hs99999909_m1
PGK1*	phosphoglycerate kinase 1	Hs99999906_m1
POLR2A*	polymerase (RNA) II (DNA directed) polypeptide A, 220 kDa	Hs00172187_m1

List of genes selected for qRT-PCR validation reporting the TaqMan Assay ID per gene symbol and the corresponding description. Genes indicated by * have been used as internal controls.

**Figure 6:**
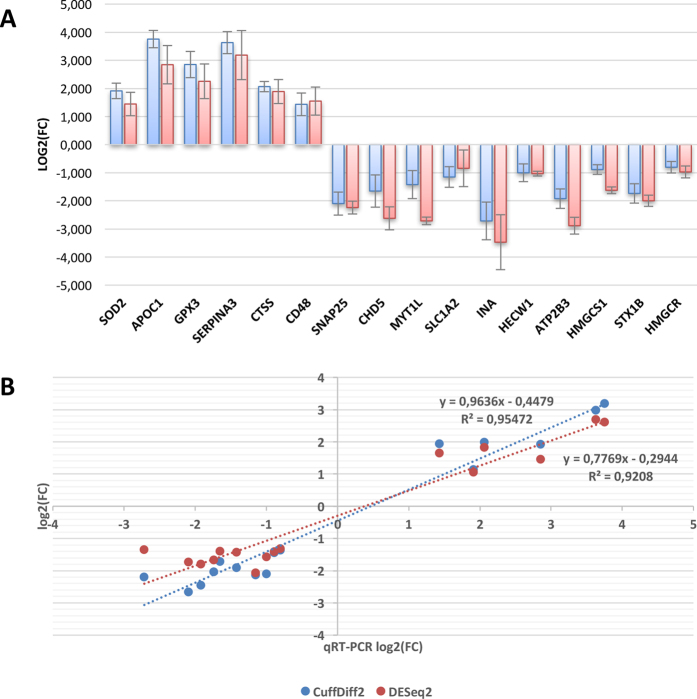
Results from qRT-PCR validation. (**A**) Results from qRT-PCR validation of 16 selected DE genes (Table 3) in the input sample group of 6 controls and 5 Als patients (blue bars), and in an independent group of 2 controls and 3 Als (red bars). Up and down regulation are in respect to ALS donors. (**B**) Linear correlation between log2(FC) values computed by CuffDiff2 (blue circles) or DESEq2 (red circles) on RNA-Seq data and log2(FC) values detected by qRT-PCR for 16 selected genes.

The qRT-PCR analysis confirmed the differential expression emerged in RNA-seq analyses for all 16 genes (Fig. 6A) and the estimates of fold change in expression level were highly consistent with those from RNA-Seq (r = 0.98, P = 8.3 × 10^−11^ for CuffDiff2 and r = 0.96, P = 4.2 × 10^−9^ for DESeq2) (Fig. 6B).

### Aberrant Alternative Splicing

RNA-Seq read alignments were further investigated to identify differential splicing between control and ALS samples. Using MATS^24^, a software tools implementing Bayesian multivariate analysis of transcript splicing, we discovered 794 aberrant splicing events (Table 6). Of these, 98% involved exon skipping (including mutually exclusive exons) and 2% altered donor or acceptor splice sites and intron retention (Table 6).

**Table 6:** Differential alternative splicing detected in RNA-Seq data by MATS program.

Event Type	N	S
SE	72689	601
MXE	18275	178
A5SS	1004	5
A3SS	1205	2
RI	452	8
Total	93625	794

For each alternative splicing type we repot the number of events in UCSC database (N) and the number of significant events (S). SE: skipped exon; MXE: mutually exclusive exon; A5SS: alternative 5′ splice site; A3SS: alternative 3′ splice site; RI: retained intron.

IPA analysis of gene targets for differential splicing revealed perturbations in genes with important neuronal functions (Supplementary Table 9). In particular, we found genes involved in the actin cytoskeleton signaling, which plays central roles in dynamic processes such as cell motility or axon guidance. GNRH signaling, which coordinates the levels of hormones in the hypothalamic-pituitary-gonadal axis and promotes the activation of G-Proteins, PLC, PKC or the mobilization and influx of calcium was also enriched in genes showing differential splicing as well as genes involved in triacylglycerol biosynthesis and mitochondrial functions (Supplementary Table 9).

Twenty-one of the genes exhibiting differential exon skipping were previously identified in an exon array analysis of motorneurons in sporadic ALS samples^25^.

Gene Ontology terms highly enriched in differentially spliced genes included: axon guidance, neuron projection guidance, anchoring junction and actin binding (Supplementary Table 10).

### miRNA-Seq analysis

An emerging hallmark of neurodegenerative diseases is the defective RNA metabolism. It involves aberrant alternative splicing as demonstrated also by our RNA-Seq results and deregulated gene expression due to malfunction of miRNA activity^26^. Several previous investigations have documented a crucial role of miRNA gene regulation in the development and function of neurons. Consequently, alterations in the function of miRNA may contribute to neurodegenerative processes. Indeed, specific miRNA alterations have been reported for ALS microglia^27^ and affecting the stability of the low molecular weight neurofilament mRNA^28^.

We performed massive sequencing of the low molecular weight RNA fraction extracted from the same control and ALS donor tissues using in the RNA-seq analysis. Between 2.8 and 4.2 million reads were generated per sample and adaptors were removed using a custom script (adapters were detected in between 94.7–95.7% of reads for each sample) (Supplementary Figure 1). Reads were mapped to the human genome and miRNAs (reads mapping perfectly to annotated miRNAs and miRNA* were counted and assigned to non-redundant annotated miRNA sequences).

The vast majority of reads in the peak from 30–34 nt in length corresponded to the 5′ portions of annotated tRNA genes (consistently from nt1 to the anticodon loop) as observed previously^29^. The peak at 18 nt was comprised, predominantly (>95%), of tRNA derived small RNAs, almost exclusively consisting of few fragments representing positions 1–18 of tRNA-gly and tRNA-ala molecules. None of the aforementioned reads demonstrated statistically significant differential expression between ALS patients and healthy controls.

The peak at 21 and 22 nucleotides was strongly enriched from known miRNAs that were subjected to differential expression analysis. After correction for multiple hypothesis testing, we found 18 dysregulated miRNAs (Padj ≤ 0.05) (Table 7). Of these, 3 were up-regulated and 15 down-regulated in ALS samples.

**Table 7:** MiRNAs differentially expressed in healthy controls and ALS patients.

miRNA	log_2_FoldChange	P-value	Corrected P-value
hsa-mir-577	−3,28	1,46E-10	3,87E-08
hsa-mir-182-5p	−2,70	6,50E-15	3,44E-12
hsa-mir-485-5p	−2,12	8,79E-05	8,45E-03
hsa-mir-124-3p	−1,83	1,05E-05	1,85E-03
hsa-mir-218-5p	−1,68	1,54E-04	1,16E-02
hsa-mir-183-5p	−1,62	3,14E-04	2,08E-02
hsa-mir-873-5p	−1,58	9,80E-04	3,25E-02
hsa-mir-133a	−1,53	6,05E-04	2,29E-02
hsa-mir-487b	−1,45	1,56E-03	4,59E-02
hsa-mir-219-5p	−1,44	9,55E-04	3,25E-02
hsa-mir-409-3p	−1,34	1,12E-03	3,48E-02
hsa-mir-889	−1,23	5,71E-04	2,29E-02
hsa-mir-136-3p	−1,15	1,75E-03	4,87E-02
hsa-mir-410	−1,12	4,02E-04	2,29E-02
hsa-mir-127-3p	−0,95	5,61E-04	2,29E-02
hsa-mir-148a-3p	1,23	4,99E-04	2,29E-02
hsa-mir-155-5p	1,82	9,56E-05	8,45E-03
hsa-mir-221-3p	2,10	4,82E-04	2,29E-02

For each differently expressed miRNA we report the standard name according to MirBase database, the log_2_ fold-change, the P-value and the corresponding corrected P-value as calculated by DESeq.

MiR-155 has previously been shown to be overexpressed in ALS microglial cells^30^ and inhibition of its expression has been shown to increase survival time in a murine ALS model^31^. MiR-485 has been implicated in synaptic formation and maintenance and has been reported to be dysregulated in Alzheimer’s disease and Huntington’s disease^32^, miR-124 is known as a neurodevelopmental regulator^33^, miR-219 required for neural precursor differentiation in zebrafish^34^ and promotes myelination in rats^35^, miR-127 regulates cell proliferation and senescence by targeting BCL6^36^ while miR-136, miR-873 and miR-410 have all been studied in the context of glioma growth, invasiveness and apoptosis^37–39^.

Uncertainties regarding both the reliability of target predictions and the extent of degradation of individual targeted mRNAs *in vivo*, as well as the mixed nature of the tissue samples complicate direct reconciliation of miRNA and putative target expression profiles. The Diana-miRPath tool^40^ recovered a striking set of 6 pathways (p ≤ 10^−16^) enriched in genes with predicted targets for differentially expressed miRNAs: the PI3K-Akt signaling pathway, Axon guidance, Neurotrophin signaling pathway, Focal adhesion, TGF-beta signaling pathway and Insulin signaling pathway (Supplementary Table 11).

### RNA editing analysis

To further characterize the transcriptome of ALS spinal cord samples, we investigated RNA editing limiting our search to A-to-I changes, as these account for over 99% of editing events in recent large-scale human investigations^41, 42^. In particular, for each RNA-Seq sample we explored known A-to-I events stored in the REDIportal database^43^, selecting only genomic positions supported by at least 30 reads and showing a RNA editing level higher than 0.1. Comparing positions in common between healthy and ALS donors, we found a strong correlation of 0,88 (Pval < 10e^−14^ by Spearman correlation test) in RNA editing levels. We also performed the non-metric multidimensional scaling (NMD) analysis on detected A-to-I changes in order to verify the grouping of input samples by RNA editing. Interestingly, we did not find specific ALS and control clusters as expected in case of global RNA editing alteration (Supplementary Figure 2), suggesting that it should not be impaired in ALS spinal cord tissues.

**Figure 7:**
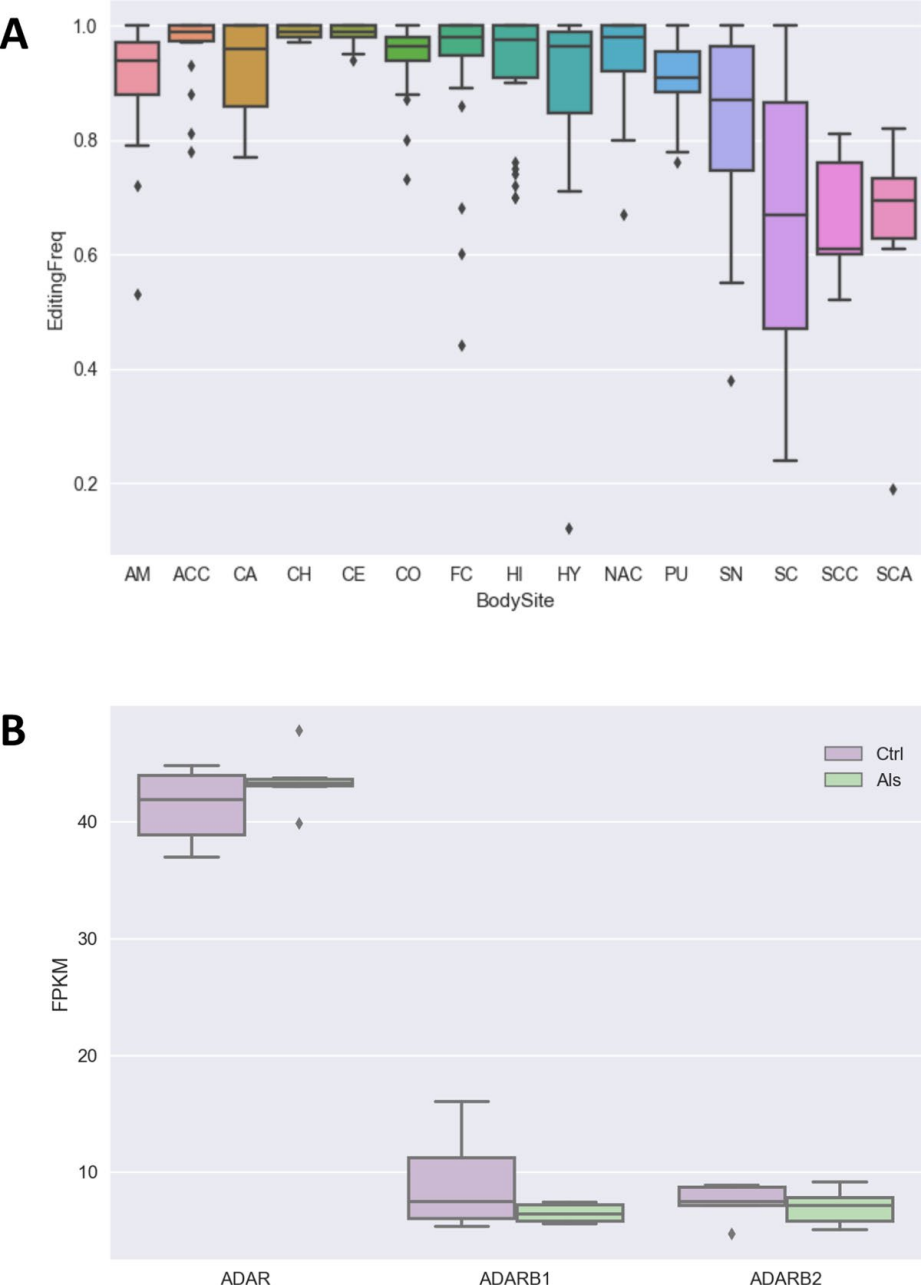
RNA editing levels of GRIA2 Q/R site and the expression of ADAR genes. (**A**) RNA editing levels of GRIA2 Q/R site in spinal cord and other brain locations. Abbreviated body site names are as follow: SC, spinal cord; AM, Amygdala; ACC, Anterior cingulate cortex; CA, Caudate; CH, Cerebellar Hemisphere; CE, Cerebellum; CO, Cortex; FC, Frontal Cortex; HI, Hippocampus; HY, Hypothalamus; NAC, Nucleus accumbens; PU, Putamen; SN, Substantia nigra; SCC, Spinal cord control group; SCA, Spinal cord ALS group. (**B**) Expression levels of ADAR genes from RNA-Seq experiments using the Cuffdiff2 tool. FPKM values for controls and ALS donors are shown as box plots.

We observed reduction of RNA editing levels at the recoding GRIA2 Q/R site, known to be edited at nearly 100% in human brain^44^ and under-edited in motor neurons of ALS donors and, thus, implicated in cell death by excitotoxicity^45^. However, reduced RNA editing levels at the recoding GRIA2 Q/R site were also observed in control samples and the difference between ALS and control groups was not significant (Mann-Whitney P = 0.43). Only one ALS sample showed strong A-to-I reduction level of 0.19 at GRIA2 Q/R site. Additionally, we compared our RNA editing levels of GRIA2 Q/R site with corresponding values from spinal cord and other brain locations of an independent group of healthy individuals from GTEx project and stored in our REDIportal database. Interestingly, GRIA2 Q/R site appeared highly edited in almost all brain locations (average editing levels from 0.82 to 0.99), while editing levels of spinal cord samples (n = 15, average editing levels of 0.66) were quite similar to our values for both ALS (average editing levels of 0.62) and control (average editing levels of 0.66) groups, meaning that RNA editing levels at GRIA2 Q/R site are not always edited at nearly 100% in spinal cord (Fig. 7A and Supplementary Table 12). Editing frequencies of GRIA2 Q/R site in spinal cord samples were always significantly lower than corresponding values from all tested brain locations (Mann-Whitney P < 0.05) (Table 8).

**Table 8:** Comparison between editing frequencies at GRIA2 Q/R site in spinal cord and other brain locations.

SpinalCord	BrainBodySites	N. SC	N. BodySites	U-Stat	Pval
SC	ACC	15	22	56,5	0,00031
SC	NAC	15	27	79,5	0,00057
SC	CH	15	28	64,5	0,00007
SC	CO	15	30	94,5	0,00085
SC	PU	15	19	70,5	0,00647
SC	CA	15	29	88	0,00058
SC	AM	15	21	80,5	0,00699
SC	CE	15	32	76,5	0,00007
SC	FC	15	28	87,5	0,00073
SC	HI	15	26	82	0,00098
SC	SN	15	18	86,5	0,04087
SC	HY	15	24	91	0,00513
SC	SCC	15	5	35	0,43058
SC	SCA	15	6	43	0,45344

Statistical significance was assessed by Mann-Whitney U Test (U-Stat and Pval columns). N. SC indicates the number of samples in spinal cord from GTEx project, N. BodySites report the number of samples per each brain body site from GTEx project. Abbreviated body site names in BrainBodySites are as follow: SC, spinal cord; AM, Amygdala; ACC, Anterior cingulate cortex; CA, Caudate; CH, Cerebellar Hemisphere; CE, Cerebellum; CO, Cortex; FC, Frontal Cortex; HI, Hippocampus; HY, Hypothalamus; NAC, Nucleus accumbens; PU, Putamen; SN, Substantia nigra; SCC, Spinal cord control group; SCA, Spinal cord ALS group.

Further, we explored RNA editing in miRNAs employing massive sequencing of the low molecular weight RNA. We found 12 A-to-I changes in 12 miRNAs (Supplementary Table 13). Of these, 9 were known as edited in the literature, while 3 appeared as novel RNA editing events. All detected changes were observed in both control and diseased donors, even though RNA editing levels were not significantly different (Supplementary Table 13).

Finally, we explored expression values of ADAR genes but no significant differences were detected between healthy and ALS tissues (Fig. 7B).

### Immunoblotting and Immunofluorescence of synaptic SNARE proteins in ventral horns of ALS donors

**Figure 8:**
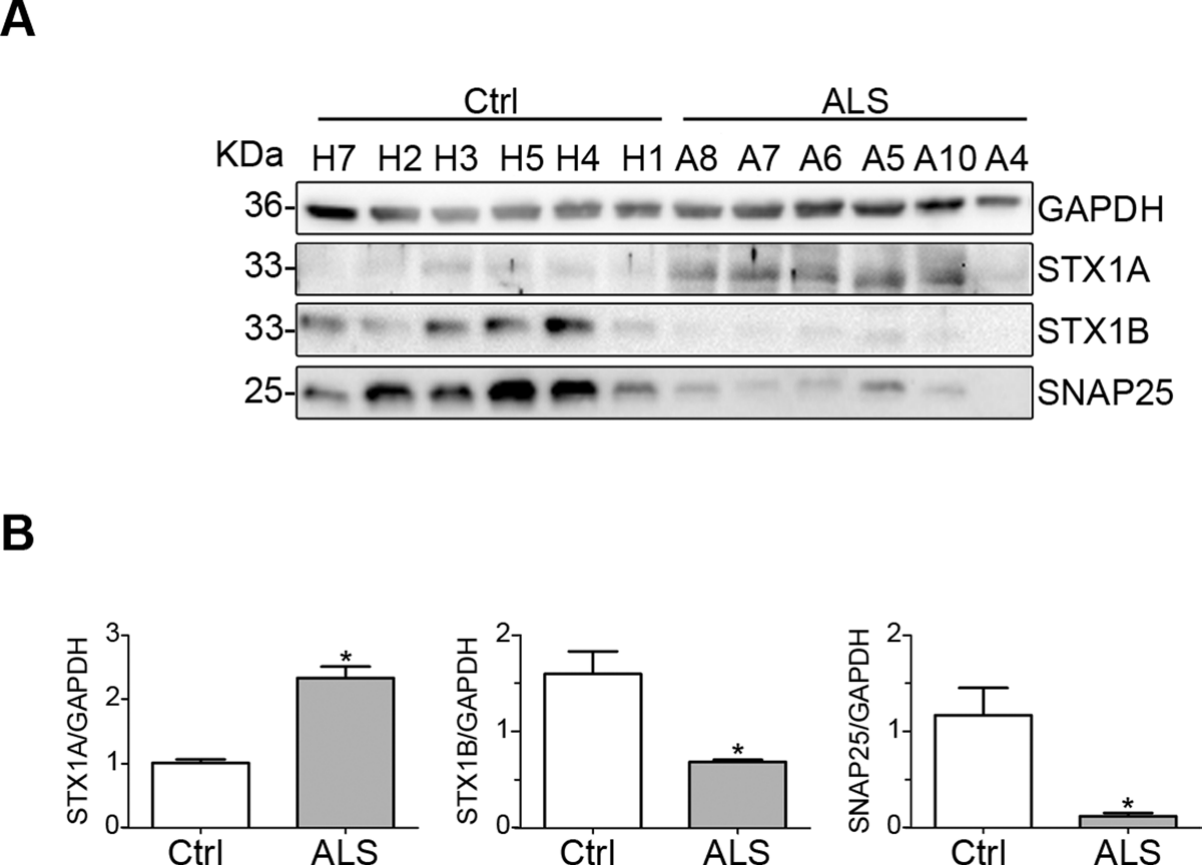
Immunoblot analysis of SNARE proteins in control (Ctrl) and ALS patient ventral horns. (**A**) Ventral horn samples were examined for STX1A, STX1B and SNAP25 expression by immunoblotting. GAPDH was used as internal control for protein loading. (**B**) Histograms summarizing the densitometry analysis of the protein bands shown in A, calculated as each SNARE/GAPDH expression ratio, as indicated (n = 6 for Ctrl and n = 6 for ALS; *P < 0.002). Samples are indicated by their Bank ID as reported in Table 1. The image in A was cropped for clarity from the original in Supplementary Figure 3.

We corroborated our RNA-Seq and qRT-PCR results performing immunoblotting of two synaptic SNARE proteins, SNAP25 and STX1B, since they might provide new insights into neurotransmission and calcium homeostasis alterations in ALS. Western blot analysis was performed on ventral horn of six patients with sporadic ALS and six controls to quantify the protein expression levels of SNAP25 and STX1B. Results obtained showed that SNAP25 and STX1B were significantly downregulated at protein level in ALS patients, in line with the mRNA data (Fig. 8 and Supplementary Figure 3). Since a functional redundancy between STX1B and its homologous STX1A has been reported^46^, STX1A protein expression levels were also analyzed in parallel. Results revealed an opposite trend for STX1 A being significantly upregulated in ALS patients (Fig. 8 and Supplementary Figure 3), as observed in FUS-silenced motor neurons^47^. Such upregulation appeared more prominent in five out of six ALS patients.

**Figure 9:**
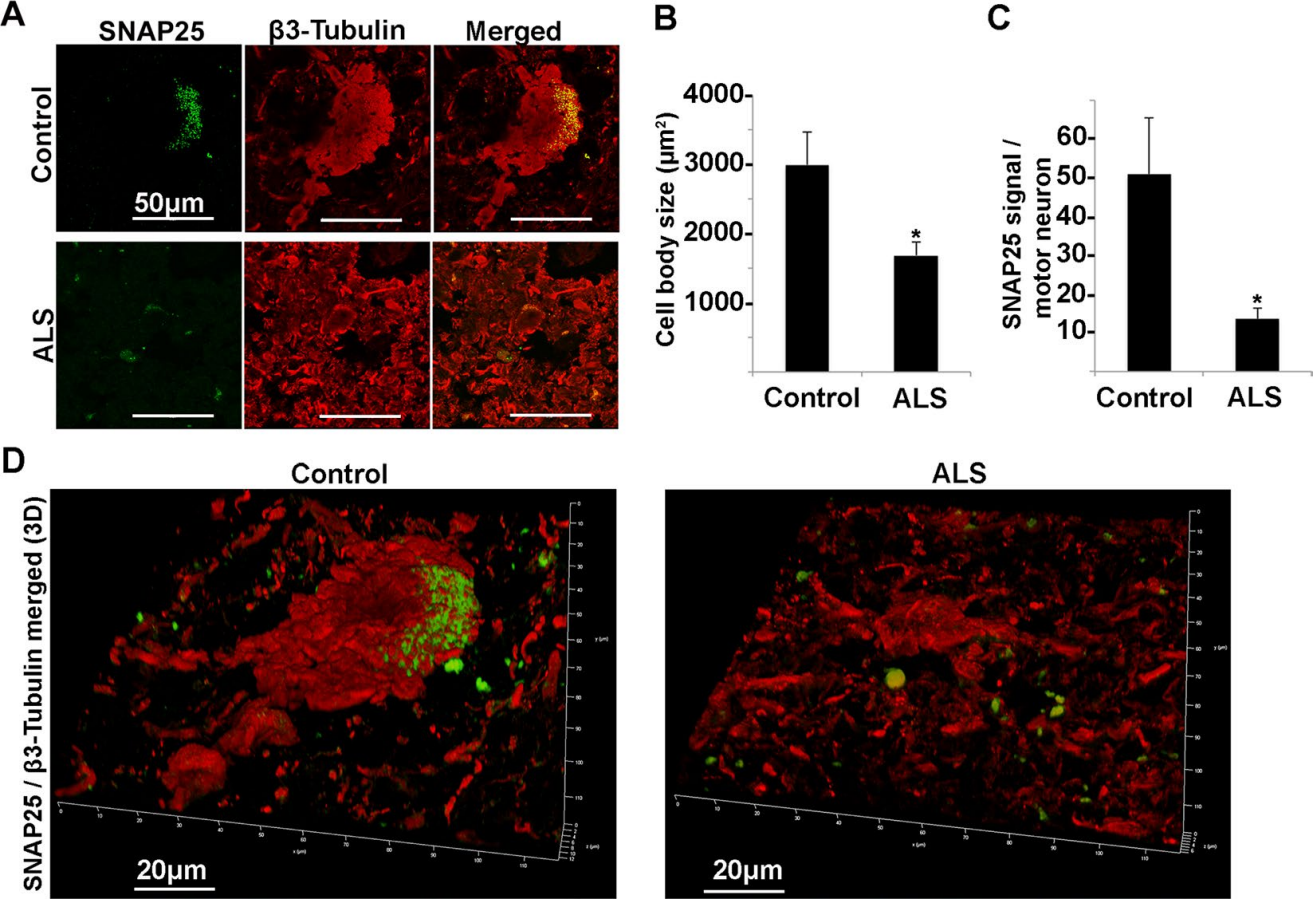
Confocal microscopy analysis of SNAP25 and β3-tubulin in spinal cord ventral horns of control and ALS patients. (**A**) Confocal microscopy images of SNAP25 (green) and β3-tubulin (red) of a typical motor neuron in control and ALS patient. (**B**,**C**) Histograms showing the motor neuron body size expressed in µm2 (**B**) and the quantitative analysis of SNAP25 signal normalized on motor neuron size (**C**) of control and ALS patients (*P < 0.05). (**D**) Confocal 3D reconstruction of SNAP25 and β3-tubulin staining

**Figure 10:**
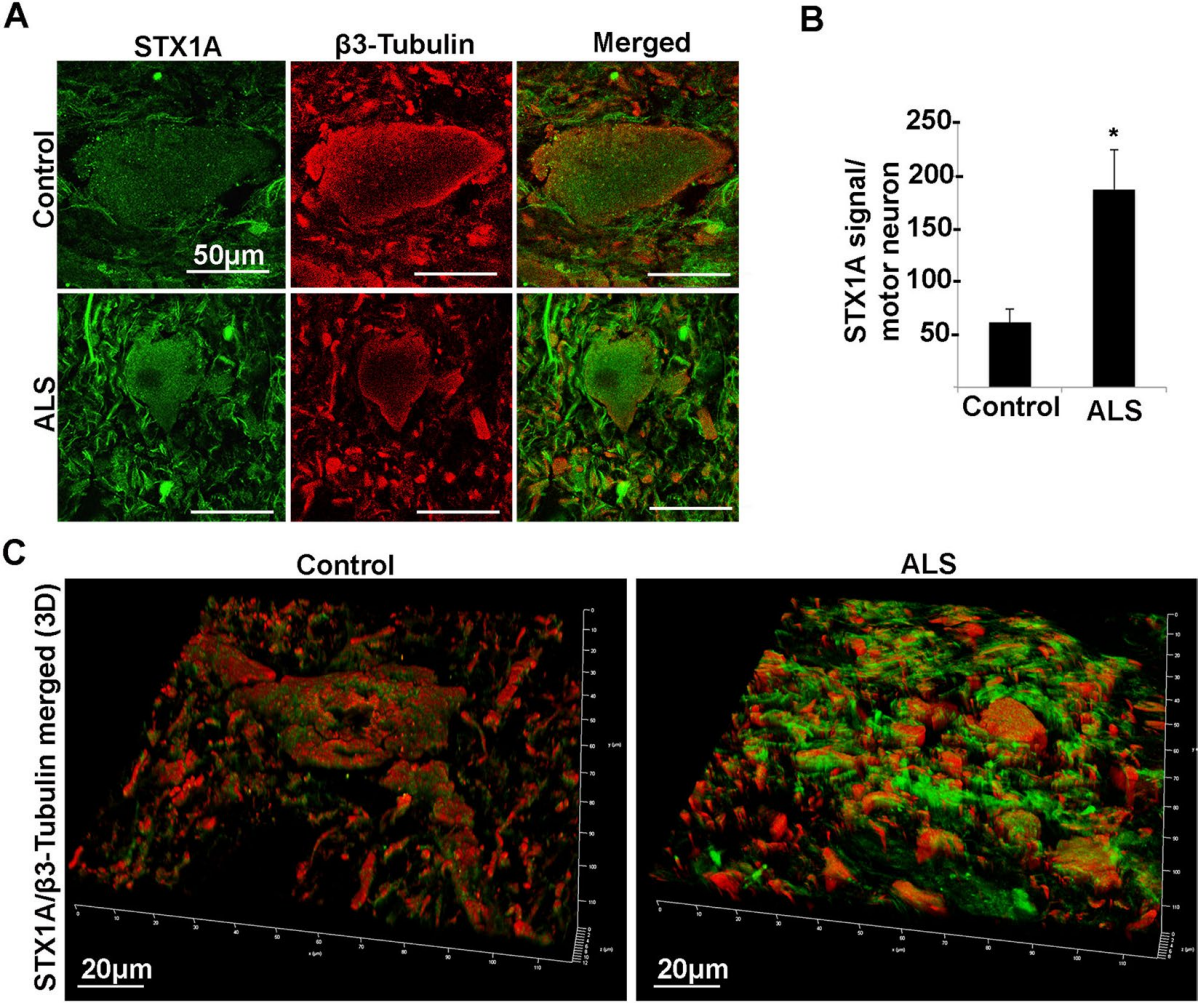
Confocal microscopy analysis of STX1A and β3-tubulin in spinal cord ventral horn of control and ALS patients. (**A**) Confocal microscopy images of STX1A (green) and β3-tubulin (red) of a typical motor neuron in control and ALS patient. (**B**) Histograms showing the quantitative analysis of STX1A signal normalized on motor neuron size of control and ALS patients (*P < 0.05). (**C**) Confocal 3D reconstruction of STX1A and β3-tubulin staining.

Additionally, we investigated in more detail the SNAP25 and STX1A deregulation by immunofluorescence followed by quantification analysis (Figs 9 and 10, Supplementary Figure 4) using confocal microscopy and β3 tubulin as motor neuron marker. The intensity of SNAP25 and STX1A signal was normalized on the size of motor neuron cell body that we found to be reduced by almost 50% in ALS patients (Fig. 9B). In agreement with the SNARE-complex deregulation detected by Western blot (Fig. 8), we found that SNAP25 signal was almost completely absent in ALS patients (Fig. 9), whereas STX1A was significantly upregulated (Fig. 10).

Together, these results validate the RNA-Seq data on the altered expression pattern of genes in ALS and provide new information on the molecular basis of synapsis and calcium handling abnormalities associated with the motor neuron degeneration typical of this pathology.

## Discussion

ALS is a rare and devastating neurodegenerative disease for which no effective therapies are available^1^. Large-scale genetic investigations, at least in the familial form of ALS, have identified several mutations in key genes for motor neuron survival^2^. Nonetheless, the causes of ALS onset and progression are yet elusive. Whole transcriptome analyses have revealed the disruption of a variety of cellular pathways contributing to the elucidation of molecular dynamics characterizing the pathobiology of ALS^3^.

The main obstacle complicating ALS investigations is that motor neurons cannot be sampled during life. As a consequence, animal models and spinal cord tissue from post-mortem donors represent the elective materials for the study of gene expression in ALS. In humans, previous transcriptome studies have been conducted on laser microdissected motor neurons, in order to avoid biases from mixed cell populations. Although motor neurons are the most affected in ALS, other cell types such as astrocytes, microglia and oligodendrocytes may contribute to disease progression. Indeed, co-culture of motor neurons and glial cells has shown an intricate interplay and an active role of non-neuronal cells in neurodegeneration^6^.

In order to characterize whole transcriptome modifications occurring in sALS, we conducted a study in which total RNA from the lumbar spinal cord of ALS donors were sequenced using the RNA-Seq technology.

Our results demonstrate the suitability of deep transcriptome sequencing for capturing molecular changes in the lumbar spinal cord and provide new exciting insights still not revealed by recent whole transcriptome studies based on RNAseq in brain^7^ and cervical spinal cord^8^ from fALS and sALS samples.

Using a dataset of cell type-specific genes derived from various cell types in the CNS^15^ as astrocytes, oligodendrocytes and neurons, our data show a depletion of neuron, motorneuron and oligodendrocyte populations with down regulated genes of neuronal derivation and linked to relevant neurological functions as impulse transmission, synaptic transmission, calcium ion transport or neurotransmitter secretion. Up regulated genes, instead, are generally involved in neuroinflammation, immune responses or cell death and tend to have microglial origin.

RNA-Seq of ALS lumbar spinal cord therefore provides a snapshot of transcriptomic events characterizing the disease, suggesting that neuronal function is compromised through the down regulation of many genes. As a consequence of neurological injury, surrounding cells are activated leading to a cascade of inflammatory events. Although our data do not provide evidence regarding early molecular events causing the death of motor neurons, transcriptomic results would seem to support these speculations. In addition, comparing our results with those by Brohawn *et al*.^8^ in cervical spinal cord of sALS donors using similar technology and methodology, it emerges that neuroinflammation is common in all sections of spinal cord but molecular signatures of neurodegeneration are more consistent in the lumbar rather than cervical spinal region. Our down regulated genes actually include genes already known to be associated to ALS motor neuron death such as INA, HECW1 or SLC1A2.

As a remarkable result, RNA-Seq data support the down-regulation in ALS donors of SNAP25 and STX1B, neuronal t-SNAREs involved in vesicle trafficking and calcium dynamics. Since a redundant role between STX1B and its homologous STX1A has been reported^48^ and being STX1A the isoform interacting with SNAP25 and Voltage Gated Calcium Channels (VGCCs) to control calcium signaling, we have explored the t-SNARE pattern as a whole. In particular, we show that SNAP25 and STX1B are strongly downregulated also at protein level. STX1A, instead, was upregulated in five out of six ALS patients. Through immunofluorescence followed by quantification analysis (Figs 9 and 10) using confocal microscopy and β3 tubulin as motor neuron marker, we confirm the deregulation of SNAP25 and STX1A in ALS patients and demonstrate that the down expression of SNAP25 is mainly due to the reduced size of motor neurons.

Ikemoto *et al*.^49^ have investigated SNAP25 and STX1 expression in ALS spinal cord finding no apparent decrease or only a mild reduction for both proteins in ALS patients. We believe that the immunolocalization approach performed in the study by Ikemoto^49^, without quantification analysis, has most likely led to an underestimation of the t-SNARE reduction in ALS patients. Moreover, the antibody used to detect STX1 was not isoform specific and therefore STX1 unaltered expression reported by Ikemoto^49^ is consistent with the data here reported where STX1A is upregulated and STX1B downregulated. Additionally, upregulation of STX1A and deregulation of SNAP25 splicing were observed also in FUS-silenced motor neurons^47^.

Experiments performed in animal models and primary cultures have shown that loss of SNAP25 impairs calcium-evoked exocytosis leaving almost unaffected spontaneous vesicle release^50, 51^ while deletion of STX1A has no effect on both types of synaptic transmission probably due to compensation by STX1B. Finally, STX1B deletion impairs both spontaneous and evoked fast synaptic vesicle exocytosis^48^.

According to our results on altered t-SNARE protein pattern, SNAP25 and STX1B reduction seem to strictly relate with the motor neuron dysfunction characteristics of ALS ventral horns in which STX1A up regulation may compensate for STX1B loss. Notably, the observed SNAP25 reduction highlights a potentially novel and relevant role of t-SNARE pattern in elevating intracellular calcium concentration and in triggering the glutamate excitotoxicity. Indeed, SNAP25 silencing in glutamatergic neurons has been reported to increase the concentration of intracellular calcium evoked by depolarization^52^. Therefore an initial accident altering the t-SNARE pattern causing SNAP25 reduction should lead to the increased intracellular calcium levels found in ALS motorneurons^12^. In turn, elevated intracellular calcium concentration causes motor neuron injury leading to increased glutamate release and excitotoxicity. In this context, STX1A may compensate for SNAP25 reduction in order to restore calcium homeostasis.

Another remarkable finding of our work is the dysregulation of cholesterol synthesis, which has been linked to chronic neurodegenerative disorders^53^. This alteration could contribute to the impaired axon guidance and synaptic transmission observed in ALS, as cholesterol is essential for the organization and physiological fluidity of cellular membranes.

RNA-Seq also confirms the disruption of RNA metabolism through aberrant alternative splicing potentially resulting from neuronal stress.

Additionally, we investigated RNA editing since its deregulation has been linked to several neurodegenerative diseases including ALS^45^. Indeed, it has been shown that reduced levels of recoding A-to-I editing at GRIA2 Q/R site may be involved in motor neuronal death through an increased Ca^2+^ permeability of AMPA receptor^45^. Our results, based on known RNA editing events, do not support significant alterations. A closer investigation of RNA editing levels at the recoding GRIA2 Q/R site reveals that A-to-I frequencies of GRIA2 Q/R site are not impaired in spinal cord tissue of ALS donors, rather their values are similar to those observed in physiological conditions without altered expression of ADAR enzymes. However, our study was conducted on entire tissues and, thus, we cannot exclude a compensating effect from non-neural cells obscuring specific motor neuron RNA editing alterations.

Small RNA expression analyses suggest important roles for miRNAs regulating signaling pathways determining cell survival and axon guidance during ALS progression. Numerous lines of evidence implicate TGF-beta signaling in motor neuron diseases. It participates in the interplay between neuronal and glial cells during ALS progression^6^ and increased levels of TGF-beta provoke an acute improvement in the motor performance of SOD1 mice^54^. Participants in the TGF-beta signaling pathway predicted to be targets of DE miRNAs include Smad2,4,5 and 7. TDP-43 and phosphorylated Smad2 are co-localized within cytoplasmic inclusions in the anterior horn cells of sporadic ALS patients^55^. Activation of the TGFβ/Smad signaling system is protective against aggregate formation of cytoplasmically mislocalized TDP-43^55^.

The Axon Guidance pathway has recently been proposed to represent a prime effector in ALS pathobiology^56^ and is intimately linked to the regulation of actin cytoskeleton and Focal Adhesion circuits.

The phosphatidylinositol 3-kinase (PI3K) /ATK prosurvival pathway has been implicated as a mediator of the protective effect of vascular endothelial growth factor in G93A-SOD1 neurons *in vitro* and induction of ATK3 is neuroprotective in SOD1_G93A mice^57^. The pathway contributes, in conjunction with mTOR family proteins, to the regulation of the activity of the glutamate transporter GLT1 in astrocytes^58^.

Neurotrophins, including Nerve Growth Factor (NGF), brain-derived neurotrophic factor (BDNF), and Neurotrophins 3 and 4 (NTF3 and NTF4) lie upstream of the Neurotrophin pathway which regulates the axon cytoskeleton (and in turn axon outgrowth/guidance and synapse formation) as well as cell differentiation and survival. NFT3, NTF4, and BDNF, as well as their cellular receptors and numerous downstream genes are all predicted targets of miRNAs dysregulated in ALS. NGF, BDNF, NT-3 are neuroprotective on axotomized extraocular motoneurons in neonatal rats^59^, while 7,8-dihydroxyflavone (7,8-DHF) a small molecule tyrosine kinase receptor B (TrkB) agonist that mimics the effects of BDNF, is neuroprotective in SOD1 mice^60^.

In conclusion, our study remarks the use of massive RNA sequencing technologies to improve deregulated pathways of ALS neurodegeneration and provide new exiting insights as the SNAP25 reduction as possible cause of calcium elevation and glutamate excitotoxicity, suggesting t-SNARE protein expression as novel indicators and potential biomarkers for sporadic ALS.

## Methods

### Post mortem tissues

Frozen lumbar spinal cord samples from 11 male human donors affected by sporadic ALS and 7 age, sex and ethnicity matched controls were obtained from the NICHD Brain & Tissue Bank for Developmental Disorders (University of Maryland - http://medschool.umaryland.edu/btbank/) and the Human Brain and Spinal Fluid Resource Center (Los Angeles, CA). Additionally, we required as an additional criterion, the presence of clinical symptoms associated to the altered function of lower motor neurons.

Each tissue was longitudinally dissected and ventral horns were collected for downstream analyses.

Detailed information regarding samples used in RNA-seq, miRNA-seq, qRT-PCR and western blot analyses are provided in Table 1, also including age, gender, ethnicity and post-mortem interval as well as donor bank IDs to access to further clinical info.

### RNA extraction

RNA was extracted from the ventral horns of the lumbar spinal cord after grinding with mortar and pestle using the mirVana™ miRNA Isolation Kits (Life Technologies Inc., Carlsbad, CA, USA) according to the manufacturer’s instructions to separate total and small RNA fractions. Each RNA sample was treated with RNAse-free DNAse (Life Technologies) and qualitatively and quantitatively checked on Agilent 2100 Bioanalyzer RNA Nano Chip (Agilent, Santa Clara, CA, USA).

### RNA sequencing

Directional RNA-Seq libraries were prepared from 1 μg of total RNA using the TruSeq Stranded Total RNA Sample Prep Kit (Illumina) according to the manufacturer’s protocol. Sequencing was performed on an Illumina NextSeq 500 platform (Illumina, San Diego, CA), generating for each sample from 109 to 219 millions of 100pb × 2 paired-end reads. RNA-Seq statistics are reported in Table 2.

### miRNA sequencing

Indexed cDNA libraries from the RNA fraction at low molecular were prepared using the TruSeq small RNA sample Preparation kit (Illumina, San Diego, CA) according to the manufacturer’s protocol and recommendations. Single end sequencing (1 × 50b), after fluorimetric quantification, was performed on an Illumina Miseq platform.

### Preprocessing and analysis of RNA-Seq reads

RNA-Seq reads in FASTQ format were initially inspected using FASTQC program (http://www.bioinformatics.babraham.ac.uk/projects/fastqc/). Adaptors and low quality regions (phred cutoff of 20) were trimmed using Trim Galore (http://www.bioinformatics.babraham.ac.uk/projects/trim_galore/), excluding reads with final length less than 50 bases.

Cleaned reads were aligned onto the complete human genome (assembly hg19) by means of GSNAP^61^ version 2013-11-27 (using as parameters: -B 5 -d hg19 -t5 -s splicesites -E1000 -N1 -n1 -Q -O-nofails -A sam–force-xs-dir -a paired) providing a list of exon-exon junctions from Ensembl, UCSC and RefSeq databases. Unique and concordant alignments in SAM format were converted in the binary BAM format by SAMtools^62^ and basic statistics were calculated using the CollectRnaSeqMetrics tool of Picard package (https://broadinstitute.github.io/picard/index.html) (Table 2). The sporadic disease nature of our samples was checked using REDItools^13^, screening known ALS mutations from ALSoD database in aligned RNAseq data^14^. Only positions showing a frequency variation higher than 0.1 and supported by more than 10 reads we taken into account.

Differential expression was performed using two independent tools: CuffDiff2 version 2.1.1^17^ (using as main parameters:–library-type fr-firststrand–labels Ctl,Als -u -b hg19.fa refgenes.gtf) and DESeq2 version 1.10.1^18^. Read counts on known human genes were calculated by featureCounts version 1.5.1^63^. Reference human transcriptome was obtained from iGenomes repository (http://cole-trapnell-lab.github.io/cufflinks/igenome_table/) and annotations for rRNA genes were downloaded from UCSC genome browser selecting the RepeatMask table.

DE genes was selected intersecting lists of significant DE genes (corrected P value < 0.05) obtained by CuffDiff2 and DEseq2. We took into account only genes with |log2(FC)| higher than 1.

Changes in cell type composition of spinal cord samples was detected according to the method described in Lin *et al*.^64^ and using cell type specific genes for neurons, motorneurons, astrocytes, oligodendrocytes and microglia collected from the literature^6, 15^. The majority of cell type specific genes were obtained from Cahoy *et al*.^15^ taking into account only genes showing >10-fold enrichment in this study. Since Cahoy *et al*. performed their investigations in mouse, we selected only genes with corresponding human orthologues and showing FPKM values > 0. Motor neuron specific genes, instead, were collected from Phatnani *et al*.^6^.

Differential alternative splicing was detected with MATS version 3.0.9^24^ using default parameters except for the read length (fixed to 90). Events were selected at 0.05 significant level, corrected for multiple testing. Heat-maps and PCA plots were generated using ClustVis^65^.

IPA system was used for pathway analysis. Enrich^66^, instead, was used to generate lists of Gene Ontology terms enriched in the upregulated, downregulated or differentially spliced gene lists, with default setting. To identify sets of genes that are both strongly correlated to expression data and functionally related to their GO annotations, we used the GO-PCA method^23^. In brief, it adopts a two-step approach in which PCA is performed first. Then, each principal component is tested for whether it is driven by functionally related genes (in the form of gene ontology annotations).

### Preprocessing and analysis of miRNA-Seq reads

Raw sequence data were processed with a custom Python script to remove adapters from inserts of between 14 and 38 nt in length and subsequently mapped onto the human genome (version hg19) using Bowtie^67^. Counts of reads exactly matching human miRNAs and (where annotated in miRBase) miRNA* sequences were submitted to the DESeq software^68^ for statistical analysis of differential expression using default parameters and treating different ALS and healthy control patients as biological replicates. Pathway analysis was conducted with miRPath v2.0^40^ with default settings.

### RNA editing detection and analysis

RNA editing candidates per each sample were detected using the REDItools package (https://sourceforge.net/projects/reditools/)^13^. In particular, REDItoolKnown.py was used to explore known RNA editing sites stored in REDIPortal database^43^. Non-Metric Multidimensional Scaling analysis of RNA editing sites was performed in R using the vegan package. RNA editing levels at the GRIA2 Q/R site in different brain regions were extracted from REDIportal^43^. Custom python scripts were used to calculate RNA editing correlation between control and ALS samples.

RNA editing detection in miRNAs was performed according to the protocol developed by Alon *et al*.^69^, while RNA editing levels were calculated by REDItools^13^.

### qRT-PCR validation

Gene expression analyses were carried out on custom TaqMan Array Fast Plates (Life Technologies), configured in order to include four potential human endogenous control genes (GAPDH, HPRT1, PGK1 and POLR2A), and sixteen genes selected for validation (Table 5), each of them in duplicate per sample. Reverse transcription of 500 ng of total RNA was performed using “iScript Reverse Transcription Supermix for RT-qPCR” (Bio-Rad, Hercules, CA, USA). qPCR reactions were set up according to the manufacturer’s instruction of “TaqMan Fast Universal PCR Master Mix” kit (Life Technologies), starting from 1 µl of diluted cDNA (1:3) as template and performed using the ABI PRISM 7900HT platform (Applied Biosystem, Life Technologies) with the following amplification conditions: hot start at 95 °C for 10 min; 45 amplification cycles (95° for 15 sec, 60° for 1 min).

Control reverse transcription reactions (RT-) were also prepared and PCR reactions, using primers for HPRT1 transcript (Forward: 5′-TGACACTGGCAAAACAATGCA-3′; Reverse: 5′-GGTCCTTTTCACCAGCAAGCT-3′), were carried out as control for genomic DNA contamination (data not shown).

Fluorescence raw data were exported from the SDS 2.2.1 software and analyzed using the comparative quantification cycle (Cq) method to determine the relative ratio of transcripts. First, the ΔCt values were obtained by calculating, for each sample, the difference of the Cq of each target compared to the arithmetic mean of the endogenous Cq genes (GAPDH and HPRT1). Then, the relative expression ratio (rER) was expressed as Log2(2^−ΔCt^); rER for each type of samples were averaged and calibrated respect to normal samples. Analyses were carried out using data related to two independent experiments.

### Antibodies

The following primary antibodies were used: goat anti-SNAP25 (SC-7538, Santa Cruz, CA, USA), rabbit anti-Syntaxin-1A (110302, Synaptic System, Goettingen, Germany), rabbit anti-Syntaxin-1B (110402, Synaptic System, Goettingen, Germany), mouse anti-GAPDH (MAB 374, Millipore, Billerica, MA, USA) and mouse anti-β3 tubulin (SC-80016, Santa Cruz Biotechnology, Santa Cruz, CA, USA).

The following secondary antibodies were used for Western blot: HorseRadishPeroxidase (HRP)-conjugated donkey anti-goat, goat anti-rabbit and goat anti-mouse IgG (Santa Cruz Biotechnology, Santa Cruz, CA, USA).

The following secondary antibodies were used for Immunofluorescence: donkey anti-rabbit and donkey anti-goat AlexaFluor488-conjugate IgG (A-21206, Thermofisher, Milan, Italy), and donkey anti-mouse AlexaFluor594-conjugate IgG (A-27027, Thermofisher, Milan, Italy).

### Protein sample preparation and immunoblotting

1 mm slices of Ctrl and ALS ventral horn samples, were collected, cut into small pieces with a razor blade and immediately solubilized in 8 volumes of RIPA buffer (10 mM Tris-HCl, pH 7.4; 140 mM NaCl; 1% Triton X-100; 1% Na deoxycholate; 0.1% SDS; 1 mM Na3VO4; 1 mM NaF and 1 mM EDTA) added with a cocktail of protease inhibitors (Roche, Milan, Italy). The lysis was performed as previously described^70^. Briefly, on ice for 1 h and the samples were then centrifuged at 22,000xg for 1 h. The protein content of the supernatant was measured with a bicinchoninic acid (BCA) Protein Assay Kit (Rockford, IL, USA).

30 ug of protein samples were separated by 13% Tris-Glycine-SDS-PAGE and transferred to polyvinylidene difluoride membranes (Millipore, Milan, Italy) as described previously^71^. Membranes with blotted proteins were incubated with primary antibodies, washed, and incubated with peroxidase-conjugated secondary antibodies. Reactive proteins were revealed with an enhanced chemiluminescent detection system (ECL Plus; GE Healthcare, Buckinghamshire, UK) and visualized on a Versadoc imaging system (BioRad, Milan, Italy). Densitometry analysis was performed using Scion Image software (Frederick, MD, USA).

All data are reported as mean ± SEM. We used the Student’s t test for unpaired data and analysis of variance for multiple statistical comparisons between groups, the significance level being set at P < 0.05. The number of samples is indicated in the figure legend.

### Immunofluorescence

Fresh sections of control and ALS spinal cord samples were rehydrated with PBS and fixed for 10 min with 4% paraformaldehyde in PBS (HT5014, Sigma, Milan Italy). Sections were washed in PBS and incubated with 1:200 diluted anti-SNAP25 and 1:1000 diluted anti-β3 tubulin or 1:200 diluted anti-Syntaxin-1A and 1:1000 diluted anti-β3 tubulin in 0.3% Triton X-100 in PBS overnight at 4 °C. After washings with PBS, sections were saturated with 0.1% Gelatin (Sigma, Milan, Italy) in PBS, and incubated with 1:1000 diluted AlexaFluor488 and AlexaFluor594 conjugated secondary antibodies for 1 hour at room temperature. After washings, sections were washed in PBS and mounted with Mowiol (Sima, Milan, Italy) and acquired using Leica TCS SP2 confocal microscope (Leica Microsystems, Heidelberg GmbH) for the quantitative analysis described in the next paragraph. Confocal 3D reconstruction was obtained using Leica TCS SP8 STED 3X and Leica HC PL APO 100x/1.40 Oil white objective with Type F Immersion liquid with 1.5 refractive index (Pisani *et al*., 2016). Excitation of AlexaFluor488 and AlexaFluor594 dyes was performed with a continuous 488 nm and 594 nm wavelength diode laser with a maxim light output in focal plane of 10 mW (NKT Photonics sign supercontinuum laser). To obtain the confocal 3D projection, 30 planes were acquired in a Z-stack of 6–12 μm, and the series processed by Leica LASX software (Leica Microsystems, Mannheim, Germany).

### Quantitative analysis of motor neuron size and SNAP25 and STX1A expression by immunofluorescence in ventral horn sections

Single confocal planes of randomly chosen areas of ventral horn sections from three control donors and three ALS patients, stained as described above, were acquired by Leica TCS SP2 confocal microscope (Leica Microsystems, Heidelberg GmbH). Leica Confocal Software Version 2.61 was used to measure motor neuron size and to quantify SNAP25 and STX1A expression levels. Motor neuron cell bodies were drawn based on the β3 tubulin staining, and the surface quantified and expressed in µm^2^. The average signal value relative to SNAP25 and STX1A for each motor neuron was also calculated and normalized on the motor neuron surface. Results were expressed as means ± standard error. Statistical significance was evaluated using unpaired Student’s t test. Difference with p < 0.05 were considered statistically significant.

### Data availability

All sequencing data produced in the present work have been submitted to dbGaP database under the accession phs000747.

## Electronic supplementary material

Supplementary Figures and Tables
