# Supplementary material for: Massive transcriptome sequencing of human spinal cord tissues provides new insights into motor neuron degeneration in ALS
Source: Sci Rep. 2017 Aug 30;7:10046. doi: 10.1038/s41598-017-10488-7 (PMC5577269; doi:10.1038/s41598-017-10488-7)
Supplement: Supplementary file 1 — Supplementary Figures and Tables [file 41598_2017_10488_MOESM1_ESM.pdf]

Supplementary Material

Massive transcriptome sequencing of human spinal cord tissues provides new insights into motor neuron degeneration in ALS.

Anna Maria D'Erchia[1,2], Angela Gallo[3], Caterina Manzari[2], Susanna Raho[3], David S. Horner[5], Matteo Chiara[2], Alessio Valletti[2], Italia Aiello[2], Francesca Mastropasqua[1], Loredana Ciaccia[1], Franco Locatelli[3], Francesco Pisani[1], Grazia Paola Nicchia[1], Maria Svelto[1,4,6], Graziano Pesole[1,2,4,6,*] and Ernesto Picardi[1,2,4,*].

[1] Department of Biosciences, Biotechnology and Biopharmaceutics, University of Bari, Via Orabona 4, 70126 Bari, Italy

[2] Institute of Biomembranes, Bioenergetics and Molecular Biotechnologies (IBIOM), National Research Council, Via Amendola 165/A, 70126 Bari, Italy

[3] Department of Pediatric Oncohaematology, Bambino Gesù Children's Hospital IRCCS, Piazza S. Onofrio 4, 00165 Rome, Italy

[4] National Institute of Biostructures and Biosystems (INBB), Viale Medaglie D'Oro 305, 00136 Rome, Italy

[5] Department of Biosciences, University of Milan, Via Celoria 26, 20133 Milan, Italy

[6] Center of Excellence in Comparative Genomics, University of Bari, Piazza Umberto I, 70121 Bari, Italy

Supplementary Figure 1

Distribution of insert size profiles after adaptor removal for miRNA-Seq. Control samples are in A while ALS samples are in B.

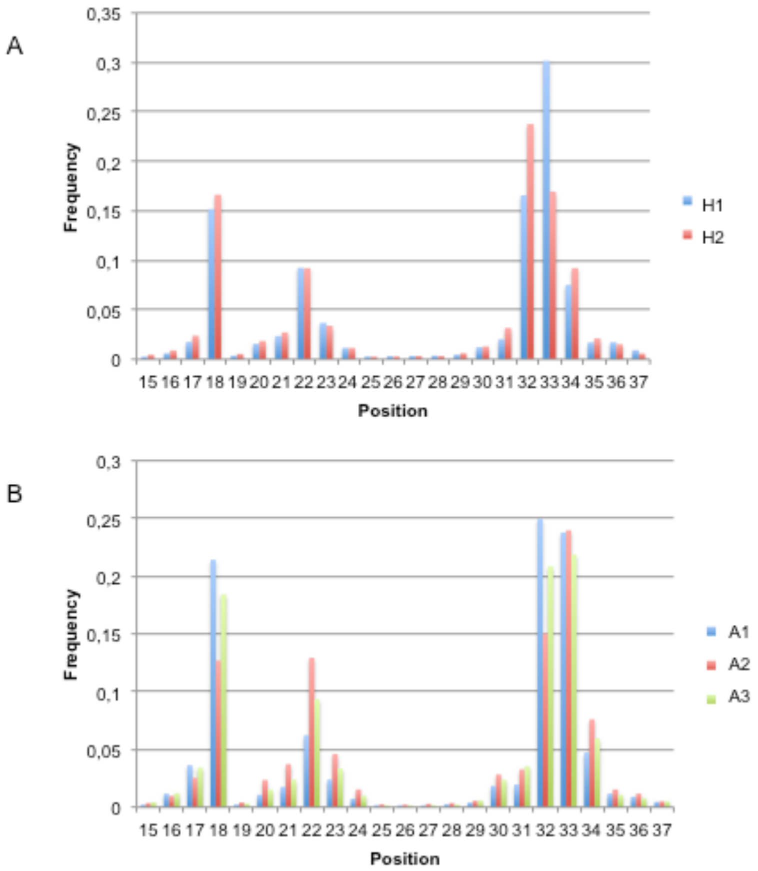

Supplementary Figure 2

Non-metric multidimensional scaling (NMD) of known RNA editing sites from REDIportal database. NMD has been calculated by *vegan* package in R. Control samples are in blue while ALS samples in red.

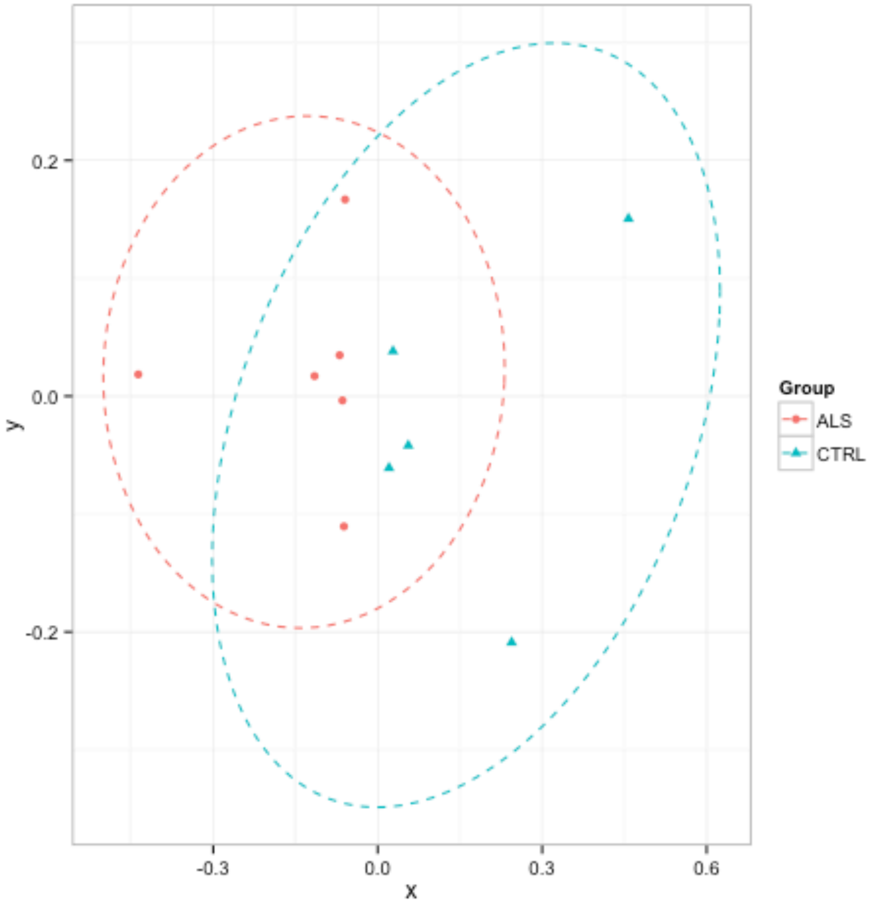

Supplementary Figure 3

Original immunoblotting images used to generate the Figure 8. In A) immunoblotting of SNAP25 protein. First six lanes are controls. In B) immunoblotting of STX1A protein. First six lanes are controls. In C) immunoblotting of STX1B protein. First six lanes are controls. In D) immunoblotting of GAPDH protein. First eight lanes are controls.

The immunoblotting methodology is described in the Methods section.

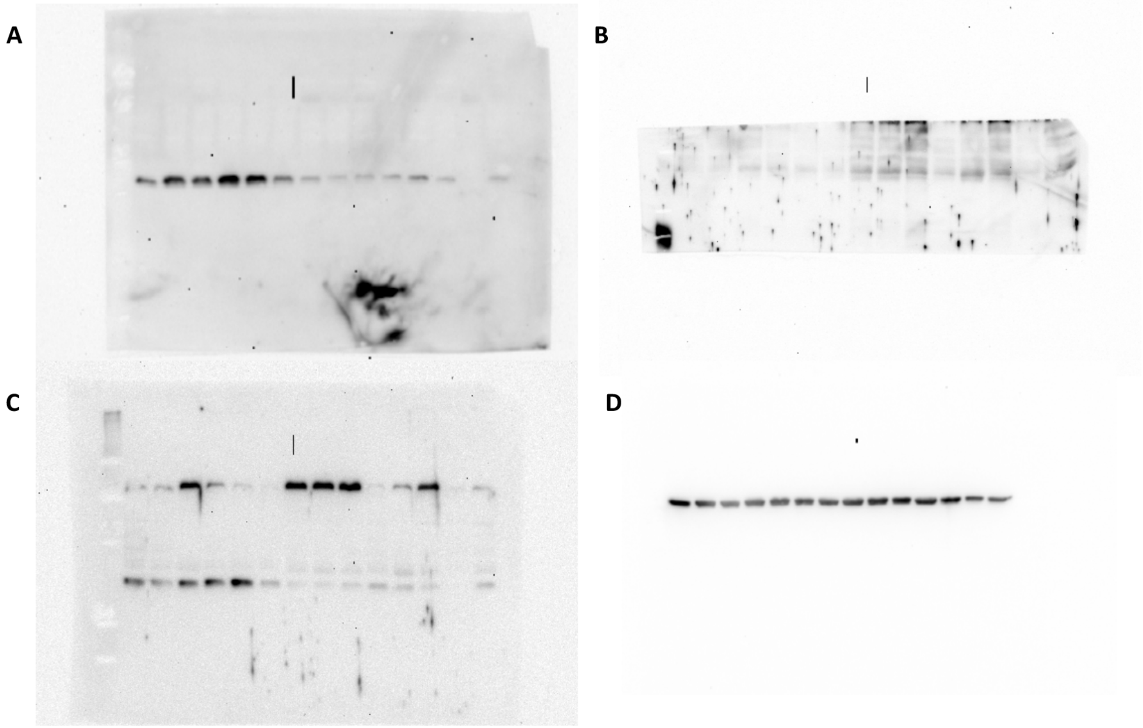

Supplementary Figure 4

Confocal immunofluorescence analysis of SNAP25 expression in control (CTRL) and ALS spinal cord sections (ventral horn) at two different magnifications as indicated by the scale bars. SNAP25 expression is reduced in ALS sample.

Control and ALS spinal cord sections were fixed for 10 min with 4% paraformaldehyde, (Sigma, HT5014, https://www.sigmaaldrich.com), washed in PBS, and incubated with SNAP25 antibodies (HPA 001830, Prestige Antibodies®, SIGMA, http://www.sigmaaldrich.com) diluted in 0.3% Triton X-100 containing PBS, overnight at 4°C. After washings, sections were saturated with 0.1% Gelatin (SIGMA), incubated with Alexa Fluor 488 conjugate secondary antibodies (A-21206, Thermofisher, https://www.thermofisher.com) for 1 hour, washed in PBS, and mounted with Mowiol (SIGMA).

For image acquisition Leica TCS SP8 STED 3X microscope and Leica LASX software were used (Leica Microsystems CMS GmbH, Am Friedensplatz 3, D-68165 Mannheim (Germany). Excitation of Alexa-Fluor488 dye was performed with a continuous wave of 488nm wavelength diode laser with a maxim light output in focal plane of 10mW (NKT Photonics sign supercontinuum laser, http://www.nktphotonics.com). Confocal images were deconvoluted by Huygens Professional Software (Laapersveld 63, 1213 VB Hilversum, The Netherlands, https://svi.nl/HuygensProfessional).

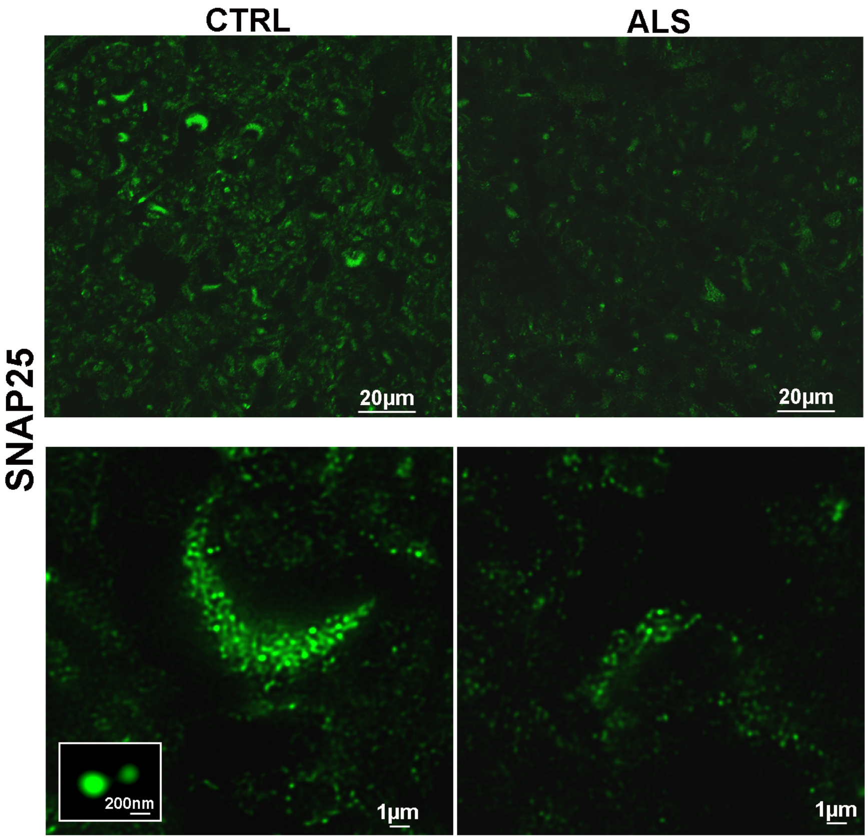
CTRL
ALS
SNAP25
20µm
20µm
200nm
1µm
1µm

Legends for Supplementary Tables

Supplementary Table 1. Cell type specific genes.
List of cell type specific genes for neurons, motorneurons, astrocytes, oligodendrocytes and microglia collected from the literature [1,2]. The majority of cell type specific genes were obtained from Cahoy et al. [1] taking into account only genes showing >10-fold enrichment in this study. Since Cahoy et al. performed their investigations in mouse, we selected only genes with corresponding human orthologues and showing FPKM values >0. Motor neuron specific genes, instead, were collected from Phatnani et al. [2].

Supplementary Table 2. List of differentially expressed genes.
List of differentially expressed genes that are in common between CuffDiff2 and DESeq2. A description for each table column is enclosed in the supplementary file. The specific cell type as inferred from literature data (see Supplementary Table 1) is also reported.

Supplementary Table 3. List of GO terms for UP regulated genes in ALS.
List of GO terms (Biological Process) for UP regulated genes in ALS detected by Enrich [3] and selected at 0,05 significant level. A description for each table column is enclosed in the supplementary file.

Supplementary Table 4. List of GO terms for DOWN regulated genes in ALS.
List of GO terms (Biological Process) for DOWN regulated genes in ALS detected by Enrich [3] and selected at 0,05 significant level. A description for each table column is enclosed in the supplementary file.

Supplementary Table 5. Neuron Specific Genes Enriched in DOWN regulated genes in ALS.
List of neuron specific genes enriched in DOWN regulated genes in ALS.

Supplementary Table 6. Differential expression of mitochondrial genes transcribed by the mitochondrial genome.
This table reports expression values for genes coded by the mitochondrial genomes as calculated by DESeq software. A description for each table column is enclosed in the supplementary file.

Supplementary Table 7. IPA pathways analysis of differentially expressed genes.
List of significant IPA pathways on differentially expressed genes. Pathways with -log(p-value) < 1,3 have been excluded. The column Ratio indicates the ratio between the number of genes in differentially expressed genes and the real number of genes in the pathway.

Supplementary Table 8. IPA list of diseases and bio-functions for differentially expressed genes.
List of enriched diseases and bio-functions in differentially expressed genes, detected by IPA system. Diseases and bio-functions have been selected at 0,05 significant level. Only diseases and bio-functions with at least 3 genes have been considered.

Supplementary Table 9. IPA pathways analysis of differentially spliced genes.
List of significant IPA pathways on differentially spliced genes. Pathways with -log(p-value) < 1,3 have been excluded. The column Ratio indicates the ratio between the number of genes in differentially expressed genes and the real number of genes in the pathway.

Supplementary Table 10. List of GO terms for differentially spliced genes.
List of GO terms (Biological Process, Cellular Component, Molecular Functions) for differentially spliced genes in ALS detected by Enrich [3] and selected at 0,05 significant level. A description for each table column is enclosed in the supplementary file.

Supplementary Table 11. Pathway analysis of DE miRNAs.
This table reports the list of significant pathways associated to differentially expressed miRNAs. Adjusted P value is also reported for each pathway.

Supplementary Table 12. RNA editing frequencies at GRIA2 Q/R site.
RNA editing frequencies at GRIA2 Q/R site in different brain location from REDIportal database [4].

Supplementary Table 13. RNA editing in miRNAs.
RNA editing sites in mature miRNAs were detected by applying a set of Perl scripts kindly provided by Prof. E. Eisenberg (Tel Aviv University), according to the method described in Alon et al. [5]. RNA editing frequencies were calculated using REDItools [6]. In table, we report mean and median values of RNA editing levels detected in miRNAs as well as Mann-Whitney P-values and Adjusted P values (by BH correction method).

References

1 Cahoy, J. D. *et al.* A transcriptome database for astrocytes, neurons, and oligodendrocytes: a new resource for understanding brain development and function. *The Journal of neuroscience : the official journal of the Society for Neuroscience* **28**, 264-278, doi:10.1523/JNEUROSCI.4178-07.2008 (2008).

2 Phatnani, H. P. *et al.* Intricate interplay between astrocytes and motor neurons in ALS. *Proceedings of the National Academy of Sciences of the United States of America* **110**, E756-765, doi:10.1073/pnas.1222361110 (2013).

3 Kuleshov, M. V. *et al.* Enrichr: a comprehensive gene set enrichment analysis web server 2016 update. *Nucleic acids research* **44**, W90-97, doi:10.1093/nar/gkw377 (2016).

4 Picardi, E., D'Erchia, A. M., Lo Giudice, C. & Pesole, G. REDIportal: a comprehensive database of A-to-I RNA editing events in humans. *Nucleic acids research*, gkw767, doi:10.1093/nar/gkw767 (2016).

5 Alon, S. *et al.* Systematic identification of edited microRNAs in the human brain. *Genome research* **22**, 1533-1540, doi:10.1101/gr.131573.111 (2012).

6 Picardi, E. & Pesole, G. REDItools: high-throughput RNA editing detection made easy. *Bioinformatics* **29**, 1813-1814, doi:10.1093/bioinformatics/btt287 (2013).

Supplementary Table 1

Gene Symbol	Cell Type
S100B	Astrocyte
AQP4	Astrocyte
PLA2G7	Astrocyte
SLC39A12	Astrocyte
MLC1	Astrocyte
DIO2	Astrocyte
SLC14A1	Astrocyte
ALDH1L1	Astrocyte
CYP4F12	Astrocyte
ALDOC	Astrocyte
TTPA	Astrocyte
ACSBG1	Astrocyte
CHRDL1	Astrocyte
SLC4A4	Astrocyte
SLC1A2	Astrocyte
SLC25A18	Astrocyte
SLC1A3	Astrocyte
F3	Astrocyte
PPP1R3G	Astrocyte
CYP4F12	Astrocyte
FZD2	Astrocyte
MERTK	Astrocyte
GJB6	Astrocyte
HAPLN1	Astrocyte
RFX4	Astrocyte
PAPSS2	Astrocyte
SLC15A2	Astrocyte
PPP1R3C	Astrocyte
TLR3	Astrocyte
ACOT11	Astrocyte
ATP1A2	Astrocyte
BMPR1B	Astrocyte
GLI3	Astrocyte
TMEM47	Astrocyte
SLC9A3R1	Astrocyte
CTH	Astrocyte
NTSR2	Astrocyte
SLC7A10	Astrocyte
VCAM1	Astrocyte
FGFR3	Astrocyte
CCDC80	Astrocyte
ENTPD2	Astrocyte

CYBRD1	Astrocyte
KIAA1161	Astrocyte
TNC	Astrocyte
TLCD1	Astrocyte
PBXIP1	Astrocyte
GRIN2C	Astrocyte
ADHFE1	Astrocyte
AGT	Astrocyte
GLDC	Astrocyte
SLC7A2	Astrocyte
GJA1	Astrocyte
PDK4	Astrocyte
EGFR	Astrocyte
SOX9	Astrocyte
CLDN10	Astrocyte
PLCD4	Astrocyte
PTPRC	Microglia
CX3CR1	Microglia
ITGAX	Microglia
ITGAM	Microglia
CD68	Microglia
CHAT	Motor Neuron
ISL2	Motor Neuron
ISL1	Motor Neuron
MNX1	Motor Neuron
FOXP1	Motor Neuron
GLRA2	Neuron
NOV	Neuron
PRDM8	Neuron
SLA	Neuron
GABRG2	Neuron
HTR2C	Neuron
HS3ST2	Neuron
MAL2	Neuron
STMN2	Neuron
GABRA5	Neuron
NTS	Neuron
GABRA1	Neuron
SATB2	Neuron
GPR88	Neuron
SYT1	Neuron
GDA	Neuron
MYT1L	Neuron

SLC17A6	Neuron
CALB1	Neuron
SLC12A5	Neuron
EPHA7	Neuron
VIP	Neuron
MEF2C	Neuron
SSTR2	Neuron
PCSK2	Neuron
SNAP25	Neuron
SCG2	Neuron
PGM2L1	Neuron
PLCXD3	Neuron
VSNL1	Neuron
SYT4	Neuron
NRG3	Neuron
KCNF1	Neuron
CCK	Neuron
VGF	Neuron
TMEM130	Neuron
CAMK4	Neuron
SLC6A7	Neuron
ICA1L	Neuron
MYO5B	Neuron
NELL1	Neuron
NEFM	Neuron
NEFL	Neuron
CDH8	Neuron
SV2B	Neuron
GAP43	Neuron
TRHDE	Neuron
CAMK2B	Neuron
RGS4	Neuron
LPL	Neuron
CACNA1B	Neuron
KCNC2	Neuron
TTC9	Neuron
L1CAM	Neuron
CLSTN2	Neuron
NAPB	Neuron
CYB561	Neuron
HPCA	Neuron
CNKSR2	Neuron
UNC13A	Neuron

CBLN1	Neuron
RHOV	Neuron
HS6ST2	Neuron
SOSTDC1	Neuron
STX1A	Neuron
CD24	Neuron
KCNQ2	Neuron
BCL11A	Neuron
ELAVL2	Neuron
NPTX1	Neuron
ENO2	Neuron
TUBB3	Neuron
MOBP	Oligodendrocyte
UGT8	Oligodendrocyte
ENPP6	Oligodendrocyte
CLDN11	Oligodendrocyte
MAG	Oligodendrocyte
PLEKHH1	Oligodendrocyte
FA2H	Oligodendrocyte
SGK2	Oligodendrocyte
IL23A	Oligodendrocyte
MBP	Oligodendrocyte
TF	Oligodendrocyte
GPR62	Oligodendrocyte
TSPAN2	Oligodendrocyte
MAL	Oligodendrocyte
ERBB3	Oligodendrocyte
ADAMTS4	Oligodendrocyte
ELOVL7	Oligodendrocyte
PPP1R14A	Oligodendrocyte
PLP1	Oligodendrocyte
PLXNB3	Oligodendrocyte
GAL3ST1	Oligodendrocyte
BCAS1	Oligodendrocyte
SRPK3	Oligodendrocyte
CPM	Oligodendrocyte
PLA2G4A	Oligodendrocyte
PRKCQ	Oligodendrocyte
SEMA3D	Oligodendrocyte
GALNT6	Oligodendrocyte
PDLIM2	Oligodendrocyte
RFFL	Oligodendrocyte
GPR17	Oligodendrocyte

HAPLN2	Oligodendrocyte
GJB1	Oligodendrocyte
SFT2D1	Oligodendrocyte
LGI3	Oligodendrocyte

Legend:

**Gene Symbol**	Gene Symbol from RefSeq annotations
**Locus (hg19)**	Genomic locus according to hg19 human genome assembly
**value_Control**	Mean expression level (in FPKM) per Control group calculated by CuffDiff2
**value_ALS**	Mean expression level (in FPKM) per ALS group calculated by CuffDiff2
**log2(fold_change)**	Log2 of the ratio value_Control/value_ALS calculated by CuffDiff2
**test_stat**	Statistical Test value calculated by CuffDiff2
**p_value**	P value calculated by CuffDiff2
**q_value**	Corrected P value calculated by CuffDiff2
**baseMean**	Mean counts calculated by DESeq2
**log2(fold_change)**	Log2 of the fold change calculated by DESeq2
**stat**	Statistical Test value calculated by DESeq2
**pvalue**	P value calculated by DESeq2
**padj**	Corrected P value calculated by DESeq2
**Cell Type**	Cell type specific gene (collected from literature)

				CuffDiff2						DESeq2			
Gene Symbo	Locus (hg19)	value_Contr	value_ALS	log2(fold_ch	test_stat	p_value	q_value	baseMean	log2(fold_change	stat	pvalue	padj	Cell Type
A4GALT	chr22:43088126-43116876	4,16147	9,59689	1,20547	1,83901	5,00E-05	0,00070533	316,796787	1,100099221	-3,1759853	0,00149329	0,01167637	-
ABCA1	chr9:107543283-107690527	14,2399	39,1365	1,45858	2,93249	5,00E-05	0,00070533	8596,66397	1,31623242	-4,4607588	8,17E-06	0,00020329	-
ABCC2	chr10:101542354-101611949	0,140736	0,645504	2,19743	1,96057	5,00E-05	0,00070533	97,4607703	1,667890438	-3,6712248	0,00024139	0,00285777	-
ABCC3	chr17:48712217-48769063	2,87229	8,29127	1,52939	2,56993	5,00E-05	0,00070533	862,872578	1,490311394	-6,1874691	6,11E-10	8,95E-08	-
ABCD2	chr12:39945021-40013843	5,80592	2,0592	-1,49544	-2,54359	5,00E-05	0,00070533	546,138983	-1,372323522	4,71136745	2,46E-06	7,96E-05	-
ABCG4	chr11:119019749-119033374	1,86046	0,546202	-1,76816	-1,9114	0,0001	0,00129637	101,073006	-1,368589196	3,79769776	0,00014605	0,00192177	-
ABI3	chr17:47283595-47300587	2,24237	7,06695	1,65606	2,22917	5,00E-05	0,00070533	273,807144	1,418675509	-5,0926224	3,53E-07	1,64E-05	-
ABI3BP	chr3:100468178-100712334	4,3517	12,8449	1,56155	2,97874	5,00E-05	0,00070533	1496,26081	1,331524109	-4,1637477	3,13E-05	0,00058913	-
ABLIM2	chr4:7967036-8160559	9,43422	4,29007	-1,1369	-1,98988	5,00E-05	0,00070533	603,467045	-1,035674058	3,76470549	0,00016675	0,00212002	-
ACADL	chr2:211052715-211090215	2,43771	6,15094	1,33528	1,99299	5,00E-05	0,00070533	313,821387	1,242168743	-4,1690481	3,06E-05	0,00057747	-
ACAT2	chr6:160181290-160210735	35,1825	14,9938	-1,23049	-1,24838	0,00525	0,0358996	1042,69701	-1,181896756	6,17041475	6,81E-10	9,83E-08	-
ACHE	chr7:100487614-100493592	30,8304	7,88532	-1,96711	-3,46889	5,00E-05	0,00070533	750,034167	-1,531101999	4,04991598	5,12E-05	0,00087646	-
ACKR1	chr1:159173802-159176290	14,1094	42,6614	1,59627	2,61926	5,00E-05	0,00070533	741,499491	1,201442776	-3,3399168	0,00083804	0,00745703	-
ACLY	chr17:40023178-40075272	88,4483	28,9974	-1,60891	-2,93109	5,00E-05	0,00070533	7010,52544	-1,352059252	4,02950099	5,59E-05	0,00093849	-
ACOT7	chr1:6324331-6453826	59,0745	14,0998	-2,06686	-3,8571	5,00E-05	0,00070533	1311,5549	-1,68820386	5,06347499	4,12E-07	1,88E-05	-
ACP5	chr19:11685474-11689801	2,73026	8,39034	1,61969	2,03127	5,00E-05	0,00070533	205,356593	1,349822989	-3,4545758	0,00055116	0,00550226	-
ACSL5	chr10:114133915-114188138	4,05946	12,4013	1,61114	2,90965	5,00E-05	0,00070533	890,00411	1,440098998	-4,563873	5,02E-06	0,00013928	-
ACSL6	chr5:131285666-131347761	30,2014	8,60402	-1,81154	-3,37283	5,00E-05	0,00070533	3150,76576	-1,538583187	4,40540931	1,06E-05	0,00024617	-
ACTG2	chr2:74120092-74146780	3,64141	19,3238	2,40781	3,23188	5,00E-05	0,00070533	404,820526	1,320676577	-2,6434509	0,00820657	0,04152587	-
ACTL6B	chr7:100240725-100254084	2,67323	0,529369	-2,33624	-2,02012	0,00035	0,00384337	69,1214647	-1,42450974	3,31549791	0,0009148	0,00793147	-
ACTN1	chr14:69340839-69446083	11,3164	30,7626	1,44277	2,95629	5,00E-05	0,00070533	2491,75979	1,323004665	-5,7248171	1,04E-08	9,50E-07	-
ADAM19	chr5:156904311-157002831	4,8554	1,92162	-1,33727	-2,37853	5,00E-05	0,00070533	614,303229	-1,215710221	5,26327249	1,42E-07	7,68E-06	-
ADAM23	chr2:207308367-207485854	21,227	4,44208	-2,25659	-3,88963	5,00E-05	0,00070533	2013,06345	-1,769055527	4,47938254	7,49E-06	0,00018954	-
ADAMTS3	chr4:73146685-73434516	0,288088	0,651139	1,17646	1,26516	0,00495	0,0342524	90,2494709	1,219568805	-4,3540802	1,34E-05	0,00029397	-
ADAMTS9	chr3:64501330-64997143	6,54395	25,0854	1,93861	3,1285	5,00E-05	0,00070533	4182,81307	1,462618636	-3,433172	0,00059656	0,00582037	-
ADAMTSL1	chr9:18474078-18910947	2,46529	1,04893	-1,23284	-1,72716	5,00E-05	0,00070533	347,21689	-1,08246524	3,25552563	0,00113183	0,00939849	-
ADAMTSL2	chr9:136397285-136440641	2,50474	0,977791	-1,35706	-1,79574	5,00E-05	0,00070533	188,970749	-1,025371941	3,83321719	0,00012648	0,00172725	-
ADD2	chr2:70889215-70995375	18,6514	6,99631	-1,41462	-2,7608	5,00E-05	0,00070533	1577,56939	-1,239420785	3,90212792	9,54E-05	0,0014024	-
ADIRF	chr10:88728187-88769960	59,9839	141,904	1,24227	2,44067	5,00E-05	0,00070533	1656,11683	1,191958129	-4,6721618	2,98E-06	9,30E-05	-
ADM	chr11:10326641-10328923	18,7856	57,4406	1,61244	3,06484	5,00E-05	0,00070533	1345,86597	1,443147544	-4,6576158	3,20E-06	9,76E-05	-
ADORA1	chr1:203096835-203136533	27,5107	13,3986	-1,03792	-2,11631	5,00E-05	0,00070533	1193,75849	-1,017450174	5,8271872	5,64E-09	5,73E-07	-
ADORA3	chr1:112025969-112106597	5,85176	24,4136	2,06075	3,46554	5,00E-05	0,00070533	779,392333	1,816230204	-6,0572305	1,38E-09	1,81E-07	-
ADRA1D	chr20:4201277-4229659	2,78751	0,39272	-2,82741	-2,4001	5,00E-05	0,00070533	69,0222072	-1,858239991	4,07151106	4,67E-05	0,00081105	-
AFAP1L1	chr5:148651400-148721367	2,24207	5,59149	1,3184	2,15965	5,00E-05	0,00070533	529,921405	1,126012671	-3,1195541	0,00181125	0,01351234	-
AHNAK2	chr14:105403590-105444694	43,7881	8,79849	-2,31521	-3,42996	5,00E-05	0,00070533	13466,9785	-1,737758257	4,13622013	3,53E-05	0,00065519	-
AIM1	chr6:106959729-107018334	0,775678	1,63341	1,07435	1,63902	0,0003	0,00336918	286,883287	1,031061369	-5,0795628	3,78E-07	1,74E-05	-
ALOX15B	chr17:7942357-7952451	0,0630054	3,54369	5,81363	3,69119	5,00E-05	0,00070533	149,39522	3,817162548	-8,5421649	1,32E-17	1,24E-14	-
ALOX5	chr10:45869623-45941567	4,38194	11,142	1,34636	2,29699	5,00E-05	0,00070533	604,912358	1,218753908	-4,7572395	1,96E-06	6,67E-05	-
ALOX5AP	chr13:31287614-31338565	7,09383	27,9966	1,98062	2,93991	5,00E-05	0,00070533	372,922796	1,79603411	-5,9222525	3,18E-09	3,72E-07	-
ALPK1	chr4:113218498-113363764	1,95176	4,8172	1,30342	2,3681	5,00E-05	0,00070533	627,668454	1,310387191	-8,5525969	1,20E-17	1,18E-14	-
ALPL	chr1:21835857-21904905	3,6957	9,03249	1,28784	2,18344	5,00E-05	0,00070533	462,005752	1,219160592	-4,6712752	2,99E-06	9,33E-05	-
AMER3	chr2:131513076-131525707	2,08699	0,413811	-2,33438	-2,73981	5,00E-05	0,00070533	182,5156	-1,676412255	3,81100298	0,0001384	0,00184494	-
AMICA1	chr11:118064441-118095809	0,81695	2,67337	1,71034	1,89776	5,00E-05	0,00070533	130,294893	1,616786615	-5,4256642	5,77E-08	3,67E-06	-
AMPH	chr7:38423296-38671167	8,13172	2,62884	-1,62913	-2,65324	5,00E-05	0,00070533	547,782893	-1,314851174	3,81932475	0,00013382	0,00180361	-
ANGPTL4	chr19:8429010-8439259	11,3162	34,0488	1,58921	3,09888	5,00E-05	0,00070533	1135,26654	1,517867964	-7,6496286	2,02E-14	9,24E-12	-
ANK1	chr8:41510743-41754280	5,62801	1,67148	-1,7515	-2,38734	5,00E-05	0,00070533	741,224179	-1,669173864	5,04465363	4,54E-07	2,03E-05	-
ANKRD22	chr10:90562486-90611732	0,0629819	0,512304	3,02399	1,92626	0,001	0,00924656	34,3331931	2,032899897	-4,5288695	5,93E-06	0,00016	-
ANKRD29	chr18:21179977-21242849	8,35528	3,18506	-1,39137	-2,06602	5,00E-05	0,00070533	365,081242	-1,17362454	3,40862826	0,0006529	0,00620649	-
ANKRD34B	chr5:79852573-79866304	0,997329	0,160357	-2,63678	-1,63266	0,00475	0,0331278	60,9121117	-1,434896557	2,88328744	0,00393548	0,02421248	-
ANKRD36BP2	chr2:89065418-89106109	2,4202	1,07701	-1,16809	-1,37401	0,00235	0,018855	149,256699	-1,109647735	4,10803153	3,99E-05	0,00072231	-
ANO4	chr12:101188373-101522419	7,88266	2,63494	-1,58091	-2,68767	5,00E-05	0,00070533	711,157618	-1,291535944	4,77184427	1,83E-06	6,30E-05	-
ANPEP	chr15:90328125-90358072	0,616532	1,98646	1,68795	1,86755	0,0002	0,0023747	157,508803	1,417821112	-3,3734908	0,00074222	0,00679788	-
AOX1	chr2:201450730-201536217	1,31873	5,48912	2,05743	3,09842	5,00E-05	0,00070533	546,636369	1,610287073	-3,9349768	8,32E-05	0,00126617	-
AP3B2	chr15:83211950-83378660	11,3138	3,0933	-1,87087	-2,06993	5,00E-05	0,00070533	814,412633	-1,488391597	4,5012782	6,75E-06	0,00017636	-
APOB	chr2:21224300-21266945	0,211437	0,586618	1,47219	1,95815	5,00E-05	0,00070533	190,455255	1,35821449	-4,2096966	2,56E-05	0,00049733	-
APOBR	chr16:28505969-28510291	0,647099	2,10854	1,70418	2,12838	5,00E-05	0,00070533	158,777925	1,616002057	-6,7976526	1,06E-11	2,68E-09	-
APOC1	chr19:45417920-45422606	18,0991	164,752	3,1863	5,0458	5,00E-05	0,00070533	1032,78874	2,600671753	-6,8297686	8,51E-12	2,17E-09	-
APOE	chr19:45409038-45412650	489,343	1138,18	1,21781	2,3071	5,00E-05	0,00070533	18775,7811	1,123515096	-3,7262419	0,00019436	0,00238624	-
APOL1	chr22:36649116-36663577	3,19721	7,95181	1,31447	2,17735	5,00E-05	0,00070533	479,854251	1,167845845	-3,9830897	6,80E-05	0,00108904	-
APOL4	chr22:36585175-36600879	0,941862	2,68154	1,50948	1,9349	5,00E-05	0,00070533	178,191865	1,330644115	-4,1492277	3,34E-05	0,00062353	-
APOLD1	chr12:12878850-12944399	23,5404	74,6741	1,66547	3,03929	5,00E-05	0,00070533	5164,11325	1,276912747	-3,1668243	0,00154113	0,01195089	-
AQP3	chr9:33441151-33447631	0,424244	1,86124	2,1333	1,71328	0,0008	0,00771573	46,1838426	1,638767613	-3,8391209	0,00012348	0,00169861	-
AQP6	chr12:50366619-50370922	3,60898	0,690557	-2,38576	-2,78591	5,00E-05	0,00070533	131,710062	-2,379572444	11,2678125	1,89E-29	7,81E-26	-
AQP9	chr15:58430407-58478110	0,144467	1,10517	2,93545	2,36735	5,00E-05	0,00070533	57,3344666	2,34172413	-5,6738265	1,40E-08	1,19E-06	-
ARHGAP30	chr1:161016731-161039760	1,97709	5,31888	1,42775	2,18171	5,00E-05	0,00070533	453,901331	1,295936933	-4,8499682	1,23E-06	4,54E-05	-
ARHGAP36	chrX:130192215-130223859	22,2865	5,19214	-2,10177	-3,23114	5,00E-05	0,00070533	960,969366	-1,3091681	2,67924086	0,00737893	0,03834405	-
ARHGAP44	chr17:12569206-12921381	7,64152	2,29523	-1,73522	-1,35528	0,00755	0,0475797	521,608392	-1,401740911	4,10897682	3,97E-05	0,00071999	-
ARHGDIB	chr12:15094949-15114562	16,1248	36,9688	1,19702	2,26922	5,00E-05	0,00070533	934,345475	1,082418623	-3,9433661	8,03E-05	0,00122937	-
ARL11	chr13:50202434-50208008	0,251668	0,727739	1,5319	1,45251	0,0018	0,015174	55,211316	1,512871536	-6,698615	2,10E-11	5,05E-09	-
ASB5	chr4:177134825-177190373	0,549541	1,6042	1,54555	1,64889	0,0003	0,00336918	92,5135108	1,330978093	-3,4290104	0,00060579	0,0058889	-
ASPHD1	chr16:29912146-29917377	23,9156	10,9019	-1,13337	-2,00304	5,00E-05	0,00070533	495,163266	-1,011468337	5,70530254	1,16E-08	1,03E-06	-
ASPHD2	chr22:26825279-26840978	3,59042	0,862209	-2,05805	-2,5392	5,00E-05	0,00070533	152,111391	-1,700216154	4,51907218	6,21E-06	0,00016516	-
ASS1	chr9:133320093-133376661	9,0271	30,3985	1,75166	3,15835	5,00E-05	0,00070533	1061,33541	1,555141666	-4,4961139	6,92E-06	0,00017918	-
ATOH8	chr2:85980908-86018506	2,0122	5,62372	1,48275	2,52222	5,00E-05	0,00070533	553,755269	1,320765723	-4,3826241	1,17E-05	0,00026795	-
ATP13A2	chr1:17312452-17338467	46,7125	20,0931	-1,21711	-2,53646	5,00E-05	0,00070533	2971,58145	-1,042835976	3,6500516	0,00026219	0,00306182	-
ATP1A3	chr19:42470733-42498428	91,0337	14,7737	-2,62337	-4,4904	5,00E-05	0,00070533	4699,18941	-1,665378926	3,44236858	0,00057664	0,00567162	-
ATP1B1	chr1:169075946-169396670	366,486	144,277	-1,34492	-2,49153	5,00E-05	0,00070533	12155,9468	-1,244307012	4,4337595	9,26E-06	0,00022507	-
ATP2B2	chr3:10365706-10547268	15,2585	4,36538	-1,80544	-3,56563	5,00E-05	0,00070533	2299,52951	-1,628846676	5,00329674	5,64E-07	2,43E-05	-
ATP2B3	chrX:152801579-152848387	5,18286	0,94717	-2,45205	-3,70455	5,00E-05	0,00070533	520,473103	-1,795091244	4,19301366	2,75E-05	0,00053087	-
ATP8A2	chr13:25946208-26595420	17,4673	2,54978	-2,77621	-4,49866	5,00E-05	0,00070533	1333,58727	-2,048228147	4,81512487	1,47E-06	5,26E-05	-
ATP8B1	chr18:55297533-55470327	1,04949	3,2016	1,60911	2,24604	5,00E-05	0,00070533	402,405753	1,321553676	-3,7678547	0,00016466	0,0021034	-
B4GALNT1	chr12:58012187-58027022	8,83671	3,1009	-1,51082	-2,16234	5,00E-05	0,00070533	320,024383	-1,236660107	3,37691615	0,00073303	0,00674083	-
B4GALT1	chr9:33110638-33179981	4,46714	13,6444	1,61089	2,93183	5,00E-05	0,00070533	810,732771	1,434155988	-5,679355	1,35E-08	1,16E-06	-
B4GALT6	chr18:29202208-29264686	9,14397	3,3475	-1,44973	-2,39937	5,00E-05	0,00070533	622,485511	-1,231353499	3,3767321	0,00073353	0,00674083	-
BAI1	chr8:143545376-143626368	11,5075	3,97983	-1,5318	-2,96889	5,00E-05	0,00070533	935,668452	-1,48639539	12,3997442	2,62E-35	2,70E-31	-
BASP1	chr5:17130136-17276954	41,5875	16,3061	-1,35074	-2,60352	5,00E-05	0,00070533	1014,4172	-1,104678624	3,59405945	0,00032557	0,00364319	-
BATF	chr14:75988783-76013334	0,126105	0,800197	2,66573	1,3892	0,00445	0,0313873	13,3653303	2,006351882	-4,9492887	7,45E-07	2,97E-05	-
BATF3	chr1:212859758-212873327	0,566642	1,7914	1,66058	1,32362	0,0061	0,0403171	30,8978047	1,21754638	-4,4356629	9,18E-06	0,00022381	-
BCL2A1	chr15:80253231-80263643	1,08078	5,83306	2,43218	2,42958	5,00E-05	0,00070533	88,7815241	1,984963009	-5,7587067	8,48E-09	7,95E-07	-
BDKRB2	chr14:96671134-96710666	0,371428	0,971386	1,38696	1,48684	0,00105	0,00963992	83,0926214	1,16371568	-3,7333802	0,00018893	0,00233165	-
BEND6	chr6:56819772-56892142	14,1016	6,05962	-1,21856	-2,17887	5,00E-05	0,00070533	799,008784	-1,131842968	3,51827914	0,00043436	0,00459401	-
BEX1	chrX:102317580-102319168	73,336	28,1694	-1,38039	-2,64114	5,00E-05	0,00070533	941,183061	-1,220540962	3,98669323	6,70E-05	0,00107592	-
BEX5	chrX:101408678-101410986	17,7559	6,30915	-1,49278	-2,04962	5,00E-05	0,00070533	211,132201	-1,255639499	3,43556719	0,00059132	0,00577736	-
BNC2	chr9:16409500-16870786	1,22207	3,42201	1,48552	2,724	5,00E-05	0,00070533	816,846014	1,294006822	-4,3143646	1,60E-05	0,00033911	-
BRCA2	chr13:32889616-32973809	0,205554	0,50898	1,30809	1,59179	0,00065	0,00650426	137,449717	1,208924673	-4,3505548	1,36E-05	0,00029778	-
BRINP2	chr1:177140632-177251558	7,25103	3,15142	-1,20218	-2,10066	5,00E-05	0,00070533	529,775126	-1,142989218	5,35452377	8,58E-08	5,22E-06	-
BRSK1	chr19:55795533-55823903	24,3724	10,5731	-1,20484	-2,43535	5,00E-05	0,00070533	1263,7716	-1,008649999	4,24054206	2,23E-05	0,00044629	-
BSN	chr3:49591921-49708982	6,06516	2,58461	-1,2306	-2,55046	5,00E-05	0,00070533	1699,4426	-1,110651262	4,12627099	3,69E-05	0,00067627	-
BTBD11	chr12:107712196-108053419	6,61647	2,79891	-1,2412	-2,17199	5,00E-05	0,00070533	585,676184	-1,031974554	3,45048354	0,00055958	0,00555144	-
BTK	chrX:100604434-100645784	1,82271	5,25168	1,52669	2,2012	5,00E-05	0,00070533	351,746728	1,373574798	-4,760447	1,93E-06	6,59E-05	-
C10orf10	chr10:45455218-45490172	41,7889	169,794	2,02259	2,76056	5,00E-05	0,00070533	5207,65464	1,693834302	-4,9835187	6,24E-07	2,64E-05	-
C11orf87	chr11:109292845-109299893	2,80594	0,553624	-2,34151	-2,83378	5,00E-05	0,00070533	193,803449	-1,841387373	4,41025664	1,03E-05	0,0002435	-
C12orf76	chr12:110478982-110505500	26,6269	12,1917	-1,12699	-2,20585	5,00E-05	0,00070533	1034,19267	-1,076823865	6,53542215	6,34E-11	1,26E-08	-
C19orf33	chr19:38794199-38806606	0,386251	14,8385	5,26366	1,00926	0,00285	0,0220658	83,4288913	3,520285844	-7,5811811	3,42E-14	1,47E-11	-
C1orf162	chr1:112016603-112021134	2,74899	12,3336	2,16562	2,76052	5,00E-05	0,00070533	275,826237	1,74261132	-14,108409	3,37E-45	6,96E-41	-
C1orf233	chr1:1533387-1535476	10,968	2,4372	-2,17	-2,77522	5,00E-05	0,00070533	205,650288	-1,834439268	5,45033728	5,03E-08	3,27E-06	-
C1QA	chr1:22963117-22966175	24,02	78,3008	1,70479	3,18673	5,00E-05	0,00070533	1254,54785	1,487227006	-4,1021077	4,09E-05	0,00073525	-
C1QB	chr1:22979681-22988029	37,3481	178,119	2,25374	4,51112	5,00E-05	0,00070533	2731,39389	2,011887512	-6,2279767	4,72E-10	7,07E-08	-
C1QC	chr1:22970117-22974603	30,5508	129,949	2,08867	4,15939	5,00E-05	0,00070533	2057,67147	1,886664319	-6,3736765	1,85E-10	3,10E-08	-
C1QTNF4	chr11:47611215-47615961	17,4742	4,73004	-1,8853	-2,5457	5,00E-05	0,00070533	237,185298	-1,636864868	5,6916841	1,26E-08	1,10E-06	-
C1R	chr12:7187514-7245043	24,4391	60,4916	1,30754	2,71788	5,00E-05	0,00070533	2835,13946	1,192408654	-4,0289994	5,60E-05	0,00093973	-
C1RL	chr12:7247145-7274447	3,9462	10,482	1,40937	2,33705	5,00E-05	0,00070533	738,196796	1,319141769	-7,1864473	6,65E-13	2,35E-10	-
C1S	chr12:7167979-7178335	44,0231	98,2672	1,15845	2,39102	5,00E-05	0,00070533	5645,94018	1,033662403	-3,3404254	0,0008365	0,00744981	-
C20orf166-AS	chr20:61141437-61167971	1,30857	0,171823	-2,929	-2,38753	5,00E-05	0,00070533	59,3068436	-2,333566355	5,62141521	1,89E-08	1,51E-06	-
C2orf40	chr2:106682112-106694609	9,41734	27,3513	1,53822	2,22692	5,00E-05	0,00070533	338,044893	1,276274843	-3,2861627	0,00101562	0,00859767	-
C2orf72	chr2:231902280-231914427	31,2036	14,4137	-1,11427	-2,35382	5,00E-05	0,00070533	1644,27976	-1,087435146	5,8669981	4,44E-09	4,77E-07	-
C3	chr19:6677845-6720662	44,457	137,212	1,62592	3,11785	5,00E-05	0,00070533	16074,9953	1,477479073	-5,0184101	5,21E-07	2,28E-05	-
C3AR1	chr12:8210918-8218955	7,42245	20,2419	1,44738	2,54124	5,00E-05	0,00070533	633,197591	1,275611918	-4,0716285	4,67E-05	0,00081105	-
C3orf52	chr3:111805181-111837073	0,161096	0,782726	2,28058	1,32229	0,00545	0,0369961	30,3478342	1,456069465	-3,1924627	0,00141065	0,01117263	-

C5AR1	chr19:47813103-47825327	2,29207	6,61965	1,5301	2,08727	5,00E-05	0,00070533	255,872501	1,268451273	-3,8985227	9,68E-05	0,00141939	-
C5AR2	chr19:47835403-47845272	0,845381	3,87134	2,19516	1,82019	0,00015	0,00184356	70,8982396	1,708688309	-4,00325	6,25E-05	0,00102565	-
CA4	chr17:58227301-58236906	3,51592	1,20519	-1,54465	-1,539	0,001	0,00924656	80,078655	-1,070017335	2,70670103	0,00679554	0,03606654	-
CABLES1	chr18:20714527-20840434	7,16304	2,99116	-1,25987	-2,30858	5,00E-05	0,00070533	602,285293	-1,159375068	5,83237341	5,46E-09	5,58E-07	-
CABP1	chr12:121078421-121105127	8,52079	1,43129	-2,57367	-2,72011	5,00E-05	0,00070533	142,655085	-1,809302062	4,45859192	8,25E-06	0,00020486	-
CABP7	chr22:30116343-30162969	38,3085	6,95463	-2,46162	-3,79717	5,00E-05	0,00070533	1437,98838	-1,980566244	4,97317833	6,59E-07	2,76E-05	-
CABYR	chr18:21718919-21741564	5,90413	1,6445	-1,84407	-2,40288	5,00E-05	0,00070533	236,431307	-1,655598005	4,65443809	3,25E-06	9,87E-05	-
CACNA1B	chr9:140772240-141019076	4,75542	2,00314	-1,24731	-2,30913	5,00E-05	0,00070533	908,925876	-1,069511798	3,82794325	0,00012922	0,00175192	Neuron
CACNA1G	chr17:48633567-48704832	1,89271	0,535285	-1,82207	-2,31384	5,00E-05	0,00070533	230,788984	-1,328522166	3,87616046	0,00010612	0,00151592	-
CACNA2D3	chr3:54156692-55108584	9,11968	2,88767	-1,65908	-2,68969	5,00E-05	0,00070533	664,430713	-1,180583167	3,25361946	0,00113945	0,00945797	-
CACNG2	chr22:36956915-37098690	3,74596	0,568898	-2,71909	-3,49253	5,00E-05	0,00070533	257,594846	-2,146237236	5,17926354	2,23E-07	1,11E-05	-
CACNG3	chr16:24266873-24373737	0,704116	0,202403	-1,79858	-1,45177	0,00195	0,0162027	27,7389596	-1,25466156	3,12945728	0,0017513	0,01316023	-
CACNG8	chr19:54466289-54493469	1,09129	0,461877	-1,24045	-1,73991	5,00E-05	0,00070533	179,638255	-1,03970324	3,4125698	0,00064353	0,00615354	-
CADPS	chr3:62384020-62861064	24,5293	8,59192	-1,51345	-2,82705	5,00E-05	0,00070533	2481,45025	-1,188174796	3,15420713	0,00160935	0,01233614	-
CALB2	chr16:71392615-71424342	11,4876	3,72155	-1,6261	-2,28552	5,00E-05	0,00070533	327,686055	-1,21421419	2,76336562	0,00572086	0,03181084	-
CALM3	chr19:47104511-47114039	191,122	86,7948	-1,13882	-2,36381	5,00E-05	0,00070533	6512,25285	-1,022400587	3,71277204	0,000205	0,00249423	-
CALML4	chr15:68483042-68498448	0,660803	1,71547	1,37631	1,80922	5,00E-05	0,00070533	166,759478	1,206724823	-5,283946	1,26E-07	7,07E-06	-
CALY	chr10:135138927-135150475	29,6118	10,8846	-1,44389	-2,28448	5,00E-05	0,00070533	400,081173	-1,086309388	2,6527348	0,00798426	0,04073057	-
CAMK2N1	chr1:20808883-20812728	55,255	26,7735	-1,0453	-2,24231	5,00E-05	0,00070533	1650,24665	-1,011808807	7,01639306	2,28E-12	6,57E-10	-
CAMK2N2	chr3:183967444-184010819	22,6882	5,3106	-2,095	-1,94795	0,00085	0,00810031	270,365206	-1,74233751	4,86537269	1,14E-06	4,28E-05	-
CAMKK1	chr17:3763616-3796337	9,87477	3,57898	-1,4642	-2,47067	5,00E-05	0,00070533	608,89668	-1,078814076	2,90592181	0,00366173	0,02296964	-
CAMSAP3	chr19:7660787-7683196	2,29319	0,770781	-1,57296	-1,9512	5,00E-05	0,00070533	136,559145	-1,330784001	4,27074166	1,95E-05	0,00040002	-
CAP2	chr6:17393735-17558023	10,8473	4,77722	-1,18309	-2,04207	5,00E-05	0,00070533	616,766558	-1,062225829	3,3990211	0,00067628	0,00634892	-
CAPG	chr2:85621870-85641197	14,2241	46,2458	1,70099	3,17327	5,00E-05	0,00070533	1200,36013	1,516140416	-4,7215086	2,34E-06	7,64E-05	-
CASKIN1	chr16:2205798-2246465	12,3322	4,85348	-1,34534	-2,00546	5,00E-05	0,00070533	1032,14736	-1,285636132	9,05893632	1,32E-19	2,09E-16	-
CASP1	chr11:104896236-104905884	3,69091	8,29446	1,16817	1,70314	0,00015	0,00184356	338,603923	1,072950188	-5,1280549	2,93E-07	1,40E-05	-
CASP4	chr11:104813593-104839325	4,23925	11,0369	1,38046	2,11765	5,00E-05	0,00070533	411,290495	1,23041777	-4,5291365	5,92E-06	0,00016	-
CASP5	chr11:104864966-104893895	0,08854	0,37462	2,08103	0,982411	0,00305	0,0232184	18,3179567	1,564970177	-3,5061782	0,00045459	0,00475202	-
CASP7	chr10:115438920-115490668	3,29574	7,61339	1,20794	1,97277	5,00E-05	0,00070533	392,667666	1,154538417	-6,2876485	3,22E-10	5,08E-08	-
CBLN1	chr16:49311828-49315742	1,72798	0,443321	-1,96266	-1,63192	0,00175	0,0148269	49,331694	-1,522868862	3,81745992	0,00013483	0,00181492	Neuron
CCDC129	chr7:31553684-31698334	5,06156	0,442685	-3,51523	-4,4362	5,00E-05	0,00070533	342,621819	-2,847761726	8,13603395	4,08E-16	3,12E-13	-
CCDC184	chr12:48577365-48579709	10,3675	1,6944	-2,61322	-3,53807	5,00E-05	0,00070533	253,285665	-2,157269306	5,87920715	4,12E-09	4,50E-07	-
CCDC3	chr10:12938624-13141773	5,25151	14,4975	1,465	2,58075	5,00E-05	0,00070533	657,588052	1,175302898	-3,6095456	0,00030673	0,003472	-
CCDC64	chr12:120427647-120532299	2,44305	1,03066	-1,24512	-1,6317	0,00035	0,00384337	181,805487	-1,146198681	4,37705526	1,20E-05	0,00027338	-
CCDC68	chr18:52568739-52626739	1,10742	2,83387	1,35557	1,91658	5,00E-05	0,00070533	259,379156	1,237755551	-4,7075573	2,51E-06	8,07E-05	-
CCDC92	chr12:124420954-124457163	37,697	16,1687	-1,22124	-2,49721	5,00E-05	0,00070533	1358,05599	-1,114768249	4,92454585	8,46E-07	3,30E-05	-
CCR1	chr3:46243199-46249832	2,61481	8,70785	1,73561	2,72665	5,00E-05	0,00070533	384,969939	1,603263453	-5,9171973	3,27E-09	3,82E-07	-
CCR5	chr3:46411632-46417697	0,787716	1,98966	1,33677	1,61468	0,0004	0,00431348	128,133778	1,189176931	-3,9268625	8,61E-05	0,00129531	-
CD14	chr5:140011312-140013286	17,1489	54,6547	1,67223	3,26235	5,00E-05	0,00070533	1354,69676	1,531572333	-5,8155114	6,04E-09	5,97E-07	-
CD163	chr12:7623411-7656414	2,97825	41,492	3,8003	6,68664	5,00E-05	0,00070533	2966,42938	3,276695745	-9,5864534	9,12E-22	2,09E-18	-
CD180	chr5:66478103-66492617	0,633639	3,01435	2,25012	2,61698	5,00E-05	0,00070533	150,033887	2,05284357	-7,806425	5,88E-15	3,19E-12	-
CD1D	chr1:158149736-158156216	0,0898832	0,604671	2,75003	2,13569	5,00E-05	0,00070533	41,5968415	2,482058529	-7,0546825	1,73E-12	5,25E-10	-
CD300A	chr17:72462521-72480937	1,79151	7,91706	2,14379	2,91985	5,00E-05	0,00070533	283,458066	1,884183648	-5,9025651	3,58E-09	4,08E-07	-
CD33	chr19:51728334-51743274	1,52702	3,36566	1,14017	1,31017	0,0045	0,0316337	105,348662	1,006514317	-3,4276147	0,00060891	0,00590731	-
CD37	chr19:49838676-49865714	6,8349	19,1712	1,48795	1,68699	0,00035	0,00384337	359,450802	1,325918635	-4,0614368	4,88E-05	0,00084007	-
CD48	chr1:160648535-160681641	1,34153	5,11169	1,92993	2,0981	0,0001	0,00129637	112,909962	1,646755096	-4,631709	3,63E-06	0,00010814	-
CD53	chr1:111413820-111442558	10,3129	28,4569	1,46433	2,58826	5,00E-05	0,00070533	873,87391	1,273330123	-4,2876973	1,81E-05	0,00037554	-
CD68	chr17:7482804-7485429	8,67752	31,2012	1,84624	3,41694	5,00E-05	0,00070533	1052,08867	1,597182919	-4,8267316	1,39E-06	5,03E-05	Microglia
CD74	chr5:149781199-149792499	192,429	463,45	1,26809	2,42811	5,00E-05	0,00070533	14852,4493	1,117208317	-3,6660607	0,00024632	0,0029094	-
CD84	chr1:160510883-160549306	2,21693	7,38274	1,73559	3,20978	5,00E-05	0,00070533	1143,71326	1,445969601	-4,1366867	3,52E-05	0,00065495	-
CD86	chr3:121774208-121839988	2,68602	7,89012	1,55458	2,53585	5,00E-05	0,00070533	468,682055	1,280798242	-3,7757795	0,00015951	0,00205973	-
CD93	chr20:23059992-23066977	5,95963	22,3778	1,90878	3,64527	5,00E-05	0,00070533	2393,56653	1,578325092	-4,440473	8,98E-06	0,0002192	-
CDC42EP3	chr2:37869024-37899678	2,78817	6,24211	1,16272	2,06379	5,00E-05	0,00070533	629,161874	1,002206436	-3,4665192	0,00052724	0,0053181	-
CDCP1	chr3:45123765-45187914	0,391872	1,76127	2,16816	2,0763	5,00E-05	0,00070533	160,719813	2,015027324	-8,9639013	3,13E-19	4,62E-16	-
CDH12	chr5:21750972-22853731	4,4152	0,712998	-2,63051	-3,32866	5,00E-05	0,00070533	314,350503	-1,923035083	4,59699901	4,29E-06	0,00012284	-
CDH18	chr5:19473154-20575982	6,86059	1,72709	-1,98999	-2,96449	5,00E-05	0,00070533	416,952355	-1,600062512	3,96413195	7,37E-05	0,00115769	-
CDH5	chr16:66400524-66438689	8,02056	18,4342	1,20061	2,2905	5,00E-05	0,00070533	1607,70857	1,005777554	-2,8627371	0,00419999	0,02541548	-
CDH8	chr16:61685914-62070739	5,17551	2,44441	-1,08221	-1,87739	5,00E-05	0,00070533	458,319998	-1,016915498	5,3005487	1,15E-07	6,55E-06	Neuron
CDHR1	chr10:85954390-85979376	2,39482	0,871253	-1,45875	-2,12773	5,00E-05	0,00070533	291,488602	-1,255320147	4,37561961	1,21E-05	0,00027398	-
CDK2	chr12:56360552-56366573	2,9052	6,99816	1,26834	1,84792	5,00E-05	0,00070533	261,225492	1,115194709	-4,0430733	5,28E-05	0,00089745	-
CDK5R1	chr17:30814104-30818271	10,8944	3,68469	-1,56397	-2,73023	5,00E-05	0,00070533	582,880243	-1,37868319	4,36134584	1,29E-05	0,00028651	-
CDK5R2	chr2:219824349-219826877	4,06052	1,02515	-1,98583	-2,2001	5,00E-05	0,00070533	91,3734175	-1,525199535	3,83040845	0,00012793	0,00174018	-
CDR2L	chr17:72983726-73001892	21,8813	9,98512	-1,13184	-2,32402	5,00E-05	0,00070533	1207,91542	-1,060057078	5,47467542	4,38E-08	2,93E-06	-
CDS1	chr4:85504056-85572493	11,1289	3,66281	-1,60329	-2,78709	5,00E-05	0,00070533	733,322249	-1,368499862	3,91207586	9,15E-05	0,00135746	-
CEBPB	chr20:48807119-48809227	7,84074	22,2081	1,50203	2,57432	5,00E-05	0,00070533	529,007434	1,426211075	-5,2523586	1,50E-07	8,05E-06	-
CEBPD	chr8:48649475-48650726	45,9398	132,377	1,52684	2,89787	5,00E-05	0,00070533	1988,70799	1,304742013	-3,3566037	0,00078906	0,00712306	-
CELF4	chr18:34823007-35146000	3,58755	1,4402	-1,31673	-1,85567	5,00E-05	0,00070533	251,649395	-1,072095602	2,72102702	0,00650794	0,03488089	-
CEND1	chr11:787109-790126	50,2655	13,1086	-1,93906	-3,60395	5,00E-05	0,00070533	987,633565	-1,619010693	4,56282187	5,05E-06	0,00013979	-
CEP170B	chr14:105331649-105363107	15,2323	5,71031	-1,41549	-2,905	5,00E-05	0,00070533	1510,0361	-1,250812958	4,95748568	7,14E-07	2,89E-05	-
CEP55	chr10:95256368-95288849	0,110398	0,387034	1,80975	1,40055	0,0017	0,0144616	24,8591081	1,586198741	-3,9914104	6,57E-05	0,00106218	-
CFD	chr19:859664-863610	0,842586	7,58392	3,17005	2,68113	5,00E-05	0,00070533	97,889142	2,587354092	-6,616463	3,68E-11	7,85E-09	-
CFI	chr4:110661847-110723335	7,39794	17,8068	1,26723	2,35436	5,00E-05	0,00070533	850,103793	1,205754475	-5,5353047	3,11E-08	2,21E-06	-
CHAC1	chr15:41245635-41248717	3,93411	1,035	-1,92641	-1,92454	0,00025	0,00289273	76,0220009	-1,588867516	4,03627309	5,43E-05	0,00091554	-
CHAT	chr10:50817140-50873150	6,12102	0,70302	-3,12214	-2,4933	5,00E-05	0,00070533	207,157863	-2,058209404	4,60553844	4,11E-06	0,00011873	Motor Neuro
CHD5	chr1:6161846-6240194	3,52217	1,06357	-1,72755	-2,97084	5,00E-05	0,00070533	599,91322	-1,392483952	4,18351035	2,87E-05	0,00054885	-
CHGA	chr14:93389444-93401638	28,7132	3,45571	-3,05466	-4,73511	5,00E-05	0,00070533	775,869049	-2,100543374	4,6246634	3,75E-06	0,00011071	-
CHGB	chr20:5891973-5906005	9,35697	3,87515	-1,27179	-2,21145	5,00E-05	0,00070533	571,276702	-1,019683659	2,65170762	0,00800859	0,04080667	-
CHI3L2	chr1:111770280-111786062	19,408	115,585	2,57423	4,39545	5,00E-05	0,00070533	2888,971	2,008805394	-4,8850527	1,03E-06	3,92E-05	-
CHIT1	chr1:203185206-203198860	0,219586	12,6799	5,85161	4,95687	5,00E-05	0,00070533	463,181801	2,691747121	-5,3622994	8,22E-08	5,03E-06	-
CHRM2	chr7:136553398-136849088	5,8723	0,477063	-3,62167	-3,73863	5,00E-05	0,00070533	211,723654	-2,455275913	5,30054677	1,15E-07	6,55E-06	-
CHRNA1	chr2:175612322-175629200	0,0615363	1,82994	4,89421	2,78935	5,00E-05	0,00070533	60,7036817	2,884211388	-5,9001859	3,63E-09	4,09E-07	-
CHRNA7	chr15:32322685-32462384	0,713302	0,201592	-1,82307	-1,41894	0,00195	0,0162027	33,4987198	-1,366476622	3,60495696	0,00031221	0,00352041	-
CHRNB2	chr1:154540256-154552353	4,08328	0,85855	-2,24976	-3,12076	5,00E-05	0,00070533	325,894355	-1,817796468	4,58804209	4,47E-06	0,00012665	-
CIDEA	chr18:12254317-12277594	0,990934	0,149765	-2,72609	-1,54748	0,00195	0,0162027	15,7403884	-1,321292436	2,78700205	0,00531981	0,03015779	-
CILP	chr15:65488336-65503840	3,8445	0,908093	-2,08189	-2,84181	5,00E-05	0,00070533	287,509014	-1,485661762	3,2953068	0,00098314	0,00835894	-
CILP2	chr19:19649073-19657468	0,0564518	0,382225	2,75933	1,53776	0,00415	0,0296446	24,3413043	1,874714105	-3,9166704	8,98E-05	0,00133874	-
CISH	chr3:50643884-50649262	0,693094	3,13553	2,17759	2,22305	5,00E-05	0,00070533	96,1022441	1,747862662	-4,4051802	1,06E-05	0,00024617	-
CITED2	chr6:139693391-139695787	5,1489	11,1429	1,1138	1,76031	0,0001	0,00129637	351,506576	1,057320361	-4,8229725	1,41E-06	5,09E-05	-
CKMT1A	chr15:43985083-43991420	8,88487	1,58129	-2,49025	-3,11236	5,00E-05	0,00070533	204,394125	-1,796623942	3,89703114	9,74E-05	0,00142411	-
CLCN5	chrX:49687224-49863892	7,47908	3,2605	-1,19777	-1,19938	0,0007	0,00690182	1017,4654	-1,179690609	3,37465507	0,00073908	0,00677821	-
CLEC10A	chr17:6977855-6983600	0,110907	0,482938	2,12249	1,31133	0,00715	0,0457976	19,1149461	1,967616171	-5,6854142	1,30E-08	1,13E-06	-
CLEC2L	chr7:139208673-139229731	10,0381	2,24838	-2,15852	-2,50546	5,00E-05	0,00070533	145,122463	-1,548340093	3,59758687	0,00032118	0,00360981	-
CLEC5A	chr7:141627156-141646783	1,38436	4,34271	1,64938	2,40079	5,00E-05	0,00070533	317,684312	1,338358329	-4,4920425	7,05E-06	0,00018143	-
CLEC7A	chr12:10269375-10282868	7,72819	18,0135	1,22088	1,45732	0,00255	0,0201233	691,659551	1,016530571	-3,3674488	0,00075867	0,00693935	-
CLGN	chr4:141309606-141348815	2,81221	0,956307	-1,55616	-1,82928	5,00E-05	0,00070533	161,8348	-1,224112066	3,34241016	0,00083054	0,00741915	-
CLIC3	chr9:139889059-139891024	0,48298	3,0521	2,65976	1,60233	0,0044	0,0310999	33,1943053	1,738175315	-3,5586191	0,00037281	0,00405959	-
CLSTN2	chr3:139654026-140286919	8,32577	3,20881	-1,37555	-2,55112	5,00E-05	0,00070533	763,647016	-1,204981541	3,94961931	7,83E-05	0,00120971	Neuron
CLSTN3	chr12:7282966-7311530	22,9114	7,91807	-1,53284	-2,95502	5,00E-05	0,00070533	1461,41943	-1,249462658	3,62484917	0,00028913	0,00330537	-
CLVS2	chr6:123317115-123394064	8,71566	1,99926	-2,12414	-3,43378	5,00E-05	0,00070533	1317,88812	-1,649470995	3,82072507	0,00013306	0,00179574	-
CMAHP	chr6:25081294-25138620	4,40965	10,9849	1,31679	2,07601	5,00E-05	0,00070533	708,037283	1,104430344	-3,4216429	0,00062244	0,00600751	-
CMKLR1	chr12:108681820-108733094	2,72938	7,56234	1,47026	2,68563	5,00E-05	0,00070533	795,677317	1,331325219	-4,9006674	9,55E-07	3,66E-05	-
CNDP1	chr18:72201691-72252261	151,331	38,3874	-1,97901	-4,30603	5,00E-05	0,00070533	5306,46853	-1,934886815	11,8017037	3,82E-32	2,63E-28	-
CNNM1	chr10:101088855-101154087	2,24714	0,875788	-1,35943	-1,96693	5,00E-05	0,00070533	249,36211	-1,119967572	3,43705933	0,00058807	0,00574835	-
CNTN6	chr3:1134341-1445292	2,88292	0,774744	-1,89574	-2,48437	5,00E-05	0,00070533	226,874687	-1,387761044	4,09721423	4,18E-05	0,00074448	-
COL2A1	chr12:48366747-48398285	0,0691916	0,73339	3,40591	2,5898	5,00E-05	0,00070533	78,4132868	2,631079418	-5,8174146	5,98E-09	5,93E-07	-
COL4A1	chr13:110801309-110959496	14,1042	38,1828	1,43679	2,6425	5,00E-05	0,00070533	4763,64078	1,107392838	-2,6840484	0,00727366	0,03787331	-
COL5A3	chr19:10070236-10121147	17,5898	7,28075	-1,27258	-2,67702	5,00E-05	0,00070533	2122,02301	-1,132101137	5,80394646	6,48E-09	6,27E-07	-
COL8A1	chr3:99273152-99897476	7,19493	18,1121	1,3319	1,46341	0,0026	0,020422	1065,58699	1,107907368	-2,8489949	0,00438576	0,02629289	-
COMP	chr19:18893582-18902114	0,202495	6,8015	5,0699	4,33479	5,00E-05	0,00070533	293,478834	1,719000716	-3,4205281	0,000625	0,00602936	-
CORO2A	chr9:100883256-100954956	3,70984	0,62186	-2,5767	-3,42947	5,00E-05	0,00070533	311,601766	-1,983818166	4,95907182	7,08E-07	2,88E-05	-
CP	chr3:148847370-148939832	10,4482	50,4829	2,27254	3,68314	5,00E-05	0,00070533	4661,42648	2,135490329	-7,7766661	7,45E-15	3,84E-12	-
CPLX1	chr4:778744-819945	33,5346	5,54186	-2,59721	-4,34446	5,00E-05	0,00070533	671,115308	-2,110591523	5,47503942	4,37E-08	2,93E-06	-
CPLX2	chr5:175223609-175311023	26,9616	6,22312	-2,11519	-3,70742	5,00E-05	0,00070533	1638,18536	-1,527671959	3,41566182	0,00063627	0,00610828	-
CPM	chr12:69244955-69357020	5,51978	13,9983	1,34257	2,52992	5,00E-05	0,00070533	1810,06127	1,148526234	-3,1877501	0,00143384	0,01128858	Oligodendroc
CPNE6	chr14:24540045-24547309	28,1313	9,28281	-1,59954	-2,8567	5,00E-05	0,00070533	1020,0282	-1,267157304	3,46734142	0,00052563	0,00530908	-
CPNE9	chr3:9745509-9771592	4,81147	0,596628	-3,01157	-3,08677	5,00E-05	0,00070533	165,716992	-2,031809681	4,72103791	2,35E-06	7,65E-05	-
CPVL	chr7:29019582-29186153	4,66363	21,935	2,23371	3,03326	5,00E-05	0,00070533	660,400975	1,908272931	-5,5781005	2,43E-08	1,82E-06	-
CREG2	chr2:101964815-102003965	5,88773	1,6034	-1,87658	-2,63766	5,00E-05	0,00070533	322,234373	-1,447503733	3,37822998	0,00072954	0,00672506	-
CRTAC1	chr10:99609994-99790585	11,8504	4,31602	-1,45716	-1,5632	0,001	0,00924656	467,515854	-1,115633311	3,68872029	0,00022539	0,00269832	-
CSF3R	chr1:36931643-36948915	3,45553	7,40024	1,09867	1,90531	5,00E-05	0,00070533	588,415178	1,058744729	-4,8302808	1,36E-06	4,97E-05	-
CSTA	chr3:122044010-122060815	0,662155	3,51728	2,40922	1,94227	0,00015	0,00184356	48,9321945	2,040428725	-5,2718541	1,35E-07	7,43E-06	-

CTGF	chr6:132269316-132272518	22,6356	111,091	2,29507	4,10479	5,00E-05	0,00070533	3679,22011	1,722645802	-3,999062	6,36E-05	0,00103736	-
CTNNA2	chr2:79740059-80875988	19,2763	7,57909	-1,34674	-2,74953	5,00E-05	0,00070533	1430,34688	-1,284392622	8,55697921	1,16E-17	1,18E-14	-
CTSC	chr11:88026759-88070941	11,8963	29,142	1,29258	2,35565	5,00E-05	0,00070533	1169,03642	1,093313098	-4,5963608	4,30E-06	0,00012305	-
CTSH	chr15:79214091-79237420	16,3702	37,1989	1,18419	2,23654	5,00E-05	0,00070533	1158,59127	1,010746194	-3,2815431	0,00103241	0,00870962	-
CTSS	chr1:150702671-150738433	6,35597	25,1437	1,98401	3,9491	5,00E-05	0,00070533	1772,20042	1,819205742	-6,4530991	1,10E-10	1,98E-08	-
CUX2	chr12:111471827-111788358	0,710698	0,157027	-2,17823	-2,167	5,00E-05	0,00070533	82,3720182	-1,55150203	4,04203431	5,30E-05	0,00090069	-
CXCL14	chr5:134906370-134914969	9,77975	2,17925	-2,16597	-3,00956	5,00E-05	0,00070533	236,914281	-1,986109555	7,1808722	6,93E-13	2,38E-10	-
CXCL16	chr17:4634722-4643223	10,1573	21,1011	1,0548	1,68394	0,0001	0,00129637	720,844627	1,020400306	-4,5725571	4,82E-06	0,00013454	-
CXCR1	chr2:219027567-219031716	0,11546	0,655132	2,50439	1,66631	0,0036	0,0264814	24,8055606	1,681273301	-3,7551938	0,00017321	0,00218468	-
CXorf21	chrX:30576940-30596033	0,847287	2,07547	1,29252	1,41794	0,00165	0,0141149	86,0216718	1,165453095	-3,9515648	7,76E-05	0,00120351	-
CYBA	chr16:88709696-88717492	28,2995	69,4228	1,29463	2,26989	5,00E-05	0,00070533	664,225944	1,228804509	-4,4908888	7,09E-06	0,0001818	-
CYBB	chrX:37639269-37672714	15,9603	37,4358	1,22993	2,56507	5,00E-05	0,00070533	3064,00401	1,079002676	-3,3303934	0,00086723	0,00760797	-
CYBRD1	chr2:172378756-172414643	44,2541	101,172	1,19293	2,37306	5,00E-05	0,00070533	7260,5646	1,007976804	-2,9992259	0,00270667	0,01823443	Astrocyte
CYP24A1	chr20:52769987-52790516	0,516838	0,0871343	-2,5684	-1,52123	0,00295	0,0226522	31,8889715	-1,355506162	3,60520954	0,0003119	0,00352041	-
CYP26B1	chr2:72356366-72374991	0,430942	1,03968	1,27057	1,36577	0,0037	0,0271576	76,7407572	1,239527019	-3,9444924	8,00E-05	0,00122689	-
CYP4B1	chr1:47264669-47285021	0,390888	7,11297	4,18562	3,5667	5,00E-05	0,00070533	236,715292	1,862030318	-3,6778825	0,00023518	0,00279063	-
CYP51A1	chr7:91741462-91764059	106,592	45,688	-1,22221	-2,70611	5,00E-05	0,00070533	5330,17837	-1,212091758	8,20125912	2,38E-16	1,89E-13	-
DAB1	chr1:57463578-58716211	2,17302	0,839789	-1,3716	-1,63553	0,0003	0,00336918	154,057528	-1,055653899	4,52066692	6,16E-06	0,00016435	-
DAB2	chr5:39371775-39425335	6,98154	16,1237	1,20757	2,50904	5,00E-05	0,00070533	1596,30136	1,160528488	-8,0881773	6,06E-16	4,31E-13	-
DACT1	chr14:59100785-59115038	2,22142	5,68267	1,35509	2,15654	5,00E-05	0,00070533	357,481981	1,280184377	-4,9764785	6,48E-07	2,72E-05	-
DAPP1	chr4:100737980-100791346	0,633525	1,50286	1,24624	1,3728	0,0035	0,0258932	97,7830855	1,131870201	-3,5367365	0,0004051	0,00434702	-
DBNDD1	chr16:90071272-90114191	15,616	7,02285	-1,1529	-1,4854	0,00115	0,010427	547,916681	-1,098914375	7,18488889	6,73E-13	2,35E-10	-
DDIT4	chr10:74033676-74035797	59,6637	176,879	1,56784	3,30928	5,00E-05	0,00070533	4811,67847	1,466271185	-5,5614795	2,67E-08	1,95E-06	-
DENND2D	chr1:111728590-111747160	0,849458	3,66407	2,10883	2,70012	5,00E-05	0,00070533	254,461239	1,985699969	-11,630195	2,89E-31	1,49E-27	-
DENND3	chr8:142138719-142205900	5,18487	14,6677	1,50026	2,9852	5,00E-05	0,00070533	1853,37204	1,328662373	-5,3505903	8,77E-08	5,32E-06	-
DGAT2	chr11:75479777-75512581	3,16555	1,26015	-1,32886	-1,58513	0,0006	0,00609837	124,476058	-1,040234164	2,71142361	0,0066995	0,03570197	-
DGCR5	chr22:18958010-19018755	4,4439	1,73254	-1,35894	-1,74941	0,00035	0,00384337	231,21269	-1,180572317	4,26098607	2,04E-05	0,00041459	-
DGKK	chrX:50108405-50213737	0,475789	0,074141	-2,68198	-2,19243	0,0001	0,00129637	58,3602125	-1,493878022	3,15159347	0,00162382	0,01241473	-
DHCR24	chr1:55315299-55352921	244,327	90,2687	-1,43651	-2,72641	5,00E-05	0,00070533	16968,7237	-1,326500792	4,90789817	9,21E-07	3,54E-05	-
DHCR7	chr11:71145456-71159477	33,7439	15,7056	-1,10335	-2,3116	5,00E-05	0,00070533	1364,04594	-1,099127844	10,4375344	1,67E-25	4,93E-22	-
DIRAS1	chr19:2714564-2721390	8,37077	3,43719	-1,28413	-2,13785	5,00E-05	0,00070533	394,882212	-1,132275767	3,41407043	0,00064	0,00612542	-
DIRAS2	chr9:93372113-93405108	14,1838	6,1722	-1,20039	-2,26104	5,00E-05	0,00070533	921,35535	-1,027654065	2,84915822	0,00438351	0,02628703	-
DLGAP3	chr1:35331036-35370984	2,15213	0,681781	-1,65839	-1,90488	0,0001	0,00129637	114,85842	-1,341103106	4,4981439	6,85E-06	0,00017838	-
DMRT2	chr9:1050345-1057554	1,38188	0,530425	-1,38141	-1,40284	0,0035	0,0258932	64,7042051	-1,197137525	5,67258282	1,41E-08	1,19E-06	-
DMTN	chr8:21911080-21940036	14,8665	6,31361	-1,23553	-2,29313	5,00E-05	0,00070533	758,711631	-1,121730995	4,59458889	4,34E-06	0,00012358	-
DNAJC5B	chr8:66933790-67012755	0,0633513	0,783694	3,62884	1,83181	0,0017	0,0144616	21,8329674	2,540996089	-5,5568579	2,75E-08	1,98E-06	-
DNASE1L3	chr3:58178352-58196730	0,155808	2,7205	4,12603	2,5106	5,00E-05	0,00070533	57,3879803	2,387507416	-4,8153049	1,47E-06	5,26E-05	-
DNASE2B	chr1:84864214-84880691	0,291311	2,60095	3,15841	2,31637	0,0002	0,0023747	47,1201629	2,502854226	-6,1532323	7,59E-10	1,08E-07	-
DNM1	chr9:130928343-131017527	32,8002	7,35493	-2,15692	-2,26102	0,00065	0,00650426	1898,86858	-1,614046114	3,9636267	7,38E-05	0,00115926	-
DOK2	chr8:21766383-21771205	0,316869	1,03714	1,71065	1,3389	0,00375	0,0274168	33,0658656	1,534523911	-4,7994589	1,59E-06	5,61E-05	-
DOK6	chr18:67068283-67516322	4,2974	1,68883	-1,34744	-2,37376	5,00E-05	0,00070533	638,10571	-1,167110273	3,46361698	0,00053297	0,00535501	-
DPEP2	chr16:68021292-68033421	0,954816	2,11959	1,15049	1,17526	0,00675	0,0438202	114,537354	1,006174971	-2,7444713	0,00606085	0,03303369	-
DRAM1	chr12:102271104-102317401	2,21538	4,90788	1,14755	1,7934	5,00E-05	0,00070533	325,037176	1,05161062	-4,5218182	6,13E-06	0,00016367	-
DSC2	chr18:28645941-28742819	2,1689	6,06469	1,48347	2,52574	5,00E-05	0,00070533	584,269165	1,415617695	-7,2085773	5,65E-13	2,05E-10	-
DSC3	chr18:28569330-28622781	0,023093	0,596603	4,69124	2,94168	0,0001	0,00129637	63,9778704	3,083625933	-6,6739034	2,49E-11	5,71E-09	-
DSG2	chr18:29078026-29136874	0,236115	1,11747	2,24268	2,42763	5,00E-05	0,00070533	113,751005	1,784410315	-4,3732873	1,22E-05	0,00027631	-
DSP	chr6:7541869-7586946	2,95379	17,2415	2,54524	4,42317	5,00E-05	0,00070533	3438,40696	1,933530186	-4,4639254	8,05E-06	0,00020103	-
DYNC1I1	chr7:95401817-95739634	42,9139	16,827	-1,35067	-2,69964	5,00E-05	0,00070533	2373,57375	-1,132970749	3,57629521	0,0003485	0,00384559	-
EBI3	chr19:4229539-4237524	1,01927	3,34388	1,71398	1,6106	0,00075	0,00731751	62,6185149	1,60397962	-5,765971	8,12E-09	7,65E-07	-
ECHDC3	chr10:11784355-11806065	1,16851	2,93962	1,33096	1,41937	0,0013	0,0115379	81,7580394	1,150749987	-3,5337196	0,00040976	0,00438427	-
EDN1	chr6:12290528-12297427	2,44068	23,718	3,28063	4,47541	5,00E-05	0,00070533	803,532257	2,444279346	-5,6471858	1,63E-08	1,33E-06	-
EEF1A2	chr20:62119364-62130668	109,662	17,1521	-2,67661	-4,72768	5,00E-05	0,00070533	2676,37456	-1,664850532	3,42897265	0,00060587	0,0058889	-
EFNA3	chr1:155051347-155060014	3,93509	1,37048	-1,52171	-1,70484	0,00035	0,00384337	96,9316001	-1,162688602	3,33757078	0,00084514	0,00750086	-
EGFL6	chrX:13587693-13651694	0,207758	1,95267	3,23247	2,65203	5,00E-05	0,00070533	77,4940489	1,795664012	-3,5970728	0,00032182	0,00361302	-
EGR3	chr8:22545173-22550815	3,40453	1,09163	-1,64098	-2,1011	5,00E-05	0,00070533	170,538203	-1,271877451	3,08962153	0,00200412	0,01454624	-
EHD3	chr2:31456879-31491260	22,8619	8,77539	-1,38141	-2,74203	5,00E-05	0,00070533	1515,73454	-1,208044884	3,87691081	0,00010579	0,00151454	-
EIF4EBP1	chr8:37888019-37917883	3,52831	8,70694	1,30319	1,59075	0,00025	0,00289273	113,388191	1,288791374	-7,6696417	1,72E-14	8,28E-12	-
EIF5A2	chr3:170606203-170626426	10,553	3,86447	-1,44932	-2,51168	5,00E-05	0,00070533	806,972219	-1,308893802	3,65538497	0,0002568	0,00300567	-
ELAVL2	chr9:23690102-23826063	14,1401	2,43456	-2,53806	-3,55025	5,00E-05	0,00070533	746,4031	-1,825591671	4,03737308	5,41E-05	0,00091314	Neuron
ELAVL4	chr1:50513685-50667540	9,2756	1,71612	-2,43429	-3,11688	5,00E-05	0,00070533	344,092925	-1,819221924	4,33090528	1,48E-05	0,00032086	-
ELF4	chrX:129198894-129244688	0,865294	2,66908	1,62508	2,23794	5,00E-05	0,00070533	209,855176	1,499681798	-8,246889	1,63E-16	1,34E-13	-
ELMOD1	chr11:107461816-107537505	16,4889	6,02641	-1,45212	-2,53006	5,00E-05	0,00070533	830,402712	-1,19679413	3,45180481	0,00055685	0,00554004	-
ELOVL4	chr6:80624528-80657315	12,5362	3,06145	-2,03381	-3,12684	5,00E-05	0,00070533	503,006399	-1,688443724	4,37431168	1,22E-05	0,00027532	-
EMB	chr5:49692030-49737234	11,8111	5,11381	-1,20767	-2,08408	5,00E-05	0,00070533	849,584859	-1,034145719	2,73263231	0,00628304	0,03394883	-
EMP3	chr19:48828628-48833810	15,8305	38,4675	1,28093	2,1886	5,00E-05	0,00070533	489,79493	1,236376444	-6,63539	3,24E-11	7,18E-09	-
EMR2	chr19:14843508-14889353	0,662191	2,30631	1,80027	2,50186	5,00E-05	0,00070533	295,734121	1,580753813	-6,1717706	6,75E-10	9,81E-08	-
ENG	chr9:130577290-130617052	11,1247	25,3171	1,18634	2,2976	5,00E-05	0,00070533	1447,17083	1,020600503	-3,3379884	0,00084387	0,00749604	-
ENO2	chr12:7023613-7032859	139,195	38,8393	-1,84152	-3,42447	5,00E-05	0,00070533	4854,17668	-1,50322684	4,0403715	5,34E-05	0,00090561	Neuron
ENTPD3	chr3:40428646-40494799	5,90299	0,639589	-3,20623	-2,84044	5,00E-05	0,00070533	237,181733	-2,129596175	4,61014053	4,02E-06	0,00011679	-
EPB41L1	chr20:34679425-34820721	49,3698	21,01	-1,23255	-2,63039	5,00E-05	0,00070533	6285,38165	-1,185075186	5,18266997	2,19E-07	1,10E-05	-
EPB41L3	chr18:5392379-5628990	113,697	50,2483	-1,17805	-2,34059	5,00E-05	0,00070533	10232,5581	-1,087947054	4,0928528	4,26E-05	0,00075472	-
EPDR1	chr7:37960162-37991542	57,5474	23,5451	-1,28933	-2,55331	5,00E-05	0,00070533	2078,87826	-1,138977031	3,49955415	0,00046604	0,00483773	-
EPHA10	chr1:38181645-38230824	1,74047	0,662972	-1,39246	-1,66631	0,0003	0,00336918	146,107232	-1,104984106	3,9803941	6,88E-05	0,00109885	-
EPHX4	chr1:92495532-92529093	2,28972	0,708711	-1,6919	-1,54211	0,00035	0,00384337	45,6473368	-1,402773416	3,99484848	6,47E-05	0,00104935	-
EPN3	chr17:48610047-48621111	1,12002	0,231835	-2,27236	-2,07852	0,0003	0,00336918	65,5512253	-1,592791269	3,81591092	0,00013568	0,00182397	-
ERAP2	chr5:96211643-96255406	2,33263	5,62972	1,27111	2,23947	5,00E-05	0,00070533	649,610492	1,129135089	-4,00609	6,17E-05	0,00101662	-
ESR1	chr6:152011630-152424408	0,376359	1,39831	1,8935	2,01197	5,00E-05	0,00070533	144,624275	1,390533571	-3,2726199	0,00106556	0,00894175	-
ESRRG	chr1:216676587-217311097	7,90498	1,69256	-2,22355	-3,547	5,00E-05	0,00070533	639,211353	-1,824722064	5,32771608	9,95E-08	5,93E-06	-
F13A1	chr6:6144310-6320924	3,88247	14,5701	1,90797	3,49585	5,00E-05	0,00070533	1119,95369	1,650145809	-4,9074834	9,23E-07	3,54E-05	-
FABP3	chr1:31838099-31845923	28,3789	11,3698	-1,31961	-2,37656	5,00E-05	0,00070533	605,575857	-1,2188026	5,11221466	3,18E-07	1,50E-05	-
FABP4	chr8:82390731-82395473	0,479724	3,73333	2,96019	1,78126	0,0017	0,0144616	48,0873958	1,66514949	-3,3578661	0,00078547	0,00711569	-
FABP6	chr5:159614373-159665729	4,04465	1,06277	-1,92819	-1,59568	0,00075	0,00731751	58,440219	-1,708095967	6,93913935	3,94E-12	1,09E-09	-
FADS6	chr17:72873441-72889909	3,21714	0,695033	-2,21063	-2,27252	5,00E-05	0,00070533	90,9927426	-1,608909241	3,73311326	0,00018913	0,00233271	-
FAIM2	chr12:50260678-50297760	79,773	35,6582	-1,16167	-2,49868	5,00E-05	0,00070533	6043,26971	-1,074475447	4,57797733	4,69E-06	0,00013163	-
FAM135B	chr8:139142265-139509065	8,18718	2,46669	-1,73079	-3,05445	5,00E-05	0,00070533	1063,42889	-1,391686301	3,8407819	0,00012264	0,00168941	-
FAM180A	chr7:135414345-135433594	0,508521	2,19985	2,11302	1,89278	5,00E-05	0,00070533	81,2246422	1,346407041	-2,7635162	0,00571823	0,03180474	-
FAM19A1	chr3:68040733-68594771	1,16319	0,40908	-1,50764	-1,29119	0,0068	0,0440427	41,7499965	-1,019150973	2,69595612	0,00701869	0,03696307	-
FAM46B	chr1:27331510-27339333	0,562692	2,30755	2,03595	2,01098	5,00E-05	0,00070533	78,7498485	1,711113219	-4,4977673	6,87E-06	0,00017847	-
FAM57B	chr16:30034654-30042186	12,6514	4,04077	-1,64659	-2,39111	5,00E-05	0,00070533	277,325241	-1,394852282	4,21408765	2,51E-05	0,00049006	-
FAM83D	chr20:37554954-37581703	0,841319	0,26127	-1,68711	-1,28677	0,0056	0,0377854	31,2241984	-1,24598726	2,85466079	0,00430828	0,02591877	-
FASN	chr17:80036213-80056106	57,1476	25,3421	-1,17316	-2,52341	5,00E-05	0,00070533	7809,30157	-1,101261261	5,8952446	3,74E-09	4,20E-07	-
FAT4	chr4:126237566-126414087	3,46938	8,34043	1,26544	2,73448	5,00E-05	0,00070533	2658,81211	1,185840939	-6,4307468	1,27E-10	2,26E-08	-
FAXC	chr6:99720792-99797531	4,51733	1,23328	-1,87297	-3,04195	5,00E-05	0,00070533	647,111986	-1,56932502	4,10445872	4,05E-05	0,00073164	-
FBLIM1	chr1:16085254-16113084	2,36172	5,91256	1,32394	1,93321	0,0001	0,00129637	319,111059	1,085371154	-3,0217788	0,00251294	0,01730877	-
FBLL1	chr5:167956581-167957639	9,90476	2,52973	-1,96914	-2,27116	5,00E-05	0,00070533	104,518518	-1,534903333	3,80523691	0,00014167	0,00187996	-
FBLN1	chr22:45898718-45997014	36,0382	93,998	1,3831	2,36257	5,00E-05	0,00070533	4294,99184	1,130689971	-2,6921428	0,00709945	0,03725769	-
FBP1	chr9:97365420-97402531	0,99779	3,59844	1,85056	1,81131	5,00E-05	0,00070533	89,2478887	1,662193653	-5,3081886	1,11E-07	6,38E-06	-
FCER1A	chr1:159259503-159278014	0,281689	1,16774	2,05155	1,34047	0,0024	0,0191464	23,7160954	1,347132819	-2,8645323	0,00417626	0,02531641	-
FCER1G	chr1:161185086-161189038	13,2532	51,8952	1,96926	3,0591	5,00E-05	0,00070533	471,667625	1,800085991	-6,6182155	3,64E-11	7,85E-09	-
FCGBP	chr19:40353962-40440533	1,33403	7,70538	2,53008	4,59485	5,00E-05	0,00070533	1955,60482	2,055853684	-5,0527017	4,36E-07	1,97E-05	-
FCGR1A	chr1:149754244-149783928	1,5131	8,27298	2,4509	1,85025	0,00105	0,00963992	331,088716	2,167795608	-6,8806424	5,96E-12	1,60E-09	-
FCGR1B	chr1:120926127-120935944	0,644989	2,77376	2,10449	2,09287	0,0011	0,0100332	97,6383717	1,807935414	-5,5131558	3,52E-08	2,46E-06	-
FCGR1C	chr1:149369293-149378303	0,393562	2,4656	2,64728	2,14601	5,00E-05	0,00070533	73,5650625	2,183384061	-5,4343825	5,50E-08	3,56E-06	-
FCGR2A	chr1:161475204-161489360	9,79371	30,617	1,64441	3,31416	5,00E-05	0,00070533	1449,92325	1,491836171	-6,1206987	9,32E-10	1,29E-07	-
FCGR2B	chr1:161632904-161648444	1,50469	11,1352	2,88759	3,76785	5,00E-05	0,00070533	337,675859	2,198488918	-5,3883233	7,11E-08	4,42E-06	-
FCGR3A	chr1:161511550-161520413	7,42393	34,9338	2,23437	4,06267	5,00E-05	0,00070533	1334,89052	1,959360839	-5,9288017	3,05E-09	3,60E-07	-
FCGR3B	chr1:161592987-161601753	0,691259	2,6292	1,92733	2,10997	5,00E-05	0,00070533	65,6563174	1,449689626	-3,6902098	0,00022407	0,00268506	-
FCN3	chr1:27695600-27701315	0,160803	0,906934	2,4957	1,27902	0,00185	0,0155409	14,2366342	1,861765406	-4,0754206	4,59E-05	0,00080205	-
FCRLB	chr1:161691333-161697933	2,60027	0,982356	-1,40434	-1,35783	0,00335	0,0250259	66,4956381	-1,093050777	3,50186759	0,00046201	0,00480765	-
FER1L6	chr8:124864226-125183763	0,0410819	0,801632	4,28637	2,12367	0,0002	0,0023747	114,621696	2,763103275	-5,8068308	6,37E-09	6,26E-07	-
FERMT3	chr11:63974151-63993726	2,30618	7,25109	1,65269	1,9396	0,00035	0,00384337	352,146271	1,472459719	-5,506977	3,65E-08	2,53E-06	-
FGD2	chr6:36973422-36996845	2,97486	6,99526	1,23355	2,02671	5,00E-05	0,00070533	510,250664	1,191703852	-5,433483	5,53E-08	3,56E-06	-
FGF12	chr3:191857181-192445388	14,7555	3,55035	-2,05522	-3,64685	5,00E-05	0,00070533	1262,69189	-1,694083023	4,3234724	1,54E-05	0,00032877	-
FGF9	chr13:22245214-22278640	4,35569	0,585982	-2,89397	-3,46631	5,00E-05	0,00070533	231,421995	-2,247370423	5,31875028	1,04E-07	6,17E-06	-
FGFBP3	chr10:93666344-93669258	2,90652	1,19886	-1,27763	-1,56514	0,0008	0,00771573	101,790048	-1,194389007	5,65221971	1,58E-08	1,31E-06	-
FGL2	chr7:76751933-76924521	19,2764	51,1186	1,40702	2,40871	5,00E-05	0,00070533	3663,85953	1,132729564	-3,0783589	0,00208144	0,01497154	-
FGR	chr1:27938800-27961727	1,92441	8,25757	2,1013	3,09476	5,00E-05	0,00070533	392,631087	1,979744183	-7,9483359	1,89E-15	1,08E-12	-
FIBCD1	chr9:133777824-133814455	0,476286	0,0674247	-2,82048	-1,65098	0,0008	0,00771573	16,6313599	-2,049237688	5,47043577	4,49E-08	2,97E-06	-
FKBP5	chr6:35541361-35704724	7,38613	59,7852	3,0169	5,50311	5,00E-05	0,00070533	4193,84669	2,627681193	-7,5425601	4,61E-14	1,94E-11	-
FLJ42627	chr16:2688982-2696130	0,756148	0,29255	-1,36998	-1,62148	0,0002	0,0023747	113,653569	-1,178632247	3,80145803	0,00014385	0,00190031	-
FLT3	chr13:28577410-28674729	2,66574	1,02375	-1,38067	-1,88623	0,0002	0,0023747	215,089012	-1,143636219	3,90741897	9,33E-05	0,00137848	-

FNDC5	chr1:33327868-33338093	6,08983	2,37196	-1,36032	-1,97384	5,00E-05	0,00070533	304,440164	-1,339166656	6,31796502	2,65E-10	4,38E-08	-
FOLH1	chr11:49168186-49230222	41,656	20,3527	-1,0333	-2,21045	5,00E-05	0,00070533	2564,27831	-1,004592528	6,52663582	6,73E-11	1,31E-08	-
FOSL2	chr2:28607275-28637516	14,5298	37,177	1,3554	2,71898	5,00E-05	0,00070533	2350,81424	1,148679728	-3,32443	0,000886	0,00773371	-
FOXD2	chr1:47901688-47906363	0,383982	1,41332	1,87998	1,82333	0,00035	0,00384337	83,7550811	1,477268605	-3,2696562	0,00107678	0,00901762	-
FPR1	chr19:52249022-52255150	3,3236	16,1321	2,27911	2,9568	5,00E-05	0,00070533	299,821542	1,822289397	-4,7381131	2,16E-06	7,17E-05	-
FPR2	chr19:52264452-52273779	0,102107	1,00574	3,30011	1,96196	5,00E-05	0,00070533	23,4524865	2,104651133	-4,7699316	1,84E-06	6,35E-05	-
FPR3	chr19:52298410-52329334	1,02046	6,23056	2,61014	3,30478	5,00E-05	0,00070533	241,173548	2,140030327	-5,905522	3,52E-09	4,03E-07	-
FRAS1	chr4:78978723-79465423	1,41212	0,568612	-1,31234	-2,02432	5,00E-05	0,00070533	424,741731	-1,086369378	4,93100569	8,18E-07	3,22E-05	-
FREM3	chr4:144498560-144621828	1,08217	0,0775383	-3,80287	-3,29437	5,00E-05	0,00070533	107,957838	-2,577656581	5,61832763	1,93E-08	1,52E-06	-
FRMPD1	chr9:37651051-37746901	1,9335	0,506751	-1,93187	-2,42983	5,00E-05	0,00070533	184,887194	-1,586849382	5,72796101	1,02E-08	9,36E-07	-
FRMPD4	chrX:12156584-12742642	1,41393	0,371812	-1,92706	-2,56761	5,00E-05	0,00070533	203,146594	-1,611947629	4,43280822	9,30E-06	0,0002258	-
FSD1	chr19:4304590-4323843	6,28395	2,4569	-1,35484	-1,89762	5,00E-05	0,00070533	222,540331	-1,077652911	3,57448162	0,00035092	0,00386805	-
FSTL1	chr3:120113060-120169918	26,317	56,7742	1,10924	2,38693	5,00E-05	0,00070533	4393,38632	1,016702157	-4,329499	1,49E-05	0,00032239	-
FSTL4	chr5:132532151-132948223	1,17129	0,379921	-1,62433	-1,93516	5,00E-05	0,00070533	116,51719	-1,280055932	3,93846641	8,20E-05	0,00125159	-
FSTL5	chr4:162305043-163085186	11,9116	3,84814	-1,63013	-2,96358	5,00E-05	0,00070533	903,683199	-1,429039745	4,39973578	1,08E-05	0,00025073	-
FXYD5	chr19:35645624-35660788	7,83852	19,4079	1,30799	2,11593	5,00E-05	0,00070533	459,141119	1,19071611	-4,4660203	7,97E-06	0,00019948	-
FXYD7	chr19:35634153-35645205	17,0125	1,94353	-3,12985	-3,15483	5,00E-05	0,00070533	130,685046	-2,125907247	5,13341471	2,85E-07	1,37E-05	-
FYB	chr5:39105353-39270759	4,01695	10,9383	1,44521	2,79625	5,00E-05	0,00070533	1357,98617	1,363935197	-4,9896796	6,05E-07	2,58E-05	-
GAB3	chrX:153903526-153979858	1,67096	3,5837	1,10077	1,81771	5,00E-05	0,00070533	433,515861	1,092402263	-6,1347982	8,53E-10	1,19E-07	-
GABBR2	chr9:101050363-101471479	3,74498	1,06124	-1,8192	-2,70978	5,00E-05	0,00070533	358,77039	-1,406251455	3,51188478	0,00044494	0,00467245	-
GABRA2	chr4:46251580-46392056	6,89696	2,3383	-1,5605	-2,30149	5,00E-05	0,00070533	311,444531	-1,336735019	3,81268643	0,00013746	0,00184074	-
GABRA3	chrX:151335633-151619831	3,8573	1,18561	-1,70196	-2,09085	5,00E-05	0,00070533	191,515926	-1,379581842	3,46544083	0,00052936	0,00533109	-
GABRA5	chr15:27111865-27194357	1,35991	0,156567	-3,11866	-2,46063	5,00E-05	0,00070533	53,0698969	-1,880695209	3,90636287	9,37E-05	0,00138299	Neuron
GABRB3	chr15:26788693-27018935	7,06615	3,17404	-1,1546	-1,97661	5,00E-05	0,00070533	719,895313	-1,0149163	2,96300491	0,00304652	0,01992715	-
GABRG2	chr5:161494647-161582545	15,4885	2,39241	-2,69466	-3,86214	5,00E-05	0,00070533	813,632077	-1,672802937	3,41817705	0,00063042	0,00607317	Neuron
GAD1	chr2:171673199-171717659	3,35416	1,23914	-1,43661	-1,87411	5,00E-05	0,00070533	209,355576	-1,156428474	3,20644211	0,00134387	0,01078182	-
GAD2	chr10:26505235-26593491	1,69708	0,485856	-1,80446	-1,79471	0,00095	0,00886719	91,4034544	-1,19163716	2,62974	0,00854502	0,04277692	-
GADD45G	chr9:92219926-92221469	3,79787	13,7811	1,85943	2,41191	5,00E-05	0,00070533	194,096443	1,758538139	-5,4939067	3,93E-08	2,69E-06	-
GADL1	chr3:30767691-30936153	0,0467762	1,33295	4,83271	3,07322	0,0003	0,00336918	82,8002902	2,841228647	-5,9286095	3,06E-09	3,60E-07	-
GAL	chr11:68451982-68458643	2,71239	0,62788	-2,11101	-1,56949	0,00085	0,00810031	26,6494853	-1,456036563	3,45286294	0,00055467	0,00552662	-
GALNT9	chr12:132680916-132905905	6,97778	0,980923	-2,83056	-3,42645	5,00E-05	0,00070533	235,290517	-2,131244212	5,21162063	1,87E-07	9,54E-06	-
GAS1	chr9:89559276-89562104	2,48041	6,64916	1,42259	1,92392	0,0001	0,00129637	234,932	1,206276914	-2,9389581	0,00329318	0,02116969	-
GDA	chr9:74729510-74867140	0,189481	0,504765	1,41356	1,32136	0,0018	0,015174	49,6663458	1,182362921	-3,2805727	0,00103597	0,0087325	Neuron
GDAP1L1	chr20:42875738-42909557	2,48739	0,669236	-1,89404	-2,16833	5,00E-05	0,00070533	112,619529	-1,436614633	3,83236456	0,00012692	0,00173096	-
GGT5	chr22:24615621-24641110	9,23198	20,964	1,1832	2,13453	5,00E-05	0,00070533	949,657983	1,156557368	-4,3003745	1,71E-05	0,00035766	-
GJA5	chr1:147228331-147245466	1,53757	4,72004	1,61814	2,21711	5,00E-05	0,00070533	256,588765	1,311812733	-3,3169385	0,0009101	0,00790065	-
GJB2	chr13:20761603-20767114	2,19809	9,48444	2,10931	2,93089	5,00E-05	0,00070533	333,565611	1,459014272	-3,1274699	0,00176318	0,01322544	-
GLIPR1	chr12:75874512-75905418	5,57227	12,7239	1,1912	1,61567	0,0004	0,00431348	922,24448	1,074678851	-4,8619912	1,16E-06	4,33E-05	-
GLRA1	chr5:151202073-151304397	19,1028	2,12702	-3,16688	-4,06647	5,00E-05	0,00070533	486,275839	-1,957357156	4,00830191	6,12E-05	0,00101097	-
GLRA3	chr4:175563161-175750466	1,93884	0,432197	-2,16543	-2,15314	5,00E-05	0,00070533	104,235251	-1,608867797	3,85764965	0,00011448	0,00160159	-
GLRB	chr4:157997276-158093242	13,5923	4,33143	-1,64987	-2,74804	5,00E-05	0,00070533	615,905619	-1,405492651	3,78950141	0,00015095	0,00197248	-
GLT1D1	chr12:129338080-129469509	2,68156	0,886604	-1,59671	-1,88712	5,00E-05	0,00070533	142,804435	-1,198085382	3,35904593	0,00078212	0,00710033	-
GOLGA8S	chr15:23599894-23613471	0,52772	0,0584098	-3,17549	-2,05457	0,00025	0,00289273	42,0666358	-1,376877148	2,71989247	0,00653032	0,03497354	-
GOT1	chr10:101156626-101190530	35,5133	12,4888	-1,50773	-2,89686	5,00E-05	0,00070533	1348,39119	-1,317142082	4,26118621	2,03E-05	0,00041459	-
GPC5	chr13:92050872-93519487	11,9973	4,87358	-1,29966	-2,33994	5,00E-05	0,00070533	618,797776	-1,17821073	4,71461208	2,42E-06	7,84E-05	-
GPC5-AS1	chr13:92050872-93519487	0,0836635	1,20264	3,84546	0,366434	0,00725	0,0461216	10,7749835	2,246025666	-4,4595761	8,21E-06	0,00020417	-
GPC6	chr13:93879077-95060273	4,12992	10,6901	1,3721	2,68632	5,00E-05	0,00070533	1269,5899	1,159064264	-3,4806855	0,00050013	0,00511916	-
GPD1	chr12:50497601-50505103	58,0063	24,2811	-1,25638	-2,61245	5,00E-05	0,00070533	2982,83225	-1,133202896	3,64396972	0,00026847	0,00311924	-
GPNMB	chr7:23286315-23314729	16,0249	127,426	2,99127	5,38158	5,00E-05	0,00070533	5615,24676	2,296612338	-5,4948098	3,91E-08	2,68E-06	-
GPR123	chr10:134901507-134945179	5,28102	2,05857	-1,35918	-2,20145	5,00E-05	0,00070533	362,62822	-1,248929882	4,68933627	2,74E-06	8,71E-05	-
GPR158	chr10:25447000-25891157	11,4761	4,72571	-1,28003	-2,5695	5,00E-05	0,00070533	1443,32866	-1,144074005	3,72399228	0,0001961	0,00240431	-
GPR160	chr3:169755734-169803183	0,947726	2,2342	1,23721	1,30891	0,00235	0,018855	68,1619235	1,194040705	-5,0149109	5,31E-07	2,30E-05	-
GPR4	chr19:46093022-46105466	0,749776	4,56388	2,60573	2,94718	5,00E-05	0,00070533	180,348736	1,987496612	-4,9218521	8,57E-07	3,33E-05	-
GPR65	chr14:88471467-88481155	1,17585	3,8821	1,72313	2,48564	5,00E-05	0,00070533	265,680582	1,571395739	-5,8328707	5,45E-09	5,58E-07	-
GPR85	chr7:112720467-112727833	7,32961	3,06762	-1,25661	-2,21205	5,00E-05	0,00070533	563,152315	-1,199781495	4,23160605	2,32E-05	0,00045905	-
GPR88	chr1:101003727-101007583	0,447303	2,51463	2,49102	2,5238	5,00E-05	0,00070533	104,948279	1,998618042	-5,044631	4,54E-07	2,03E-05	Neuron
GPRIN2	chr10:46993545-47000568	2,74973	0,647998	-2,08523	-1,76872	0,00125	0,0111709	74,3137002	-1,59799303	3,90169771	9,55E-05	0,00140289	-
GPX1	chr3:49394608-49395791	73,3211	158,178	1,10925	2,32153	5,00E-05	0,00070533	1977,31971	1,051850622	-3,8387154	0,00012368	0,00169916	-
GPX3	chr5:150399998-150408554	20,8838	78,8778	1,91724	3,42301	5,00E-05	0,00070533	2278,04791	1,458506079	-3,3636477	0,0007692	0,00702317	-
GRAMD1B	chr11:123396343-123498479	18,0604	7,29856	-1,30714	-2,66344	5,00E-05	0,00070533	2632,11971	-1,130373215	3,74838229	0,00017798	0,00222987	-
GRB14	chr2:165349322-165478360	2,98704	1,02436	-1,544	-1,79158	5,00E-05	0,00070533	140,843633	-1,153106272	3,11860552	0,00181709	0,01354612	-
GREM2	chr1:240652872-240775462	2,43308	0,611178	-1,99312	-2,47925	5,00E-05	0,00070533	146,063533	-1,592851007	4,40311229	1,07E-05	0,00024797	-
GRIN1	chr9:140033608-140064491	17,9087	3,53297	-2,34171	-3,88071	5,00E-05	0,00070533	948,642428	-1,677502425	3,74800566	0,00017825	0,00223186	-
GRIN2A	chr16:9847261-10276611	5,76592	2,24503	-1,36082	-2,6746	5,00E-05	0,00070533	1429,33709	-1,237571856	4,00180312	6,29E-05	0,00102994	-
GRIN2B	chr12:13714409-14133022	3,19885	0,726611	-2,1383	-3,03649	5,00E-05	0,00070533	322,784304	-1,646287508	4,26641426	1,99E-05	0,00040664	-
GRM3	chr7:86273229-86494192	19,6443	8,89014	-1,14383	-2,40329	5,00E-05	0,00070533	1524,00212	-1,087416814	5,5806406	2,40E-08	1,81E-06	-
GRM4	chr6:33986419-34123399	0,928878	0,229668	-2,01594	-1,99319	5,00E-05	0,00070533	98,8416544	-1,498199147	3,5675014	0,0003604	0,00395405	-
GRM5	chr11:88237743-88796846	5,86651	2,00303	-1,55031	-2,73944	5,00E-05	0,00070533	733,320884	-1,385878466	4,97197185	6,63E-07	2,76E-05	-
GRP	chr18:56887399-56898002	1,31846	0,242614	-2,44212	-1,43571	0,00645	0,0421659	15,039543	-1,883628542	5,3020643	1,15E-07	6,53E-06	-
GUCY1A2	chr11:106544737-106889171	12,7523	3,83692	-1,73274	-3,28733	5,00E-05	0,00070533	2940,98636	-1,540340446	4,55703781	5,19E-06	0,00014243	-
GYPC	chr2:127413510-127454251	8,23425	21,7261	1,39972	2,32869	5,00E-05	0,00070533	461,10413	1,312132711	-5,3142508	1,07E-07	6,26E-06	-
H19	chr11:2016405-2019065	1,05023	8,0544	2,93907	3,06241	5,00E-05	0,00070533	242,348333	1,570695077	-3,1101617	0,00186985	0,01381961	-
HAMP	chr19:35773409-35776045	1,84753	11,189	2,59841	2,12473	0,0001	0,00129637	107,608046	1,577411754	-3,5150972	0,00043959	0,00463516	-
HAPLN1	chr5:82934016-83016896	0,577412	0,208475	-1,46973	-1,29848	0,002	0,0165609	47,6311409	-1,09594185	2,63986363	0,00829394	0,04184485	Astrocyte
HAPLN4	chr19:19366451-19373596	12,4681	1,91252	-2,70469	-3,87986	5,00E-05	0,00070533	483,689557	-1,891350058	4,10406294	4,06E-05	0,00073225	-
HAVCR2	chr5:156512842-156536248	7,09857	18,0464	1,34611	2,50192	5,00E-05	0,00070533	873,112059	1,177318285	-4,3983298	1,09E-05	0,00025207	-
HCK	chr20:30639990-30689657	3,13074	9,17482	1,55118	2,45281	5,00E-05	0,00070533	434,928022	1,344746852	-4,5828725	4,59E-06	0,00012929	-
HCLS1	chr3:121350244-121379795	6,6239	23,4881	1,82618	3,43181	5,00E-05	0,00070533	1095,41253	1,750048402	-8,2646259	1,40E-16	1,22E-13	-
HCN1	chr5:45255051-45696220	4,07743	1,11522	-1,87033	-2,84585	5,00E-05	0,00070533	559,147431	-1,413425092	3,27861144	0,00104319	0,00877906	-
HCN2	chr19:589892-617159	48,768	16,9012	-1,52881	-3,08842	5,00E-05	0,00070533	2030,96519	-1,303199786	4,00816501	6,12E-05	0,00101097	-
HCN4	chr15:73612199-73661605	0,640228	0,158084	-2,01789	-1,97886	5,00E-05	0,00070533	57,547866	-1,564983482	3,95379289	7,69E-05	0,00119525	-
HCRTR2	chr6:55039070-55147418	0,680517	0,107102	-2,66764	-1,60001	0,00175	0,0148269	17,0547462	-1,498381002	3,20780996	0,0013375	0,01074941	-
HECW1	chr7:43152197-43602938	5,22901	1,21194	-2,10922	-3,35641	5,00E-05	0,00070533	619,918168	-1,581720881	4,17161352	3,02E-05	0,00057258	-
HES5	chr1:2460183-2461684	2,00176	0,513762	-1,9621	-1,59749	0,00095	0,00886719	27,3265441	-1,522663731	4,53637862	5,72E-06	0,00015559	-
HGF	chr7:81331443-81399452	2,1737	5,84091	1,42604	2,08315	5,00E-05	0,00070533	342,782309	1,368222993	-6,0692398	1,29E-09	1,69E-07	-
HHIP	chr4:145564067-145659881	20,5998	9,22347	-1,15925	-2,34105	5,00E-05	0,00070533	1424,96042	-1,121521758	5,90080563	3,62E-09	4,09E-07	-
HIF3A	chr19:46720994-46846690	5,15816	11,6629	1,177	1,84055	5,00E-05	0,00070533	1056,77065	1,074403356	-3,7583315	0,00017105	0,00216011	-
HILPDA	chr7:128095883-128098472	24,0067	71,0102	1,56459	2,94975	5,00E-05	0,00070533	1511,84267	1,296241019	-3,5321071	0,00041226	0,00440298	-
HIST1H1B	chr6:27834569-27835359	2,07419	7,06237	1,7676	1,86272	5,00E-05	0,00070533	96,4295285	1,507842236	-3,7685916	0,00016417	0,00210023	-
HIST1H1D	chr6:26234439-26235216	10,8675	32,77	1,59236	2,46073	5,00E-05	0,00070533	457,525661	1,398088916	-3,8660694	0,0001106	0,00155895	-
HIST1H2AG	chr6:27100816-27101314	5,17816	11,9454	1,20595	1,28904	0,0031	0,0235351	81,6952734	1,129186896	-3,64241	0,0002701	0,00313292	-
HIST1H2AL	chr6:27833106-27833576	2,60823	9,18918	1,81686	1,6664	5,00E-05	0,00070533	49,7074673	1,652158216	-5,4757992	4,36E-08	2,93E-06	-
HIST1H2AM	chr6:27860476-27860963	4,49347	12,8872	1,52004	1,61239	0,00015	0,00184356	75,9613475	1,436417356	-5,180098	2,22E-07	1,11E-05	-
HIST1H2BH	chr6:26251878-26252303	3,13117	8,23731	1,39547	1,22711	0,0036	0,0264814	52,6508202	1,330608859	-3,6617922	0,00025046	0,00294651	-
HIST1H2BI	chr6:26273203-26273640	2,85763	8,05925	1,49583	1,34535	0,0019	0,0158579	52,3763758	1,314179026	-3,778494	0,00015778	0,00204252	-
HIST1H2BO	chr6:27861202-27861669	1,50199	5,10537	1,76515	1,38609	0,00315	0,0238285	29,2210637	1,506700624	-3,7814114	0,00015594	0,00202254	-
HIST1H3B	chr6:26031816-26032288	1,85956	7,97953	2,10135	1,66455	0,001	0,00924656	46,996545	1,447525846	-3,0855719	0,00203161	0,01470727	-
HIST1H3F	chr6:26250369-26250835	1,4052	6,26946	2,15757	1,68304	0,0002	0,0023747	34,9499636	1,643256129	-4,2156083	2,49E-05	0,00048723	-
HIST1H3H	chr6:27777841-27778314	3,33736	10,7118	1,68242	1,57033	0,0003	0,00336918	56,7956304	1,389934462	-3,4327785	0,00059743	0,00582254	-
HIST1H4F	chr6:26240653-26241021	7,25543	15,6524	1,10925	1,12229	0,00775	0,048585	72,5871855	1,045748564	-3,4546428	0,00055102	0,00550226	-
HK2	chr2:75059781-75120481	4,08273	8,51885	1,06113	2,14076	5,00E-05	0,00070533	1243,32481	1,018559727	-4,9398904	7,82E-07	3,10E-05	-
HLA-C	chr6_ssto_hap7:2570004-2573382	6,85124	18,5932	1,44034	2,08169	5,00E-05	0,00070533	2867,90849	1,108323828	-3,4248738	0,00061509	0,00595283	-
HMGCR	chr5:74632992-74657926	30,232	11,7184	-1,36731	-2,9522	5,00E-05	0,00070533	2511,95039	-1,321766157	7,65492526	1,93E-14	9,07E-12	-
HMGCS1	chr5:43287571-43313614	47,6251	17,4488	-1,44859	-3,07996	5,00E-05	0,00070533	4271,8678	-1,418382814	6,66983547	2,56E-11	5,78E-09	-
HOXA10	chr7:27202056-27219880	8,03707	2,72569	-1,56005	-1,69875	0,00015	0,00184356	242,105289	-1,323274424	3,91059498	9,21E-05	0,00136476	-
HOXD10	chr2:176981491-176984670	2,71507	0,39986	-2,76342	-1,75801	0,00145	0,0126751	71,999926	-1,414768407	2,79782169	0,00514485	0,02940831	-
HPGD	chr4:175411327-175444049	0,43399	1,05128	1,27641	1,1973	0,00695	0,0447726	53,4435574	1,092434581	-3,8534414	0,00011647	0,00162388	-
HPSE	chr4:84213613-84256306	1,07898	4,52131	2,06708	2,81787	5,00E-05	0,00070533	334,340348	1,701748581	-4,5912221	4,41E-06	0,00012508	-
HRH1	chr3:11178778-11304939	1,44147	4,19682	1,54175	2,38119	5,00E-05	0,00070533	332,576629	1,355707629	-4,6572513	3,20E-06	9,77E-05	-
HRH2	chr5:175085039-175113245	0,670183	1,93052	1,52636	1,60331	0,00055	0,00564481	92,9849397	1,246608591	-3,2978298	0,00097435	0,00830473	-
HRH3	chr20:60790016-60795323	1,97762	0,582314	-1,7639	-1,70998	0,0003	0,00336918	58,5247339	-1,351741934	3,57082868	0,00035585	0,00390795	-
HS3ST5	chr6:114376749-114384041	3,74883	1,49375	-1,32751	-1,79952	0,0001	0,00129637	174,403038	-1,205093966	4,66618336	3,07E-06	9,49E-05	-
HSD17B6	chr12:57157001-57181574	6,59136	2,46585	-1,41849	-1,91491	5,00E-05	0,00070533	191,591692	-1,239675562	6,37400677	1,84E-10	3,10E-08	-
HSPA12A	chr10:118430702-118502085	60,6089	15,7945	-1,94011	-3,54957	5,00E-05	0,00070533	5469,5127	-1,634214538	4,49284767	7,03E-06	0,00018107	-
HTR2A	chr13:47405676-47471211	3,58361	1,39962	-1,35638	-1,97382	5,00E-05	0,00070533	318,368101	-1,097323132	2,74256096	0,00609621	0,03318263	-
HTR2C	chrX:113818550-114144624	5,95028	1,3209	-2,17144	-2,89134	5,00E-05	0,00070533	360,394514	-1,562797387	3,42621941	0,00061205	0,00593495	Neuron
HTR5A	chr7:154858778-154879102	0,925137	0,228871	-2,01513	-1,63797	0,00175	0,0148269	60,7449628	-1,508260355	3,54205436	0,00039702	0,00427588	-
HTRA4	chr8:38831667-38846181	0,0522202	1,05647	4,3385	2,30799	0,0003	0,00336918	49,7207121	2,700552923	-6,1021946	1,05E-09	1,40E-07	-
HYAL2	chr3:50355220-50360281	11,7449	31,6612	1,43068	2,75158	5,00E-05	0,00070533	1025,8148	1,290230972	-5,0462635	4,51E-07	2,02E-05	-
HYAL3	chr3:50330258-50336899	3,64759	0,987364	-1,88529	-1,40969	0,0039	0,0283163	80,5680158	-1,382943625	3,3512855	0,00080437	0,00721977	-
ICOSLG	chr21:45642877-45660887	15,184	6,83705	-1,1511	-2,15696	5,00E-05	0,00070533	936,609208	-1,009903075	3,85742163	0,00011459	0,001602	-

ID3	chr1:23884420-23886285	48,3201	102,542	1,08552	2,21486	5,00E-05	0,00070533	2199,63913	1,035865331	-3,7938072	0,00014836	0,00194492	-
IDI1	chr10:1064846-1095061	75,687	35,2936	-1,10064	-2,37378	5,00E-05	0,00070533	2846,16959	-1,097697761	7,46680209	8,22E-14	3,26E-11	-
IER5L	chr9:131937830-131940540	6,89536	14,6585	1,08804	1,82241	5,00E-05	0,00070533	516,158005	1,001296574	-2,8437489	0,00445862	0,02658294	-
IFI16	chr1:158979681-159024945	12,4642	27,8715	1,161	2,44944	5,00E-05	0,00070533	2229,71502	1,095625979	-5,6499276	1,61E-08	1,32E-06	-
IFI30	chr19:18284589-18288934	7,17506	30,0419	2,06591	3,24983	5,00E-05	0,00070533	528,342663	1,675165774	-5,2450663	1,56E-07	8,24E-06	-
IFITM1	chr11:313990-315272	77,3463	164,103	1,0852	2,25986	5,00E-05	0,00070533	1965,75159	1,01580198	-3,9830194	6,80E-05	0,00108904	-
IFITM2	chr11:308106-309410	41,3698	110,688	1,41985	2,75094	5,00E-05	0,00070533	1063,01177	1,298141496	-5,4730441	4,42E-08	2,94E-06	-
IFITM3	chr11:319672-320914	146,329	345,592	1,23986	2,64532	5,00E-05	0,00070533	3299,88559	1,166000441	-4,7338835	2,20E-06	7,30E-05	-
IFNLR1	chr1:24480646-24513765	0,693429	1,59685	1,20341	1,54976	0,0004	0,00431348	138,996255	1,110104736	-5,9328634	2,98E-09	3,55E-07	-
IGF1	chr12:102789644-102874378	1,03505	4,72989	2,19211	2,13863	5,00E-05	0,00070533	372,509875	1,494768268	-3,3308106	0,00086594	0,00760488	-
IGFBP3	chr7:45951843-45960871	7,98464	19,315	1,27442	2,28015	5,00E-05	0,00070533	873,547491	1,044441459	-2,6351968	0,00840885	0,04225929	-
IGFBP4	chr17:38599675-38613982	15,7616	49,0762	1,63861	3,10578	5,00E-05	0,00070533	1531,40529	1,336524811	-3,5324014	0,0004118	0,00440061	-
IGFBP5	chr2:217536827-217560272	29,2747	78,952	1,43132	2,90384	5,00E-05	0,00070533	8844,98719	1,304105796	-4,3591503	1,31E-05	0,00028847	-
IGLON5	chr19:51815101-51834102	8,17344	1,84428	-2,14789	-3,05571	5,00E-05	0,00070533	340,845816	-1,731437212	4,51865625	6,22E-06	0,00016528	-
IKZF1	chr7:50343678-50472798	2,44667	6,02611	1,30041	1,73985	5,00E-05	0,00070533	591,108863	1,216243853	-4,6165289	3,90E-06	0,00011438	-
IL10RA	chr11:117857105-117872199	3,8531	9,78549	1,34463	2,37786	5,00E-05	0,00070533	753,761502	1,153558275	-3,922751	8,75E-05	0,00131284	-
IL13RA1	chrX:117861558-117928496	15,9822	41,5035	1,37676	3,00098	5,00E-05	0,00070533	3049,77627	1,315474252	-9,2421238	2,42E-20	4,53E-17	-
IL18	chr11:112013973-112034840	3,2653	14,1873	2,11931	2,92808	5,00E-05	0,00070533	299,850959	1,859253549	-6,6020093	4,06E-11	8,45E-09	-
IL18R1	chr2:102972742-103015235	0,471039	2,16345	2,19942	2,40817	5,00E-05	0,00070533	164,290677	1,745965345	-4,3581249	1,31E-05	0,0002892	-
IL18RAP	chr2:103035249-103069024	0,197631	0,810227	2,03551	1,67725	0,00095	0,00886719	55,2803358	1,702032021	-4,6447791	3,40E-06	0,00010256	-
IL1R1	chr2:102686835-102796334	4,56273	17,3624	1,92799	3,69101	5,00E-05	0,00070533	1601,97698	1,626354652	-5,1006248	3,39E-07	1,57E-05	-
IL1R2	chr2:102608305-102644884	0,113094	0,721743	2,67396	1,34511	0,0018	0,015174	25,9746571	2,002865935	-4,4221105	9,77E-06	0,00023425	-
IL20RB	chr3:136676706-136729926	0,157751	0,644966	2,03157	1,48696	0,00105	0,00963992	31,4784473	1,623065858	-4,2019472	2,65E-05	0,00051273	-
IL2RG	chrX:70327253-70331481	0,663853	1,65783	1,32036	1,26825	0,0058	0,0387613	57,49983	1,234533096	-5,1917238	2,08E-07	1,05E-05	-
IL4R	chr16:27325229-27376099	3,74395	16,1552	2,10936	3,61684	5,00E-05	0,00070533	1087,69489	1,769086075	-5,4310935	5,60E-08	3,59E-06	-
IL7R	chr5:35856976-35879705	0,623903	1,77108	1,50524	1,85816	0,0002	0,0023747	172,646646	1,247142396	-3,2604941	0,00111218	0,00924946	-
IMPDH1	chr7:128032330-128050036	23,5615	8,86473	-1,41028	-2,43143	5,00E-05	0,00070533	936,25319	-1,006458755	2,61342439	0,00896399	0,04436843	-
INA	chr10:105036919-105050108	25,9855	5,65681	-2,19965	-3,63526	5,00E-05	0,00070533	1144,12165	-1,358943385	2,76000762	0,00578	0,03200093	-
INPP5F	chr10:121485558-121588662	59,8947	21,9455	-1,4485	-2,7204	5,00E-05	0,00070533	4437,80773	-1,135044693	3,90429457	9,45E-05	0,00139287	-
INSIG1	chr7:155089485-155101945	81,2824	35,9198	-1,17817	-2,5213	5,00E-05	0,00070533	3787,75907	-1,105365743	4,48734942	7,21E-06	0,00018416	-
IQSEC3	chr12:176048-287625	5,65782	1,81803	-1,63787	-2,69996	5,00E-05	0,00070533	529,596489	-1,421903107	4,52641387	6,00E-06	0,0001614	-
IRAK3	chr12:66582977-66648394	2,33794	7,50122	1,68189	3,11311	5,00E-05	0,00070533	1094,60704	1,54254638	-5,7557206	8,63E-09	8,06E-07	-
ISL2	chr15:76629146-76634816	0,855887	0,0876419	-3,28773	-1,86264	0,0014	0,0122699	17,8432215	-1,791088195	3,74845643	0,00017793	0,00222987	Motor Neuro
ITGA11	chr15:68594041-68724492	0,623317	1,78913	1,52122	1,99104	5,00E-05	0,00070533	219,491424	1,330778874	-3,7011659	0,00021461	0,0025928	-
ITGA5	chr12:54747474-54867386	4,61117	16,773	1,86294	2,029	0,0002	0,0023747	1535,68499	1,539786545	-4,7525294	2,01E-06	6,78E-05	-
ITGB2	chr21:46305867-46349595	10,2694	31,1914	1,60279	3,06304	5,00E-05	0,00070533	1775,64479	1,40863907	-4,2115914	2,54E-05	0,00049457	-
ITGB3	chr17:45331207-45401267	1,022	3,91684	1,93829	2,79946	5,00E-05	0,00070533	320,265384	1,655716336	-5,5640409	2,64E-08	1,94E-06	-
ITGBL1	chr13:102104943-103054124	7,78817	18,0568	1,21318	1,72798	0,00045	0,00473416	1178,07796	1,101743183	-3,9073244	9,33E-05	0,00137848	-
ITPRIPL1	chr2:96991061-96994091	0,465482	1,41602	1,60504	1,41997	0,003	0,0229313	46,2197012	1,47450418	-5,5859112	2,32E-08	1,77E-06	-
JAK3	chr19:17935592-17958841	1,21089	4,83815	1,99839	3,05799	5,00E-05	0,00070533	413,615111	1,810910196	-6,1032222	1,04E-09	1,40E-07	-
JPH3	chr16:87635440-87731761	12,5023	3,40381	-1,87698	-3,1896	5,00E-05	0,00070533	656,204607	-1,622208071	4,83603224	1,32E-06	4,85E-05	-
KCNA1	chr12:5019072-5027422	16,4768	1,48259	-3,47425	-5,31514	5,00E-05	0,00070533	1642,65865	-2,317280162	4,85858335	1,18E-06	4,39E-05	-
KCNA2	chr1:111136201-111174096	18,5984	5,86769	-1,66431	-2,91433	5,00E-05	0,00070533	762,515074	-1,477616383	5,25333873	1,49E-07	8,03E-06	-
KCNAB2	chr1:5922867-6161253	28,3818	7,92244	-1,84095	-2,99212	5,00E-05	0,00070533	1562,36967	-1,444588467	3,70980049	0,00020742	0,00252071	-
KCNC1	chr11:17757494-17804602	6,40707	1,55735	-2,04057	-2,75487	5,00E-05	0,00070533	324,362666	-1,442664678	3,49207445	0,00047929	0,00494008	-
KCNC2	chr12:75433857-75603528	2,48042	1,00634	-1,30147	-1,66091	0,0001	0,00129637	204,822077	-1,10809881	2,63388509	0,00844141	0,04239192	Neuron
KCNC3	chr19:50815198-50836772	12,4224	3,10086	-2,00221	-3,07336	5,00E-05	0,00070533	467,889685	-1,655351052	4,41000195	1,03E-05	0,0002435	-
KCNE1L	chrX:108866928-108868393	6,31197	2,2088	-1,51482	-1,87776	0,0001	0,00129637	120,21321	-1,346813826	4,70777094	2,50E-06	8,07E-05	-
KCNE3	chr11:74165885-74178600	1,44255	3,31532	1,20053	1,661	0,0001	0,00129637	192,231018	1,132216946	-4,233874	2,30E-05	0,00045619	-
KCNE4	chr2:223916647-223920357	3,58123	13,3235	1,89544	3,29541	5,00E-05	0,00070533	664,887895	1,76408524	-6,8820841	5,90E-12	1,60E-09	-
KCNG1	chr20:49620192-49639675	2,03805	0,660733	-1,62505	-1,64884	0,0009	0,00846161	63,9549435	-1,329943394	3,93855804	8,20E-05	0,00125159	-
KCNG4	chr16:84254740-84273356	1,94018	0,386639	-2,32713	-2,29612	5,00E-05	0,00070533	76,5417542	-1,835890173	4,48558988	7,27E-06	0,00018478	-
KCNH5	chr14:63173290-63511956	1,42191	0,454209	-1,6464	-1,62237	0,00065	0,00650426	98,9556561	-1,16434909	2,71139227	0,00670013	0,03570197	-
KCNJ10	chr1:160007256-160040051	86,7556	38,7911	-1,16123	-2,49081	5,00E-05	0,00070533	8085,31635	-1,144854684	6,52820981	6,66E-11	1,31E-08	-
KCNJ12,KCNJ	chr17:21279698-21323179	2,92207	0,720939	-2,01904	-2,54271	5,00E-05	0,00070533	102,209176	-1,524797032	3,79756643	0,00014612	0,00192177	-
KCNJ14	chr19:48958963-48969367	3,14301	0,659373	-2,25298	-2,58755	5,00E-05	0,00070533	132,443987	-1,725609915	5,27819115	1,30E-07	7,20E-06	-
KCNJ15	chr21:39601836-39673746	0,146683	0,50885	1,79454	1,36032	0,00015	0,00184356	29,4228379	1,337260779	-3,1002551	0,00193354	0,01419374	-
KCNJ16	chr17:68071365-68131746	55,3486	12,187	-2,1832	-4,48442	5,00E-05	0,00070533	3182,22825	-2,068720922	8,10244054	5,39E-16	3,97E-13	-
KCNJ5	chr11:128761312-128787951	1,47848	5,15094	1,80072	2,20302	5,00E-05	0,00070533	269,213952	1,576382383	-4,7150674	2,42E-06	7,84E-05	-
KCNK3	chr2:26915580-26954066	7,7852	1,90035	-2,03447	-3,1749	5,00E-05	0,00070533	383,960063	-1,744452108	5,22940218	1,70E-07	8,86E-06	-
KCNK4	chr11:64058792-64067503	1,07886	0,168376	-2,67974	-1,67834	0,0042	0,0299381	24,0023542	-1,327235084	3,03369746	0,00241577	0,0168504	-
KCNK6	chr19:38810483-38819649	0,648903	1,52626	1,23393	1,30094	0,00265	0,0207565	66,4953221	1,13722596	-4,9422051	7,72E-07	3,07E-05	-
KCNK9	chr8:140613080-140715299	4,80407	0,628834	-2,93351	-3,11965	5,00E-05	0,00070533	170,929258	-2,17507493	4,92325413	8,51E-07	3,31E-05	-
KCNN1	chr19:18062110-18109930	5,04762	1,15662	-2,12569	-2,67657	5,00E-05	0,00070533	201,544706	-1,777225461	5,27007843	1,36E-07	7,48E-06	-
KCNQ2	chr20:62037541-62103993	9,1524	3,32131	-1,4624	-1,76668	0,00015	0,00184356	342,034934	-1,409292847	3,86649521	0,00011041	0,00155895	Neuron
KCNT1	chr9:138594030-138684993	2,36313	0,699536	-1,75623	-2,22281	5,00E-05	0,00070533	195,504873	-1,335649729	3,53186739	0,00041264	0,00440298	-
KIAA0040	chr1:175126122-175162229	1,92913	6,41457	1,7334	2,74864	5,00E-05	0,00070533	557,136493	1,431770576	-4,0631598	4,84E-05	0,00083459	-
KIAA0513	chr16:85061356-85127828	30,326	11,6153	-1,38452	-2,8579	5,00E-05	0,00070533	3692,22859	-1,231325026	4,18560009	2,84E-05	0,00054544	-
KIAA1024	chr15:79724857-79764642	3,31626	1,30115	-1,34978	-2,18479	5,00E-05	0,00070533	419,461011	-1,192294247	3,66367785	0,00024862	0,00292824	-
KIAA1045	chr9:34958191-34982541	10,2295	2,31409	-2,14422	-3,7629	5,00E-05	0,00070533	912,234846	-1,792438142	4,96814798	6,76E-07	2,78E-05	-
KIAA1211L	chr2:99410308-99552684	0,65506	1,7995	1,4579	1,76059	0,00015	0,00184356	130,110272	1,413194425	-5,6795531	1,35E-08	1,16E-06	-
KIAA1467	chr12:13197314-13236383	15,5892	6,88476	-1,17907	-2,28505	5,00E-05	0,00070533	1194,3733	-1,060483749	3,78955383	0,00015092	0,00197248	-
KIF21A	chr12:39687029-39837192	64,8256	25,9957	-1,31829	-2,47556	5,00E-05	0,00070533	9442,80192	-1,160436502	3,89024663	0,00010014	0,00145115	-
KIF3A	chr5:132028322-132073265	41,7713	16,6057	-1,33084	-2,64966	5,00E-05	0,00070533	4698,30435	-1,212629761	4,04634642	5,20E-05	0,00088719	-
KIF3C	chr2:26149454-26205443	34,0457	10,4596	-1,70264	-3,36313	5,00E-05	0,00070533	2951,42961	-1,460970742	4,44830016	8,66E-06	0,00021313	-
KIF5A	chr12:57943846-57978554	73,4326	18,6621	-1,97631	-3,8358	5,00E-05	0,00070533	5959,19562	-1,707140596	5,25127326	1,51E-07	8,07E-06	-
KIF5C	chr2:149632791-149883273	133,682	63,2084	-1,08062	-2,13338	5,00E-05	0,00070533	18582,0398	-1,056029487	6,79083886	1,11E-11	2,77E-09	-
KLC2	chr11:66024764-66035332	17,3971	7,32777	-1,2474	-2,34053	5,00E-05	0,00070533	940,714264	-1,056195809	4,23313379	2,30E-05	0,00045721	-
KLF2	chr19:16435650-16438339	12,8117	32,2124	1,33016	2,29506	5,00E-05	0,00070533	654,133995	1,209410217	-3,7606594	0,00016947	0,00215043	-
KLF5	chr13:73629113-73651680	1,30619	11,235	3,10456	4,27303	5,00E-05	0,00070533	537,689017	1,774287598	-3,5787553	0,00034523	0,00382184	-
KLF9	chr9:72999512-73029573	20,9256	54,1961	1,37292	2,88118	5,00E-05	0,00070533	4566,32134	1,245575978	-4,6824983	2,83E-06	8,94E-05	-
KLHDC7A	chr1:18807423-18812480	0,0384678	0,40433	3,39381	2,12934	0,00095	0,00886719	31,3499636	2,567523537	-5,8411416	5,18E-09	5,43E-07	-
KLHL1	chr13:70274724-70713885	2,5584	0,77171	-1,72911	-2,20694	5,00E-05	0,00070533	179,230318	-1,450485003	4,31618103	1,59E-05	0,00033737	-
KLHL6	chr3:183205318-183273499	1,05668	3,80532	1,84848	2,91062	5,00E-05	0,00070533	476,530024	1,625638632	-5,8342078	5,40E-09	5,58E-07	-
KLK7	chr19:51479734-51487320	2,69555	0,725789	-1,89296	-1,86183	0,00015	0,00184356	90,9643211	-1,373919657	2,98820037	0,00280626	0,018801	-
KNDC1	chr10:134973970-135039916	24,4192	10,6484	-1,19738	-2,56797	5,00E-05	0,00070533	3036,02709	-1,109964745	5,38742634	7,15E-08	4,43E-06	-
KRT16	chr17:39766030-39769079	0,0673971	3,88151	5,84779	3,64055	0,0001	0,00129637	106,167403	2,043098069	-4,0430825	5,28E-05	0,00089745	-
KRT19	chr17:39679868-39684641	0,521321	1,58154	1,60108	1,25301	0,00785	0,0491202	41,0741388	1,410612353	-3,8267611	0,00012984	0,00175804	-
KRT222	chr17:38811871-38821416	4,22724	1,32547	-1,67321	-1,90934	5,00E-05	0,00070533	155,07531	-1,168060941	2,58448983	0,00975232	0,04726141	-
KRTAP5-AS1	chr11:1593970-1620414	0,978022	0,364986	-1,42202	-1,26239	0,00605	0,0400655	40,7194897	-1,103344836	2,90475293	0,00367543	0,02301746	-
KYNU	chr2:143635194-143799885	1,46009	3,30255	1,17752	1,42126	0,0014	0,0122699	153,788647	1,118600601	-4,6770116	2,91E-06	9,11E-05	-
L1CAM	chrX:153126968-153151628	13,8779	3,29903	-2,07267	-3,71177	5,00E-05	0,00070533	1114,23396	-1,689724963	4,49655268	6,91E-06	0,00017918	Neuron
LAIR1	chr19:54865232-54882163	4,65963	12,5076	1,42452	2,52146	5,00E-05	0,00070533	777,104807	1,277872961	-4,9728578	6,60E-07	2,76E-05	-
LAMP5	chr20:9485826-9511171	14,8398	3,1828	-2,2211	-3,26826	5,00E-05	0,00070533	419,112607	-1,75103944	4,30077038	1,70E-05	0,00035766	-
LAPTM5	chr1:31205314-31230683	24,3915	88,3701	1,85718	3,76905	5,00E-05	0,00070533	3026,78402	1,640754348	-5,6176555	1,94E-08	1,52E-06	-
LAT2	chr7:73624086-73644164	4,34843	10,619	1,28808	1,99791	0,0001	0,00129637	483,771671	1,119660291	-4,252602	2,11E-05	0,00042663	-
LCP2	chr5:169675087-169724822	2,52062	5,34693	1,08493	1,70502	0,0001	0,00129637	454,663859	1,081627872	-6,0433091	1,51E-09	1,94E-07	-
LEFTY2	chr1:226124297-226129083	0,217691	0,731952	1,74947	1,32956	0,00185	0,0155409	22,0308993	1,399375989	-3,4981078	0,00046857	0,00485147	-
LEPR	chr1:65886130-66103176	15,1656	52,1677	1,78235	2,28673	5,00E-05	0,00070533	4192,77739	1,294109965	-3,0118739	0,00259641	0,01767628	-
LGALS3	chr14:55595934-55612148	34,0127	78,94	1,21468	2,34782	5,00E-05	0,00070533	1367,05605	1,071225955	-3,5471384	0,00038944	0,00421926	-
LGALS9	chr17:25958173-25976586	5,38817	14,2233	1,40039	2,26231	5,00E-05	0,00070533	442,943791	1,253651865	-4,8901659	1,01E-06	3,83E-05	-
LGI2	chr4:25000470-25032414	5,64884	1,83355	-1,62332	-2,75176	5,00E-05	0,00070533	594,747517	-1,400589822	3,96445602	7,36E-05	0,001157	-
LGI3	chr8:22004342-22014344	53,1506	23,8505	-1,15606	-2,47864	5,00E-05	0,00070533	2638,94605	-1,06071146	4,19690769	2,71E-05	0,00052231	Oligodendroc
LGR6	chr1:202163117-202288889	7,71578	1,80782	-2,09356	-3,13741	5,00E-05	0,00070533	428,891945	-1,794080783	6,00671833	1,89E-09	2,37E-07	-
LHFPL2	chr5:77781037-77944648	10,1528	23,9377	1,23741	2,6338	5,00E-05	0,00070533	2191,85799	1,166619846	-5,8378413	5,29E-09	5,48E-07	-
LHFPL4	chr3:9540044-9595486	4,84414	2,00626	-1,27174	-2,08178	5,00E-05	0,00070533	350,218093	-1,153792015	4,43411449	9,25E-06	0,00022497	-
LHX6	chr9:124964855-124991091	0,567	1,58845	1,4862	1,60852	0,0005	0,00518549	99,1437737	1,247190431	-3,3318575	0,00086268	0,00758479	-
LILRA1	chr19:55105040-55113846	1,01901	2,16983	1,09041	1,20127	0,0065	0,0424433	123,624357	1,029738386	-3,4756167	0,00050968	0,00518858	-
LILRA2	chr19:55084463-55099028	1,37828	4,57649	1,73138	2,06141	5,00E-05	0,00070533	174,293205	1,448295362	-4,4633359	8,07E-06	0,00020134	-
LILRA4	chr19:54844691-54850421	1,48583	4,95605	1,73792	1,97791	5,00E-05	0,00070533	185,519565	1,316225321	-3,2452768	0,00117337	0,00968496	-
LILRA5	chr19:54818352-54824409	0,103855	1,03223	3,31312	1,72366	0,0058	0,0387613	24,7855054	1,990666197	-4,5104016	6,47E-06	0,00017074	-
LILRB1	chr19:55128383-55148981	1,58092	4,12477	1,38355	1,86656	5,00E-05	0,00070533	258,247858	1,126542863	-3,9927296	6,53E-05	0,00105711	-
LILRB3	chr19:54720146-54746711	0,332422	2,26983	2,7715	2,45719	5,00E-05	0,00070533	91,9256655	2,39947956	-8,0368308	9,22E-16	5,94E-13	-
LILRB4	chr19:55174270-55181810	3,4605	8,77165	1,34187	1,56235	0,0006	0,00609837	537,119679	1,144552337	-3,3974647	0,00068013	0,00637645	-
LIMK1	chr7:73498106-73536855	30,8052	11,7543	-1,38998	-2,75892	5,00E-05	0,00070533	1496,72521	-1,191501614	3,76032784	0,00016969	0,00215043	-
LINC00087	chrX:134229014-134232733	8,69515	3,21893	-1,43363	-2,30261	5,00E-05	0,00070533	345,007807	-1,218327518	3,73296415	0,00018924	0,00233271	-
LINC00599	chr8:9757573-9760839	2,60763	1,00069	-1,38175	-1,66563	0,00025	0,00289273	136,912087	-1,205983573	3,44947261	0,00056168	0,00556425	-
LINC00836	chr10:25940640-26021864	4,0676	9,03328	1,15107	1,61262	0,00015	0,00184356	276,332524	1,008646566	-3,3935504	0,00068993	0,00644792	-
LINC00839	chr10:42970938-42990785	4,00032	0,69687	-2,52115	-2,82169	5,00E-05	0,00070533	151,647272	-1,858488927	4,44083218	8,96E-06	0,0002192	-
LINC00856	chr10:80008381-80012402	2,31851	0,901755	-1,36239	-1,4833	0,00125	0,0111709	114,280211	-1,198008528	4,07577201	4,59E-05	0,00080205	-
LINC00961	chr9:35909479-35911617	0,703181	3,15535	2,16583	2,06635	5,00E-05	0,00070533	75,6867543	1,647758526	-4,0729565	4,64E-05	0,00080902	-

LINC01272	chr20:48884022-48896332	0,0623828	0,448007	2,8443	1,46097	0,0052	0,0356302	15,2944042	2,328200746	-5,9984747	1,99E-09	2,46E-07	-
LINC01314	chr15:80487820-80544603	3,26044	1,30475	-1,32129	-2,24009	5,00E-05	0,00070533	406,026238	-1,275891133	6,39687322	1,59E-10	2,73E-08	-
LINGO4	chr1:151772764-151777882	3,28088	0,232278	-3,82016	-3,22307	5,00E-05	0,00070533	93,4871431	-2,614715256	5,64699466	1,63E-08	1,33E-06	-
LMNB1	chr5:126112314-126172712	1,18751	3,29948	1,4743	1,9352	5,00E-05	0,00070533	184,448117	1,406568803	-5,5667535	2,60E-08	1,93E-06	-
LOC1002872	chr18:48918411-49088839	5,61904	2,50176	-1,16738	-1,69833	0,0002	0,0023747	275,287643	-1,092829339	6,24149781	4,33E-10	6,53E-08	-
LOC1005067	chr7:35756046-35759322	25,9811	9,78019	-1,40953	-2,43575	5,00E-05	0,00070533	541,01172	-1,358755864	7,47952394	7,46E-14	3,02E-11	-
LOC1019276	chr2:200472790-200523855	3,0222	1,30508	-1,21145	-1,46643	0,00125	0,0111709	210,922235	-1,199880652	4,31272156	1,61E-05	0,00034024	-
LOC1019279	chr19:42041859-42043266	0,215287	2,07341	3,26767	1,82404	0,0024	0,0191464	49,8049063	2,21022402	-6,384516	1,72E-10	2,93E-08	-
LOC1019281	chr2:86116402-86119375	9,10821	3,82565	-1,25146	-1,64963	0,00025	0,00289273	219,871413	-1,218613553	5,26887071	1,37E-07	7,51E-06	-
LOC1019293	chr14:101433988-101452987	8,08518	1,66567	-2,27918	-2,22701	5,00E-05	0,00070533	207,148435	-1,612466026	4,70349885	2,56E-06	8,19E-05	-
LOC440040	chr11:49580079-49831969	0,848136	0,211809	-2,00153	-1,56788	0,0005	0,00518549	32,4830401	-1,312968395	3,07205919	0,00212588	0,01523175	-
LOC441666	chr10:42827313-42863493	2,70751	1,16686	-1,21433	-1,90786	5,00E-05	0,00070533	341,180422	-1,150021092	3,75870219	0,0001708	0,00215956	-
LOC54944	chr22:31742744-31747141	3,29745	1,52225	-1,11514	-1,7004	0,00015	0,00184356	235,749228	-1,075280168	6,50838611	7,60E-11	1,46E-08	-
LOC645638	chr17:58160926-58165828	0,522534	3,20738	2,6178	1,67178	0,0033	0,0247184	49,0441093	1,57173757	-3,1085232	0,00188025	0,01387659	-
LOC729737	chr1:134772-140566	0,35829	0,862597	1,26756	1,37407	0,00295	0,0226522	75,3097775	1,128073566	-4,1443177	3,41E-05	0,00063532	-
LOX	chr5:121398889-121414055	1,96979	5,35068	1,44168	2,21756	5,00E-05	0,00070533	432,416877	1,124510935	-3,0319087	0,00243013	0,01691253	-
LPAR3	chr1:85279085-85358896	0,35876	1,62738	2,18146	1,7125	0,0004	0,00431348	41,3067923	1,420712849	-2,9735441	0,00294382	0,01945732	-
LPPR3	chr19:812487-821952	1,50712	0,423198	-1,83239	-1,54718	0,00135	0,0119	39,3423589	-1,226326341	2,91611615	0,00354419	0,02242694	-
LPPR4	chr1:99729847-99775138	5,87443	2,6922	-1,12566	-1,9854	5,00E-05	0,00070533	546,14367	-1,060758331	4,39688216	1,10E-05	0,00025348	-
LRFN2	chr6:40359372-40555126	1,44942	0,635059	-1,19051	-1,28084	0,00465	0,0325248	70,1351671	-1,05586658	3,39800008	0,0006788	0,00636687	-
LRG1	chr19:4537226-4540036	0,18762	1,01056	2,42927	1,63025	0,0036	0,0264814	28,3745288	1,691965024	-3,8426575	0,00012171	0,00167907	-
LRRC25	chr19:18501953-18508415	0,902268	3,49586	1,95402	2,22386	0,0001	0,00129637	128,072474	1,850318577	-6,7178082	1,84E-11	4,48E-09	-
LRRC32	chr11:76368567-76381791	5,43512	13,8851	1,35315	2,36345	5,00E-05	0,00070533	1000,88062	1,032999386	-2,6115533	0,00901319	0,04455851	-
LRRC38	chr1:13801444-13840242	0,200815	1,10903	2,46536	1,84945	5,00E-05	0,00070533	31,6300484	1,630693705	-3,4704487	0,00051959	0,00526608	-
LRRC73	chr6:43474702-43478081	2,56065	0,671784	-1,93044	-1,63354	0,0011	0,0100332	47,2674961	-1,387963503	3,84578826	0,00012017	0,00166641	-
LRRN4CL	chr11:62453873-62457371	1,55439	5,43498	1,80592	2,28678	5,00E-05	0,00070533	198,646561	1,375624032	-3,0984841	0,00194513	0,01424338	-
LSP1	chr11:1874199-1913493	4,23086	10,5046	1,31201	2,02092	5,00E-05	0,00070533	383,646996	1,137552063	-3,2430414	0,00118261	0,00975346	-
LTBP2	chr14:74964885-75079034	6,70966	20,6807	1,62398	3,10407	5,00E-05	0,00070533	3164,71034	1,338839441	-3,4109907	0,00064727	0,00617498	-
LY86	chr6:6346697-6655216	6,32912	17,1095	1,43473	1,9414	5,00E-05	0,00070533	262,033563	1,195390407	-3,2789361	0,00104199	0,00877419	-
LY96	chr8:74903563-74941307	5,06171	12,1404	1,26212	1,49325	0,00125	0,0111709	120,695964	1,234581323	-5,430091	5,63E-08	3,60E-06	-
LYN	chr8:56792385-56925006	3,36205	8,51477	1,34063	2,42137	5,00E-05	0,00070533	676,220828	1,18881894	-4,6607613	3,15E-06	9,67E-05	-
LYPD5	chr19:44300078-44324808	4,46985	1,84146	-1,27937	-1,78081	5,00E-05	0,00070533	200,102093	-1,105736349	3,82193684	0,00013241	0,00178928	-
LYZ	chr12:69742133-69748013	5,96822	41,4071	2,79451	4,55857	5,00E-05	0,00070533	846,678589	2,426868061	-7,2667543	3,68E-13	1,38E-10	-
MAFB	chr20:39314487-39317880	3,19447	19,2064	2,58794	4,51836	5,00E-05	0,00070533	844,945041	2,43264509	-10,455967	1,38E-25	4,73E-22	-
MAGEE1	chrX:75648045-75651746	9,76647	4,04195	-1,27279	-2,24629	5,00E-05	0,00070533	541,110734	-1,142549167	3,68206966	0,00023135	0,00275628	-
MAL2	chr8:120220609-120257914	0,578852	2,28426	1,98046	1,89082	0,00025	0,00289273	83,4402022	1,463377994	-3,3074603	0,00094146	0,00809123	Neuron
MALL	chr2:110841446-110874143	0,156113	0,504162	1,6913	1,22581	0,00465	0,0325248	23,6614938	1,408142581	-3,4878475	0,00048693	0,00500633	-
MAN1A1	chr6:119498365-119670931	8,43802	20,2519	1,26308	2,60356	5,00E-05	0,00070533	1653,40937	1,200113391	-5,5664463	2,60E-08	1,93E-06	-
MANEAL	chr1:38259773-38267278	10,6868	3,34714	-1,67483	-2,66002	5,00E-05	0,00070533	408,488555	-1,415861529	3,8944655	9,84E-05	0,00143304	-
MAP1A	chr15:43663256-43910998	139,174	58,2146	-1,25743	-1,96635	5,00E-05	0,00070533	31680,4186	-1,166451389	4,69082296	2,72E-06	8,68E-05	-
MAP2	chr2:210288770-210598834	69,6617	20,3256	-1,77707	-2,79125	5,00E-05	0,00070533	12258,2615	-1,565963858	3,92242563	8,77E-05	0,00131366	-
MAP3K6	chr1:27681669-27693337	3,63683	7,80828	1,10232	1,97601	5,00E-05	0,00070533	674,092922	1,082628093	-5,3118001	1,09E-07	6,31E-06	-
MAP3K8	chr10:30722949-30750762	3,18493	7,11646	1,1599	1,83379	0,00015	0,00184356	503,562879	1,071482267	-4,2507209	2,13E-05	0,00042897	-
MAP3K9	chr14:71189242-71275888	7,5746	1,69487	-2,16	-3,69618	5,00E-05	0,00070533	1164,21861	-1,748398089	4,50817846	6,54E-06	0,0001721	-
MAP7D2	chrX:20024830-20135114	24,0559	2,19947	-3,45116	-5,14318	5,00E-05	0,00070533	1487,7531	-2,160980304	4,45302473	8,47E-06	0,00020924	-
MAP9	chr4:156263811-156298122	19,8086	8,72549	-1,18282	-2,37122	5,00E-05	0,00070533	2778,13559	-1,065304145	3,48287503	0,00049606	0,00508757	-
MAPK8IP2	chr22:51039113-51049979	31,8722	11,7136	-1,44411	-2,98	5,00E-05	0,00070533	1403,38108	-1,258654071	4,79343327	1,64E-06	5,75E-05	-
MAPRE3	chr2:27193524-27250087	33,1768	12,7411	-1,38068	-2,61045	5,00E-05	0,00070533	1024,51817	-1,186421725	3,84648643	0,00011982	0,00166358	-
MARCH11	chr5:16067473-16179897	0,552128	0,105844	-2,38306	-1,32721	0,0072	0,0459951	9,29707168	-1,383752115	2,86120859	0,00422029	0,02550095	-
MAST1	chr19:12949258-12985766	15,4922	2,77776	-2,47955	-4,34948	5,00E-05	0,00070533	1010,58146	-1,972985119	5,32369529	1,02E-07	6,05E-06	-
MCF2	chrX:138663929-138790381	5,14999	1,92138	-1,42243	-2,24545	5,00E-05	0,00070533	420,603204	-1,272252151	4,82305078	1,41E-06	5,09E-05	-
MCHR1	chr22:41075181-41078818	2,43416	0,655069	-1,8937	-1,89608	0,00015	0,00184356	79,9698026	-1,399431907	3,28576766	0,00101705	0,00860467	-
MDH1	chr2:63348534-63834330	137,589	57,7904	-1,25147	-2,33234	5,00E-05	0,00070533	3621,83651	-1,07342167	3,23942729	0,0011977	0,00986215	-
ME1	chr6:83920109-84140938	27,4279	12,0004	-1,19256	-2,46485	5,00E-05	0,00070533	1690,30161	-1,114693974	4,49551161	6,94E-06	0,00017947	-
MEFV	chr16:3292027-3306627	0,130963	0,658931	2,33097	1,84887	0,0001	0,00129637	42,2193173	1,831597861	-5,5046016	3,70E-08	2,55E-06	-
MEG3	chr14:101292444-101327360	48,2681	22,6302	-1,09282	-1,57447	0,00025	0,00289273	3034,99927	-1,134320737	3,5050588	0,00045651	0,00476335	-
MERTK	chr2:112656190-112786945	6,25982	13,9142	1,15237	2,24848	5,00E-05	0,00070533	1190,51598	1,117307423	-5,8809864	4,08E-09	4,50E-07	Astrocyte
MEST	chr7:130126015-130371406	23,12	8,15472	-1,50344	-2,22325	5,00E-05	0,00070533	922,308149	-1,22994598	3,34100741	0,00083475	0,00744063	-
MFAP5	chr12:8798539-8815433	1,21875	8,63584	2,82493	3,81503	5,00E-05	0,00070533	391,270882	1,989157482	-4,4027415	1,07E-05	0,00024811	-
MFSD4	chr1:205538111-205572046	7,04742	2,29058	-1,62138	-2,59124	5,00E-05	0,00070533	406,044576	-1,436046215	4,73071793	2,24E-06	7,38E-05	-
MGP	chr12:15034114-15038853	32,5121	145,031	2,15731	3,82064	5,00E-05	0,00070533	3090,00191	1,419956976	-2,9954511	0,00274039	0,01844358	-
MIAT	chr22:27053445-27176856	11,6332	4,30273	-1,43493	-2,73075	5,00E-05	0,00070533	1795,06224	-1,251236502	3,81447815	0,00013647	0,001831	-
MIR124-2HG	chr8:65285774-65300061	2,64659	0,761043	-1,79808	-1,11174	0,0058	0,0387613	118,723779	-1,177932568	2,70819697	0,00676499	0,03596895	-
MKI67	chr10:129894924-129924468	0,0575056	0,393134	2,77325	2,71989	5,00E-05	0,00070533	124,738015	2,043224127	-4,4856997	7,27E-06	0,00018478	-
MLKL	chr16:74705752-74734789	2,05123	4,48212	1,12769	1,56568	0,0003	0,00336918	301,262941	1,061174246	-3,191266	0,00141651	0,01119325	-
MLLT11	chr1:151032150-151040973	37,4803	9,21641	-2,02386	-3,56471	5,00E-05	0,00070533	1074,20105	-1,669966724	4,36398675	1,28E-05	0,0002846	-
MMP15	chr16:58059281-58080804	7,69537	2,61652	-1,55634	-2,55968	5,00E-05	0,00070533	423,106233	-1,322566727	4,56213018	5,06E-06	0,00013988	-
MNDA	chr1:158801167-158819270	3,04097	6,6541	1,12971	1,73706	0,0001	0,00129637	324,276837	1,025809484	-4,4354855	9,19E-06	0,00022381	-
MNX1	chr7:156797546-156803347	1,5343	0,138455	-3,4701	-2,11808	0,0008	0,00771573	26,4168442	-2,069356113	4,3220091	1,55E-05	0,00033062	Motor Neuro
MPP3	chr17:41878166-41910547	5,35094	1,3388	-1,99885	-2,56331	5,00E-05	0,00070533	298,730083	-1,435456274	4,20624544	2,60E-05	0,00050403	-
MPZL2	chr11:118124130-118135251	2,52162	21,8214	3,11332	4,56267	5,00E-05	0,00070533	798,140752	2,39775872	-5,8725004	4,29E-09	4,66E-07	-
MR1	chr1:181002560-181031074	2,0872	4,99446	1,25876	2,37941	5,00E-05	0,00070533	688,977338	1,20539893	-7,0362882	1,97E-12	5,90E-10	-
MRAP2	chr6:84743419-84800605	6,9055	1,9924	-1,79324	-2,37753	5,00E-05	0,00070533	235,1641	-1,360422364	3,24574189	0,00117145	0,00967302	-
MRGPRE	chr11:3249040-3253616	3,11244	1,08121	-1,5254	-1,39726	0,0016	0,0137713	52,9636471	-1,087420107	2,70790553	0,00677093	0,03599127	-
MS4A14	chr11:60163486-60185228	0,459476	1,61323	1,81189	2,14808	5,00E-05	0,00070533	139,78609	1,625158146	-5,123792	2,99E-07	1,43E-05	-
MS4A4A	chr11:60048013-60076445	2,722	14,0013	2,36282	3,33031	5,00E-05	0,00070533	385,349214	2,002142454	-6,676381	2,45E-11	5,68E-09	-
MS4A6A	chr11:59939079-59952139	6,83437	27,2864	1,9973	3,43361	5,00E-05	0,00070533	997,985812	1,705776376	-5,8689049	4,39E-09	4,74E-07	-
MS4A7	chr11:60145957-60163426	3,45074	17,4757	2,34037	3,83105	5,00E-05	0,00070533	955,168845	1,874291572	-5,3995031	6,68E-08	4,20E-06	-
MSMO1	chr4:166248817-166264314	104,545	33,7953	-1,62923	-3,5286	5,00E-05	0,00070533	3120,9352	-1,554009934	7,68646248	1,51E-14	7,43E-12	-
MSR1	chr8:15965386-16050300	6,55947	31,0949	2,24503	4,21432	5,00E-05	0,00070533	1843,32839	1,951297518	-6,2518673	4,06E-10	6,20E-08	-
MST4	chrX:131157244-131209971	0,819074	1,89148	1,20745	1,40381	0,0034	0,0252982	112,549293	1,240187059	-5,1941145	2,06E-07	1,04E-05	-
MT1L	chr16:56651372-56652730	4,41791	12,447	1,49436	1,45877	0,0021	0,0172529	68,387086	1,117288459	-3,1266527	0,00176809	0,01325744	-
MT1X	chr16:56716381-56718108	69,8918	172,412	1,30267	2,37263	5,00E-05	0,00070533	919,321964	1,295439739	-3,9455476	7,96E-05	0,00122241	-
MT2A	chr16:56642477-56643409	139,634	311,301	1,15666	2,30578	5,00E-05	0,00070533	1550,24601	1,064389567	-3,9247098	8,68E-05	0,00130505	-
MTUS2	chr13:29598747-30080084	1,25679	0,446584	-1,49274	-1,62749	0,00055	0,00564481	145,100603	-1,189405223	3,42489098	0,00061505	0,00595283	-
MVK	chr12:110011499-110035071	17,0308	7,20078	-1,24192	-2,28704	5,00E-05	0,00070533	604,807586	-1,136726537	6,46989822	9,81E-11	1,81E-08	-
MXRA8	chr1:1288068-1298921	14,1363	32,5964	1,20531	2,17487	5,00E-05	0,00070533	1173,10824	1,064125983	-2,7290711	0,0063513	0,03425486	-
MYADML2	chr17:79897520-79905109	1,06119	0,235945	-2,16917	-1,73324	0,0004	0,00431348	33,5731265	-1,421478027	3,07522376	0,00210345	0,01509726	-
MYD88	chr3:38179968-38184512	2,65735	5,8733	1,14419	1,81656	5,00E-05	0,00070533	315,625447	1,053739751	-7,5043899	6,17E-14	2,55E-11	-
MYO1G	chr7:45002259-45018704	0,365265	1,66534	2,1888	2,20065	5,00E-05	0,00070533	122,918638	1,888980427	-6,3114575	2,76E-10	4,49E-08	-
MYOC	chr1:171604556-171621773	1,08787	9,79322	3,17028	3,62486	5,00E-05	0,00070533	373,9205	1,580454038	-3,1254606	0,00177527	0,01329197	-
MYOF	chr10:95066185-95242074	7,42102	17,0135	1,19699	2,51795	5,00E-05	0,00070533	3064,38696	1,142582381	-4,6807712	2,86E-06	8,98E-05	-
MYT1L	chr2:1792884-2335045	2,87538	0,762737	-1,91449	-2,81719	5,00E-05	0,00070533	376,679643	-1,437508973	3,75854815	0,0001709	0,00215956	Neuron
NABP1	chr2:192542797-192553248	3,05151	6,81059	1,15826	1,89074	5,00E-05	0,00070533	493,905406	1,125355602	-6,6908831	2,22E-11	5,20E-09	-
NAP1L2	chrX:72432136-72434710	18,966	8,71174	-1,12238	-2,092	5,00E-05	0,00070533	818,877643	-1,019037383	3,4185801	0,00062949	0,00606985	-
NAPB	chr20:23355155-23402156	45,9093	18,5475	-1,30756	-2,52652	5,00E-05	0,00070533	2898,41023	-1,108501255	3,36065284	0,00077759	0,00707731	Neuron
NBEAP1	chr15:20874796-20961480	5,78906	1,44469	-2,00257	-1,524	0,0007	0,00690182	40,738146	-1,47837666	4,02244665	5,76E-05	0,00096236	-
NCAN	chr19:19322781-19363061	25,8857	8,62961	-1,58479	-3,06748	5,00E-05	0,00070533	2753,93543	-1,32855886	3,51801333	0,00043479	0,00459626	-
NCDN	chr1:36023392-36032380	52,1191	19,545	-1,41534	-2,89841	5,00E-05	0,00070533	2545,47099	-1,203140778	3,7534263	0,00017443	0,00219612	-
NCF2	chr1:183524696-183560056	2,30004	8,44113	1,87578	2,86717	5,00E-05	0,00070533	459,28297	1,692205203	-5,318417	1,05E-07	6,17E-06	-
NCF4	chr22:37257029-37274059	1,88742	5,32918	1,4975	1,85339	0,0001	0,00129637	156,432219	1,36051771	-4,6682748	3,04E-06	9,43E-05	-
NCKAP1L	chr12:54891494-54936899	4,66805	11,3563	1,2826	2,3942	5,00E-05	0,00070533	1099,47972	1,104926137	-3,9215904	8,80E-05	0,00131726	-
NCS1	chr9:132934856-132999583	49,3602	19,2683	-1,35712	-2,90289	5,00E-05	0,00070533	3342,51695	-1,269509879	5,13081318	2,88E-07	1,39E-05	-
NDRG4	chr16:58497548-58547523	157,29	53,9039	-1,54497	-2,98514	5,00E-05	0,00070533	7795,13424	-1,380576764	4,39997067	1,08E-05	0,00025073	-
NEDD9	chr6:11183530-11382581	10,2937	22,6424	1,13727	1,64329	0,0001	0,00129637	1653,62925	1,245881841	-3,8524408	0,00011695	0,00162943	-
NEFH	chr22:29876180-29887277	483,255	31,4984	-3,93943	-4,71487	5,00E-05	0,00070533	27791,7724	-2,285979041	4,61990274	3,84E-06	0,00011285	-
NEFL	chr8:24808468-24814383	610,19	46,9682	-3,6995	-4,16493	5,00E-05	0,00070533	29548,4449	-2,138659314	4,29536583	1,74E-05	0,00036478	Neuron
NEFM	chr8:24771273-24776606	499,888	33,5911	-3,89545	-3,75355	5,00E-05	0,00070533	27910,6277	-2,079116606	4,13534601	3,54E-05	0,00065709	Neuron
NELL2	chr12:44902057-45307711	15,544	4,8381	-1,68308	-2,88384	5,00E-05	0,00070533	892,194839	-1,379565038	3,78849714	0,00015156	0,00197816	-
NETO1	chr18:70409548-70534810	2,33434	0,757825	-1,62308	-1,92649	5,00E-05	0,00070533	218,454505	-1,31069291	3,4816631	0,00049831	0,00510558	-
NETO2	chr16:47115430-47177936	4,24966	1,73239	-1,29458	-1,91534	5,00E-05	0,00070533	256,926405	-1,155117652	5,01541716	5,29E-07	2,30E-05	-
NEURL1	chr10:105253734-105352309	3,70524	1,37899	-1,42596	-1,95889	5,00E-05	0,00070533	196,992552	-1,236809374	3,81225507	0,00013771	0,00184176	-
NEXN	chr1:78347032-78409578	0,929917	2,07656	1,15902	1,55902	0,0006	0,00609837	211,382943	1,047735289	-3,5059862	0,00045492	0,00475304	-
NINJ2	chr12:673461-772755	17,6456	8,59795	-1,03724	-1,67019	0,00045	0,00473416	331,711097	-1,012900139	5,39509849	6,85E-08	4,27E-06	-
NIPA1	chr15:23043278-23086843	55,666	26,9708	-1,0454	-2,2931	5,00E-05	0,00070533	5864,68353	-1,042157892	5,54546718	2,93E-08	2,09E-06	-
NLRC4	chr2:32449517-32490812	0,358332	1,37242	1,93735	1,91313	0,0001	0,00129637	102,94836	1,804496594	-5,6229866	1,88E-08	1,51E-06	-
NMNAT2	chr1:183217371-183387634	8,09664	3,15022	-1,36187	-2,39208	5,00E-05	0,00070533	834,434108	-1,121890223	2,9476285	0,00320222	0,02072052	-
NNMT	chr11:114166534-114183238	7,42235	39,3283	2,40562	3,71798	5,00E-05	0,00070533	949,669386	1,513216608	-3,1281044	0,00175938	0,01320173	-
NOS3	chr7:150688143-150721586	3,013	9,93867	1,72185	2,65611	5,00E-05	0,00070533	663,056133	1,325810566	-3,3586689	0,00078319	0,0071069	-
NOV	chr8:120428551-120436678	4,36978	29,6281	2,76133	4,18634	5,00E-05	0,00070533	1219,24781	1,549060329	-3,1092493	0,00187563	0,01384748	Neuron
NPAS1	chr19:47524142-47549017	1,1557	0,238004	-2,27971	-1,73009	0,00015	0,00184356	30,3613184	-1,678699121	4,91564739	8,85E-07	3,41E-05	-

NPM2	chr8:21881620-21894408	4,60123	1,39664	-1,72006	-1,8261	0,0003	0,00336918	128,891642	-1,209372982	3,41137612	0,00064636	0,00617166	-
NPNT	chr4:106816596-106892828	2,1016	7,89443	1,90934	2,88738	5,00E-05	0,00070533	593,590721	1,389186456	-3,1282864	0,00175829	0,01319835	-
NPR1	chr1:153651163-153666468	0,656876	1,88999	1,52469	1,83954	5,00E-05	0,00070533	142,617723	1,311015441	-3,982331	6,82E-05	0,00109077	-
NPR3	chr5:32710742-32791830	1,82197	0,520737	-1,80687	-2,26915	5,00E-05	0,00070533	195,433782	-1,471120153	4,12905689	3,64E-05	0,00066991	-
NPTX1	chr17:78440632-78450404	3,20432	1,03797	-1,62625	-2,37505	5,00E-05	0,00070533	246,976354	-1,393362503	4,27550775	1,91E-05	0,00039273	Neuron
NPTX2	chr7:98246596-98259181	0,726601	2,52966	1,79971	1,98717	0,0001	0,00129637	100,406209	1,477288457	-3,6353088	0,00027765	0,00319535	-
NRG1	chr8:31497267-32622558	19,2259	2,03666	-3,23877	-4,21024	5,00E-05	0,00070533	635,198048	-2,350264949	5,31515562	1,07E-07	6,25E-06	-
NRG4	chr15:76234327-76304785	2,20214	0,655633	-1,74794	-1,72747	5,00E-05	0,00070533	140,524015	-1,249989819	2,91502649	0,00355658	0,02247094	-
NRIP2	chr12:2934513-2944221	7,41874	3,03001	-1,29185	-2,10239	5,00E-05	0,00070533	380,834784	-1,097135097	3,45160733	0,00055726	0,00554004	-
NRIP3	chr11:9002122-9025596	20,4104	3,42969	-2,57315	-4,0963	5,00E-05	0,00070533	1017,97916	-1,915106728	4,33787984	1,44E-05	0,00031414	-
NRSN1	chr6:24126413-24147757	8,06701	1,99258	-2,0174	-2,74295	5,00E-05	0,00070533	265,660835	-1,584694879	3,9373855	8,24E-05	0,00125539	-
NSF	chr17_ctg5_hap1:195720-462618	26,8278	8,60344	-1,64074	-3,09326	5,00E-05	0,00070533	1943,98236	-1,378688985	3,77219873	0,00016182	0,00207653	-
NSG1	chr4:4387982-4543775	24,2943	6,45974	-1,91107	-2,43621	5,00E-05	0,00070533	800,869345	-1,559350321	4,01785622	5,87E-05	0,00097812	-
NXPH1	chr7:8473584-8792593	5,29577	1,7836	-1,57005	-2,209	5,00E-05	0,00070533	260,167229	-1,262698621	3,26040418	0,00111254	0,00924946	-
NXPH4	chr12:57610577-57620232	1,20331	0,361131	-1,73641	-1,2891	0,00325	0,0244419	21,4767656	-1,267264928	2,993324	0,00275957	0,01853627	-
NYAP2	chr2:226265601-226518734	1,75189	0,290554	-2,59204	-2,60198	5,00E-05	0,00070533	117,508512	-1,982993657	4,68378323	2,82E-06	8,90E-05	-
OGDHL	chr10:50942686-50970425	15,6052	2,94768	-2,40437	-3,97778	5,00E-05	0,00070533	878,895043	-1,929938329	5,02142925	5,13E-07	2,25E-05	-
OLFM2	chr19:9964393-10047070	45,8624	18,5032	-1,30954	-2,63154	5,00E-05	0,00070533	1160,77559	-1,216116716	6,5592483	5,41E-11	1,09E-08	-
OLFM3	chr1:102268122-102462790	3,0747	0,530248	-2,53571	-2,7163	5,00E-05	0,00070533	173,627274	-1,720586216	3,83718458	0,00012445	0,00170637	-
ONECUT2	chr18:55102916-55158530	0,442911	0,124987	-1,82523	-2,00664	5,00E-05	0,00070533	108,313858	-1,349363497	3,03862263	0,00237662	0,0166412	-
OPRK1	chr8:54138275-54164257	1,07901	0,156173	-2,7885	-2,32345	5,00E-05	0,00070533	66,8872685	-1,932313178	4,1827963	2,88E-05	0,00054965	-
OR7A5	chr19:14937138-14939276	0,205823	1,45805	2,82457	1,88008	0,00115	0,010427	39,9868941	1,733028491	-3,6206637	0,00029385	0,00333896	-
OSBPL11	chr3:125247701-125314381	9,66751	26,3827	1,44837	3,0186	5,00E-05	0,00070533	2490,31107	1,38216854	-5,5583188	2,72E-08	1,98E-06	-
OSCAR	chr19:54597932-54605994	0,565223	2,44251	2,11147	1,84012	0,00045	0,00473416	61,3697854	1,669558035	-5,0833927	3,71E-07	1,71E-05	-
OSMR	chr5:38845959-38935743	6,71448	27,2332	2,02002	3,10091	5,00E-05	0,00070533	2149,46338	1,830016546	-6,0121213	1,83E-09	2,30E-07	-
OTOA	chr16:21689834-21772050	0,227825	0,595386	1,3859	1,22414	0,0029	0,0223705	50,6338263	1,14873311	-2,896877	0,00376898	0,02341849	-
OTOF	chr2:26680070-26781566	1,30936	0,260156	-2,33142	-2,52221	5,00E-05	0,00070533	129,330521	-1,538914148	4,57004434	4,88E-06	0,00013579	-
P2RX1	chr17:3799884-3819960	0,360098	0,950983	1,40103	1,32277	0,0028	0,0217288	64,8035313	1,198351832	-3,9910304	6,58E-05	0,00106305	-
PACSIN1	chr6:34433837-34503000	17,4285	4,4457	-1,97097	-3,53574	5,00E-05	0,00070533	1102,45725	-1,52976511	3,74900994	0,00017753	0,002227	-
PAK3	chrX:110187512-110464173	11,6708	3,99537	-1,5465	-2,61425	5,00E-05	0,00070533	646,912475	-1,250839606	3,94357393	8,03E-05	0,00122937	-
PALM3	chr19:14164178-14169971	2,21838	0,674745	-1,71709	-1,90416	0,0001	0,00129637	87,9253204	-1,310540013	3,40507219	0,00066147	0,00626243	-
PANX2	chr22:50609159-50618724	10,0878	2,53222	-1,99414	-3,10412	5,00E-05	0,00070533	367,00262	-1,712211786	5,03926599	4,67E-07	2,08E-05	-
PAPL	chr19:39574944-39602128	1,7743	0,552159	-1,68409	-1,81749	0,0001	0,00129637	85,6739076	-1,459399648	4,9616631	6,99E-07	2,84E-05	-
PAQR5	chr15:69591293-69699976	1,49045	4,13674	1,47274	2,32071	5,00E-05	0,00070533	381,490549	1,296002532	-4,1745434	2,99E-05	0,00056682	-
PAQR9	chr3:142680073-142682178	2,8321	0,438089	-2,69258	-2,32446	5,00E-05	0,00070533	69,2496035	-1,841233137	3,87391327	0,0001071	0,00152627	-
PARP15	chr3:122296448-122355536	0,269385	1,14387	2,08618	1,86196	0,0004	0,00431348	68,6010719	1,825114876	-5,4969058	3,87E-08	2,66E-06	-
PARVG	chr22:44568835-44604349	1,89409	5,39688	1,51062	2,2678	5,00E-05	0,00070533	472,863095	1,330743901	-5,5622156	2,66E-08	1,95E-06	-
PCBP3	chr21:47269874-47362368	7,27796	2,01304	-1,85416	-2,45109	5,00E-05	0,00070533	260,583995	-1,490546289	4,30031717	1,71E-05	0,00035766	-
PCLO	chr7:82383320-82792197	6,01406	2,59562	-1,21226	-2,4192	5,00E-05	0,00070533	2646,47865	-1,147891615	3,73016088	0,00019136	0,0023532	-
PCOLCE2	chr3:142536701-142608045	0,862663	6,90276	3,0003	3,30473	5,00E-05	0,00070533	204,774762	1,9607768	-4,1273754	3,67E-05	0,00067363	-
PCP4	chr21:41239346-41301322	13,1499	5,50757	-1,25556	-1,53334	0,0009	0,00846161	139,017942	-1,084299984	3,13244196	0,00173359	0,01304584	-
PCP4L1	chr1:161228516-161255240	12,7916	2,21691	-2,52858	-3,39962	5,00E-05	0,00070533	264,478722	-2,00428244	5,00675208	5,54E-07	2,39E-05	-
PCSK2	chr20:17206751-17465222	8,317	1,59086	-2,38626	-3,70044	5,00E-05	0,00070533	555,677374	-1,947414831	5,25321446	1,49E-07	8,03E-06	Neuron
PCYT1B	chrX:24576203-24690979	7,75228	3,60411	-1,10498	-2,12313	5,00E-05	0,00070533	818,175978	-1,058767667	5,82031789	5,87E-09	5,88E-07	-
PDK4	chr7:95212808-95225925	20,7302	74,0911	1,83756	3,91632	5,00E-05	0,00070533	4883,99159	1,709508411	-7,9204042	2,37E-15	1,32E-12	Astrocyte
PDLIM1	chr10:96997324-97050905	6,13536	16,0835	1,39037	2,20514	5,00E-05	0,00070533	466,631188	1,138388703	-3,1348497	0,00171942	0,01297742	-
PDZRN4	chr12:41582249-41968392	3,48995	7,082	1,02095	1,86799	5,00E-05	0,00070533	692,57716	1,044573505	-4,7191425	2,37E-06	7,71E-05	-
PECAM1	chr17:62396776-62407083	4,98619	17,318	1,79627	3,37974	5,00E-05	0,00070533	1364,3227	1,558824134	-5,0471226	4,49E-07	2,02E-05	-
PEG10	chr7:94285636-94299006	34,8696	14,1179	-1,30445	-2,68317	5,00E-05	0,00070533	4091,66409	-1,193029358	3,97409176	7,06E-05	0,00112141	-
PEX5L	chr3:179512746-179754841	56,6387	25,0938	-1,17445	-2,4574	5,00E-05	0,00070533	9906,60753	-1,13246996	6,59336723	4,30E-11	8,87E-09	-
PHACTR1	chr6:12717036-13295818	11,5844	4,24324	-1,44894	-2,57451	5,00E-05	0,00070533	746,097717	-1,261295039	5,12285189	3,01E-07	1,44E-05	-
PHLDA2	chr11:2949502-2950650	3,51884	1,05849	-1,73309	-1,35169	0,00615	0,0406156	30,3933015	-1,220867166	2,92916886	0,0033987	0,02173292	-
PHLDB2	chr3:111393522-111695364	4,61847	10,969	1,24794	2,35287	5,00E-05	0,00070533	1560,40049	1,012911359	-2,7109835	0,0067084	0,03573251	-
PHYHIP	chr8:22077215-22089851	22,4924	9,34142	-1,26772	-2,4664	5,00E-05	0,00070533	1117,15784	-1,089663192	3,44776359	0,00056525	0,00559152	-
PIK3AP1	chr10:98353068-98480279	3,16692	7,99551	1,33611	2,46116	5,00E-05	0,00070533	793,236907	1,224002661	-4,7833383	1,72E-06	6,01E-05	-
PIK3CG	chr7:106505722-106549423	1,78591	4,33495	1,27936	2,28991	5,00E-05	0,00070533	602,220451	1,156179229	-3,9836151	6,79E-05	0,00108826	-
PIK3R5	chr17:8782232-8869029	1,27191	3,61564	1,50725	2,27171	5,00E-05	0,00070533	365,775199	1,380343169	-5,790887	7,00E-09	6,75E-07	-
PKP2	chr12:32943679-33049780	2,86593	8,52996	1,57354	2,66624	5,00E-05	0,00070533	720,859693	1,213646919	-2,8863312	0,00389762	0,02403686	-
PLA1A	chr3:119316694-119348658	3,32408	12,7027	1,93411	2,85519	5,00E-05	0,00070533	398,780458	1,452248495	-3,8752068	0,00010653	0,00152029	-
PLA2G5	chr1:20396700-20418394	3,37327	1,05281	-1,6799	-1,87882	0,00015	0,00184356	107,333144	-1,476971089	8,58944139	8,74E-18	1,00E-14	-
PLA2G7	chr6:46655611-46703430	1,51625	6,18359	2,02794	1,53552	0,0039	0,0283163	221,975673	1,557128999	-3,9491633	7,84E-05	0,00120974	Astrocyte
PLAC8	chr4:84011210-84035911	0,491329	2,04696	2,05872	1,6479	0,0003	0,00336918	40,5044769	1,523990275	-3,6428372	0,00026965	0,00312948	-
PLB1	chr2:28718937-28866653	0,418142	0,850691	1,02464	1,20669	0,0061	0,0403171	173,069016	1,020207207	-4,094923	4,22E-05	0,00075059	-
PLD5	chr1:242251688-242687998	4,16649	1,42176	-1,55115	-2,17352	5,00E-05	0,00070533	226,261034	-1,396523586	6,19345354	5,89E-10	8,68E-08	-
PLEKHD1	chr14:69951470-69995215	1,17319	0,145025	-3,01607	-1,89747	0,00115	0,010427	41,9081737	-1,565952973	3,56068754	0,00036989	0,0040384	-
PLIN2	chr9:19115758-19127604	6,39862	19,785	1,62857	3,00198	5,00E-05	0,00070533	802,046913	1,546807208	-7,3833394	1,54E-13	6,01E-11	-
PLP2	chrX:49028183-49031468	7,0575	16,0775	1,18781	1,7834	5,00E-05	0,00070533	261,632515	1,115499679	-5,8546268	4,78E-09	5,09E-07	-
PLSCR1	chr3:146232966-146262628	9,01153	21,0663	1,22509	2,29338	5,00E-05	0,00070533	1014,48917	1,126162602	-4,9872314	6,13E-07	2,60E-05	-
PLVAP	chr19:17462263-17488137	0,351177	1,91212	2,4449	2,32729	5,00E-05	0,00070533	88,062985	1,758338353	-3,8701605	0,00010876	0,00154144	-
PLXNA4	chr7:131808090-132333447	5,05119	2,19931	-1,19957	-1,8546	5,00E-05	0,00070533	1068,96579	-1,223616095	4,86185998	1,16E-06	4,33E-05	-
PNMA3	chrX:152224765-152228827	7,91216	2,1228	-1,89811	-2,93689	5,00E-05	0,00070533	461,58792	-1,511168599	3,88410074	0,00010271	0,001479	-
PNP	chr14:20937537-20946165	5,18195	12,3194	1,24936	2,12364	5,00E-05	0,00070533	556,498928	1,047012632	-3,4447347	0,00057162	0,00564138	-
PNPLA3	chr22:44319618-44343448	1,17234	0,434925	-1,43055	-1,35897	0,00345	0,0256022	65,9076385	-1,382248918	5,31315747	1,08E-07	6,28E-06	-
PODN	chr1:53527723-53551174	4,15177	9,90552	1,25451	2,16041	5,00E-05	0,00070533	654,725751	1,055489969	-2,8063282	0,00501096	0,02892342	-
POLR3G	chr5:89770680-89810369	11,444	3,62673	-1,65785	-2,67723	5,00E-05	0,00070533	657,000829	-1,351611089	3,68699437	0,00022692	0,00271447	-
POM121L9P	chr22:24647588-24661492	0,416937	1,19337	1,51714	1,79547	5,00E-05	0,00070533	145,1662	1,410692021	-5,0414976	4,62E-07	2,06E-05	-
POPDC3	chr6:105585561-105627858	5,99604	2,21156	-1,43894	-1,74252	0,00015	0,00184356	163,947416	-1,223551783	4,39531412	1,11E-05	0,00025503	-
POSTN	chr13:38136718-38172981	0,453648	2,04944	2,17558	2,1069	5,00E-05	0,00070533	142,628633	1,696852638	-4,0116799	6,03E-05	0,00099843	-
POU2F2	chr19:42590261-42636625	0,622873	1,37399	1,14136	1,23946	0,00385	0,0280381	169,49544	1,109891976	-4,4793824	7,49E-06	0,00018954	-
PPEF1	chrX:18706762-18846034	7,7859	2,80762	-1,47152	-2,37032	5,00E-05	0,00070533	524,192088	-1,124308813	2,76530862	0,0056869	0,03169028	-
PPFIA3	chr19:49622645-49654287	5,02618	1,92615	-1,38375	-2,30674	5,00E-05	0,00070533	441,752397	-1,046101699	4,48666222	7,23E-06	0,00018431	-
PPFIA4	chr1:203020310-203047864	3,4238	1,41754	-1,27221	-1,95611	0,0001	0,00129637	329,869353	-1,069735352	4,44437544	8,81E-06	0,00021654	-
PPL	chr16:4932507-4987136	0,49064	1,66862	1,76592	2,34534	5,00E-05	0,00070533	243,363969	1,477020114	-3,6339863	0,00027908	0,00320821	-
PPM1E	chr17:56833229-57184266	7,62716	2,55327	-1,5788	-1,57958	0,00075	0,00731751	784,66589	-1,388111357	4,27981182	1,87E-05	0,00038714	-
PPP1R14C	chr6:150464187-150571528	4,05496	1,19263	-1,76554	-2,08709	5,00E-05	0,00070533	120,318836	-1,360274608	3,62191612	0,00029243	0,00332834	-
PPP1R1C	chr2:182818967-182996109	5,39367	1,81533	-1,57103	-1,58452	0,00015	0,00184356	171,994861	-1,403584139	3,65934296	0,00025286	0,00297143	-
PPP2R2C	chr4:6322304-6565327	47,9686	20,402	-1,23338	-2,65024	5,00E-05	0,00070533	3823,78585	-1,165857699	4,96602079	6,83E-07	2,80E-05	-
PRDM6	chr5:122424840-122523745	0,604292	1,41524	1,22773	1,24327	0,004	0,0288925	89,012259	1,081460205	-2,8109504	0,00493954	0,02861355	-
PRELP	chr1:203444882-203460479	6,33097	19,0693	1,59075	3,01191	5,00E-05	0,00070533	1991,57517	1,279020599	-3,1790377	0,00147765	0,01155849	-
PRG4	chr1:185703682-186446655	0,0355437	2,50518	6,13917	0,492728	0,0021	0,0172529	237,544342	1,896622379	-3,7703518	0,00016302	0,00208806	-
PRKAR1B	chr7:588833-826116	33,4513	11,8171	-1,50119	-2,50458	5,00E-05	0,00070533	1278,33083	-1,234684358	3,71829926	0,00020057	0,00245186	-
PRPH	chr12:49686413-49725514	78,4501	5,65481	-3,79422	-5,37392	5,00E-05	0,00070533	1613,80474	-2,338996415	4,8505711	1,23E-06	4,54E-05	-
PRRT3	chr3:9987225-9996471	4,76919	1,86752	-1,35262	-2,01574	5,00E-05	0,00070533	257,843317	-1,145472914	4,45550118	8,37E-06	0,00020734	-
PRRX1	chr1:170633312-170708541	5,44799	20,8649	1,93728	3,66082	5,00E-05	0,00070533	1342,80566	1,676972626	-5,2255004	1,74E-07	9,00E-06	-
PRRX2	chr9:132427919-132484951	0,418412	2,69545	2,68753	1,64517	0,00405	0,0291035	42,6804648	1,650863383	-3,3374404	0,00084554	0,0075011	-
PRSS12	chr4:119201192-119273922	0,770399	0,245715	-1,64862	-1,59267	0,0009	0,00846161	64,8518005	-1,235331631	3,76820483	0,00016443	0,00210218	-
PRSS23	chr11:86511490-86522273	6,53406	15,2672	1,22439	2,33545	5,00E-05	0,00070533	980,117119	1,033045401	-3,0639055	0,00218468	0,01556719	-
PRSS3	chr9:33750463-33799229	7,02632	1,11483	-2,65595	-2,39969	5,00E-05	0,00070533	71,0796942	-1,758337165	3,97948913	6,91E-05	0,00110219	-
PRSS56	chr2:233385172-233390425	0,983939	0,305929	-1,68537	-1,28465	0,0044	0,0310999	28,5551969	-1,182091299	3,05464288	0,00225329	0,01596175	-
PRUNE2	chr9:79226291-79521003	95,8851	31,3939	-1,61082	-2,20572	5,00E-05	0,00070533	24384,6639	-1,366861636	3,83301349	0,00012658	0,00172754	-
PSTPIP2	chr18:43563501-43652250	0,689761	2,09363	1,60184	1,95131	5,00E-05	0,00070533	147,598653	1,493127016	-6,4538126	1,09E-10	1,98E-08	-
PTAFR	chr1:28473676-28520447	2,63032	6,70899	1,35086	2,23972	5,00E-05	0,00070533	498,54507	1,200030621	-3,7033395	0,00021278	0,0025752	-
PTGDR	chr14:52734430-52743442	1,02371	4,03897	1,98018	2,29528	5,00E-05	0,00070533	188,585998	1,53547868	-3,4372764	0,0005876	0,00574646	-
PTGER3	chr1:71318035-71546972	3,21943	0,780547	-2,04425	-0,967148	0,00765	0,0481377	306,770652	-1,829398163	4,98766967	6,11E-07	2,60E-05	-
PTGIR	chr19:47123724-47128354	0,196584	1,12927	2,52218	1,61866	0,00415	0,0296446	30,830097	1,677651822	-3,5673378	0,00036063	0,00395405	-
PTGS1	chr9:125132808-125157982	2,6588	6,49214	1,28792	2,20056	5,00E-05	0,00070533	618,595161	1,084536796	-3,2943177	0,00098661	0,00838151	-
PTPN5	chr11:18749474-18814268	4,60642	1,71822	-1,42273	-1,98907	5,00E-05	0,00070533	247,29708	-1,157025378	3,4183126	0,00063011	0,00607298	-
PTPN6	chr12:7055739-7070479	3,28589	7,16741	1,12517	1,77735	5,00E-05	0,00070533	380,253089	1,00748081	-3,4534463	0,00055347	0,00551735	-
PTPRC	chr1:198608097-198726605	11,0013	28,8548	1,39114	2,28079	5,00E-05	0,00070533	2749,202	1,310663418	-5,3175844	1,05E-07	6,18E-06	Microglia
PTPRN	chr2:220154344-220174295	21,1543	4,70995	-2,16717	-3,65927	5,00E-05	0,00070533	1095,74651	-1,579717976	3,64627163	0,00026607	0,00309843	-
PTPRT	chr20:40701391-41818557	1,47992	0,577542	-1,35752	-2,10518	5,00E-05	0,00070533	357,941488	-1,07701433	3,01592333	0,00256198	0,01752286	-
PVALB	chr22:37196744-37215517	42,7952	4,30132	-3,3146	-3,94493	5,00E-05	0,00070533	293,897656	-2,164298473	4,57457487	4,77E-06	0,00013343	-
PYCARD	chr16:31212806-31214773	4,77833	11,5116	1,26851	1,51265	0,0013	0,0115379	125,638715	1,115175852	-2,9266787	0,00342603	0,02183324	-
PYGL	chr14:51371934-51411248	4,60963	13,3311	1,53207	2,83962	5,00E-05	0,00070533	917,225696	1,444584519	-7,2703191	3,59E-13	1,37E-10	-
QDPR	chr4:17488015-17513857	293,552	112,692	-1,38124	-2,96556	5,00E-05	0,00070533	6582,8157	-1,350029539	6,63098759	3,33E-11	7,32E-09	-
RAB20	chr13:111175412-111214071	4,52894	12,0461	1,41133	2,08321	5,00E-05	0,00070533	303,548966	1,258737648	-4,4460871	8,74E-06	0,00021508	-
RAB26	chr16:2198650-2204141	2,83464	0,840399	-1,75402	-1,75527	0,0006	0,00609837	72,1111761	-1,352907055	4,10213274	4,09E-05	0,00073525	-
RAB27A	chr15:55495163-55582013	3,21712	6,8258	1,08523	1,79655	0,0001	0,00129637	448,44869	1,005777461	-4,8279783	1,38E-06	5,01E-05	-
RAB3A	chr19:18307610-18314874	33,1643	6,08341	-2,44668	-3,81928	5,00E-05	0,00070533	615,985112	-1,849428297	4,37620595	1,21E-05	0,00027354	-
RAB3B	chr1:52373627-52456436	2,35527	0,631518	-1,899	-2,85894	5,00E-05	0,00070533	500,604868	-1,402677701	3,17515264	0,00149758	0,01169663	-
RAB3C	chr5:57878938-58147406	32,4321	5,26608	-2,62262	-3,53712	5,00E-05	0,00070533	510,371177	-1,851524161	4,07275035	4,65E-05	0,00080905	-

RAB3D	chr19:11406814-11450344	28,8973	7,52772	-1,94065	-3,26598	5,00E-05	0,00070533	1681,20224	-1,543495245	3,80731684	0,00014048	0,00186662	-
RAB42	chr1:28918711-28921088	0,305683	2,8217	3,20646	2,5955	5,00E-05	0,00070533	50,6323669	2,716305883	-6,8770853	6,11E-12	1,60E-09	-
RAB6B	chr3:133543079-133614691	49,2622	19,2917	-1,3525	-2,73225	5,00E-05	0,00070533	4344,0281	-1,236491181	4,11641827	3,85E-05	0,00070022	-
RAC2	chr22:37621300-37640339	1,7718	4,13645	1,22318	1,45643	0,00075	0,00731751	113,741374	1,21104813	-5,3607167	8,29E-08	5,06E-06	-
RALYL	chr8:85095452-85834078	5,84616	2,27729	-1,36017	-1,88777	0,00015	0,00184356	254,911547	-1,065142268	3,08053614	0,00206628	0,0149083	-
RBFOX1	chr16:6069131-7763340	9,71285	2,39891	-2,01752	-3,19767	5,00E-05	0,00070533	636,343176	-1,550224645	4,44218596	8,90E-06	0,00021824	-
RBFOX3	chr17:77085426-77512230	2,34146	0,441077	-2,4083	-2,5189	5,00E-05	0,00070533	93,8400609	-1,806587377	4,31519206	1,59E-05	0,00033853	-
RBM47	chr4:40425271-40631883	1,09924	3,94638	1,84402	2,62658	5,00E-05	0,00070533	351,396103	1,729358005	-7,0202013	2,22E-12	6,53E-10	-
RBP4	chr10:95351592-95360993	1,65766	5,36052	1,69322	1,75065	0,0001	0,00129637	82,6367814	1,251912218	-2,786867	0,00532203	0,03016208	-
RCAN2	chr6:46188466-46459804	48,6339	16,8285	-1,53105	-3,13423	5,00E-05	0,00070533	2512,57403	-1,417890307	4,77378754	1,81E-06	6,27E-05	-
REEP1	chr2:86441119-86565206	33,3492	5,23957	-2,67013	-4,41465	5,00E-05	0,00070533	1627,11787	-2,159414779	5,30775056	1,11E-07	6,38E-06	-
REEP2	chr5:137774689-137782658	41,4719	15,722	-1,39935	-2,84216	5,00E-05	0,00070533	1365,92921	-1,239602889	4,49620894	6,92E-06	0,00017918	-
RELL2	chr5:141016516-141030986	13,2032	3,09161	-2,09446	-2,18973	0,0002	0,0023747	390,711929	-1,598809744	4,40825888	1,04E-05	0,00024463	-
RET	chr10:43572516-43625797	15,7637	2,99234	-2,39726	-4,20547	5,00E-05	0,00070533	1074,64244	-2,014688705	5,89093366	3,84E-09	4,28E-07	-
RFPL1S	chr22:29833003-29838444	4,97646	2,10293	-1,24272	-2,06443	5,00E-05	0,00070533	464,184929	-1,1120097	3,40008974	0,00067364	0,00632704	-
RFPL2	chr22:32586421-32600718	2,12192	0,328292	-2,69232	-2,03468	5,00E-05	0,00070533	47,8334412	-1,463416669	3,03308798	0,00242065	0,01687504	-
RFTN1	chr3:16357351-16555222	4,28704	11,0578	1,36701	2,42453	5,00E-05	0,00070533	674,498546	1,265828095	-4,6583925	3,19E-06	9,74E-05	-
RGS1	chr1:192544856-192549159	60,7783	187,429	1,62471	3,17966	5,00E-05	0,00070533	6729,81501	1,279345174	-3,9302242	8,49E-05	0,00128483	-
RGS18	chr1:192127591-192154945	1,318	3,63471	1,46349	1,83299	0,0002	0,0023747	153,063869	1,302571163	-4,7336378	2,21E-06	7,30E-05	-
RGS4	chr1:163038395-163046592	7,10165	2,46071	-1,52908	-2,32582	5,00E-05	0,00070533	357,722926	-1,311875509	3,68097371	0,00023235	0,00276496	Neuron
RGS7	chr1:240938813-241520530	4,97946	1,38286	-1,84834	-2,53307	5,00E-05	0,00070533	263,509115	-1,49924948	5,28401228	1,26E-07	7,07E-06	-
RGS7BP	chr5:63801773-63908121	14,9869	2,1965	-2,77042	-4,03278	5,00E-05	0,00070533	845,025779	-2,17896122	5,22595676	1,73E-07	9,00E-06	-
RHBDF2	chr17:74466974-74497509	3,79174	11,5716	1,60965	2,88921	5,00E-05	0,00070533	824,657248	1,446413745	-5,6674416	1,45E-08	1,22E-06	-
RHBDL3	chr17:30593194-30651680	6,5512	2,68177	-1,28857	-2,25077	5,00E-05	0,00070533	510,902303	-1,199727291	6,08452481	1,17E-09	1,56E-07	-
RIMKLA	chr1:42846467-42889900	8,15027	1,27851	-2,67239	-4,45093	5,00E-05	0,00070533	1186,39566	-2,162263879	5,34216108	9,18E-08	5,53E-06	-
RIMS1	chr6:72596405-73112845	11,5788	3,90999	-1,56624	-3,05191	5,00E-05	0,00070533	1763,27489	-1,408204981	6,10247601	1,04E-09	1,40E-07	-
RIMS2	chr8:104512975-105266656	4,29972	1,76049	-1,28827	-2,17081	5,00E-05	0,00070533	608,15647	-1,107050569	3,72351666	0,00019647	0,00240742	-
RIN3	chr14:92980124-93155334	2,89709	6,31112	1,12329	1,90505	5,00E-05	0,00070533	484,818038	1,067573397	-5,9073913	3,48E-09	4,01E-07	-
RIPK3	chr14:24805226-24809242	0,587906	1,59256	1,4377	1,37848	0,0043	0,0305473	78,8952311	1,187833414	-5,627045	1,83E-08	1,48E-06	-
RNASE2	chr14:21423629-21424594	0,43631	3,54641	3,02294	1,85769	0,00165	0,0141149	39,9557279	2,147981317	-4,7458182	2,08E-06	6,93E-05	-
RNASE6	chr14:21249209-21250626	4,48405	15,0433	1,74624	2,39513	5,00E-05	0,00070533	251,723126	1,548278668	-4,9572895	7,15E-07	2,89E-05	-
RNASET2	chr6:167343003-167370077	20,6236	53,5061	1,37541	2,60959	5,00E-05	0,00070533	1280,11937	1,252204661	-4,5203205	6,17E-06	0,0001644	-
RND1	chr12:49250915-49259653	9,45302	3,98552	-1,24601	-1,91485	5,00E-05	0,00070533	302,977291	-1,076693875	4,63791731	3,52E-06	0,00010556	-
RNF128	chrX:105937067-106040246	4,05762	1,21894	-1,73501	-2,06342	5,00E-05	0,00070533	162,172235	-1,346314959	3,28106256	0,00103417	0,00872091	-
RNF157	chr17:74132414-74236390	27,7694	10,2567	-1,43693	-2,33017	5,00E-05	0,00070533	2517,26362	-1,294708106	4,77674419	1,78E-06	6,19E-05	-
RNF175	chr4:154631311-154681387	5,52543	1,03369	-2,41828	-2,39024	5,00E-05	0,00070533	112,10417	-1,637967428	3,92800083	8,57E-05	0,00129035	-
RPH3A	chr12:113229548-113336684	13,1304	2,10603	-2,64032	-4,30926	5,00E-05	0,00070533	954,674362	-2,016281028	4,98314955	6,26E-07	2,64E-05	-
RRM2	chr2:10262694-10271546	0,0840307	0,644491	2,93917	1,67847	0,00065	0,00650426	31,052247	1,928945834	-4,0171688	5,89E-05	0,0009794	-
RSPO3	chr6:127440047-127520626	0,178189	1,27956	2,84416	2,62914	5,00E-05	0,00070533	93,2267544	1,562797633	-3,1549398	0,00160531	0,01231645	-
RTBDN	chr19:12936290-12946242	1,29982	0,203925	-2,6722	-1,61507	5,00E-05	0,00070533	25,5234181	-1,241405844	2,74572443	0,00603775	0,0329513	-
RTL1	chr14:101346991-101351184	1,18822	0,167913	-2,82302	-2,73071	5,00E-05	0,00070533	80,781407	-2,201025486	5,25042173	1,52E-07	8,09E-06	-
RTN1	chr14:60062693-60337557	133,201	43,8022	-1,60453	-2,837	5,00E-05	0,00070533	4473,74783	-1,150277439	3,63888797	0,00027382	0,00316362	-
RTN2	chr19:45988545-46000313	10,8862	2,57848	-2,07791	-2,87277	5,00E-05	0,00070533	303,651237	-1,672401414	4,40181514	1,07E-05	0,00024889	-
RUNDC3A	chr17:42385926-42396038	37,6248	13,125	-1,51937	-3,0229	5,00E-05	0,00070533	1245,96037	-1,298989688	4,68382292	2,82E-06	8,90E-05	-
RUNX1	chr21:36160097-36421595	2,07993	5,7607	1,46971	2,10566	5,00E-05	0,00070533	557,876313	1,378841699	-4,7931987	1,64E-06	5,75E-05	-
RUNX3	chr1:25226001-25291501	0,442058	1,09468	1,3082	1,33888	0,0033	0,0247184	79,7435251	1,2098927	-3,9765243	6,99E-05	0,00111343	-
RYR2	chr1:237205701-237997288	6,04666	1,96246	-1,62348	-3,21067	5,00E-05	0,00070533	2147,14143	-1,38214677	4,56110025	5,09E-06	0,00014019	-
S100A11	chr1:152004981-152009511	16,5575	52,0069	1,65122	2,735	5,00E-05	0,00070533	463,123597	1,571289267	-6,6688326	2,58E-11	5,78E-09	-
S100A3	chr1:153519808-153521734	0,641722	2,11734	1,72223	1,23174	0,0036	0,0264814	24,5674308	1,403898633	-3,6547414	0,00025744	0,00301136	-
S100A4	chr1:153516094-153518282	15,0265	35,6263	1,24543	1,82486	5,00E-05	0,00070533	318,514659	1,037690952	-2,5943174	0,0094779	0,04622462	-
S100A9	chr1:153330329-153333503	6,62682	27,7635	2,0668	2,48371	5,00E-05	0,00070533	284,443584	1,491989846	-3,3053864	0,00094846	0,00814456	-
S100P	chr4:6695565-6698897	0,259997	6,54216	4,6532	2,62707	0,00025	0,00289273	39,1894014	2,599770261	-5,2813262	1,28E-07	7,11E-06	-
S1PR3	chr9:91605777-91620069	10,7068	25,2383	1,23708	2,32314	5,00E-05	0,00070533	1804,71718	1,04819275	-3,3200535	0,0009	0,00783279	-
SAA2	chr11:18252901-18270221	0,103816	1,59038	3,93726	1,66443	0,00315	0,0238285	16,9148869	1,687090117	-3,3938155	0,00068926	0,00644737	-
SAMSN1	chr21:15857548-16015428	2,61069	7,13459	1,4504	2,14682	5,00E-05	0,00070533	345,048288	1,333015649	-4,9668901	6,80E-07	2,79E-05	-
SASH3	chrX:128913891-128929176	2,18421	5,65259	1,3718	2,07625	5,00E-05	0,00070533	306,69811	1,190120962	-4,1002545	4,13E-05	0,00073668	-
SBNO2	chr19:1107632-1174282	5,04012	10,9818	1,12358	2,1339	5,00E-05	0,00070533	993,010528	1,002858874	-3,6211538	0,00029329	0,00333551	-
SCAMP5	chr15:75287875-75313836	25,5585	11,0676	-1,20746	-2,44881	5,00E-05	0,00070533	1229,54251	-1,092828757	4,29411124	1,75E-05	0,00036595	-
SCIMP	chr17:5095378-5138931	0,638178	2,40273	1,91265	1,67272	0,0032	0,0241415	110,649288	1,632739653	-5,5319836	3,17E-08	2,24E-06	-
SCIN	chr7:12610202-12693228	6,81261	21,1233	1,63256	3,07254	5,00E-05	0,00070533	1445,5756	1,328349807	-3,5743524	0,0003511	0,00386805	-
SCN1A	chr2:166813927-167232497	16,801	5,77525	-1,54059	-2,09612	5,00E-05	0,00070533	2527,60915	-1,198286941	3,0113051	0,00260127	0,01769775	-
SCN1B	chr19:35521591-35531353	41,108	15,5779	-1,39992	-2,5994	5,00E-05	0,00070533	773,62903	-1,190532247	3,82778197	0,0001293	0,00175192	-
SCN4B	chr11:118004091-118023630	31,1896	4,58526	-2,76599	-4,70401	5,00E-05	0,00070533	1748,79104	-2,137974732	5,10601435	3,29E-07	1,54E-05	-
SCN8A	chr12:51985019-52206648	11,961	2,04443	-2,54857	-4,41785	5,00E-05	0,00070533	2116,03639	-1,960154969	4,76196567	1,92E-06	6,55E-05	-
SCN9A	chr2:166813927-167232497	7,29808	3,22688	-1,17738	-1,11321	0,00745	0,047144	1361,3921	-1,017177739	2,96697212	0,00300748	0,0197658	-
SCRT1	chr8:145554227-145559943	5,76729	1,21101	-2,25168	-2,97894	5,00E-05	0,00070533	216,971642	-1,788567095	4,40827489	1,04E-05	0,00024463	-
SCUBE1	chr22:43599228-43739394	0,762668	3,15789	2,04984	2,52164	5,00E-05	0,00070533	244,664332	1,602117147	-3,6694692	0,00024306	0,00287417	-
SDR16C5	chr8:57212569-57233241	1,52939	0,396844	-1,94631	-1,86538	0,0002	0,0023747	65,9588607	-1,530234118	3,65672192	0,00025546	0,00299514	-
SEC14L5	chr16:5008317-5069156	28,7852	12,3392	-1,22208	-2,58994	5,00E-05	0,00070533	3356,58978	-1,126160479	4,08277185	4,45E-05	0,00078153	-
SEC24D	chr4:119643977-119757326	3,37013	7,27472	1,11009	1,98437	5,00E-05	0,00070533	693,740899	1,019112809	-4,3058506	1,66E-05	0,00035026	-
SECTM1	chr17:80278899-80291921	0,488824	1,27769	1,38615	1,30062	0,0034	0,0252982	45,3563007	1,300887174	-3,9283432	8,55E-05	0,00129019	-
SEMA3F	chr3:50192847-50226508	1,03605	2,55861	1,30427	1,6604	5,00E-05	0,00070533	177,381056	1,056653224	-2,7184605	0,00655865	0,03507976	-
SERPINA1	chr14:94843083-94857029	2,42065	9,72232	2,00591	3,21661	5,00E-05	0,00070533	569,544726	1,770825327	-5,4901423	4,02E-08	2,74E-06	-
SERPINA3	chr14:95078713-95090390	42,9171	338,446	2,9793	5,94478	5,00E-05	0,00070533	9712,99552	2,687969288	-9,2238233	2,87E-20	4,93E-17	-
SERPINA5	chr14:95047705-95059457	1,65032	3,80398	1,20477	1,56829	0,0005	0,00518549	189,367556	1,019296117	-2,7528433	0,00590802	0,03245791	-
SERPINF1	chr17:1665258-1680859	4,47274	29,6985	2,73116	4,08651	5,00E-05	0,00070533	803,273851	2,043736583	-4,6244639	3,76E-06	0,00011071	-
SERPING1	chr11:57365026-57382326	22,4722	49,0691	1,12667	2,36985	5,00E-05	0,00070533	2027,56169	1,066308502	-4,6012854	4,20E-06	0,00012084	-
SEZ6	chr17:27281946-27333458	3,84702	1,21992	-1,65696	-2,32576	5,00E-05	0,00070533	258,893469	-1,413005117	4,31304367	1,61E-05	0,00034009	-
SEZ6L	chr22:26565439-26779563	15,2133	6,62499	-1,19934	-2,3538	5,00E-05	0,00070533	1775,50159	-1,059890577	3,54256234	0,00039626	0,00427211	-
SEZ6L2	chr16:29882479-29910585	24,5962	6,38817	-1,94496	-3,70516	5,00E-05	0,00070533	1195,63845	-1,597140998	4,59407123	4,35E-06	0,00012372	-
SFN	chr1:27189632-27190947	0,455222	1,35335	1,57189	1,1904	0,0039	0,0283163	27,8257206	1,214525435	-2,7785129	0,00546083	0,03072929	-
SFRP2	chr4:154701741-154710228	11,896	63,5338	2,41705	4,01247	5,00E-05	0,00070533	1797,72965	1,784892496	-4,035672	5,44E-05	0,00091714	-
SHANK1	chr19:51165083-51220195	3,37155	1,36109	-1,30865	-2,04858	5,00E-05	0,00070533	349,392882	-1,111633561	4,01692608	5,90E-05	0,00097962	-
SHC3	chr9:91620685-91793682	9,81544	3,86949	-1,34291	-2,66167	5,00E-05	0,00070533	1669,83175	-1,176492887	3,69790632	0,00021739	0,00261865	-
SHH	chr7:155595557-155604967	8,45221	0,766855	-3,4623	-3,43478	5,00E-05	0,00070533	146,146926	-2,280194016	4,91587933	8,84E-07	3,41E-05	-
SIAH3	chr13:46354415-46425846	0,685476	0,174554	-1,97343	-1,64795	0,0015	0,013051	41,7701897	-1,582559507	3,95194612	7,75E-05	0,0012027	-
SIGLEC1	chr20:3667616-3687775	0,592214	1,3154	1,15131	1,49967	0,0005	0,00518549	186,866286	1,07819173	-3,4283995	0,00060715	0,0058958	-
SIGLEC10	chr19:51913274-51921057	1,58375	6,74781	2,09107	3,293	5,00E-05	0,00070533	422,46067	1,943259617	-8,6960825	3,44E-18	4,17E-15	-
SIGLEC14	chr19:52145805-52150132	0,630316	1,86047	1,56152	1,56399	0,00135	0,0119	85,9019732	1,317551983	-3,951426	7,77E-05	0,00120351	-
SIGLEC5	chr19:52114755-52133727	0,251626	0,772566	1,61838	1,25876	0,00775	0,048585	41,8032799	1,462763898	-4,432394	9,32E-06	0,00022581	-
SIGLEC7	chr19:51645557-51685222	0,321923	1,82924	2,50646	2,03294	5,00E-05	0,00070533	60,9925595	2,090578937	-5,8419967	5,16E-09	5,43E-07	-
SIGLEC8	chr19:51954250-51961708	2,15171	14,5102	2,75351	4,33059	5,00E-05	0,00070533	693,383483	2,379968487	-7,1470304	8,87E-13	3,00E-10	-
SIGLEC9	chr19:51628136-51639520	1,27549	5,51069	2,11118	2,5275	5,00E-05	0,00070533	185,087644	1,898609641	-6,0328299	1,61E-09	2,05E-07	-
SIRPB2	chr20:1455235-1472233	0,780641	2,57524	1,72197	2,08487	5,00E-05	0,00070533	176,53707	1,432876845	-4,7955666	1,62E-06	5,71E-05	-
SIX1	chr14:61111416-61116155	0,112887	8,88136	6,29783	4,11818	5,00E-05	0,00070533	322,927464	1,980613192	-3,9309007	8,46E-05	0,00128216	-
SLA	chr8:133879204-134147143	2,8959	13,1065	2,1782	3,54609	5,00E-05	0,00070533	742,517386	1,975835352	-7,1143084	1,12E-12	3,65E-10	Neuron
SLC10A4	chr4:48485359-48491541	18,576	0,862375	-4,42898	-4,49313	5,00E-05	0,00070533	374,283381	-2,946917709	6,29330862	3,11E-10	4,93E-08	-
SLC11A1	chr2:219246751-219261617	1,32278	13,1624	3,31477	4,84998	5,00E-05	0,00070533	935,821109	2,682105982	-7,9656435	1,64E-15	9,79E-13	-
SLC12A5	chr20:44650328-44688789	21,3296	3,16883	-2,75083	-4,57711	5,00E-05	0,00070533	1595,04229	-1,967434549	4,44063642	8,97E-06	0,0002192	Neuron
SLC13A4	chr7:135365986-135412933	0,910105	4,97732	2,45126	3,06167	5,00E-05	0,00070533	281,816476	1,806187668	-4,0495384	5,13E-05	0,00087663	-
SLC13A5	chr17:6588031-6616740	2,23659	0,177233	-3,65758	-3,27835	5,00E-05	0,00070533	100,418004	-2,780934217	8,90908975	5,15E-19	7,08E-16	-
SLC14A1	chr18:43304091-43332485	15,2482	49,7214	1,70523	3,62761	5,00E-05	0,00070533	4077,87766	1,592062153	-5,8186009	5,93E-09	5,92E-07	Astrocyte
SLC17A6	chr11:22359666-22401046	4,82045	1,05669	-2,18962	-2,55189	5,00E-05	0,00070533	272,735653	-1,431564315	3,00986015	0,00261368	0,01775874	Neuron
SLC17A9	chr20:61583998-61599949	0,628867	1,71908	1,45081	1,3997	0,00385	0,0280381	94,5216847	1,234902521	-4,715399	2,41E-06	7,84E-05	-
SLC18A3	chr10:50817140-50873150	17,0185	1,82862	-3,21828	-3,56269	5,00E-05	0,00070533	321,839895	-2,409317991	5,66777874	1,45E-08	1,22E-06	-
SLC1A2	chr11:35272751-35441610	307,907	70,068	-2,13567	-3,40832	5,00E-05	0,00070533	49174,1861	-2,074744234	9,32781654	1,08E-20	2,23E-17	Astrocyte
SLC1A5	chr19:47278139-47291842	4,44719	13,8861	1,64268	2,70237	5,00E-05	0,00070533	529,970996	1,521202716	-6,1166549	9,56E-10	1,31E-07	-
SLC24A2	chr9:19507449-19787017	39,8816	18,9106	-1,07653	-2,29701	5,00E-05	0,00070533	8268,34376	-1,045593767	5,19097156	2,09E-07	1,05E-05	-
SLC26A2	chr5:149340299-149366963	4,50055	22,2833	2,30779	4,63573	5,00E-05	0,00070533	2580,32758	2,000998414	-6,2743316	3,51E-10	5,45E-08	-
SLC26A7	chr8:92114846-92410382	0,154961	2,7677	4,15871	3,46237	5,00E-05	0,00070533	199,941712	2,66858018	-5,7220536	1,05E-08	9,59E-07	-
SLC29A4	chr7:5322560-5343704	15,6192	4,93449	-1,66235	-2,95511	5,00E-05	0,00070533	617,725749	-1,582232455	7,78543667	6,95E-15	3,68E-12	-
SLC2A5	chr1:9097004-9129887	6,57343	21,3158	1,69721	2,76604	5,00E-05	0,00070533	794,580367	1,534118703	-5,7790333	7,51E-09	7,18E-07	-
SLC2A6	chr9:136336215-136344276	12,053	4,32407	-1,47893	-2,43184	5,00E-05	0,00070533	448,798978	-1,258432239	4,59089889	4,41E-06	0,0001251	-
SLC32A1	chr20:37353104-37358015	6,26262	1,28038	-2,29019	-2,80732	5,00E-05	0,00070533	167,816197	-1,673607019	3,78951985	0,00015094	0,00197248	-
SLC35F3	chr1:234040678-234460262	5,45146	1,32763	-2,03779	-2,68328	5,00E-05	0,00070533	217,113309	-1,585960989	4,1840457	2,86E-05	0,00054816	-
SLC35F4	chr14:58030639-58332592	0,970847	0,17225	-2,49474	-1,57327	0,004	0,0288925	30,2189603	-1,405881369	3,1250156	0,00177796	0,01330244	-
SLC37A2	chr11:124933012-124960412	2,09431	6,25917	1,57949	2,35607	5,00E-05	0,00070533	465,982977	1,227728789	-3,3114305	0,0009282	0,00801065	-
SLC39A12	chr10:18240767-18332221	11,0143	4,22149	-1,38356	-2,35013	5,00E-05	0,00070533	528,545645	-1,293981452	5,16742092	2,37E-07	1,17E-05	Astrocyte
SLC39A14	chr8:22224761-22291640	6,72668	23,9837	1,83409	3,55284	5,00E-05	0,00070533	1806,26395	1,605577012	-5,4008431	6,63E-08	4,19E-06	-
SLC43A3	chr11:57174426-57195053	3,76795	8,24426	1,12961	1,9022	5,00E-05	0,00070533	472,099092	1,087851997	-5,7159281	1,09E-08	9,83E-07	-
SLC45A1	chr1:8378144-8404227	7,22663	2,69952	-1,42062	-2,11233	5,00E-05	0,00070533	255,103379	-1,180294592	4,07060069	4,69E-05	0,00081312	-

SLC45A4	chr8:142217264-142264728	6,15148	2,66558	-1,20648	-2,03221	5,00E-05	0,00070533	660,926793	-1,043641429	3,55916677	0,00037203	0,00405496	-
SLC47A1	chr17:19437166-19482346	2,36394	14,8081	2,64712	3,73538	5,00E-05	0,00070533	742,850571	1,448459565	-2,896261	0,00377638	0,02345039	-
SLC4A3	chr2:220492291-220506702	13,799	4,95819	-1,47668	-2,71855	5,00E-05	0,00070533	894,392236	-1,209476145	4,12570402	3,70E-05	0,00067733	-
SLC52A3	chr20:740723-749228	1,90256	6,86231	1,85075	2,57632	5,00E-05	0,00070533	276,769079	1,458149804	-3,7140773	0,00020395	0,0024854	-
SLC5A5	chr19:17982781-18005983	0,0687092	0,634246	3,20647	1,92911	0,00125	0,0111709	32,9118423	2,489229053	-5,9346708	2,94E-09	3,53E-07	-
SLC5A7	chr2:108602994-108630443	34,068	2,99308	-3,50872	-4,93693	5,00E-05	0,00070533	2230,85516	-1,901581017	3,77424504	0,00016049	0,00206727	-
SLC6A11	chr3:10857916-10980146	31,6589	8,04054	-1,97725	-3,59792	5,00E-05	0,00070533	887,404578	-1,871596527	7,61253476	2,69E-14	1,18E-11	-
SLC6A15	chr12:85253266-85306608	10,9104	2,22482	-2,29394	-3,47494	5,00E-05	0,00070533	652,347144	-1,851876539	4,53877666	5,66E-06	0,00015403	-
SLC6A20	chr3:45796940-45838035	1,05735	3,14576	1,57296	2,25748	5,00E-05	0,00070533	300,218059	1,205401092	-2,8332477	0,00460777	0,02725427	-
SLC6A5	chr11:20620945-20676610	11,8152	1,55158	-2,92883	-3,51864	5,00E-05	0,00070533	434,05883	-1,515194215	3,0006908	0,00269368	0,01815913	-
SLC7A14	chr3:170177341-170303863	18,4949	5,80284	-1,6723	-3,20031	5,00E-05	0,00070533	3046,82061	-1,45896684	4,0736648	4,63E-05	0,00080724	-
SLC7A2	chr8:17354596-17428077	12,1582	39,0287	1,68261	3,55316	5,00E-05	0,00070533	4428,3844	1,532727333	-5,7005505	1,19E-08	1,05E-06	Astrocyte
SLC7A7	chr14:23242431-23289020	2,47792	8,25012	1,73529	2,54772	5,00E-05	0,00070533	326,112613	1,598008911	-6,3119548	2,76E-10	4,49E-08	-
SLC8A3	chr14:70510933-70655787	3,63576	1,53693	-1,2422	-1,98955	5,00E-05	0,00070533	330,360516	-1,122453565	3,82038486	0,00013324	0,00179705	-
SLC9A7	chrX:46458685-46618607	13,9516	4,86929	-1,51864	-2,86905	5,00E-05	0,00070533	2281,07156	-1,299474013	3,51601443	0,00043808	0,00462154	-
SLCO2A1	chr3:133651539-133748920	0,709776	2,5124	1,82363	2,3076	5,00E-05	0,00070533	178,078447	1,69843649	-6,2661878	3,70E-10	5,70E-08	-
SLCO4A1	chr20:61273796-61303647	2,73963	24,3526	3,15202	4,64146	5,00E-05	0,00070533	884,166595	2,223146387	-4,9628794	6,95E-07	2,83E-05	-
SLITRK4	chrX:142710594-142723926	3,61694	0,985192	-1,87629	-2,68292	5,00E-05	0,00070533	450,923413	-1,512648101	3,60339018	0,00031409	0,00353977	-
SLPI	chr20:43880879-43883205	1,89637	125,624	6,04972	6,1576	5,00E-05	0,00070533	994,914385	2,008043995	-3,9779832	6,95E-05	0,00110833	-
SMAD6	chr15:66994673-67074337	3,80082	10,5664	1,4751	2,23128	5,00E-05	0,00070533	349,431893	1,382928829	-5,0605662	4,18E-07	1,89E-05	-
SMIM17	chr19:57154526-57168614	6,63875	1,88042	-1,81986	-2,21872	5,00E-05	0,00070533	199,434908	-1,467973802	3,74358177	0,00018142	0,00226605	-
SMIM3	chr5:150157507-150176298	3,90772	8,56053	1,13137	1,80446	0,0001	0,00129637	340,247743	1,005821565	-3,4718631	0,00051686	0,00524098	-
SNAI2	chr8:49830238-49833999	0,944448	3,28061	1,79642	2,02399	5,00E-05	0,00070533	114,552538	1,524225796	-4,6153759	3,92E-06	0,00011469	-
SNAP25	chr20:10004459-10288066	232,315	36,6625	-2,66371	-4,36353	5,00E-05	0,00070533	6695,68697	-1,740946272	3,60528961	0,00031181	0,00352041	Neuron
SNAP91	chr6:84262604-84419127	31,7503	7,7241	-2,03933	-3,53413	5,00E-05	0,00070533	2000,93389	-1,6276222	4,17032534	3,04E-05	0,00057529	-
SNCB	chr5:176047209-176057557	19,1329	4,74134	-2,01269	-2,8835	5,00E-05	0,00070533	296,978337	-1,40768452	3,16022645	0,00157647	0,0121791	-
SNCG	chr10:88718287-88723017	130,709	28,2775	-2,20863	-4,05013	5,00E-05	0,00070533	1578,53966	-1,698029458	4,21221974	2,53E-05	0,00049367	-
SNPH	chr20:1246959-1289971	17,8142	6,5011	-1,45427	-2,87037	5,00E-05	0,00070533	1363,19748	-1,279507061	4,13035531	3,62E-05	0,00066792	-
SNX10	chr7:26331514-26413949	48,3872	14,7923	-1,70978	-2,97321	5,00E-05	0,00070533	1708,28488	-1,38318809	3,40136134	0,00067051	0,00631493	-
SOAT1	chr1:179262848-179327814	7,0464	17,081	1,27744	2,6922	5,00E-05	0,00070533	1932,01378	1,212358995	-6,4746319	9,50E-11	1,77E-08	-
SOD2	chr6:160100148-160114353	120,911	265,45	1,13449	2,39563	5,00E-05	0,00070533	8017,08226	1,058793795	-5,7662827	8,10E-09	7,65E-07	-
SOWAHD	chrX:118892575-118894165	0,368185	1,21872	1,72686	1,29379	0,004	0,0288925	26,9914769	1,302262171	-3,0088836	0,0026221	0,01779249	-
SPI1	chr11:47376408-47400127	4,23144	11,31	1,41838	2,0117	5,00E-05	0,00070533	264,361773	1,270392553	-4,854534	1,21E-06	4,46E-05	-
SPIRE2	chr16:89894906-89937727	5,64312	1,90804	-1,5644	-2,26901	5,00E-05	0,00070533	284,052613	-1,273133348	3,95975871	7,50E-05	0,00117462	-
SPN	chr16:29674270-29681823	0,899815	2,31157	1,36117	2,05306	5,00E-05	0,00070533	296,092392	1,256216495	-4,619943	3,84E-06	0,00011285	-
SPTB	chr14:65213000-65289866	5,01677	2,02657	-1,30772	-2,42049	5,00E-05	0,00070533	912,683719	-1,104210806	3,84190637	0,00012208	0,00168281	-
SPTBN2	chr11:66452719-66488870	11,5753	4,16202	-1,47569	-2,98536	5,00E-05	0,00070533	1724,33059	-1,223872628	3,95470868	7,66E-05	0,00119157	-
SPTSSB	chr3:161062579-161089871	2,61683	0,484404	-2,43354	-1,98302	0,00045	0,00473416	74,3598666	-1,698832839	3,74052548	0,00018364	0,00227998	-
SQLE	chr8:126010719-126034525	55,3877	17,8618	-1,63269	-3,48588	5,00E-05	0,00070533	2474,39022	-1,567229515	8,02826103	9,89E-16	6,18E-13	-
SQRDL	chr15:45923345-45983492	5,76869	12,2657	1,08831	1,9144	5,00E-05	0,00070533	511,490561	1,029593003	-4,3831319	1,17E-05	0,00026763	-
SRD5A1	chr5:6633499-6669675	19,0541	7,51109	-1,34301	-2,2656	5,00E-05	0,00070533	549,098401	-1,181353959	3,75159434	0,00017571	0,00220685	-
SRRM3	chr7:75831210-75916609	2,72358	0,952581	-1,51559	-1,91153	5,00E-05	0,00070533	145,658863	-1,1980772	3,63815138	0,0002746	0,00317091	-
SRRM4	chr12:119419299-119600856	1,32196	0,36792	-1,84521	-2,29814	5,00E-05	0,00070533	179,484194	-1,365178471	3,62683405	0,00028692	0,00328737	-
SSTR2	chr17:71161159-71168062	1,79774	4,54804	1,33906	1,9058	0,0001	0,00129637	240,400919	1,114840969	-2,9463966	0,003215	0,0207902	Neuron
SSTR3	chr22:37600276-37608416	2,21899	0,502656	-2,14226	-2,48561	5,00E-05	0,00070533	111,853815	-1,617670188	3,88674564	0,0001016	0,00146811	-
ST14	chr11:130029681-130080257	0,061112	1,53494	4,65058	3,06079	5,00E-05	0,00070533	77,7787242	3,706779763	-10,396405	2,57E-25	6,64E-22	-
ST8SIA3	chr18:55019720-55036161	2,9603	0,850788	-1,79887	-2,69233	5,00E-05	0,00070533	469,07088	-1,428991282	3,38433568	0,00071351	0,0066148	-
STAB1	chr3:52529355-52569093	5,93625	14,0689	1,24488	2,30977	5,00E-05	0,00070533	2223,04431	1,125324892	-3,895753	9,79E-05	0,0014286	-
STAC2	chr17:37366788-37382040	2,52489	0,832528	-1,60065	-2,01223	5,00E-05	0,00070533	139,715284	-1,287150906	3,79340896	0,00014859	0,00194681	-
STAR	chr8:38000217-38008600	1,4759	0,461201	-1,67812	-1,64544	0,00095	0,00886719	70,3042125	-1,449988922	4,23640171	2,27E-05	0,00045283	-
STC1	chr8:23699433-23712320	0,457993	3,8204	3,06033	3,5754	5,00E-05	0,00070533	223,401206	2,048023029	-4,4706467	7,80E-06	0,000196	-
STEAP3	chr2:119981383-120023227	6,19681	14,5283	1,22927	2,40596	5,00E-05	0,00070533	1044,83083	1,144822169	-6,0496844	1,45E-09	1,88E-07	-
STEAP4	chr7:87905743-87936228	1,88641	10,6386	2,49559	3,84469	5,00E-05	0,00070533	681,599369	1,946821646	-4,7952853	1,62E-06	5,71E-05	-
STMN2	chr8:80523048-80578410	218,259	22,9352	-3,25041	-4,56594	5,00E-05	0,00070533	6857,18643	-2,007708195	4,08797484	4,35E-05	0,00076682	Neuron
STOM	chr9:124101265-124132582	72,6018	164,506	1,18006	2,44081	5,00E-05	0,00070533	8739,18793	1,089363904	-5,8792661	4,12E-09	4,50E-07	-
STX11	chr6:144471653-144513076	0,515144	1,38278	1,42452	1,71345	0,0001	0,00129637	124,971836	1,180905234	-3,5224091	0,00042764	0,00453232	-
STX1B	chr16:31000576-31021829	20,0254	4,88064	-2,03669	-3,73462	5,00E-05	0,00070533	1289,11374	-1,665055223	4,32940113	1,50E-05	0,00032239	-
STXBP1	chr9:130374485-130454995	42,5228	16,5869	-1,35819	-2,80452	5,00E-05	0,00070533	2974,19673	-1,224813225	4,40523096	1,06E-05	0,00024617	-
STXBP2	chr19:7701986-7712760	1,40866	4,43799	1,65558	2,06753	5,00E-05	0,00070533	185,104911	1,373033931	-5,3376065	9,42E-08	5,65E-06	-
SULF1	chr8:70378858-70573147	4,82557	13,271	1,45951	2,92998	5,00E-05	0,00070533	1594,28154	1,282993545	-4,0696069	4,71E-05	0,00081523	-
SULT1B1	chr4:70592685-70626430	0,625076	3,5079	2,4885	2,01191	0,00035	0,00384337	102,018198	1,941569899	-4,3469825	1,38E-05	0,00030235	-
SULT1C2	chr2:108905094-108926371	0,644863	1,82346	1,49962	1,69375	0,00015	0,00184356	123,892619	1,195301963	-2,9376564	0,00330703	0,02125215	-
SULT4A1	chr22:44220386-44258378	31,5617	3,95229	-2,99741	-4,45321	5,00E-05	0,00070533	882,92351	-1,89261288	3,91043427	9,21E-05	0,00136476	-
SUSD2	chr22:24577443-24585074	20,4706	6,13492	-1,73844	-2,94234	5,00E-05	0,00070533	975,790552	-1,313301609	3,18249362	0,00146013	0,01143877	-
SUSD4	chr1:223394160-223537544	7,71846	2,00638	-1,94372	-2,49605	5,00E-05	0,00070533	328,442971	-1,531619375	3,91356741	9,09E-05	0,00135202	-
SV2B	chr15:91643181-91844539	6,95847	1,88621	-1,88328	-3,3849	5,00E-05	0,00070533	1176,69642	-1,563783549	4,10034101	4,13E-05	0,00073668	Neuron
SV2C	chr5:75379304-75621416	38,3882	3,9139	-3,29398	-4,91477	5,00E-05	0,00070533	1212,41062	-2,096307346	4,33575	1,45E-05	0,00031653	-
SVOP	chr12:109304654-109459045	4,60337	0,990054	-2,21711	-2,58414	5,00E-05	0,00070533	219,65263	-1,573359457	3,54234584	0,00039659	0,00427339	-
SYK	chr9:93563961-93660842	3,49867	8,15994	1,22175	2,23706	5,00E-05	0,00070533	921,357518	1,079682388	-3,3885679	0,00070259	0,00652763	-
SYN2	chr3:12045833-12233532	13,4298	3,14344	-2,09502	-2,11682	0,00125	0,0111709	739,133301	-1,630389868	3,97078392	7,16E-05	0,00113447	-
SYNDIG1L	chr14:74872595-74892805	5,17948	0,803248	-2,68889	-3,05905	5,00E-05	0,00070533	170,901378	-2,010891205	4,83889478	1,31E-06	4,79E-05	-
SYNGR3	chr16:2039945-2044276	22,0731	5,93732	-1,8944	-3,04463	5,00E-05	0,00070533	490,094267	-1,429923101	3,39328136	0,00069061	0,00645119	-
SYP	chrX:49044264-49058913	62,7363	23,1595	-1,4377	-2,87541	5,00E-05	0,00070533	2238,67897	-1,175257345	3,19892902	0,00137939	0,01100261	-
SYT12	chr11:66790189-66818334	5,73524	1,17038	-2,29288	-3,1842	5,00E-05	0,00070533	280,474797	-1,930413403	6,4178793	1,38E-10	2,40E-08	-
SYT13	chr11:45261852-45307884	5,1387	1,18278	-2,11922	-3,05831	5,00E-05	0,00070533	377,276999	-1,605909162	3,80734899	0,00014046	0,00186662	-
SYT2	chr1:202559724-202679551	33,9228	3,92306	-3,11221	-5,08862	5,00E-05	0,00070533	3360,48466	-1,954021437	4,00571548	6,18E-05	0,00101662	-
SYT3	chr19:51125233-51143092	11,4916	1,38242	-3,05532	-3,97841	5,00E-05	0,00070533	331,476137	-2,272833829	5,42856859	5,68E-08	3,62E-06	-
SYT4	chr18:40847856-40857615	19,0189	3,86007	-2,30074	-3,75212	5,00E-05	0,00070533	1121,81403	-1,712521548	3,87024712	0,00010873	0,00154144	Neuron
SYT7	chr11:61281187-61348344	1,4393	0,537035	-1,42228	-1,79584	0,0001	0,00129637	151,970366	-1,152386812	3,49831626	0,00046821	0,00485011	-
TACR1	chr2:75273589-75426645	2,70602	0,60974	-2,1499	-2,0357	5,00E-05	0,00070533	179,109548	-1,70089404	3,80115273	0,00014402	0,00190144	-
TAGLN	chr11:117049938-117075508	119,954	326,807	1,44595	1,94474	0,00085	0,00810031	6514,13663	1,134923811	-2,7563483	0,00584507	0,03222363	-
TAGLN2	chr1:159887896-159895332	43,6525	92,5473	1,08413	2,29681	5,00E-05	0,00070533	2154,07667	1,01579079	-5,2850459	1,26E-07	7,07E-06	-
TAGLN3	chr3:111717585-111732735	49,1403	11,7759	-2,06107	-3,55501	5,00E-05	0,00070533	835,080891	-1,640625515	4,46100506	8,16E-06	0,00020329	-
TBC1D30	chr12:65218351-65274798	3,03574	1,2358	-1,29661	-2,07153	5,00E-05	0,00070533	429,810062	-1,073350339	3,09487419	0,00196896	0,01436181	-
TBX15	chr1:119425665-119532179	0,151789	2,17814	3,84295	3,35741	5,00E-05	0,00070533	131,141286	2,257622469	-4,6065376	4,09E-06	0,00011837	-
TBXAS1	chr7:139478046-139720125	5,33867	14,3718	1,42869	2,42829	5,00E-05	0,00070533	590,428888	1,302768391	-5,1377135	2,78E-07	1,35E-05	-
TEAD4	chr12:3068477-3149842	0,982006	2,92326	1,57377	1,72215	0,00025	0,00289273	105,488669	1,142552146	-2,8045323	0,00503896	0,02905254	-
TF	chr3:133464976-133497850	1013,74	407,427	-1,31508	-2,10453	5,00E-05	0,00070533	54487,1429	-1,241351167	5,4312878	5,59E-08	3,59E-06	Oligodendroc
TFEC	chr7:115575201-115670867	1,73309	5,20561	1,58672	2,68459	5,00E-05	0,00070533	623,622695	1,440898494	-4,8908351	1,00E-06	3,82E-05	-
TGM2	chr20:36756863-36793700	16,432	64,0409	1,96249	3,62701	5,00E-05	0,00070533	4000,02918	1,536426154	-3,9021405	9,53E-05	0,0014024	-
THSD4	chr15:71433787-72075722	5,72698	17,4562	1,6079	3,03747	5,00E-05	0,00070533	2654,32561	1,289500302	-3,2776244	0,00104685	0,00879905	-
THY1	chr11:119252487-119369944	86,5186	14,172	-2,60996	-4,51806	5,00E-05	0,00070533	2616,65591	-1,948978012	4,47263246	7,73E-06	0,00019467	-
TIMP1	chrX:47420498-47479256	37,7137	100,795	1,41826	1,69598	0,00045	0,00473416	1387,93038	1,281560614	-4,55002	5,36E-06	0,00014641	-
TLR1	chr4:38797875-38806412	5,04258	11,1946	1,15057	2,08991	5,00E-05	0,00070533	649,531961	1,061630211	-4,6301011	3,65E-06	0,00010883	-
TLR2	chr4:154605440-154627242	4,20682	17,814	2,08221	3,75707	5,00E-05	0,00070533	978,470679	1,87918781	-7,0155334	2,29E-12	6,57E-10	-
TLR5	chr1:223282747-223316624	1,63696	4,22038	1,36635	2,11225	5,00E-05	0,00070533	364,613326	1,254317584	-5,0151965	5,30E-07	2,30E-05	-
TLR7	chrX:12885201-12908480	2,43407	6,79987	1,48214	2,58537	5,00E-05	0,00070533	640,077362	1,286020678	-3,8433299	0,00012138	0,00167756	-
TLR8	chrX:12920935-12961419	0,456072	1,77469	1,96024	2,20549	5,00E-05	0,00070533	126,131117	1,586196632	-4,0693444	4,71E-05	0,00081547	-
TMEFF2	chr2:192814746-193059644	22,4003	6,66727	-1,74835	-3,07689	5,00E-05	0,00070533	682,962413	-1,546911253	5,23008031	1,69E-07	8,86E-06	-
TMEM119	chr12:108983621-108991894	3,99525	9,47527	1,24588	2,01194	5,00E-05	0,00070533	416,427844	1,071111518	-3,0159689	0,0025616	0,01752286	-
TMEM130	chr7:98444110-98467673	27,6918	5,80008	-2,25531	-3,79349	5,00E-05	0,00070533	1113,42959	-1,667011801	3,81905818	0,00013396	0,00180438	Neuron
TMEM132D	chr12:129556270-130388212	1,74603	0,602938	-1,53399	-1,95036	0,0001	0,00129637	168,958325	-1,247582704	3,46027541	0,00053962	0,00541592	-
TMEM151B	chr6:44238479-44247182	10,5014	4,36532	-1,26642	-2,34716	5,00E-05	0,00070533	679,030228	-1,126278206	3,80684962	0,00014075	0,00186895	-
TMEM154	chr4:153547265-153601317	0,791498	1,73249	1,13019	1,37623	0,0032	0,0241415	120,517775	1,095609354	-4,1242891	3,72E-05	0,0006809	-
TMEM173	chr5:138855112-138862343	5,5663	12,4703	1,16371	1,94181	5,00E-05	0,00070533	482,44274	1,06053609	-3,7549702	0,00017336	0,0021853	-
TMEM178B	chr7:140774031-141180179	10,8939	4,70709	-1,21062	-2,49834	5,00E-05	0,00070533	1956,64567	-1,13708121	4,36264912	1,28E-05	0,00028585	-
TMEM179	chr14:105057200-105071097	14,627	3,79902	-1,94494	-3,10094	5,00E-05	0,00070533	535,366408	-1,534533403	4,16365556	3,13E-05	0,00058913	-
TMEM196	chr7:19758937-19812404	1,47149	0,35424	-2,05448	-1,94716	0,0001	0,00129637	74,5325714	-1,618081017	3,88438352	0,00010259	0,00147831	-
TMEM30B	chr14:61744088-61748530	0,510493	9,32105	4,19053	4,59001	5,00E-05	0,00070533	519,642517	1,834274449	-3,6227026	0,00029154	0,00332557	-
TMEM35	chrX:100333835-100351355	15,259	3,75588	-2,02244	-2,99817	5,00E-05	0,00070533	420,723521	-1,600811002	3,86322199	0,0001119	0,00157294	-
TMEM59L	chr19:18723681-18731849	40,6616	11,7178	-1,79496	-3,22215	5,00E-05	0,00070533	784,028865	-1,47325339	4,15040647	3,32E-05	0,00062089	-
TNFRSF11B	chr8:119935795-119964383	0,796998	20,5953	4,69159	5,49702	5,00E-05	0,00070533	712,926581	2,535051241	-5,1098965	3,22E-07	1,51E-05	-
TNFRSF1A	chr12:6437922-6451283	16,3209	37,5042	1,20033	2,46354	5,00E-05	0,00070533	1423,7626	1,149373148	-6,6924351	2,19E-11	5,20E-09	-
TNFRSF1B	chr1:12226999-12269277	6,74969	15,2574	1,17662	2,28569	5,00E-05	0,00070533	1102,50501	1,027933233	-4,0941408	4,24E-05	0,00075238	-
TNFSF13B	chr13:108921976-108960832	1,51773	3,57588	1,23638	1,67442	0,0003	0,00336918	186,655241	1,137769285	-4,5569314	5,19E-06	0,00014243	-
TNFSF8	chr9:117655622-117692875	0,573273	1,54238	1,42787	1,67988	0,0001	0,00129637	124,085889	1,278265708	-4,6405465	3,47E-06	0,00010453	-
TNNC1	chr3:52485106-52488057	1,26403	6,73735	2,41415	2,38415	5,00E-05	0,00070533	90,7364617	1,70174468	-3,6210741	0,00029338	0,00333551	-
TNNT2	chr1:201328135-201346828	0,068104	1,44908	4,41126	2,06997	5,00E-05	0,00070533	54,4300604	2,674364639	-5,6597216	1,52E-08	1,26E-06	-
TOP2A	chr17:38544772-38574202	0,102068	0,583659	2,51559	2,04851	0,0003	0,00336918	76,0243677	1,868282765	-4,2252408	2,39E-05	0,00046997	-
TOR4A	chr9:140172279-140177093	0,974912	2,02511	1,05465	1,27515	0,0035	0,0258932	110,427895	1,039192905	-4,4101282	1,03E-05	0,0002435	-
TP63	chr3:189349215-189615068	0,457727	1,58888	1,79545	1,96892	5,00E-05	0,00070533	136,658672	1,462264941	-3,5550748	0,00037787	0,00411037	-

TPM2	chr9:35681989-35690053	28,817	67,2913	1,2235	2,31199	5,00E-05	0,00070533	1943,69942	1,033373466	-2,6812721	0,00733429	0,03812166	-
TPRG1	chr3:188889762-189041271	0,349389	0,895232	1,35743	1,26168	0,0062	0,0408335	66,8216208	1,246018549	-4,1845036	2,86E-05	0,00054757	-
TREM2	chr6:41126243-41130924	11,351	40,5973	1,83857	3,15109	5,00E-05	0,00070533	756,799659	1,505297917	-3,9579639	7,56E-05	0,00118258	-
TRHR	chr8:110099652-110131812	2,14648	0,128522	-4,06189	-2,5082	0,0003	0,00336918	45,4347448	-2,295465856	4,62729769	3,70E-06	0,00010984	-
TRIM22	chr11:5710816-5732093	15,4189	33,6102	1,12419	2,41495	5,00E-05	0,00070533	2232,5611	1,081498299	-8,061503	7,54E-16	5,18E-13	-
TRIM36	chr5:114460458-114516243	19,0581	7,30683	-1,38309	-2,20716	5,00E-05	0,00070533	1396,62746	-1,220793615	3,83825618	0,00012391	0,0017012	-
TRIM58	chr1:248020500-248043438	2,51166	1,01795	-1,30297	-1,72527	5,00E-05	0,00070533	195,012649	-1,123920331	2,98408856	0,00284424	0,01896316	-
TRNP1	chr1:27320194-27327377	11,6153	3,98169	-1,54458	-2,20469	5,00E-05	0,00070533	254,928605	-1,247452045	3,22419876	0,00126326	0,01028702	-
TRPC5	chrX:111017541-111326004	1,81206	0,447066	-2,01907	-2,34164	5,00E-05	0,00070533	159,063587	-1,559778028	3,74009607	0,00018395	0,00228251	-
TRPV4	chr12:110220891-110271241	0,149	0,447229	1,5857	1,22563	0,003	0,0229313	32,7040733	1,664378655	-4,6448857	3,40E-06	0,00010256	-
TSKU	chr11:76493356-76509198	3,26532	10,2357	1,64831	2,43054	5,00E-05	0,00070533	396,059499	1,343726557	-3,2360814	0,00121183	0,00993882	-
TSPAN13	chr7:16793350-16824161	22,2986	5,03286	-2,1475	-3,12736	5,00E-05	0,00070533	454,8358	-1,677971877	4,03727295	5,41E-05	0,00091314	-
TSPAN7	chrX:38420730-38548172	70,9384	32,504	-1,12595	-2,38537	5,00E-05	0,00070533	1910,97729	-1,110494253	4,95187469	7,35E-07	2,94E-05	-
TTC39A	chr1:51752929-51810785	3,04113	0,603451	-2,3333	-2,68229	5,00E-05	0,00070533	170,948538	-1,536848413	3,63935458	0,00027332	0,00315967	-
TUBA4A	chr2:220110191-220136910	34,4381	9,02514	-1,93198	-2,74888	5,00E-05	0,00070533	1050,95465	-1,480326152	3,63621777	0,00027667	0,00318588	-
TUBA8	chr22:18593452-18614498	2,24463	0,701436	-1,67809	-1,8512	5,00E-05	0,00070533	78,9677325	-1,291984076	3,31412784	0,0009193	0,00794722	-
TUBB2A	chr6:3153901-3157783	112,495	42,0449	-1,41985	-2,90047	5,00E-05	0,00070533	2415,71352	-1,435896808	3,95574466	7,63E-05	0,00118911	-
TUBB3	chr16:89988416-90002505	237,507	40,2813	-2,55979	-4,40022	5,00E-05	0,00070533	5127,41159	-1,978192177	4,8777997	1,07E-06	4,05E-05	Neuron
TUNAR	chr14:96343108-96391908	1,68151	0,499689	-1,75065	-1,7593	0,00055	0,00564481	78,5059678	-1,382526876	3,45536863	0,00054954	0,00549408	-
TYROBP	chr19:36395302-36399211	16,1591	53,8265	1,73597	2,69457	5,00E-05	0,00070533	439,493447	1,489940016	-4,5081901	6,54E-06	0,0001721	-
UBE2O	chr17:74385612-74449288	14,8385	6,47271	-1,1969	-2,42611	5,00E-05	0,00070533	1573,15584	-1,018836233	3,67995475	0,00023328	0,00277443	-
UBE2QL1	chr5:6448735-6496834	3,83521	0,642724	-2,57704	-3,48581	5,00E-05	0,00070533	318,803267	-2,041303332	4,94549718	7,59E-07	3,03E-05	-
UCHL1	chr4:41258897-41270446	398,901	109,355	-1,867	-3,51738	5,00E-05	0,00070533	6881,06298	-1,520771832	3,99825747	6,38E-05	0,00104007	-
UHRF1	chr19:4903091-4962165	0,406255	1,08358	1,41535	1,42072	0,00465	0,0325248	94,0264458	1,341788635	-3,9190459	8,89E-05	0,00133028	-
UNC13A	chr19:17712136-17799008	3,87494	1,35031	-1,52088	-2,80768	5,00E-05	0,00070533	767,164694	-1,318400235	5,07014388	3,98E-07	1,82E-05	Neuron
UNC13C	chr15:54305100-54920806	5,28577	2,18955	-1,27148	-2,43801	5,00E-05	0,00070533	942,221565	-1,187296618	4,61085834	4,01E-06	0,00011655	-
UNC5A	chr5:176237559-176326333	2,74595	0,750432	-1,87151	-1,91269	0,00045	0,00473416	147,7846	-1,489474204	3,78736683	0,00015225	0,00198379	-
UNC79	chr14:93799564-94173689	4,45023	2,14354	-1,05388	-2,01031	5,00E-05	0,00070533	973,361143	-1,011460682	5,06093265	4,17E-07	1,89E-05	-
UPK1B	chr3:118892424-118924000	0,0317167	3,39562	6,74229	3,58653	0,0029	0,0223705	104,095864	2,255264765	-4,4569322	8,31E-06	0,0002062	-
VAMP1	chr12:6561176-6579843	133,788	18,8139	-2,83008	-4,54757	5,00E-05	0,00070533	4424,9285	-2,143576972	5,12065618	3,04E-07	1,45E-05	-
VAMP8	chr2:85804613-85809156	7,40727	19,6102	1,40459	2,03753	5,00E-05	0,00070533	257,396405	1,260422257	-4,3094013	1,64E-05	0,00034504	-
VAT1L	chr16:77822482-78014001	92,2157	16,2857	-2,50141	-4,32479	5,00E-05	0,00070533	4851,54873	-2,015031064	5,08162851	3,74E-07	1,72E-05	-
VIM	chr10:17256237-17279592	301,443	668,842	1,14978	2,02258	5,00E-05	0,00070533	29981,4895	1,078775524	-4,0269005	5,65E-05	0,00094585	-
VNN1	chr6:133001996-133035194	0,185309	0,703858	1,92535	1,57729	0,0023	0,018498	53,0916065	1,659826355	-4,9183634	8,73E-07	3,38E-05	-
VNN2	chr6:133065008-133084598	0,764833	2,55211	1,73847	1,8131	5,00E-05	0,00070533	100,315482	1,605017616	-6,3032525	2,91E-10	4,70E-08	-
VSIG4	chrX:65241579-65259967	3,17834	21,4405	2,75399	4,3237	5,00E-05	0,00070533	674,095954	2,468859256	-8,2626613	1,42E-16	1,22E-13	-
VSNL1	chr2:17721806-17837706	38,233	8,08046	-2,24231	-3,72082	5,00E-05	0,00070533	1017,27168	-1,479420193	3,07263081	0,00212181	0,01520789	Neuron
VSTM2A	chr7:54610018-54639419	6,8928	1,17317	-2,55468	-2,32148	0,0001	0,00129637	154,847719	-1,939425095	4,61371174	3,96E-06	0,00011519	-
VSTM2B	chr19:30017490-30055226	11,5918	4,99018	-1,21594	-1,74927	0,0001	0,00129637	339,901561	-1,063804924	3,89379326	9,87E-05	0,00143412	-
VSTM2L	chr20:36531498-36573747	4,85441	1,52737	-1,66825	-1,86858	0,00015	0,00184356	109,434355	-1,316195887	3,51053404	0,00044721	0,00468433	-
VSTM5	chr11:93553734-93583668	2,10254	0,448364	-2,22939	-1,41952	0,0069	0,044553	24,8362884	-1,599361299	3,41578283	0,00063599	0,00610828	-
VWA5B2	chr3:183948316-183967313	4,18819	0,908373	-2,20497	-1,99662	0,0005	0,00518549	230,821914	-1,810919415	5,24885001	1,53E-07	8,14E-06	-
WAS	chrX:48542185-48549817	1,64897	3,7449	1,18336	1,41422	0,00155	0,0134097	160,301248	1,00258921	-3,6099104	0,0003063	0,00346902	-
WISP1	chr8:134203281-134243932	0,916992	2,20426	1,26532	1,73095	0,0002	0,0023747	198,483686	1,094162387	-3,284657	0,00102107	0,00862805	-
WISP2	chr20:43343884-43356452	0,957686	6,54961	2,77378	2,29269	5,00E-05	0,00070533	113,790931	1,828504142	-3,8027255	0,00014311	0,00189425	-
WNT7A	chr3:13860081-13921618	5,41811	1,19256	-2,18372	-2,52571	5,00E-05	0,00070533	130,655106	-1,97951097	8,55467817	1,18E-17	1,18E-14	-
YBX3	chr12:10851675-10875953	11,4237	34,907	1,61149	2,89699	5,00E-05	0,00070533	1437,90107	1,382340025	-4,3186975	1,57E-05	0,00033527	-
YBX3P1	chr16:31579087-31580845	0,708315	2,67715	1,91824	1,8389	0,0004	0,00431348	96,2366831	1,65298564	-4,0895181	4,32E-05	0,00076369	-
YWHAG	chr7:75956107-75988342	227,039	70,6636	-1,6839	-2,85758	5,00E-05	0,00070533	11883,7019	-1,468579691	4,23525227	2,28E-05	0,00045428	-
ZBTB16	chr11:113930430-114121397	16,1013	46,8398	1,54056	3,19406	5,00E-05	0,00070533	2077,88625	1,490297936	-6,5485437	5,81E-11	1,16E-08	-
ZC3H12D	chr6:149768765-149806148	0,303037	0,721973	1,25245	1,23399	0,0029	0,0223705	61,6081309	1,131659454	-4,2402959	2,23E-05	0,00044634	-
ZMAT4	chr8:40388110-40755343	2,06365	0,397631	-2,37569	-2,33532	5,00E-05	0,00070533	79,1148794	-1,438008464	3,27810127	0,00104508	0,00878778	-
ZNF366	chr5:71739233-71803249	1,27067	3,45088	1,44137	1,86374	0,0001	0,00129637	212,463063	1,202285371	-3,2178576	0,00129152	0,01047583	-
ZNF804A	chr2:185463092-185804214	1,81896	0,551067	-1,72281	-2,07435	5,00E-05	0,00070533	154,192817	-1,243572209	2,81347729	0,00490089	0,02843919	-
ZSCAN31	chr6:28292514-28304152	10,1912	4,07543	-1,3223	-2,20977	5,00E-05	0,00070533	583,336481	-1,207592944	3,71798456	0,00020082	0,00245346	-

Supplementary Table 3

Legend:

**Term**	GO term - Biological Process
**P-value**	P value computed using a standard statistical method: Fisher's exact test or the hypergeometric test.
**Adjusted P-v**	Adjusted p-value using the Benjamini-Hochberg method for correction for multiple hypotheses testing
**Z-score**	Score computed using a modification to Fisher's exact test for deviation from an expected rank.
**Combined Sc**	Is a combination of the p-value and z-score calculated by multiplying the two scores as follows: c = log(p) * z, where p is the p value and z the z-score
**Genes**	List of genes related to the GO term

GO Biological Process - UP Genes (Pval < 0,05)

Term	P-value	Adjusted P-value	Z-score	Combined Score	Genes
inflammatory response (GO:0006954)	3,2369E-29	1,09149E-25	-2,3823266	136,9291905	[illegible]
activation of immune response (GO:0002253)	2,0978E-17	1,76843E-14	-3,4536108	109,3623892	[illegible]
regulation of cytokine production (GO:0001817)	1,337E-17	1,50282E-14	-2,5058637	79,75875748	[illegible]
regulation of inflammatory response (GO:00507	9,4101E-18	1,50282E-14	-2,4297828	77,33719153	[illegible]
positive regulation of defense response (GO:003	4,1506E-17	2,79917E-14	-2,4280398	75,77151754	[illegible]
cellular response to cytokine stimulus (GO:00713	1,0806E-16	5,21992E-14	-2,4532707	75,03012017	[illegible]
regulation of cell activation (GO:0050865)	5,0985E-16	2,14901E-13	-2,512276	73,27957281	[illegible]
regulation of response to wounding (GO:190303	1,2347E-15	3,78487E-13	-2,4676374	70,58083265	[illegible]
regulation of leukocyte activation (GO:0002694)	3,3915E-15	7,78669E-13	-2,4970911	69,62187412	[illegible]
response to molecule of bacterial origin (GO:000	1,5388E-15	3,99153E-13	-2,412436	68,87367394	[illegible]
response to other organism (GO:0051707)	1,0185E-15	3,4344E-13	-2,3902969	68,60095485	[illegible]
leukocyte activation (GO:0045321)	6,5701E-16	2,46161E-13	-2,3591027	68,49133888	[illegible]
myeloid leukocyte activation (GO:0002274)	1,0836E-16	5,21992E-14	-2,2332762	68,30187127	[illegible]
response to lipopolysaccharide (GO:0032496)	1,3596E-15	3,82045E-13	-2,3798901	68,04876325	[illegible]
regulation of immune effector process (GO:0002	3,4638E-15	7,78669E-13	-2,4342581	67,87001296	[illegible]
defense response to other organism (GO:009854	4,6699E-15	9,84183E-13	-2,3304978	64,43119093	[illegible]
leukocyte migration (GO:0050900)	4,2048E-14	8,3403E-12	-2,230298	56,89472931	[illegible]
secretion by cell (GO:0032940)	6,045E-13	9,7065E-11	-2,3687084	54,61208867	[illegible]
cytokine-mediated signaling pathway (GO:00192	4,4683E-13	8,37064E-11	-2,3530357	54,59914927	[illegible]
taxis (GO:0042330)	5,4857E-13	9,24889E-11	-2,3575787	54,46933709	[illegible]
chemotaxis (GO:0006935)	5,4857E-13	9,24889E-11	-2,3549327	54,40820551	[illegible]
immune response-activating signal transduction	2,8574E-09	1,78428E-07	-3,4319142	53,32878814	[illegible]
regulation of lymphocyte proliferation (GO:0050	1,7314E-12	2,65379E-10	-2,3556835	51,94250075	[illegible]
regulation of mononuclear cell proliferation (GO:	1,9894E-12	2,91657E-10	-2,3544141	51,6922031	[illegible]
positive regulation of response to external stimu	2,516E-12	3,39362E-10	-2,3345561	50,90254927	[illegible]
regulation of leukocyte proliferation (GO:007066	3,9228E-12	5,08758E-10	-2,3483553	50,252568	[illegible]
regulation of leukocyte mediated immunity (GO:	2,1657E-12	3,04276E-10	-2,2765408	49,88603246	[illegible]
regulation of lymphocyte activation (GO:005124	1,0882E-11	1,3031E-09	-2,4348826	49,81408638	[illegible]
positive regulation of cytokine production (GO:0	1,1207E-11	1,3031E-09	-2,3820947	48,73412456	[illegible]
regulation of adaptive immune response (GO:00	4,3742E-12	5,46294E-10	-2,2558306	48,11204841	[illegible]
positive regulation of cell activation (GO:005086	3,4874E-11	3,67481E-09	-2,366847	45,96834646	[illegible]
regulation of T cell activation (GO:0050863)	4,8476E-11	4,95334E-09	-2,3776165	45,46764311	[illegible]
positive regulation of immune effector process (	1,8356E-11	2,06326E-09	-2,2612173	45,22203623	[illegible]
positive regulation of leukocyte activation (GO:0	6,4664E-11	6,22989E-09	-2,3624733	44,63634964	[illegible]
myeloid cell activation involved in immune respo	9,3871E-11	8,79259E-09	-2,3340425	43,29498825	[illegible]
positive regulation of inflammatory response (G	3,2548E-11	3,5404E-09	-2,2057096	42,92096048	[illegible]
regulation of adaptive immune response based c	6,2084E-11	6,15731E-09	-2,2243698	42,05310211	[illegible]
macrophage activation (GO:0042116)	9,5369E-10	6,84224E-08	-2,5419086	41,93530333	[illegible]
negative regulation of immune system process (	2,414E-10	2,1421E-08	-2,3654075	41,77048385	[illegible]
regulation of myeloid leukocyte mediated immu	6,0188E-10	4,71983E-08	-2,4078146	40,61720475	[illegible]
activation of innate immune response (GO:0002	2,3625E-10	2,1421E-08	-2,1747686	38,40401101	[illegible]
regulation of T cell proliferation (GO:0042129)	2,8266E-10	2,32467E-08	-2,1815099	38,3446255	[illegible]
regulation of innate immune response (GO:0045	6,8E-10	5,21127E-08	-2,2797373	38,23086744	[illegible]
cellular response to molecule of bacterial origin (	5,2308E-10	4,19958E-08	-2,2165007	37,64880582	[illegible]
positive regulation of innate immune response (	7,2869E-10	5,46029E-08	-2,2369535	37,40897167	[illegible]
cell activation involved in immune response (GO:	2,808E-10	2,32467E-08	-2,1259027	37,36721199	[illegible]
leukocyte activation involved in immune respons	2,808E-10	2,32467E-08	-2,1227314	37,31146943	[illegible]
cellular response to biotic stimulus (GO:0071216	7,6665E-10	5,6199E-08	-2,1998236	36,72466055	[illegible]
cellular response to lipopolysaccharide (GO:0071	1,5178E-09	1,06623E-07	-2,1726521	34,87969094	[illegible]
positive regulation of lymphocyte activation (GO	5,2408E-09	3,04692E-07	-2,2973825	34,46984476	[illegible]
response to bacterium (GO:0009617)	2,3034E-09	1,52292E-07	-2,1916558	34,40343816	[illegible]
positive regulation of T cell activation (GO:00508	3,4348E-09	2,10584E-07	-2,2041161	33,88471907	[illegible]
regulation of leukocyte differentiation (GO:1902	5,5573E-09	3,17616E-07	-2,2115546	33,0902163	[illegible]
positive regulation of response to wounding (GO	3,7834E-09	2,27816E-07	-2,1359472	32,66872971	[illegible]
regulation of alpha-beta T cell activation (GO:00	1,6038E-09	1,10369E-07	-2,0382979	32,65238069	[illegible]
regulation of cytokine secretion (GO:0050707)	5,9438E-09	3,3404E-07	-2,1815464	32,53123141	[illegible]
granulocyte chemotaxis (GO:0071621)	1,6714E-09	1,12716E-07	-2,0268044	32,42560805	[illegible]
regulation of mast cell degranulation (GO:00433	1,2114E-08	6,48377E-07	-2,275548	32,4238116	[illegible]
leukocyte chemotaxis (GO:0030595)	2,3644E-09	1,53323E-07	-2,0537562	32,22490823	[illegible]
regulation of protein transport (GO:0051223)	3,1165E-08	1,38475E-06	-2,3713401	31,98935886	[illegible]
granulocyte migration (GO:0097530)	2,7388E-09	1,74251E-07	-1,9945348	31,04048385	[illegible]
regulation of leukocyte migration (GO:0002685)	1,0598E-08	5,76416E-07	-2,1604405	31,03783042	[illegible]
coagulation (GO:0050817)	1,8585E-08	9,35376E-07	-2,2180392	30,79152385	[illegible]
blood coagulation (GO:0007596)	1,8585E-08	9,35376E-07	-2,2156064	30,75775046	[illegible]
adaptive immune response (GO:0002250)	4,2087E-09	2,48975E-07	-2,0171286	30,67228188	[illegible]
regulation of mast cell activation involved in imm	2,0413E-08	9,83307E-07	-2,202167	30,4611316	[illegible]
hemostasis (GO:0007599)	2,5845E-08	1,21039E-06	-2,2022217	30,00432333	[illegible]
B cell receptor signaling pathway (GO:0050853)	1,3931E-08	7,22718E-07	-2,062914	29,17011316	[illegible]
regulation of apoptotic signaling pathway (GO:2	8,8088E-08	3,22861E-06	-2,3034286	29,12330809	[illegible]
regulation of mast cell activation (GO:0033003)	2,9198E-08	1,33047E-06	-2,1482213	29,06539105	[illegible]
lymphocyte activation (GO:0046649)	3,6785E-08	1,57011E-06	-2,1330055	28,50626921	[illegible]
negative regulation of cell activation (GO:005086	4,2176E-08	1,74598E-06	-2,1323791	28,27149953	[illegible]
defense response to bacterium (GO:0042742)	3,1621E-08	1,38475E-06	-2,0901347	28,19590097	[illegible]
negative regulation of immune response (GO:00	2,6627E-08	1,22993E-06	-2,0484406	27,87630756	[illegible]
icosanoid metabolic process (GO:0006690)	1,9932E-08	9,74059E-07	-2,001731	27,70754858	[illegible]
fatty acid derivative metabolic process (GO:1901	1,9932E-08	9,74059E-07	-1,9984214	27,66173831	[illegible]
positive regulation of leukocyte proliferation (GC	3,5472E-08	1,53348E-06	-2,0480736	27,41954848	[illegible]
regulation of regulated secretory pathway (GO:1	8,2698E-08	3,06437E-06	-2,1479104	27,26916041	[illegible]
neutrophil chemotaxis (GO:0030593)	2,4503E-08	1,16372E-06	-1,9874307	27,15602811	[illegible]
positive regulation of protein transport (GO:005	1,5136E-07	5,10392E-06	-2,2112859	26,94562918	[illegible]
regulation of lymphocyte mediated immunity (G	5,7911E-08	2,29735E-06	-2,0618706	26,77081777	[illegible]
neutrophil migration (GO:1990266)	3,1619E-08	1,38475E-06	-1,9503677	26,31044503	[illegible]
regulation of tumor necrosis factor superfamily c	4,1824E-08	1,74598E-06	-1,9808358	26,26230901	[illegible]
cellular response to lipid (GO:0071396)	2,7804E-07	8,44647E-06	-2,246135	26,23881409	[illegible]
regulation of protein secretion (GO:0050708)	1,301E-07	4,57972E-06	-2,1305227	26,19237385	[illegible]
negative regulation of adaptive immune respons	8,2698E-08	3,06437E-06	-2,0584676	26,13362374	[illegible]
cell chemotaxis (GO:0060326)	7,0628E-08	2,70635E-06	-2,0343178	26,07976708	[illegible]
positive regulation of myeloid leukocyte mediate	9,0035E-09	4,97699E-07	-1,7843719	25,89707072	[illegible]
response to interferon-gamma (GO:0034341)	5,3057E-08	2,12985E-06	-1,9800717	25,85866617	[illegible]
response to virus (GO:0009615)	1,6154E-07	5,28858E-06	-2,1180386	25,73408562	[illegible]
cytokine production (GO:0001816)	4,2459E-08	1,74598E-06	-1,9253793	25,52705629	[illegible]
regulation of interleukin-6 production (GO:0032	7,9436E-08	3,00966E-06	-2,0014484	25,44578469	[illegible]
innate immune response-activating signal transd	1,1537E-07	4,18326E-06	-2,043663	25,30957897	[illegible]
positive regulation of cell migration (GO:003033	3,5802E-07	1,04976E-05	-2,1891996	25,09777495	[illegible]
adaptive immune response based on somatic rec	4,5419E-08	1,84521E-06	-1,883592	24,86890497	[illegible]
positive regulation of alpha-beta T cell activation	6,5236E-08	2,55787E-06	-1,922663	24,75685579	[illegible]
positive regulation of lymphocyte proliferation (	1,3038E-07	4,57972E-06	-2,0011015	24,60128556	[illegible]
negative regulation of lymphocyte activation (GC	1,5288E-07	5,10423E-06	-2,0176236	24,58563365	[illegible]
positive regulation of mononuclear cell prolifera	1,474E-07	5,02051E-06	-1,9925588	24,31316049	[illegible]
positive regulation of leukocyte differentiation (	1,8762E-07	6,02521E-06	-2,0019875	24,06300648	[illegible]
positive regulation of cell motility (GO:2000147)	5,688E-07	1,58512E-05	-2,1753363	24,04240108	[illegible]
unsaturated fatty acid metabolic process (GO:00	1,1689E-07	4,19312E-06	-1,94136	24,03804661	[illegible]
aging (GO:0007568)	3,0049E-07	8,96676E-06	-2,0612696	23,956048	[illegible]
negative regulation of leukocyte activation (GO:	2,3756E-07	7,34899E-06	-2,021305	23,89373932	[illegible]
regulation of leukocyte degranulation (GO:0043	2,5185E-07	7,7205E-06	-2,019251	23,76987964	[illegible]
negative regulation of interleukin-12 production	6,7989E-08	2,63516E-06	-1,8353002	23,57730499	[illegible]
regulation of establishment of protein localizatio	1,0067E-06	2,63308E-05	-2,2323904	23,54004953	[illegible]
positive regulation of lymphocyte differentiation	1,433E-07	4,98159E-06	-1,9274591	23,53381594	[illegible]
myeloid leukocyte migration (GO:0097529)	1,6851E-07	5,46376E-06	-1,9108416	23,15438125	[illegible]
positive regulation of leukocyte migration (GO:0	2,3118E-07	7,21808E-06	-1,9501639	23,0878375	[illegible]
positive regulation of cellular component moven	1,0073E-06	2,63308E-05	-2,1711495	22,894278	[illegible]
platelet activation (GO:0030168)	3,2621E-07	9,64893E-06	-1,9731844	22,78764182	[illegible]
positive regulation of T cell differentiation (GO:0	1,471E-07	5,02051E-06	-1,8622205	22,7227751	[illegible]
regulation of phagocytosis (GO:0050764)	1,5761E-07	5,21031E-06	-1,852788	22,53892763	[illegible]
toll-like receptor signaling pathway (GO:0002224	2,9926E-07	8,96676E-06	-1,9381037	22,52461472	[illegible]
regulation of tumor necrosis factor production (	2,3118E-07	7,21808E-06	-1,8862097	22,33068884	[illegible]
pattern recognition receptor signaling pathway (	4,8379E-07	1,39432E-05	-1,985947	22,20392243	[illegible]
immune response-activating cell surface recepto	4,5279E-05	0,000658102	-2,9918666	21,91886541	[illegible]
exocytosis (GO:0006887)	7,0174E-07	1,9238E-05	-1,9896666	21,60504185	[illegible]
positive regulation of locomotion (GO:0040017)	1,6391E-06	4,09419E-05	-2,1335966	21,55648759	[illegible]
T cell activation (GO:0042110)	7,7678E-07	2,11235E-05	-1,9782708	21,29633184	[illegible]
negative regulation of adaptive immune respons	4,9207E-07	1,39433E-05	-1,877821	20,99500274	[illegible]
negative regulation of cellular component mover	9,6322E-07	2,55746E-05	-1,9849914	20,98912043	[illegible]
integrin-mediated signaling pathway (GO:00072	5,6301E-07	1,58206E-05	-1,8916433	20,91060418	[illegible]
lipopolysaccharide-mediated signaling pathway (	9,0554E-07	2,42339E-05	-1,9445238	20,6659283	[illegible]
positive regulation of T cell proliferation (GO:004	4,8817E-07	1,39433E-05	-1,8459937	20,63915739	[illegible]
endocytosis (GO:0006897)	1,5194E-06	3,88145E-05	-2,029692	20,61500823	[illegible]
negative regulation of locomotion (GO:0040013)	1,2452E-06	3,20517E-05	-1,9717114	20,40358528	[illegible]
positive regulation of tumor necrosis factor supe	4,4243E-07	1,28609E-05	-1,797828	20,24591404	[illegible]
regulation of cytokine biosynthetic process (GO:	1,8565E-06	4,56841E-05	-1,9559456	19,54725281	[illegible]
regulation of extrinsic apoptotic signaling pathwa	2,8323E-06	6,32492E-05	-2,0003678	19,34041373	[illegible]
lipid transport (GO:0006869)	2,1527E-06	4,94729E-05	-1,9315278	19,14933093	[illegible]
extracellular matrix organization (GO:0030198)	3,4869E-06	7,4416E-05	-1,9868221	18,88641096	[illegible]
extracellular structure organization (GO:0043062	3,6728E-06	7,69226E-05	-1,9814109	18,76933113	[illegible]
regulation of lymphocyte differentiation (GO:004	1,9878E-06	4,6548E-05	-1,8764958	18,7180968	[illegible]
response to tumor necrosis factor (GO:0034612)	2,6365E-06	5,92689E-05	-1,9184785	18,67336828	[illegible]
regulation of B cell activation (GO:0050864)	1,6382E-06	4,09419E-05	-1,8469977	18,66087608	[illegible]
single organismal cell-cell adhesion (GO:0016337	3,0335E-06	6,55695E-05	-1,9340818	18,62984917	[illegible]
regulation of production of molecular mediator c	1,8803E-06	4,56841E-05	-1,8557461	18,54588234	[illegible]
interaction with host (GO:0051701)	1,6382E-06	4,09419E-05	-1,8309604	18,49884613	[illegible]
positive regulation of nucleocytoplasmic transpo	2,1002E-06	4,88409E-05	-1,8622646	18,48659178	[illegible]
regulation of inflammatory response to antigenic	1,0615E-06	2,75333E-05	-1,7565724	18,44421447	[illegible]
regulation of blood circulation (GO:1903522)	4,8872E-06	9,64853E-05	-1,9721407	18,23464935	[illegible]
positive regulation of protein import into nucleu	2,1567E-06	4,94729E-05	-1,8269836	18,11287077	[illegible]
single organism cell adhesion (GO:0098602)	4,0732E-06	8,37483E-05	-1,9288062	18,10704457	[illegible]
negative regulation of immune effector process (	2,4684E-06	5,58625E-05	-1,8473219	18,09011728	[illegible]
regulation of alpha-beta T cell differentiation (GC	1,9111E-06	4,56841E-05	-1,8055427	18,04416182	[illegible]
regulation of endocytosis (GO:0030100)	3,9641E-06	8,20062E-05	-1,9021944	17,89720643	[illegible]
regulation of vesicle-mediated transport (GO:00	5,9709E-06	0,000113111	-1,9582082	17,79451726	[illegible]
lymphocyte mediated immunity (GO:0002449)	1,7753E-06	4,40176E-05	-1,7529564	17,58376538	[illegible]
negative regulation of cell migration (GO:003033	4,3042E-06	8,63907E-05	-1,8647522	17,44779808	[illegible]
defense response to virus (GO:0051607)	3,7238E-06	7,75107E-05	-1,8400185	17,41594976	[illegible]
response to acid chemical (GO:0001101)	1,0626E-05	0,000188582	-2,0062766	17,20578118	[illegible]
regulation of T cell differentiation (GO:0045580)	3,0108E-06	6,54991E-05	-1,7838313	17,18449229	[illegible]
regulation of cell adhesion (GO:0030155)	9,6175E-06	0,000173424	-1,9791171	17,13869959	[illegible]
immune response-regulating cell surface recepto	0,00014443	0,001725052	-2,6878779	17,10161763	[illegible]
regulation of acute inflammatory response (GO:	1,9675E-06	4,6395E-05	-1,7131536	17,09439154	[illegible]
regulation of peptidyl-tyrosine phosphorylation (	6,3345E-06	0,00011801	-1,8830481	17,03168278	[illegible]
positive regulation of protein localization to nucl	3,6548E-06	7,69226E-05	-1,7943658	16,99750712	[illegible]
platelet degranulation (GO:0002576)	2,4684E-06	5,58625E-05	-1,7329175	16,96979931	[illegible]
negative regulation of leukocyte mediated immu	3,3865E-06	7,27345E-05	-1,7723872	16,88853593	[illegible]
negative regulation of cell motility (GO:2000146)	5,942E-06	0,000113111	-1,8524324	16,83331772	[illegible]
artery morphogenesis (GO:0048844)	4,2749E-06	8,63907E-05	-1,7889969	16,73898379	[illegible]
positive regulation of intracellular transport (GO:	1,1919E-05	0,000206273	-1,9623744	16,65331535	[illegible]
regulation of T cell mediated immunity (GO:0002	4,2749E-06	8,63907E-05	-1,7745886	16,60416994	[illegible]
leukocyte differentiation (GO:0002521)	8,4905E-06	0,000155598	-1,8856537	16,53385201	[illegible]
negative regulation of T cell activation (GO:0050	5,4636E-06	0,000104677	-1,7604873	16,13422274	[illegible]
cellular response to tumor necrosis factor (GO:0	6,7645E-06	0,000124644	-1,7884822	16,07853988	[illegible]
positive regulation of tumor necrosis factor prod	2,8911E-06	6,41358E-05	-1,6635806	16,06105201	[illegible]
positive regulation of adaptive immune response	6,2578E-06	0,000117229	-1,7625135	15,95317891	[illegible]
blood vessel morphogenesis (GO:0048514)	6,2578E-06	0,000117229	-1,7466763	15,80983017	[illegible]
organic hydroxy compound transport (GO:00158	5,3196E-06	0,000103091	-1,7177652	15,76891152	[illegible]
leukocyte mediated immunity (GO:0002443)	3,5833E-06	7,59929E-05	-1,6623286	15,76697196	[illegible]
positive regulation of cytokine biosynthetic proc	4,9216E-06	9,64853E-05	-1,6851872	15,58144278	[illegible]
regulation of chemokine production (GO:003264	3,007E-06	6,54991E-05	-1,6113114	15,52252642	[illegible]
regulation of cell adhesion mediated by integrin	5,3731E-06	0,000103531	-1,6869187	15,4785503	[illegible]
antigen processing and presentation of peptide	6,6126E-06	0,000122514	-1,7043971	15,35198621	[illegible]
positive regulation of alpha-beta T cell differenti	4,2832E-06	8,63907E-05	-1,6198467	15,15630748	[illegible]
positive regulation of leukocyte mediated immur	1,0529E-05	0,000187859	-1,754931	15,056987	[illegible]
negative regulation of cytokine production (GO:	1,8228E-05	0,000296932	-1,8416838	14,9581666	[illegible]
positive regulation of intracellular protein transp	1,8425E-05	0,000298692	-1,8186096	14,76001419	[illegible]
skeletal system development (GO:0001501)	1,3005E-05	0,000223747	-1,7473789	14,68670869	[illegible]
acute inflammatory response (GO:0002526)	4,9216E-06	9,64853E-05	-1,5793424	14,60278939	[illegible]
antigen processing and presentation (GO:001988	1,9871E-05	0,00031907	-1,8041997	14,52398861	[illegible]
regulation of interferon-gamma production (GO:	1,1929E-05	0,000206273	-1,6993651	14,42133709	[illegible]
regulation of intracellular transport (GO:003238	5,0871E-05	0,000736208	-1,9565187	14,11432054	[illegible]
positive regulation of acute inflammatory respor	5,3175E-06	0,000103091	-1,5247511	13,99706154	[illegible]
regulation of epithelial cell proliferation (GO:005	4,1546E-05	0,000611758	-1,8692619	13,83099319	[illegible]
negative regulation of lymphocyte proliferation (	1,4451E-05	0,000246112	-1,6413745	13,63936783	[illegible]
macrophage activation involved in immune respo	6,7392E-07	1,86266E-05	-1,2517092	13,632264	SBNO2;TYROBP;SYK;TLR8;TLR7;TLR2
negative regulation of mononuclear cell prolifera	1,4451E-05	0,000246112	-1,6371577	13,6043273	[illegible]
response to fluid shear stress (GO:0034405)	9,1179E-06	0,000166192	-1,5435612	13,43263505	[illegible]
regulation of myeloid cell differentiation (GO:00	3,3064E-05	0,000499964	-1,7481874	13,28792889	[illegible]
positive regulation of MAPK cascade (GO:004341	5,3059E-05	0,000751748	-1,8442175	13,26565758	[illegible]
regulation of peptidase activity (GO:0052547)	5,2669E-05	0,000750989	-1,8399167	13,23658144	[illegible]
regulation of cell growth (GO:0001558)	4,1147E-05	0,000608539	-1,7827379	13,20019152	[illegible]
unsaturated fatty acid biosynthetic process (GO:	1,0426E-05	0,000187	-1,5326834	13,15717157	[illegible]
protein secretion (GO:0009306)	1,5901E-05	0,000266761	-1,5736389	12,9497228	[illegible]
reactive oxygen species metabolic process (GO:0	2,1619E-05	0,00034386	-1,6228331	12,94254205	[illegible]
negative regulation of defense response (GO:00	3,1529E-05	0,000478899	-1,6693392	12,7604646	[illegible]
positive regulation of adaptive immune response	2,6789E-05	0,000418212	-1,6388585	12,74953481	[illegible]
cellular ion homeostasis (GO:0006873)	7,0906E-05	0,000964087	-1,8281591	12,6953384	[illegible]
regulation of leukocyte chemotaxis (GO:0002688	1,8182E-05	0,000296932	-1,5528757	12,61246556	[illegible]
positive regulation of cytokine secretion (GO:005	2,0739E-05	0,000331435	-1,5722342	12,59686445	[illegible]
negative regulation of leukocyte proliferation (G	2,2933E-05	0,00036306	-1,5892873	12,58865502	[illegible]
regulation of granulocyte chemotaxis (GO:00716	1,4935E-05	0,000251796	-1,4914722	12,35966707	[illegible]
regulation of B cell proliferation (GO:0030888)	1,6917E-05	0,000282392	-1,5095374	12,33626185	[illegible]
cellular chemical homeostasis (GO:0055082)	7,9821E-05	0,001051386	-1,7980363	12,33029652	[illegible]
regulation of intrinsic apoptotic signaling pathwa	5,2783E-05	0,000750989	-1,6994229	12,22585188	[illegible]
dendritic cell differentiation (GO:0097028)	1,4935E-05	0,000251796	-1,4711606	12,19134697	CD86;LYN;BATF3;SPI1;TREM2;LILRB1;BATF
fatty acid derivative biosynthetic process (GO:19	1,8084E-05	0,000296932	-1,4987754	12,17306249	[illegible]
icosanoid biosynthetic process (GO:0046456)	1,8084E-05	0,000296932	-1,494923	12,14177364	[illegible]
regulation of leukocyte apoptotic process (GO:2	2,6789E-05	0,000418212	-1,5479469	12,04228584	[illegible]
negative regulation of apoptotic signaling pathw	7,5669E-05	0,001016562	-1,747076	12,03967601	[illegible]
response to wounding (GO:0009611)	7,2733E-05	0,000984966	-1,725336	11,94433554	[illegible]
long-chain fatty acid metabolic process (GO:000	2,7078E-05	0,000420764	-1,5027586	11,68160181	[illegible]
regulation of MAP kinase activity (GO:0043405)	9,1159E-05	0,001182266	-1,7311792	11,66870619	[illegible]
antigen receptor-mediated signaling pathway (G	5,733E-05	0,000798828	-1,6278157	11,61017441	[illegible]
circulatory system process (GO:0003013)	7,3073E-05	0,00098561	-1,6490045	11,41482088	[illegible]
lipoprotein metabolic process (GO:0042157)	2,5853E-05	0,000407368	-1,459137	11,38972172	[illegible]
regulation of system process (GO:0044057)	0,0001329	0,001612024	-1,7672034	11,36358603	[illegible]
response to steroid hormone (GO:0048545)	0,00012242	0,001517664	-1,7505173	11,36187722	[illegible]
arachidonic acid metabolic process (GO:0019369	1,9731E-05	0,000318339	-1,3798909	11,111423	[illegible]
cation homeostasis (GO:0055080)	0,00012905	0,001571014	-1,7186147	11,09543516	[illegible]
respiratory burst (GO:0045730)	2,9273E-06	6,45161E-05	-1,141022	11,00925894	HCK;NCF2;SLC11A1;CYBB;CYBA;PIK3CG
positive regulation of cytosolic calcium ion conce	9,5888E-05	0,001229407	-1,6360733	10,96369174	[illegible]
response to protozoan (GO:0001562)	9,2328E-06	0,000167383	-1,2539939	10,90376362	SPN;IL4R;CLEC7A;SLC11A1;CD37;BATF
receptor-mediated endocytosis (GO:0006898)	9,5888E-05	0,001229407	-1,6072317	10,7704177	[illegible]
metal ion homeostasis (GO:0055065)	0,00015519	0,001803145	-1,7031668	10,76098758	[illegible]
regulation of endopeptidase activity (GO:005254	0,0001711	0,001955755	-1,6918001	10,55172182	[illegible]
positive regulation of protein processing (GO:00	0,0001508	0,001784207	-1,6495896	10,43989131	[illegible]
positive regulation of protein maturation (GO:19	0,0001508	0,001784207	-1,6469709	10,42331827	[illegible]
regulation of cytokine production involved in imr	5,8194E-05	0,000807527	-1,4507823	10,33179675	[illegible]
regulation of hypersensitivity (GO:0002883)	1,2395E-08	6,53063E-07	-0,7253147	10,3296357	SPN;C3;FCER1G;IL20RB;FCER1A;FCGR1A
intrinsic apoptotic signaling pathway in response	6,8456E-05	0,000934552	-1,4794074	10,3195229	[illegible]
antigen processing and presentation of exogenou	6,8456E-05	0,000934552	-1,4673371	10,2353277	[illegible]
cellular defense response (GO:0006968)	4,0528E-05	0,000602021	-1,3733525	10,18370845	[illegible]
regulation of acute inflammatory response to an	1,9033E-07	6,05477E-06	-0,8384057	10,07316215	SPN;C3;FCER1G;IL20RB;FCER1A;FCGR1A
positive regulation of regulated secretory pathw	4,4173E-06	8,81372E-05	-1,0764133	10,05005751	FGR;FCER1G;SYK;ADORA3;RAB27A;FCER1A
cellular metal ion homeostasis (GO:0006875)	0,00022725	0,00248511	-1,6657169	9,990034715	[illegible]
MyD88-dependent toll-like receptor signaling pa	9,4327E-05	0,001218655	-1,4604784	9,799820311	[illegible]
positive regulation of peptidase activity (GO:001	0,00014478	0,001725052	-1,536623	9,77676098	[illegible]
regulation of heart contraction (GO:0008016)	0,00015561	0,001803145	-1,5429894	9,748950611	[illegible]
positive regulation of fat cell differentiation (GO:	7,6279E-05	0,001016656	-1,4097949	9,715230249	[illegible]
cellular cation homeostasis (GO:0030003)	0,0002695	0,002838751	-1,6355203	9,591330909	[illegible]
regulation of protein import into nucleus (GO:00	0,00019239	0,002133995	-1,5558447	9,568071107	[illegible]
response to alcohol (GO:0097305)	0,00027431	0,002872546	-1,6224899	9,495713877	[illegible]
positive regulation of secretion (GO:0051047)	0,00026193	0,002786211	-1,6086504	9,463807247	[illegible]
regulation of interleukin-10 production (GO:003	4,3703E-05	0,000637952	-1,2809192	9,424040738	[illegible]
response to inorganic substance (GO:0010035)	0,00032297	0,003330474	-1,6415899	9,364680343	[illegible]
leukotriene metabolic process (GO:0006691)	6,3751E-05	0,000881013	-1,3286878	9,346571821	[illegible]
cell-substrate adhesion (GO:0031589)	0,00015561	0,001803145	-1,4779302	9,337892294	[illegible]
positive regulation of phagocytosis (GO:0050766	4,3703E-05	0,000637952	-1,2663946	9,317179514	[illegible]
response to temperature stimulus (GO:0009266)	0,00016072	0,00185602	-1,4808215	9,313360907	[illegible]

positive regulation of interferon-gamma product	9,0228E-05	0,00117926	-1,3768495	9,283915231 PYCARD;SASH3;SLC11A1;EBI3;IL18;TLR8;TLR7;IL18R1
negative regulation of intracellular signal transdu	0,00030509	0,003165476	-1,611144	9,272861736 LYN;CD74;GPX1;BCL2A1;MYOC;CD300A;IRAK3;IGF1;SOD2;YBX3;ESR1;PYCARD;VNN1;SFRP2;HYAL2;DDIT4;BDKRB2;SNAI2;PTPN6;APOE;FBP1;DACT1
vascular process in circulatory system (GO:00030	0,00014132	0,001695867	-1,4467128	9,22939235 EDN1;HRH1;GPX1;NPR1;NOS3;P2RX1;BDKRB2;ADM;APOE;SOD2
positive regulation of proteolysis (GO:0045862)	0,00024455	0,002617828	-1,5464982	9,194566414 PCOLCE2;FBLN1;NLRC4;TNFRSF1B;MEFV;CTGF;PYCARD;CASP7;DAB2;SFRP2;IFI16;P2RX1;CASP1;CTSH;APOE;S100A9;CTSC
response to metal ion (GO:0010038)	0,00031789	0,003288112	-1,6034195	9,167457633 CD86;TNNC1;MT1L;AQP9;CYBRD1;MT1X;TNFRSF11B;SOD2;AQP3;ASS1;MT2A;FABP4;KRT16;TNNT2;ALOX5AP;CD14;FBP1;S100A9
positive regulation of phosphatidylinositol 3-kina	7,8239E-05	0,00103736	-1,3335603	9,162994953 FGR;MYOC;ADORA3;HGF;HCLS1;PTPN6;IGF1;PIK3AP1
antigen processing and presentation of peptide a	0,00022773	0,00248511	-1,5171454	9,098986278 CD74;FCER1G;ERAP2;NCF2;SLC11A1;NCF4;HLA-C;CYBB;CYBA;IFI30;MR1;CTSS;FCGR1A;SEC24D;FCGR1B
positive regulation of protein secretion (GO:0050	0,00018628	0,002086798	-1,4707099	9,077404787 FGR;PYCARD;RBP4;IL4R;SYK;HYAL2;CLEC5A;CASP1;CD14;ALOX15B;TLR2
cellular response to abiotic stimulus (GO:007121	0,00029992	0,003121379	-1,573177	9,076413682 ADIRF;CYBA;YBX3;TNFRSF1A;SFRP2;IFI16;CASP5;TRPV4;HYAL2;CASP1;SNAI2;TLR8;TLR7;TLR5;MYD88;TP63;ENG
cellular response to mechanical stimulus (GO:007	9,8577E-05	0,0012591	-1,3578538	9,066875741 CASP5;CASP1;TLR8;TLR7;CYBA;TLR5;MYD88;ENG;TNFRSF1A
positive regulation of production of molecular m	9,0228E-05	0,00117926	-1,344026	9,062590616 SASH3;CD74;RBP4;FCER1G;PTPRC;CD37;IL13RA1;TLR2
regulation of neutrophil chemotaxis (GO:009002	3,1229E-05	0,000476492	-1,164524	8,90751339 CD74;EDN1;C5AR2;RAC2;C3AR1;NCKAP1L
regulation of neutrophil migration (GO:1902622)	3,1229E-05	0,000476492	-1,1510499	8,804448765 CD74;EDN1;C5AR2;RAC2;C3AR1;NCKAP1L
regulation of lipid storage (GO:0010883)	7,6279E-05	0,001016656	-1,2619751	8,696568885 C3;ABCA1;MSR1;ITGB3;HILPDA;APOB;OSBPL11
negative regulation of protein phosphorylation ((	0,00040148	0,003901402	-1,5540851	8,619607801 LYN;CD300A;IGFBP3;IRAK3;SMAD6;SAMSN1;GADD45G;PYCARD;SFRP2;FABP4;PTPRC;HYAL2;DDIT4;BDKRB2;PTPN6;SFN;APOE;ENG
response to heat (GO:0009408)	0,00013922	0,00168264	-1,3329157	8,513854648 LYN;ABCC2;NOS3;TRPV4;SLC52A3;MYOF;TFEC;CD14;MKI67
regulation of multi-organism process (GO:00439(	0,00041305	0,004002328	-1,5218401	8,401895251 IFITM3;TOP2A;IFITM1;B4GALT1;IFITM2;CD180;LY86;LY96;LILRB1;IFNLR1;TMEM173;SPN;HCK;PLSCR1;SLPI;IFI16;CD37;TIMP1;MYD88;TLR2
response to interleukin-1 (GO:0070555)	0,00015554	0,001803145	-1,3054713	8,248258398 PYCARD;EDN1;IL1R1;HYAL2;IRAK3;CYBA;APOE;KLF2;MYD88
acute-phase response (GO:0006953)	9,0746E-05	0,001181446	-1,2234614	8,247372739 SERPINA3;CEBPB;PLSCR1;CD163;SERPINA1;SAA2;ASS1
regulation of T cell mediated cytotoxicity (GO:00	5,2242E-05	0,000749624	-1,1197784	8,0578564 PTPRC;RIPK3;HLA-C;LILRB1;IL7R;MR1
negative regulation of lymphocyte mediated imn	5,2242E-05	0,000749624	-1,1190479	8,052600281 SPN;PTPRC;IL20RB;PTPN6;LILRB1;IL7R
response to extracellular stimulus (GO:0009991)	0,00055028	0,00506981	-1,51251	7,992786356 ABCA1;LYN;GPX1;STC1;CYBB;ADM;CYBA;TNFRSF11B;SSTR2;SOD2;AQP3;FSTL1;ASS1;SFRP2;IFI16;KYNU;PDK4;ALPL;APOE;DSC2
regulation of protein localization to nucleus (GO:	0,00045053	0,004279402	-1,448684	7,901038338 IL18;IGF1;TMEM173;TNFRSF1A;DAB2;SFRP2;HYAL2;SNAI2;HCLS1;TLR7;IL18R1;TLR2;DACT1
regulation of B cell mediated immunity (GO:0002	0,00012639	0,001549725	-1,2184649	7,883075596 C3;FCER1G;PTPRC;BTK;FCER1A;PTPN6;FCGR1A
response to nutrient levels (GO:0031667)	0,00057799	0,005253362	-1,5014887	7,881144761 ABCA1;GPX1;STC1;CYBB;ADM;CYBA;TNFRSF11B;SSTR2;SOD2;AQP3;FSTL1;ASS1;SFRP2;IFI16;KYNU;PDK4;ALPL;APOE;DSC2
negative regulation of intrinsic apoptotic signalin	0,00023766	0,002552184	-1,3110864	7,828242664 CD74;VNN1;GPX1;SFRP2;BCL2A1;BDKRB2;SNAI2;SOD2;YBX3
response to zinc ion (GO:0010043)	0,00012639	0,001549725	-1,2090824	7,822374148 MT2A;KRT16;MT1L;MT1X;SOD2;S100A9;ASS1
regulation of nucleocytoplasmic transport (GO:0(	0,00049601	0,004658883	-1,4436024	7,750671712 IL18;IGF1;TMEM173;TNFRSF1A;DAB2;SFRP2;HYAL2;SNAI2;HCLS1;TLR7;SFN;IL18R1;DACT1;TLR2
negative regulation of leukocyte differentiation (	0,00021436	0,00236219	-1,2789191	7,735115424 LYN;CD74;IL4R;LILRB1;LILRB3;LILRB4;JAK3;RUNX1;C1QC
myeloid cell differentiation (GO:0030099)	0,00039794	0,003880982	-1,3916574	7,726018525 LYN;CD86;BATF3;SPI1;CITED2;ZBTB16;GAB3;RUNX1;BATF;DAB2;IFI16;HCLS1;PTPN6
cytosolic calcium ion homeostasis (GO:0051480)	0,00044272	0,004217097	-1,4103156	7,71246338 CCR1;EDN1;C5AR1;LPAR3;ADM;ESR1;PIK3CG;PTPRC;TRPV4;BDKRB2;C3AR1;S1PR3;CCR5;TGM2
positive regulation of leukocyte chemotaxis (GO:	0,00015444	0,001803145	-1,2107534	7,649809767 CCR1;CD74;EDN1;RAC2;C3AR1;NCKAP1L;PLA2G7;CMKLR1
positive regulation of chemokine production (GC	0,00012639	0,001549725	-1,1613515	7,513570412 PYCARD;CD74;IL4R;TLR7;ALOX15B;MYD88;TLR2
negative regulation of extrinsic apoptotic signalir	0,00038185	0,003753903	-1,3420248	7,49515344 LGALS3;DAB2;COL2A1;SFRP2;GPX1;NOS3;HGF;SNAI2;GAS1;IGF1
positive regulation of secretion by cell (GO:1903!	0,0006155	0,005534613	-1,4337853	7,451000203 EDN1;IL4R;FCER1G;SYK;RAB27A;ALOX15B;RUNX1;FGR;PYCARD;RBP4;HYAL2;ADORA3;CLEC5A;CASP1;FCER1A;CD14;TLR2
negative regulation of response to wounding (GC	0,00050412	0,004721943	-1,3810787	7,396415085 SPN;EDN1;GPX1;NOS3;SERPINF1;IL20RB;ACP5;SERPING1;APOE;MEFV;TNFRSF1B;TNFRSF1A
regulation of ERK1 and ERK2 cascade (GO:00703	0,0006084	0,005485382	-1,4177862	7,38052495 LYN;CCR1;CD74;SYK;C5AR2;C5AR1;TREM2;IGF1;NPNT;SCIMP;CTGF;PYCARD;PTPN6
cellular divalent inorganic cation homeostasis (G	0,00065477	0,005856421	-1,4283206	7,341877396 CCR1;EDN1;SLC11A1;C5AR1;STC1;ADM;LPAR3;ESR1;SLC39A14;PIK3CG;PTPRC;TRPV4;BDKRB2;C3AR1;S1PR3;APOE;CCR5;TGM2
leukocyte degranulation (GO:0043299)	3,1229E-05	0,000476492	-0,9544935	7,30097788 LAT2;HCK;STXBP2;RAB27A;PIK3CG;STX11
regulation of angiogenesis (GO:0045765)	0,00052464	0,00487349	-1,3662557	7,273870226 NPR1;NOS3;SERPINF1;ADM;SULF1;RUNX1;TNFRSF1A;C3;SFRP2;LRG1;STAB1;C3AR1;CTSH;ANGPTL4
regulation of myeloid leukocyte differentiation ((	0,00041481	0,00400788	-1,2870243	7,103721191 CCR1;LYN;TYROBP;HCLS1;LILRB1;IKZF1;LILRB3;LILRB4;C1QC;RUNX1
regulation of chemotaxis (GO:0050920)	0,00054805	0,00506308	-1,34161	7,091455912 LYN;CCR1;CD74;EDN1;C5AR2;RAC2;C3AR1;NCKAP1L;SEMA3F;PLA2G7;CMKLR1
negative regulation of phosphorylation (GO:0042	0,00087824	0,007312133	-1,4279134	7,022792547 LYN;CD300A;IGFBP3;IRAK3;IGF1;SMAD6;SAMSN1;GADD45G;PYCARD;FABP4;SFRP2;PTPRC;HYAL2;DDIT4;BDKRB2;PTPN6;SFN;APOE;DACT1;ENG
positive regulation of B cell activation (GO:00508	0,00019846	0,002194123	-1,1349576	6,948179465 SASH3;CD74;PTPRC;SYK;BTK;NCKAP1L;TNFSF13B;IL13RA1
cellular response to external stimulus (GO:00714	0,00072761	0,006389363	-1,3623038	6,883885329 LYN;CYBA;TNFRSF1A;SFRP2;IFI16;CASP5;PDK4;CASP1;TLR8;TLR7;TLR5;DSC2;MYD88;ENG
regulation of interleukin-12 production (GO:0032	0,00023165	0,002511609	-1,143807	6,847779887 ACP5;LILRB1;IRAK3;MEFV;JAK3;TLR2;CMKLR1
regulation of protein targeting (GO:1903533)	0,00085719	0,007154547	-1,3679058	6,757464448 IL18;IGF1;TMEM173;TNFRSF1A;DAB2;SFRP2;HYAL2;SNAI2;HCLS1;TLR7;IL18R1;TLR2;DACT1
morphogenesis of a branching epithelium (GO:0(	0,00064502	0,005784571	-1,3004023	6,700402519 EDN1;B4GALT1;SIX1;ADM;IGF1;NPNT;ESR1;SFRP2;COL4A1;RSPO3;FAT4;TGM2;ENG
epithelial cell differentiation (GO:0030855)	0,00093815	0,007715745	-1,3668198	6,648884468 DSP;TAGLN;ST14;CSTA;CEBPB;SIX1;RAB27A;IGF1;KLF2;LGALS3;SULT1B1;UPK1B;DAB2;SCUBE1;COL4A1;KRT16;ID3;TAGLN2;TP63
response to estrogen (GO:0043627)	0,00081062	0,006899704	-1,3184397	6,560921035 IL4R;GPX1;ABCC2;CITED2;TNFRSF11B;SMAD6;SSTR2;ESR1;ASS1;CTGF;TNFRSF1A;KRT19;ZNF366
cell killing (GO:0001906)	0,00017262	0,00196645	-1,0501922	6,544299643 STXBP2;CTSH;PTPN6;FCGR1A;APOL1;HAMP;CTSC
regulation of muscle system process (GO:009025	0,00073808	0,006464468	-1,2950031	6,528673275 EDN1;NOS3;TNNC1;TNNT2;GJA5;P2RX1;STC1;IGF1;NPNT;SSTR2;PIK3CG;CTGF
positive regulation of apoptotic signaling pathwa	0,00090598	0,007505691	-1,3248853	6,481463144 STEAP3;RIPK3;RUNX3;PYCARD;PTPRC;HYAL2;CTSH;SFN;SIGLEC1;JAK3;S100A9;CTSC;TP63
fatty acid metabolic process (GO:0006631)	0,00095435	0,007810879	-1,3310433	6,458538342 GGT5;CD74;EDN1;GPX1;SYK;HPGD;ACSL5;ALOX15B;TNFRSF1A;PTGS1;C3;ACADL;ALOX5;TBXAS1;ALOX5AP;DPEP2;FCER1A;TLR2
positive regulation of angiogenesis (GO:0045766	0,00052822	0,004893245	-1,2027586	6,398555289 C3;SFRP2;LRG1;NOS3;C3AR1;CTSH;ADM;ANGPTL4;RUNX1;TNFRSF1A
regulation of blood vessel size (GO:0050880)	0,00035493	0,00356194	-1,1250574	6,342454637 EDN1;GPX1;NPR1;NOS3;P2RX1;BDKRB2;APOE;SOD2
cellular response to interleukin-1 (GO:0071347)	0,00023165	0,002511609	-1,0543875	6,312440372 PYCARD;EDN1;IL1R1;HYAL2;IRAK3;APOE;KLF2
positive regulation of cell adhesion (GO:0045785	0,00106652	0,008411141	-1,2834674	6,132661334 EGFL6;SYK;MYOC;CITED2;NPNT;DAB2;SFRP2;ABI3BP;COL8A1;PTPN6;NCKAP1L;ITGA5;TGM2
response to transition metal nanoparticle (GO:19	0,00077369	0,006741287	-1,2191175	6,094983335 MT2A;KRT16;MT1L;AQP9;ADIRF;CYBRD1;MT1X;SOD2;S100A9;ASS1
regulation of tube size (GO:0035150)	0,00039586	0,003880359	-1,0971446	6,091157949 EDN1;GPX1;NPR1;NOS3;P2RX1;BDKRB2;APOE;SOD2
angiogenesis (GO:0001525)	0,00103263	0,008249425	-1,2645834	6,066980363 B4GALT1;GPX1;SYK;NOS3;ITGB3;IL18;PIK3CG;APOLD1;CTGF;KLF5;GJA5;ANPEP;COL8A1;HPSE;ANGPTL4;ITGA5
regulation of intracellular protein transport (GO:	0,0013232	0,009875121	-1,3034533	6,019004041 TMEM30B;IL18;IGF1;TMEM173;TNFRSF1A;DAB2;SFRP2;HYAL2;SNAI2;HCLS1;SFN;TLR7;IL18R1;DACT1;TLR2
negative regulation of response to external stimu	0,00110032	0,008432451	-1,2541193	5,989257305 EDN1;GPX1;NOS3;SERPINF1;C5AR2;IL20RB;SEMA3F;MEFV;TNFRSF1B;TNFRSF1A;SPN;SNAI2;ACP5;SERPING1;APOE
phagocytosis (GO:0006909)	0,00034965	0,003553799	-1,0463678	5,90124023 HCK;CD93;SLC11A1;PECAM1;CD14;MERTK;TGM2
leukocyte cell-cell adhesion (GO:0007159)	0,00026672	0,002828226	-1,0021043	5,880453438 PTPRC;SYK;ITGB2;RAC2;ITGA5;S100A9;FERMT3
morphogenesis of a branching structure (GO:000	0,00101056	0,008178732	-1,2188153	5,857892413 EDN1;B4GALT1;SIX1;ADM;IGF1;NPNT;ESR1;SFRP2;COL4A1;RSPO3;FAT4;TGM2;ENG
regulation of osteoblast differentiation (GO:0045	0,00077369	0,006741287	-1,1682634	5,840738141 IFITM1;CEBPB;SFRP2;IGFBP5;HGF;SNAI2;ID3;IGF1;NPNT;TP63
regulation of phosphatidylinositol 3-kinase signal	0,00048925	0,004608273	-1,0803024	5,811921066 FGR;MYOC;ADORA3;HGF;HCLS1;PTPN6;IGF1;PIK3AP1
natural killer cell activation (GO:0030101)	0,00034965	0,003553799	-1,0269558	5,791761768 ELF4;IL18;RAB27A;IKZF1;MERTK;IL18R1;STX11
tissue morphogenesis (GO:0048729)	0,00120827	0,009114739	-1,2315849	5,78581631 DSP;EDN1;B4GALT1;CITED2;TNNC1;SIX1;ADM;IGF1;NPNT;ESR1;SFRP2;COL4A1;TNNT2;PKP2;SNAI2;CTSH;RSPO3;FAT4;TP63;TGM2;ENG
positive regulation of endopeptidase activity (GC	0,00094119	0,007721894	-1,1767626	5,723414943 PYCARD;CASP7;SFRP2;IFI16;P2RX1;CASP1;CTSH;NLRC4;MEFV;S100A9;CTGF
cellular response to heat (GO:0034605)	0,00012743	0,001556813	-0,8811631	5,69682006 LYN;TRPV4;MYOF;SLC52A3;TFEC;MKI67
response to amino acid (GO:0043200)	0,00066372	0,005874169	-1,1055606	5,679475414 LYN;EDN1;CEBPB;GPX1;COL4A1;CYBA;ASS1;CTGF;TNFRSF1A
T cell activation involved in immune response (G	0,00034965	0,003553799	-1,005764	5,672245685 CD86;SLC11A1;RAB27A;LILRB1;IL18R1;STX11;BATF
response to ionizing radiation (GO:0010212)	0,00088202	0,007325509	-1,1451163	5,629841429 GPX1;SFRP2;PTPRC;IFI16;KRT16;NABP1;SNAI2;CYBA;BRCA2;SOD2;TP63
interferon-gamma-mediated signaling pathway (	0,00044055	0,004217097	-1,0278883	5,621118313 HCK;MT2A;PTAFR;HLA-C;PTPN6;FCGR1A;IFI30;FCGR1B
regulation of lymphocyte apoptotic process (GO:	0,00039823	0,003880982	-0,9960073	5,529501084 LYN;CD74;LGALS3;RIPK3;BTK;SIGLEC1;JAK3
regulation of ossification (GO:0030278)	0,00118624	0,008968644	-1,1719814	5,524744611 CCR1;IFITM1;CEBPB;IGFBP5;ZBTB16;HGF;IGF1;NPNT;SFRP2;MGP;SNAI2;ID3;TP63
negative regulation of inflammatory response (G	0,00085223	0,007130802	-1,1140439	5,507088298 SPN;GPX1;SERPINF1;ACP5;IL20RB;APOE;MEFV;TNFRSF1B;TNFRSF1A
regulation of cysteine-type endopeptidase activit	0,0013365	0,009875121	-1,185448	5,474086658 GPX1;HGF;NLRC4;MEFV;CTGF;PYCARD;CASP7;SFRP2;IFI16;P2RX1;CASP1;CTSH;SFN;S100A9
positive regulation of lymphocyte mediated imm	0,00066209	0,005874169	-1,0632169	5,461947998 C3;SASH3;FCER1G;PTPRC;FCER1A;HLA-C;FCGR1A;MR1
cytokine secretion (GO:0050663)	0,00015555	0,001803145	-0,8644701	5,461914955 LYN;ABCA1;PNP;LILRB1;LCP2;NLRC4
lymphocyte differentiation (GO:0030098)	0,00112506	0,008602467	-1,1373005	5,408666858 SPI1;RIPK3;IKZF1;MERTK;POU2F2;BATF;MAFB;PTPRC;TNFSF8;IL7R;JAK3;IL18R1;MPZL2
negative regulation of epithelial cell proliferation	0,00103171	0,008249425	-1,1264083	5,40406994 SFRP2;SERPINF1;SNAI2;GAS1;SFN;APOE;BRCA2;SULF1;RUNX3;ENG
regulation of vasculature development (GO:1901	0,0013365	0,009875121	-1,1525148	5,322009704 NPR1;NOS3;SERPINF1;ADM;SULF1;RUNX1;TNFRSF1A;C3;SFRP2;LRG1;STAB1;C3AR1;CTSH;ANGPTL4
negative regulation of interleukin-6 production ((	0,00018836	0,002096225	-0,8619169	5,315973439 C5AR2;NCKAP1L;PRG4;IRAK3;KLF2;TNFRSF1A
cellular response to interferon-gamma (GO:0071	0,00078509	0,006823037	-1,0648426	5,310849656 HCK;MT2A;PTAFR;HLA-C;PTPN6;FCGR1A;IFI30;FCGR1B;ASS1
regulation of activated T cell proliferation (GO:00	0,0002264	0,00248511	-0,8793663	5,273945149 CD86;PYCARD;RIPK3;IL18;IGF1;LRRC32
regulation of blood pressure (GO:0008217)	0,00128849	0,00967657	-1,1226865	5,207073731 KCNK6;EDN1;ERAP2;NPR1;NCF2;NOS3;P2RX1;CYBA;SOD2;PTGS1;ENG
positive regulation of endocytosis (GO:0045807)	0,00092395	0,007617544	-1,0658772	5,198604216 C3;PYCARD;DAB2;FCER1G;SLC11A1;NCKAP1L;CD14;FCGR1A;MERTK
negative regulation of myeloid cell differentiatio	0,00066209	0,005874169	-1,0089503	5,183170043 LYN;MAFB;ZBTB16;LILRB1;LILRB3;LILRB4;C1QC;RUNX1
plasma lipoprotein particle organization (GO:007	0,00018836	0,002096225	-0,8357163	5,154378483 ABCA1;SOAT1;APOC1;APOE;APOB;PLA2G7
negative regulation of peptidyl-tyrosine phospho	0,00027024	0,002838751	-0,870221	5,103316237 SFRP2;PTPRC;CD300A;HYAL2;PTPN6;SAMSN1
positive regulation of interleukin-6 production (G	0,00051152	0,004777956	-0,9373371	5,008888212 PYCARD;TLR1;FCER1G;HYAL2;TLR7;MYD88;TLR2
regulation of exocytosis (GO:0017157)	0,00118494	0,008968644	-1,0477344	4,939041899 FGR;LYN;B4GALT1;FCER1G;SYK;CD300A;ADORA3;STXBP2;RAB27A;FCER1A
action potential (GO:0001508)	0,00066209	0,005874169	-0,9560577	4,911450815 CHRNA1;DSP;GJA5;P2RX1;GPR88;PKP2;DSG2;DSC2
regulation of vasoconstriction (GO:0019229)	0,00057705	0,005253362	-0,9078754	4,765335447 EDN1;HRH1;HRH2;P2RX1;TBXAS1;BDKRB2;ADM
protein-lipid complex subunit organization (GO:0	0,00027024	0,002838751	-0,8117375	4,760345992 ABCA1;SOAT1;APOC1;APOE;APOB;PLA2G7
regulation of muscle contraction (GO:0006937)	0,00135624	0,009941844	-1,0162017	4,6857089 EDN1;TNNC1;P2RX1;TNNT2;GJA5;STC1;NPNT;SSTR2;PIK3CG;CTGF
regulation of interleukin-8 production (GO:0032(	0,00057705	0,005253362	-0,8601853	4,515015283 PYCARD;C5AR2;HYAL2;TLR8;TLR7;TLR5;TLR2
defense response to Gram-positive bacterium (G	0,00057705	0,005253362	-0,8391549	4,404629361 FGR;HCK;C5AR1;ACP5;TNFSF8;MYD88;TLR2
positive regulation of transcription factor import	0,00044269	0,004217097	-0,8040131	4,39683244 IL18;HCLS1;TLR7;TMEM173;IL18R1;TLR2
regulation of interleukin-1 beta production (GO:(	0,00059848	0,005410407	-0,8246188	4,304040882 PYCARD;IFI16;CASP1;ACP5;S1PR3;MEFV
positive regulation of B cell proliferation (GO:003	0,00044269	0,004217097	-0,7616576	4,165207315 SASH3;CD74;PTPRC;NCKAP1L;TNFSF13B;IL13RA1
positive regulation of interleukin-8 production (G	0,00051603	0,004806768	-0,7599063	4,056174833 PYCARD;HYAL2;TLR8;TLR7;TLR5;TLR2
regulation of viral genome replication (GO:0045(	0,00101143	0,008178732	-0,7967952	3,829571682 IFITM3;TOP2A;IFITM1;PLSCR1;IFITM2;IFI16;SLPI
regulated secretory pathway (GO:0045055)	0,00044269	0,004217097	-0,6932654	3,791196913 LAT2;HCK;STXBP2;RAB27A;PIK3CG;STX11
cartilage development (GO:0051216)	0,00115465	0,008769126	-0,7890906	3,737542051 COMP;EDN1;COL2A1;PRRX1;ZBTB16;HYAL2;PRRX2;SULF1
regulation of immunoglobulin mediated immune	0,00079376	0,006845382	-0,7302727	3,639811382 C3;FCER1G;PTPRC;FCER1A;PTPN6;FCGR1A
regulation of calcium-mediated signaling (GO:00!	0,00059848	0,005410407	-0,6955306	3,630274012 SYK;ADORA3;FCER1A;TREM2;IGF1;CMKLR1
phagosome maturation (GO:0090382)	0,0006908	0,006081906	-0,6924234	3,533046881 NCF2;NOS3;SLC11A1;NCF4;CYBB;CYBA
negative regulation of myeloid leukocyte differer	0,00079376	0,006845382	-0,6942404	3,460219825 LYN;LILRB1;LILRB3;LILRB4;C1QC;RUNX1
homotypic cell-cell adhesion (GO:0034109)	0,00101143	0,008178732	-0,7082121	3,403821937 ITGB3;RAC2;PTPN6;S100A9;DSC2;PIK3CG;FERMT3
regulation of interleukin-1 production (GO:0032(	0,00103485	0,008249425	-0,6978044	3,347794718 PYCARD;IFI16;CASP1;ACP5;S1PR3;MEFV
negative regulation of T cell proliferation (GO:00	0,00103485	0,008249425	-0,689792	3,30935401 SPN;IL20RB;LILRB1;PTPN6;VSIG4;LRRC32
negative regulation of viral genome replication ((	0,00079376	0,006845382	-0,6487266	3,233371069 IFITM3;IFITM1;PLSCR1;IFITM2;IFI16;SLPI
positive regulation of osteoblast differentiation (	0,00124402	0,009363503	-0,6477223	3,025469138 CEBPB;IFITM1;SFRP2;HGF;IGF1;NPNT;TP63
T cell proliferation (GO:0042098)	0,00090816	0,007505691	-0,5808699	2,841669901 CD86;PTPRC;ELF4;SLC11A1;LILRB1;PIK3CG
cholesterol transport (GO:0030301)	0,00103485	0,008249425	-0,574663	2,75701001 ABCA1;MSR1;SOAT1;APOC1;APOE;APOB
sterol transport (GO:0015918)	0,00103485	0,008249425	-0,5699065	2,734190328 ABCA1;MSR1;SOAT1;APOC1;APOE;APOB
regulation of leukocyte mediated cytotoxicity (G(	0,0013285	0,009875121	-0,5787311	2,672427814 PTPRC;RIPK3;HLA-C;LILRB1;IL7R;MR1
regulation of nitric oxide biosynthetic process (G	0,0013285	0,009875121	-0,5696122	2,630318956 EDN1;ACP5;SOD2;ESR1;ASS1;TLR2
positive regulation of neutrophil migration (GO:1	0,00010613	0,001330398	-0,3946026	2,613167596 CD74;EDN1;RAC2;C3AR1;NCKAP1L
positive regulation of neutrophil chemotaxis (GO	0,00010613	0,001330398	-0,3833875	2,53889805 CD74;EDN1;RAC2;C3AR1;NCKAP1L
response to gamma radiation (GO:0010332)	0,0013285	0,009875121	-0,4956952	2,288989711 GPX1;PTPRC;CYBA;BRCA2;SOD2;TP63
defense response to protozoan (GO:0042832)	5,6674E-05	0,00079297	-0,2875577	2,053082932 IL4R;CLEC7A;SLC11A1;CD37;BATF
neutrophil activation (GO:0042119)	0,00014074	0,001694898	-0,3202491	2,043231876 FCER1G;TYROBP;SYK;STXBP2;STX11
positive regulation of granulocyte chemotaxis (G	0,00018336	0,002060953	-0,304358	1,882328237 CD74;EDN1;RAC2;C3AR1;NCKAP1L
response to laminar fluid shear stress (GO:00346	3,9878E-05	0,000594999	-0,2404094	1,785508892 ABCA1;NCF2;SMAD6;ASS1;KLF2
detection of external biotic stimulus (GO:009858	0,00018336	0,002060953	-0,2387269	1,476427495 TLR1;LY96;CD1D;NLRC4;TLR2
positive regulation of immunoglobulin mediated	0,00081233	0,006899704	-0,2924554	1,455339029 C3;FCER1G;PTPRC;FCER1A;FCGR1A
granulocyte activation (GO:0036230)	0,00023516	0,002533379	-0,234198	1,400082616 TYROBP;FCER1G;SYK;STXBP2;STX11
positive regulation of B cell mediated immunity (	0,00081233	0,006899704	-0,2800865	1,393787933 C3;FCER1G;PTPRC;FCER1A;FCGR1A
response to interferon-beta (GO:0035456)	0,00010613	0,001330398	-0,2033381	1,346561159 IFITM3;IFITM1;PLSCR1;IFITM2;TMEM173
detection of biotic stimulus (GO:0009595)	0,0003713	0,003660839	-0,2314877	1,298660715 TLR1;LY96;CD1D;NLRC4;TLR2
regulation of B cell differentiation (GO:0045577)	0,00023516	0,002533379	-0,2100591	1,255775726 PTPRC;SYK;BTK;NCKAP1L;PTPN6
myeloid dendritic cell activation (GO:0001773)	0,00018336	0,002060953	-0,1943457	1,201948119 CD86;PYCARD;BATF3;SPI1;BATF
plasma lipoprotein particle assembly (GO:00343	5,6674E-05	0,00079297	-0,1624476	1,159831185 ABCA1;SOAT1;APOC1;APOE;APOB
positive regulation of lipid storage (GO:0010884)	0,00010613	0,001330398	-0,1745926	1,156200883 C3;MSR1;HILPDA;OSBPL11;APOB
immunoglobulin mediated immune response (GC	5,6674E-05	0,00079297	-0,1586486	1,132707231 CD74;FCER1G;TLR8;FCGR1A;MYD88
positive regulation of immunoglobulin productio	0,00081233	0,006899704	-0,2254905	1,122103048 SASH3;RBP4;PTPRC;CD37;IL13RA1
prostaglandin metabolic process (GO:0006693)	0,0003713	0,003660839	-0,1871731	1,050053096 CD74;EDN1;HPGD;PTGS1;TNFRSF1A
protein-lipid complex assembly (GO:0065005)	0,00010613	0,001330398	-0,1555543	1,030123897 ABCA1;SOAT1;APOC1;APOE;APOB
mast cell activation (GO:0045576)	7,8448E-05	0,00103736	-0,1446751	0,99407392 LAT2;FCER1G;CD48;LCP2;PIK3CG
B cell mediated immunity (GO:0019724)	0,00018336	0,002060953	-0,1591581	0,984326838 CD74;FCER1G;TLR8;FCGR1A;MYD88
prostanoid metabolic process (GO:0006692)	0,0003713	0,003660839	-0,1647972	0,924522789 CD74;EDN1;HPGD;PTGS1;TNFRSF1A
middle ear morphogenesis (GO:0042474)	0,0003713	0,003660839	-0,1634001	0,916684653 EDN1;PRRX1;PRRX2;SIX1;GAS1
positive regulation of interleukin-10 production (	0,0004583	0,004328857	-0,1669155	0,908429534 SASH3;PYCARD;FCER1G;IL20RB;TLR2
cell communication involved in cardiac conductic	0,0003713	0,003660839	-0,1577634	0,885062373 DSP;GJA5;PKP2;DSG2;DSC2
regulation of platelet activation (GO:0010543)	0,00081233	0,006899704	-0,1744261	0,867992367 LYN;FCER1G;SYK;NOS3;APOE
regulation of CD4-positive, alpha-beta T cell diffe	0,00096643	0,007871485	-0,1673618	0,810785691 CD86;SASH3;IL4R;NCKAP1L;JAK3
positive regulation of calcium-mediated signaling	0,00055982	0,00514363	-0,146192	0,770431206 SYK;ADORA3;FCER1A;TREM2;IGF1
cholesterol efflux (GO:0033344)	0,0003713	0,003660839	-0,1251982	0,702369666 ABCA1;SOAT1;APOC1;APOE;APOB
T cell selection (GO:0045058)	0,00081233	0,006899704	-0,1333626	0,663649102 SPN;CD74;PTPRC;SYK;CD1D
leukotriene biosynthetic process (GO:0019370)	0,0004583	0,004328857	-0,1155589	0,62892355 GGT5;SYK;ALOX5;ALOX5AP;FCER1A
heterotypic cell-cell adhesion (GO:0034113)	0,00029737	0,003104408	-0,1043536	0,60263498 PTPRC;ITGB3;ITGB2;CD1D;ITGA5
superoxide anion generation (GO:0042554)	2,721E-05	0,000420884	-0,0644489	0,50097088 EDN1;NCF2;CYBB;CYBA;SOD2
regulation of macrophage derived foam cell diffe	0,00096643	0,007871485	-0,0986729	0,478021701 ABCA1;MSR1;ITGB3;APOB;ALOX15B
leukocyte mediated cytotoxicity (GO:0001909)	0,00067732	0,005978853	-0,0337105	0,17258192 STXBP2;CTSH;PTPN6;FCGR1A;CTSC
embryonic cranial skeleton morphogenesis (GO:(	0,00114122	0,008686668	0,00011797	-0,000559904 TBX15;PRRX1;PRRX2;SIX1;GAS1
intrinsic apoptotic signaling pathway in response	0,00133835	0,009875121	0,07922478	-0,365839194 PYCARD;IFI16;DDIT4;BRCA2;TP63
cardiac muscle cell action potential (GO:0086001	0,00133835	0,009875121	0,08501377	-0,392571224 DSP;GJA5;PKP2;DSG2;DSC2
positive regulation of leukocyte degranulation (G	1,1277E-05	0,000197022	0,04924155	-0,42013859 FGR;FCER1G;SYK;ADORA3;FCER1A
cardiac muscle cell action potential involved in cc	0,00114122	0,008686668	0,08954952	-0,424998967 DSP;GJA5;PKP2;DSG2;DSC2
negative regulation of T cell mediated immunity	1,1277E-05	0,000197022	0,05825362	-0,497031374 SPN;PTPRC;IL20RB;LILRB1;IL7R
lipoxygenase pathway (GO:0019372)	1,7904E-05	0,000296932	0,06755662	-0,548695265 GPX1;HPGD;ALOX5;ALOX5AP;ALOX15B
positive regulation of mast cell activation (GO:00	1,1277E-05	0,000197022	0,13122549	-1,119641725 FGR;FCER1G;SYK;ADORA3;FCER1A
positive regulation of mast cell degranulation (G(	1,9238E-06	4,56841E-05	0,38629197	-3,860509558 FGR;FCER1G;SYK;ADORA3;FCER1A
positive regulation of mast cell activation involve	1,9238E-06	4,56841E-05	0,41155263	-4,112958553 FGR;FCER1G;SYK;ADORA3;FCER1A
neutrophil activation involved in immune respon	1,9238E-06	4,56841E-05	0,5338552	-5,335221133 FCER1G;TYROBP;SYK;STXBP2;STX11
myeloid dendritic cell differentiation (GO:004301	0,00107522	0,008411141	1,41207514	-6,747174761 CD86;BATF3;SPI1;BATF
regulation of tumor necrosis factor biosynthetic		0,00084069	0,007051722	1,37367161	-6,805833165 SPN;TLR1;LILRB1;TLR2
regulation of antigen processing and presentatio	0,0013521	0,009933096	1,50722571	-6,95114879 PYCARD;CD74;SLC11A1;TREM2
regulation of interferon-gamma biosynthetic pro	0,00084069	0,007051722	1,40492378	-6,960671524 EBI3;TLR8;LILRB1;TLR7
dendritic cell chemotaxis (GO:0002407)	0,00084069	0,007051722	1,41686696	-7,019843811 CCR1;CXCR1;CCR5;PIK3CG
positive regulation of alpha-beta T cell proliferati	0,00084069	0,007051722	1,43408044	-7,105127722 PTPRC;SYK;EBI3;IL18
regulation of granulocyte differentiation (GO:003	0,00107522	0,008411141	1,49304596	-7,134069408 HCLS1;IKZF1;RUNX1;C1QC
programmed necrotic cell death (GO:0097300)	0,0013521	0,009933096	1,54998719	-7,148359736 RIPK3;MLKL;CASP1;PYGL
positive regulation of cell adhesion mediated by	0,00084069	0,007051722	1,45560069	-7,211749424 SFRP2;SYK;NCKAP1L;PTPN6
azole transport (GO:0045117)	0,00107522	0,008411141	1,56509883	-7,478352289 LYN;EDN1;ADORA3;BTK
positive regulation of antigen processing and pre	0,00035306	0,003553799	1,70253292	-9,601839914 PYCARD;CD74;SLC11A1;TREM2
bundle of His cell to Purkinje myocyte communic	8,752E-07	2,36095E-05	0,91808495	-9,781151254 DSP;GJA5;PKP2;DSG2;DSC2
positive regulation of insulin-like growth factor r	0,00035306	0,003553799	1,77350971	-10,00213046 IGFBP5;IGFBP4;IGFBP3;IGF1
regulation of B cell receptor signaling pathway (C	0,00035306	0,003553799	1,83340792	-10,33994068 LYN;PTPRC;CD300A;PTPN6
antigen processing and presentation of endogen	0,00035306	0,003553799	1,91348441	-10,79155112 CD74;ERAP2;HLA-C;CD1D
response to interleukin-15 (GO:0070672)	0,00035306	0,003553799	1,95943531	-11,05070218 CXCR1;IL7R;IL2RG;JAK3
regulation of gamma-delta T cell activation (GO:(	0,00024994	0,002667036	1,98923116	-11,78975013 PTPRC;SYK;NCKAP1L;LILRB1
response to peptidoglycan (GO:0032494)	0,00017038	0,001954186	2,20370442	-13,74622658 C5AR1;IRAK3;MYD88;TLR2
angiogenesis involved in wound healing (GO:006	0,00017038	0,001954186	2,3784928	-14,8365183 GPX1;B4GALT1;ITGB3;HPSE
positive regulation of inflammatory response to a	0,00011087	0,00137958	2,28199461	-15,02916143 C3;FCER1G;FCER1A;FCGR1A
histamine transport (GO:0051608)	0,00011087	0,00137958	2,4643295	-16,23001466 LYN;EDN1;ADORA3;BTK
positive regulation of gamma-delta T cell activati	6,8028E-05	0,000934552	2,55409533	-17,81594836 PTPRC;SYK;NCKAP1L;LILRB1
production of molecular mediator involved in inf	3,8648E-05	0,000579209	2,83092715	-21,10129793 IL4R;ALOX5;ALOX5AP;SLC7A2
histamine secretion (GO:0001821)	3,8648E-05	0,000579209	3,05280966	-22,75517623 LYN;EDN1;ADORA3;BTK
positive regulation by symbiont of host defense r	0,00109504	0,008411141	6,93171356	-33,12110073 TLR8;TLR7;TLR2
modulation by symbiont of host defense respons	0,00109504	0,008411141	6,93929751	-33,15733833 TLR8;TLR7;TLR2
modulation by symbiont of host immune respon:	0,00109504	0,008411141	7,01338268	-33,51133195 TLR8;TLR7;TLR2
modulation by organism of defense response of (	0,00109504	0,008411141	7,07268556	-33,79469286 TLR8;TLR7;TLR2
positive regulation by organism of defense respo	0,00109504	0,008411141	7,07474892	-33,804552 TLR8;TLR7;TLR2
modulation by organism of immune response of	0,00109504	0,008411141	7,18420722	-34,32756544 TLR8;TLR7;TLR2
regulation of response to osmotic stress (GO:004	0,00109504	0,008411141	7,95817083	-38,02571694 TRPV4;BDKRB2;YBX3
very-low-density lipoprotein particle assembly (G	0,00109504	0,008411141	8,12831541	-38,83870142 SOAT1;APOC1;APOB
desmosome organization (GO:0002934)	0,00109504	0,008411141	8,17181839	-39,04656727 DSP;PKP2;SNAI2
type B pancreatic cell proliferation (GO:0044342	0,00109504	0,008411141	8,27942093	-39,56071355 IGFBP5;IGFBP4;IGFBP3
gap junction assembly (GO:0016264)	0,00109504	0,008411141	8,41350319	-40,20138517 GJB2;GJA5;PKP2
positive regulation of chemokine secretion (GO:(	0,00109504	0,008411141	9,29308441	-44,4041985 PYCARD;IL4R;ALOX15B

Supplementary Table 4

Legend:

**Term**	GO term - Biological Process
**P-value**	P value computed using a standard statistical method: Fisher's exact test or the hypergeometric test.
**Adjusted P-v**	Adjusted p-value using the Benjamini-Hochberg method for correction for multiple hypotheses testing
**Z-score**	Score computed using a modification to Fisher's exact test for deviation from an expected rank.
**Combined Sc**	Is a combination of the p-value and z-score calculated by multiplying the two scores as follows: c = log(p) * z, where p is the p value and z the z-score
**Genes**	List of genes related to the GO term

GO Biological Process - DOWN Genes (Pval < 0,05)

Term	P-value	Adjusted P-v	Z-score	Combined Score	Genes
synaptic transmission (GO:0(	7,60E-47	1,98E-43	-2,3519823	231,2688704	KCNG1;GABRB3;SNAP25;ACHE;KCNG4;RAB3A;KCNC1;KCNK9;KCNC2;KCNC3;CPNE6;HTR2C;HTR2A;CDH8;GRM3;RIMS1;PANX2;GRM[illegible]
neurotransmitter transport (	2,90E-26	3,77E-23	-2,1434195	110,6721043	SNAP25;BRSK1;RAB3A;STXBP1;CHAT;CACNA1B;CPLX2;CPLX1;RIMS2;RIMS1;SV2C;GRM4;SV2B;HRH3;SLC17A6;PPFIA3;SLC18A3;GAB[illegible]
neurotransmitter secretion (	1,03E-23	8,96E-21	-2,1200619	97,86524065	SNAP25;BRSK1;RAB3A;STXBP1;CHAT;CACNA1B;CPLX1;RIMS2;RIMS1;GRM4;HRH3;PPFIA3;SLC18A3;SYT4;UNC13A;SYT2;SLC32A1;GA[illegible]
regulation of neurotransmitt	9,45E-23	6,14E-20	-2,2078766	97,66860544	ACHE;SNAP25;BRSK1;RAB3A;STXBP1;CHAT;CACNA1B;CPLX1;RIMS2;RIMS1;GRM4;HRH3;SLC17A6;PPFIA3;SLC18A3;GABRA2;SYT4;SY[illegible]
behavior (GO:0007610)	1,13E-19	5,88E-17	-2,4021183	89,77327629	ATP8A2;HTR2C;HTR2A;GRM5;UCHL1;JPH3;GRM4;NETO1;SCN9A;ADORA1;SCN1A;CHRNB2;NRG1;TACR1;SEZ6L;MAPK8IP2;GABRG2;[illegible]
regulation of ion transmemb	7,60E-19	3,30E-16	-2,3398233	83,41178236	HCN4;KCNG1;RYR2;KCNG4;KCNC1;KCNK9;KCNC2;KCNA1;KCNC3;KCNA2;CACNA1B;CABP1;CACNA1G;CACNG8;JPH3;SCN9A;KCNE1L;[illegible]
secretion by cell (GO:003294	2,91E-18	1,08E-15	-2,4042785	82,8488968	SNAP25;RAB3A;RAB3D;RIMS2;ARHGAP44;SCAMP5;RIMS1;PCLO;GRM4;PPFIA3;SLC18A3;NSF;UNC13C;UNC13A;SLC32A1;SLC6A11;P[illegible]
regulation of transmembran	3,82E-18	1,24E-15	-2,3525875	80,74871234	HCN4;KCNG1;RYR2;KCNG4;KCNC1;KCNK9;KCNC2;KCNA1;KCNC3;KCNA2;CACNA1B;CABP1;CACNA1G;CACNG8;JPH3;SCN9A;KCNE1L;[illegible]
single-organism behavior (GC	3,83E-17	9,96E-15	-2,3853055	76,90370641	SLC24A2;SHC3;CHRNA7;CHAT;SEZ6;SLC1A2;ATP1A3;CEND1;HTR2C;HMGCR;HTR2A;SEZ6L2;HOXD10;GLRA1;GRM5;UCHL1;GRIN2A;J[illegible]
signal release (GO:0023061)	1,97E-17	5,70E-15	-2,2180334	72,74927241	MEG3;SNAP25;BRSK1;RAB3A;STXBP1;CHAT;CACNA1B;CPLX1;RIMS2;RIMS1;PCLO;GRM4;HRH3;PPFIA3;SLC18A3;SYT4;UNC13A;SYT2;[illegible]
inorganic cation transmembr	1,67E-16	3,95E-14	-2,3050208	71,13940803	KCNG1;RYR2;KCNG4;KCNC1;KCNK9;KCNC2;KCNC3;JPH3;KCNT1;NCS1;SCN9A;SCN1B;SCN1A;TRPC5;KCNH5;CACNA2D3;KCNAB2;SLC3[illegible]
regulation of membrane pot	4,77E-16	1,03E-13	-2,3623278	70,63289771	HCN4;RYR2;POPDC3;KCNC2;CHRNA7;CELF4;CACNA1B;SEZ6;CACNA1G;RIMS2;RIMS1;GLRA1;GRIN2A;CUX2;NETO1;SCN9A;ADORA1;[illegible]
regulation of synaptic transm	1,17E-15	2,17E-13	-2,3565141	68,71807469	SLC24A2;RAB3B;NAPB;SNAP25;ACHE;RAB3A;KCNC3;STXBP1;CHRNA7;CHAT;HTR2A;CPLX2;GRM3;RIMS1;GRM5;GRIN2A;JPH3;GRM4[illegible]
potassium ion transport (GO:	5,36E-16	1,07E-13	-2,1640641	64,62732974	HCN4;KCNG1;SLC24A2;KCNG4;KCNC1;KCNK9;KCNC2;KCNA1;KCNC3;KCNA2;ATP1A3;KCNT1;KCNN1;KCNE1L;NSF;SLC12A5;KCNH5;KC[illegible]
cellular potassium ion transp	5,24E-15	8,52E-13	-2,1284394	59,15292551	HCN4;KCNG1;KCNG4;KCNC1;KCNK9;KCNC2;KCNA1;KCNC3;KCNA2;ATP1A3;KCNT1;KCNN1;KCNE1L;KCNH5;KCNJ10;KCNJ12;KCNJ14;K[illegible]
potassium ion transmembrar	5,24E-15	8,52E-13	-2,1257584	59,07841764	HCN4;KCNG1;KCNG4;KCNC1;KCNK9;KCNC2;KCNA1;KCNC3;KCNA2;ATP1A3;KCNT1;KCNN1;KCNE1L;KCNJ10;KCNH5;KCNJ12;KCNJ14;K[illegible]
adult behavior (GO:0030534)	4,88E-14	7,47E-12	-2,2492881	57,62795894	CHRNA7;CHAT;SEZ6;SLC1A2;CEND1;ATP1A3;HTR2A;SEZ6L2;HOXD10;GLRA1;UCHL1;SNCG;SPTBN2;SCN1A;CHRNB2;KCNJ10;KLHL1;S[illegible]
monovalent inorganic cation	1,79E-13	2,58E-11	-2,3362852	56,95745411	HCN4;SLC24A2;KCNG1;KCNG4;KCNC1;KCNK9;KCNC2;KCNC3;KCNA1;KCNA2;ATP1A3;KCNT1;SCN9A;SLC17A6;KCNN1;KCNE1L;SLC13A[illegible]
membrane depolarization (G	3,53E-13	4,84E-11	-2,2081835	52,44990099	CHRNB2;CACNA1B;CELF4;SEZ6;MAPK8IP2;GRIN2B;CACNA1G;GRIN1;RIMS2;STX1B;RIMS1;GRIN2A;CUX2;NETO1;SCN8A;SCN9A;ADO[illegible]
regulation of exocytosis (GO:	9,66E-12	1,20E-09	-2,2216721	45,642709	NSF;RAB3B;SYT4;NAPB;RAB3C;RAB3A;RAB3D;STXBP1;LGI3;CPLX2;CPLX1;SYT7;CACNA1G;SCAMP5;RIMS2;STX1B;GLRA1;PCLO;RAB2[illegible]
regulation of synaptic plastic	4,95E-12	6,44E-10	-2,1198762	44,86370533	SLC24A2;SNAP25;UNC13A;KCNJ10;RAB3A;STXBP1;ATP2B2;SYP;CPLX2;GRIN2B;GRIN1;RIMS1;GRM5;GRIN2A;JPH3;NETO1;STAR;NEU[illegible]
adult locomotory behavior ((	6,47E-11	7,65E-09	-2,2231252	41,54791397	KCNJ10;CHAT;KLHL1;SEZ6;ATP1A3;CEND1;SEZ6L2;SEZ6L;HOXD10;GRIN1;GLRA1;UCHL1;DAB1;GLRB;SCN8A;SNCG;FGF12;SCN1A
locomotory behavior (GO:00	1,42E-10	1,60E-08	-2,283975	40,99361689	CHAT;CACNA1B;SEZ6;HTR2C;CEND1;ATP1A3;SEZ6L2;HOXD10;GLRA1;GRM5;UCHL1;SNCG;SCN1A;CHRNB2;KCNJ10;KLHL1;OPRK1;AT[illegible]
regulation of neuronal synap	5,45E-10	5,90E-08	-2,2572103	37,57226618	KCNJ10;RAB3A;UNC13A;SYP;GRIN1;RIMS1;GRM5;JPH3;STAR;NETO1;NEURL1;NCDN;PPFIA3
exocytosis (GO:0006887)	1,13E-09	1,05E-07	-2,2276597	35,80257923	SNAP25;RAB3A;STXBP1;RAB3D;CPLX2;CPLX1;RIMS2;SCAMP5;ARHGAP44;RIMS1;GLRA1;PCLO;RAB26;OTOF;NSF;SYT4;UNC13C;SYT3;[illegible]
neuromuscular process (GO:(	8,73E-10	8,71E-08	-2,1729194	35,32458971	RBFOX1;ATP8A2;ATP2B2;NPAS1;HOXD10;GRIN2B;GLRA1;UCHL1;GRIN2A;JPH3;GLRB;SCN8A;NEFL;FGF12;SHANK1;SCN1A
learning (GO:0007612)	9,04E-10	8,71E-08	-2,1358905	34,72262113	SLC24A2;CHRNB2;SLC12A5;GABRA5;CHRNA7;ATP1A3;HMGCR;TACR1;MAPK8IP2;GRIN2B;GRIN1;GRM5;GRIN2A;JPH3;GRM4;NETO1[illegible]
learning or memory (GO:000	3,87E-09	3,36E-07	-2,2356804	33,32754887	CHRNB2;SLC24A2;SHC3;SLC12A5;GABRA5;CHRNA7;ATP1A3;HMGCR;TACR1;HTR2A;MAPK8IP2;GRIN2B;GRIN1;GRM5;GRIN2A;JPH3;[illegible]
regulation of postsynaptic m	8,53E-10	8,71E-08	-2,0159147	32,77220588	CELF4;SEZ6;MAPK8IP2;GRIN2B;GRIN1;RIMS2;STX1B;RIMS1;GLRA1;GRIN2A;CUX2;NETO1;ADORA1;SHANK1
regulation of excitatory post	2,15E-09	1,93E-07	-2,0408041	31,55327891	CELF4;SEZ6;MAPK8IP2;GRIN2B;GRIN1;RIMS2;STX1B;RIMS1;GRIN2A;CUX2;NETO1;ADORA1;SHANK1
cognition (GO:0050890)	1,32E-08	1,07E-06	-2,2453282	30,85906779	SLC24A2;SHC3;CHRNA7;ATP1A3;HMGCR;HTR2A;GRM5;GRIN2A;JPH3;GRM4;CUX2;HRH3;NETO1;ADORA1;CHRNB2;SLC12A5;GABRA[illegible]
alcohol biosynthetic process	8,22E-09	6,90E-07	-2,1552719	30,57650838	CDS1;IDI1;ACHE;PCYT1B;MVK;GOT1;HMGCS1;INSIG1;CHAT;CYP51A1;MSMO1;DHCR24;HMGCR;PLA2G5;SQLE;STAR;DHCR7
cholesterol biosynthetic proc	2,86E-08	2,23E-06	-2,2532889	29,32549613	IDI1;SQLE;MVK;HMGCS1;INSIG1;CYP51A1;MSMO1;DHCR24;DHCR7;HMGCR
sodium ion transport (GO:00	2,91E-08	2,23E-06	-2,1553057	28,05029084	HCN4;SLC10A4;SLC24A2;SLC6A15;ATP1A3;ATP1B1;SLC8A3;SLC5A7;SLC9A7;SCN8A;SC[illegible]
memory (GO:0007613)	4,75E-08	3,53E-06	-2,1462596	26,94595204	CHRNB2;SLC24A2;CHRNA7;ATP1A3;TACR1;HTR2A;GRIN2B;GRIN1;GRIN2A;JPH3;CUX2[illegible]
positive regulation of excitat	4,15E-07	2,63E-05	-2,5263927	26,63948615	RIMS2;STX1B;RIMS1;CUX2;NETO1;SHANK1;GRIN1
adult walking behavior (GO:(	9,29E-08	6,71E-06	-2,2285969	26,54621969	GLRA1;UCHL1;DAB1;KCNJ10;GLRB;SCN8A;CHAT;KLHL1;CEND1;SCN1A
anion transport (GO:000682(	1,05E-07	7,16E-06	-2,2301607	26,42145154	GABRB3;SNAP25;ATP8A2;RAB3A;STXBP1;SLC1A2;AQP6;SLC7A14;PLA2G5;SLC4A3;CPL[illegible]
sodium ion transmembrane t	9,72E-08	6,83E-06	-2,1421941	25,4785978	SLC24A2;HCN4;ATP1A3;ATP1B1;SLC8A3;SLC9A7;SCN8A;SCN9A;SCN4B;HCN2;SCN1B;[illegible]
sterol biosynthetic process ((	1,22E-07	8,12E-06	-2,1730868	25,47220116	IDI1;SQLE;MVK;HMGCS1;INSIG1;CYP51A1;MSMO1;DHCR24;DHCR7;HMGCR
synaptic vesicle exocytosis ((	6,41E-07	3,85E-05	-2,4277914	24,67878042	SNAP25;RIMS1;PCLO;RAB3A;OTOF;CPLX2;CPLX1
calcium ion transmembrane t	1,93E-07	1,25E-05	-2,0919726	23,61364189	SLC24A2;RYR2;TRPC5;CACNA1B;CACNA2D3;ATP2B3;ATP2B2;GRIN2B;CACNA1G;GRIN[illegible]
regulation of neuron projecti	5,55E-07	3,44E-05	-2,2372689	22,99586366	RET;SNAP25;ACHE;ATP8A2;SEZ6;STMN2;KNDC1;THY1;CUX2;NCS1;NEFL;NEFM;PLXNA[illegible]
regulation of vesicle-mediate	1,20E-06	6,65E-05	-2,2915944	22,04222509	RAB3B;NAPB;RAB3C;RAB3A;INSIG1;RAB3D;STXBP1;CPLX2;CPLX1;CACNA1G;RIMS2;S[illegible]
divalent metal ion transport	1,51E-06	8,02E-05	-2,2525807	21,2434312	SLC24A2;CHRNB2;RYR2;TRPC5;CHRNA7;NIPA1;CACNA1B;CACNA2D3;ATP2B3;HTR2C;[illegible]
calcium ion transport (GO:00	1,41E-06	7,64E-05	-2,2354425	21,19002334	CHRNB2;SLC24A2;RYR2;TRPC5;CHRNA7;CACNA1B;CACNA2D3;ATP2B3;HTR2C;ATP2B[illegible]
synapse organization (GO:00	8,02E-07	4,54E-05	-2,097279	20,97467809	ACHE;UNC13A;CHAT;SEZ6;WNT7A;ATP2B2;NRG1;SEZ6L2;SEZ6L;SNCG;SNCB;PAK3;CA[illegible]
action potential (GO:000150(	6,51E-07	3,85E-05	-2,0343078	20,67897433	CHRNB2;GLRA1;RYR2;KCNC2;SCN8A;CHRNA7;SCN9A;KCNE1L;FGF12;SCN4B;SCN1B;S[illegible]
divalent inorganic cation trar	2,00E-06	0,0001022	-2,249614	20,67074266	SLC24A2;CHRNB2;RYR2;TRPC5;CHRNA7;NIPA1;CACNA1B;CACNA2D3;ATP2B3;HTR2C;[illegible]
organic hydroxy compound b	1,79E-06	9,30E-05	-2,1676569	20,12293167	CDS1;IDI1;ACHE;PCYT1B;MVK;GOT1;HMGCS1;INSIG1;CHRNA7;CHAT;CYP51A1;MSM[illegible]
regulation of transporter acti	2,46E-06	0,00011863	-2,2060841	19,94187713	RYR2;NETO2;CABP1;ATP1B1;MAPK8IP2;SYNGR3;CACNG8;JPH3;GAL;CALM3;KCNE1L;[illegible]
nervous system developmen	2,19E-06	0,00010951	-2,1767508	19,85088469	PCSK2;CHRM2;ACHE;TRPC5;SRRM4;NRSN1;BEX1;ACTL6B;MYT1L;CPNE6;LIMK1;CABL[illegible]
regulation of neuron differer	3,21E-06	0,00014928	-2,2516725	19,83657626	RET;SNAP25;ACHE;ATP8A2;SEZ6;STMN2;KNDC1;THY1;BRINP2;CUX2;NCS1;NEFL;NEF[illegible]
neuron-neuron synaptic tran	7,72E-07	4,46E-05	-1,9649725	19,6832638	SLC6A5;NAPB;GLRA1;GRIN2A;SHC3;UNC13A;GLRB;GRIN2B;CDH8;GABRG2;GRIN1
regulation of ion transmemb	2,52E-06	0,00011901	-2,170906	19,61696414	RYR2;NETO2;CABP1;ATP1B1;MAPK8IP2;CACNG8;JPH3;GAL;CALM3;KCNE1L;CACNG2;[illegible]
regulation of transmembran	3,68E-06	0,00016792	-2,1797937	18,94686546	RYR2;NETO2;CABP1;ATP1B1;MAPK8IP2;CACNG8;JPH3;GAL;CALM3;KCNE1L;CACNG2;[illegible]
positive regulation of membr	7,00E-06	0,00029384	-2,3089492	18,7774709	RIMS2;STX1B;RIMS1;CUX2;NETO1;SHANK1;GRIN1
positive regulation of calciun	2,39E-06	0,00011736	-2,0607868	18,65070976	SCAMP5;GLRA1;STXBP1;SYT7;CDK5R2;CACNA1G
neuron projection morphoge	3,82E-06	0,00017115	-2,1350284	18,51704535	CHRNB2;BRSK1;ATP8A2;RAB3A;NYAP2;MAPK8IP2;MAP2;EPB41L3;BAI1;NEFL;LPPR4;[illegible]
neuron projection guidance (	1,12E-05	0,00042351	-2,2431106	17,4221192	MEG3;NCAN;CACNA1G;RND1;SHH;ABLIM2;KIF5C;TUBB3;KIF5A;PLXNA4;SCN1B;MNX1[illegible]
axon guidance (GO:0007411)	1,12E-05	0,00042351	-2,240499	17,40183524	MEG3;NCAN;CACNA1G;RND1;SHH;ABLIM2;KIF5C;TUBB3;KIF5A;PLXNA4;SCN1B;MNX1[illegible]
regulation of long-term neur	9,22E-06	0,00037467	-2,1935684	17,30607544	RIMS1;GRM5;KCNJ10;NETO1;NEURL1;SYP;GRIN1
synaptic vesicle transport (G(	5,16E-06	0,0002199	-2,0380994	17,16555696	RIMS1;SNAP25;PCLO;RAB3A;OTOF;AMPH;CPLX2;AP3B2;CPLX1;PACSIN1
establishment of synaptic ve	5,16E-06	0,0002199	-2,0340495	17,13144726	RIMS1;SNAP25;PCLO;RAB3A;OTOF;AMPH;CPLX2;AP3B2;CPLX1;PACSIN1
carboxylic acid transport (GC	9,77E-06	0,00039084	-2,1520098	16,88726619	SLC10A4;SNAP25;KCNJ10;RAB3A;SLC32A1;SLC6A15;STXBP1;SLC1A2;ACSL6;SLC7A14;[illegible]
organic acid transport (GO:0(	1,11E-05	0,00042351	-2,1551869	16,73922022	SLC10A4;SNAP25;KCNJ10;RAB3A;SLC32A1;SLC6A15;STXBP1;ACSL6;SLC7A14;SLC1A2;[illegible]
feeding behavior (GO:00076:	8,13E-06	0,00033579	-2,0546826	16,4354335	CHRNB2;UCHL1;ATP8A2;GAL;HRH3;HTR2C;TACR1;MCHR1;MRAP2;GRIN2B;HCRTR2;[illegible]
developmental maturation ((	1,15E-05	0,00042744	-2,1101952	16,37025963	RET;UNC13A;RAB3A;STXBP1;SEZ6;CEND1;SYP;SEZ6L2;SEZ6L;GRIN1;RND1;CABYR;CDK[illegible]
neuromuscular synaptic tran	1,64E-05	0,00057471	-2,1435176	15,99417356	CHRNB2;SLC5A7;RAB3A;EGR3;STXBP1;CHAT
positive regulation of exocyt	1,04E-05	0,0004085	-2,015027	15,72330935	SCAMP5;GLRA1;RAB3A;STXBP1;RAB3D;NCS1;CDK5R2;SYT7;CACNA1G
steroid biosynthetic process	1,57E-05	0,00055906	-2,0426671	15,29806834	IDI1;MVK;HMGCS1;SRD5A1;INSIG1;CYP51A1;MSMO1;DHCR24;HMGCR;HSD17B6;SQL[illegible]
glutamate receptor signaling	1,68E-05	0,00058152	-2,0269013	15,10013872	GRM3;GRM5;GRIN2A;GRM4;ATP1A3;GRIN2B;CDK5R1;GRIN1
establishment of vesicle loca	2,03E-05	0,00068532	-2,0671334	15,06036156	RIMS1;SNAP25;PCLO;RAB3A;KIF3A;OTOF;AMPH;CPLX2;AP3B2;CPLX1;DYNC1I1;PACSI[illegible]
regulation of cell projection (	3,44E-05	0,00111934	-2,2081689	15,00454957	RET;SNAP25;ACHE;ATP8A2;SEZ6;STMN2;KNDC1;THY1;CUX2;TBC1D30;NCS1;NEFL;NE[illegible]
cellular response to cAMP (G	2,93E-05	0,00097629	-2,1065918	14,60237917	SLC8A3;HCN4;EGR3;DMTN;STAR;GPD1;HCN2;HCN1
cell projection morphogenes	4,23E-05	0,00132485	-2,1722176	14,39410096	CHRNB2;BRSK1;ATP8A2;RAB3A;NYAP2;MAPK8IP2;MAP2;KIF3A;EPB41L3;NEFL;BAI1;L[illegible]
long-chain fatty-acyl-CoA me	3,95E-05	0,00125369	-2,1351741	14,26651179	ACLY;ACOT7;DGAT2;ELOVL4;FASN;ACSL6
associative learning (GO:000(	1,73E-05	0,00059364	-1,9129477	14,21175264	CHRNB2;GRIN2A;NETO1;GABRA5;CHRNA7;ATP1A3;TACR1;HMGCR;GRIN1;SHANK1
lipid biosynthetic process (G(	5,15E-05	0,00154009	-2,1695749	14,04998783	CDS1;IDI1;ACHE;MVK;FAM57B;INSIG1;CHAT;MSMO1;HSD17B6;HMGCR;PLA2G5;FAD[illegible]
regulation of calcium ion-dep	4,68E-05	0,00141494	-2,0140155	13,21328737	SCAMP5;GLRA1;SYT4;STXBP1;CDK5R2;SYT7;CACNA1G
chloride transmembrane trai	3,18E-05	0,00104624	-1,9187947	13,16783003	GABRA2;GABRB3;GLRA1;CLCN5;SLC12A5;GLRA3;GLRB;GABRA5;GABRA3;ANO4;GABR[illegible]
dicarboxylic acid transport (G	3,69E-05	0,00118482	-1,9073105	12,85176911	RIMS1;SNAP25;KCNJ10;RAB3A;STXBP1;SLC1A2;SLC17A6;SLC13A5;CPLX1
cell differentiation in hindbra	8,37E-05	0,00231534	-2,10319	12,76257899	CEND1;WNT7A;ATP2B2;KNDC1;FAIM2;CBLN1
cerebellar Purkinje cell layer	1,38E-05	0,00049945	-1,6650761	12,65790049	KLHL1;SEZ6;FAIM2;SEZ6L2;SEZ6L
organic anion transport (GO:	0,000103	0,0026711	-2,0923121	12,39750303	SLC10A4;SNAP25;ATP8A2;KCNJ10;RAB3A;SLC32A1;SLC6A15;STXBP1;ACSL6;SLC7A14;[illegible]
neurofilament cytoskeleton (	4,62E-06	0,00020359	-1,4465131	12,29451172	ATP8A2;NEFL;NEFM;NEFH;INA
cell part morphogenesis (GO	0,00013	0,00323186	-2,1436501	12,29318815	CHRNB2;BRSK1;ATP8A2;RAB3A;NYAP2;MAPK8IP2;MAP2;KIF3A;EPB41L3;NEFL;BAI1;L[illegible]
regulation of neurotransmitt	8,31E-05	0,00231534	-2,0214762	12,26672315	RAB3B;STX1B;NAPB;RIMS1;RAB3A;UNC13A;KCNC3;STXBP1;SNCG
axonogenesis (GO:0007409)	8,04E-05	0,00229808	-2,0174244	12,25722969	CHRNB2;BRSK1;ATP8A2;RAB3A;MAP2;BAI1;LPPR4;NPTX1;CTNNA2;PAK3;NEFH;PLXNA[illegible]
synaptic transmission, glutan	6,90E-05	0,00202247	-1,9494106	12,09304348	NAPB;GRIN2A;SHC3;UNC13A;GRIN2B;CDH8;GRIN1
membrane depolarization du	9,92E-05	0,00260656	-2,0247466	12,04668047	SCN8A;SCN9A;CACNA1B;SCN4B;SCN1B;CACNA1G;SCN1A
regulation of synapse organi:	9,43E-05	0,00256601	-1,9847515	11,8398429	CHRNB2;CUX2;FRMPD4;NCAN;NEURL1;WNT7A;CTNNA2;CBLN1;GRIN1
intermediate filament cytosk	8,30E-05	0,00231534	-1,940038	11,77253995	SHH;ATP8A2;NEFL;NEFM;INA;NEFH;PRPH
startle response (GO:000196	1,38E-05	0,00049945	-1,5426746	11,72740535	GLRA1;GRIN2A;GLRB;NPAS1;GRIN2B
amino acid transport (GO:00(	9,50E-05	0,00256601	-1,9656161	11,72569228	SNAP25;KCNJ10;RAB3A;SLC32A1;STXBP1;SLC6A15;SLC1A2;SLC7A14;SLC6A11;CPLX1;[illegible]
regulation of neurotransmitt	0,000105	0,00269804	-1,9793704	11,70842751	STX1B;NAPB;RIMS1;RAB3A;UNC13A;KCNC3;STXBP1;SNCG
chloride transport (GO:0006(	7,46E-05	0,00215467	-1,9055029	11,70000865	GABRA2;GABRB3;GLRA1;CLCN5;SLC12A5;GLRA3;GLRB;GABRA5;GABRA3;ANO4;GABR[illegible]
regulation of glutamate rece	0,000195	0,0046215	-2,1333362	11,47102487	CACNG8;NETO2;MAPK8IP2;CACNG2;CACNG3;SHANK1
intermediate filament-based	9,92E-05	0,00260656	-1,9232393	11,44273971	SHH;ATP8A2;NEFL;NEFM;NEFH;INA;PRPH
cochlea development (GO:00	6,92E-05	0,00202247	-1,777793	11,02842476	GABRB3;KCNK9;GABRA5;ATP2B2;KCNK3
fatty-acyl-CoA metabolic pro	0,00016	0,00387703	-1,9712344	10,94564318	ACLY;DGAT2;ACOT7;ELOVL4;FASN;ACSL6
anion transmembrane transp	0,000161	0,00387703	-1,9190914	10,65610973	GABRA2;GABRB3;SLC12A5;GABRA5;SLC6A15;GABRA3;SLC7A14;ANO4;GABRG2;GLRA[illegible]
triglyceride biosynthetic proc	0,000164	0,00391026	-1,9024278	10,54734726	ACLY;DGAT2;ELOVL4;FASN;GPD1;PNPLA3;ACSL6
vesicle docking involved in ex	0,00016	0,00387703	-1,8951225	10,523018	STX1B;SNAP25;UNC13A;SNPH;STXBP1;CPLX2
regulation of heart contracti	0,000234	0,00534808	-2,0063583	10,49529457	HCN4;CHRM2;RYR2;KCNJ12;CHRNA7;CACNA1B;ADRA1D;ATP1B1;CACNA1G;ADORA1;[illegible]
regulation of blood circulatio	0,000333	0,00721717	-2,1030573	10,37079012	HCN4;CHRM2;RYR2;KCNJ12;CHRNA7;CACNA1B;ADRA1D;HMGCR;HTR2A;TACR1;ATP1[illegible]
regulation of alpha-amino-3-	0,000223	0,00514084	-1,9410637	10,23045195	CACNG8;MAPK8IP2;CACNG2;CACNG3;SHANK1
neurotransmitter biosynthet	9,57E-05	0,00256601	-1,6936672	10,1034075	SLC5A7;ACHE;GAD1;CHAT;GAD2
glutamate secretion (GO:001	0,000129	0,00323186	-1,7534238	10,05535757	SNAP25;RIMS1;RAB3A;STXBP1;CPLX1
acylglycerol biosynthetic pro	0,000223	0,00514084	-1,9005711	10,01703408	ACLY;DGAT2;ELOVL4;FASN;GPD1;PNPLA3;ACSL6
response to cAMP (GO:0051!	0,000289	0,00645306	-1,9824759	9,998024851	HCN4;SLC8A3;DMTN;EGR3;STAR;PTPRN;GPD1;PLA2G5;HCN2;HCN1
neutral lipid biosynthetic pro	0,000223	0,00514084	-1,8951789	9,988614362	ACLY;DGAT2;ELOVL4;FASN;GPD1;PNPLA3;ACSL6
positive regulation of ion trai	0,000377	0,00796602	-2,0634185	9,97161596	CHRNB2;RAB3B;RYR2;ATP8A2;ACSL6;ATP2B2;THY1;ATP1B1;MCHR1;GAL;ADORA1;KC[illegible]
calcium ion-dependent exocy	0,000339	0,00727667	-2,0043098	9,867381855	RIMS2;RIMS1;GLRA1;SYT3;GLRB;TRIM36
regulation of dendrite develo	0,000264	0,00596644	-1,8915912	9,68798418	CHRNB2;ACHE;CUX2;NEURL1;SEZ6;KNDC1;GRIN1;CDK5R1;PACSIN1;SHANK1
cholesterol metabolic proces	0,00029	0,00645306	-1,8914873	9,539150731	IDI1;SQLE;MVK;HMGCS1;STAR;INSIG1;CYP51A1;MSMO1;DHCR24;DHCR7;HMGCR
response to purine-containin	0,000489	0,01001924	-2,0025968	9,218450024	HCN4;SLC8A3;RYR2;EGR3;DMTN;STAR;HMGCS1;PTPRN;GPD1;PLA2G5;HCN2;HCN1
inorganic anion transmembra	0,000315	0,00688201	-1,8235699	9,079270355	GABRA2;GABRB3;GLRA1;CLCN5;SLC12A5;GLRA3;GLRB;GABRA5;GABRA3;ANO4;GABR[illegible]
inorganic anion transport (G(	0,000344	0,00732678	-1,8426518	9,058880548	GABRA2;GABRB3;GLRA1;CLCN5;SLC12A5;GLRA3;GLRB;GABRA5;GABRA3;AQP6;ANO4[illegible]
membrane docking (GO:002:	0,000299	0,0065927	-1,7823127	8,950405443	STX1B;SNAP25;PEX5L;UNC13A;SNPH;STXBP1;CPLX2
regulation of synaptic transm	0,000394	0,00826851	-1,8417068	8,831539743	GRM3;GRM5;GRM4;UNC13A;ADORA1;HTR2A;MAPK8IP2
vesicle docking (GO:0048278	0,000401	0,0083446	-1,831456	8,765605497	STX1B;SNAP25;UNC13A;SNPH;STXBP1;CPLX2
regulation of axonogenesis ((	0,000502	0,01020436	-1,8614133	8,534468786	RET;ACHE;DAB1;LIMK1;WNT7A;NEFL;NEFM;THY1;PLXNA4;GRIN1;CDK5R1
steroid metabolic process (G	0,000711	0,01369385	-1,9684983	8,446448437	IDI1;MVK;HMGCS1;SRD5A1;INSIG1;CYP51A1;MSMO1;DHCR24;HMGCR;HSD17B6;SQ[illegible]
positive regulation of nervou	0,000924	0,01715865	-2,0641714	8,391378647	RET;ATP8A2;LIMK1;STMN2;ACSL6;SEZ6;NAP1L2;WNT7A;GRM5;BRINP2;SHH;CUX2;D[illegible]
membrane hyperpolarization	0,000677	0,01314959	-1,8418054	7,97753075	RIMS2;GLRA1;RIMS1;KCNJ10;CACNG2
dendrite development (GO:0	0,000472	0,00974761	-1,7208751	7,968913561	DAB1;MAP2;MCF2;CHAT;KLHL1;PAK3
sterol metabolic process (GO	0,000722	0,01381363	-1,8568552	7,951239294	IDI1;SQLE;MVK;STAR;HMGCS1;INSIG1;CYP51A1;MSMO1;DHCR24;DHCR7;HMGCR
neuron development (GO:00	0,00083	0,01564951	-1,9088513	7,935697003	GABRB3;ISL2;OLFM3;ONECUT2;GABRA5;MAP2;ACSL6;THY1;HOXD10;HCN1;PACSIN1
regulation of system process	0,001389	0,02360945	-2,0929514	7,840422781	HCN4;CHRM2;RYR2;KCNJ10;KCNJ12;CHRNA7;CACNA1B;OPRK1;HTR2C;ADRA1D;HMG[illegible]
visual behavior (GO:0007632	0,000512	0,01032419	-1,711667	7,827908576	CHRNB2;GRIN2A;NETO1;SLC1A2;ATP1A3;HMGCR;GRIN1
phospholipase C-activating G	0,00074	0,01404307	-1,8028799	7,690412161	CHRM2;GRM5;NPR3;HTR2C;OPRK1;HTR2A;TACR1
cytoskeleton-dependent intr	0,000918	0,01715865	-1,8745826	7,62065212	UCHL1;KLC2;KIF5C;KIF3A;KIF5A;NEFL;NEFM;KIF3C;AP3B2;DYNC1I1
small molecule biosynthetic (	0,001343	0,02313018	-2,0062027	7,556596871	CDS1;IDI1;ACHE;PCYT1B;ACOT7;MVK;HMGCS1;GOT1;ELOVL4;NCAN;INSIG1;GAD1;CY[illegible]
multicellular organismal resp	0,000645	0,01261126	-1,7172491	7,509814238	RET;GABRA5;SCN9A;CACNA1B;HTR2C;TACR1;MAPK8IP2;GRIN2B
gamma-aminobutyric acid sig	0,000555	0,01093909	-1,6624795	7,506780408	GABRA2;GABBR2;GABRA5;GABRA3;GABRG2
ionotropic glutamate recept(	0,000555	0,01093909	-1,6586514	7,48949475	GRIN2A;ATP1A3;GRIN2B;CDK5R1;GRIN1
central nervous system neun	0,000972	0,0178893	-1,8269786	7,350943828	ISL2;SHH;CEND1;CHD5;WNT7A;KNDC1;ATP2B2;FAIM2;CBLN1
positive regulation of neuron	0,001061	0,01916157	-1,8380857	7,269350488	RET;CUX2;ATP8A2;NEURL1;LIMK1;ACSL6;STMN2;SEZ6;NEFL;SCN1B;PACSIN1;SHANK1
anatomical structure matura	0,000555	0,01093909	-1,5965441	7,209054701	RET;CEND1;CDK5R2;CDK5R1;GRIN1
establishment of organelle lc	0,001324	0,02295462	-1,8827264	7,105854223	SNAP25;RAB3A;CPLX2;CPLX1;AP3B2;RIMS1;PCLO;KIF3A;SPIRE2;OTOF;AMPH;DYNC1I[illegible]
positive regulation of neurog	0,001716	0,02806615	-1,9874339	7,101480923	RET;ATP8A2;LIMK1;ACSL6;STMN2;SEZ6;NAP1L2;GRM5;BRINP2;SHH;CUX2;DAB1;STA[illegible]
cellular calcium ion homeost	0,001774	0,0283013	-1,9824462	7,067118208	SLC24A2;CHRM2;RYR2;CHRNA7;HTR2C;ATP2B2;TACR1;HTR2A;ATP1B1;MCHR1;GRIN[illegible]
neurotransmitter metabolic (	0,000981	0,0178893	-1,7052751	6,861263508	SLC5A7;ACHE;GAD1;CHAT;GAD2
positive regulation of neuron	0,001821	0,02835941	-1,8808684	6,70115141	RET;ATP8A2;LIMK1;SEZ6;ACSL6;STMN2;NAP1L2;BRINP2;CUX2;DAB1;NEURL1;NEFL;S[illegible]
microtubule-based process ((	0,002393	0,03496552	-1,9905937	6,675242362	AP3B2;TUBA4A;CAMSAP3;UCHL1;KLC2;TUBB2A;CABYR;KIF5C;TUBB3;MAP2;KIF3A;KI[illegible]
coenzyme metabolic process	0,001883	0,02915303	-1,8668034	6,599516905	ACOT7;DGAT2;MVK;MDH1;ELOVL4;ACSL6;HMGCR;NMNAT2;QDPR;ACLY;FOLH1;FAS[illegible]
cytosolic calcium ion homeo:	0,002009	0,03073514	-1,8872274	6,571984018	CHRM2;RYR2;HTR2C;ATP2B2;TACR1;HTR2A;MCHR1;SLC8A3;CALB2;JPH3;HRH3;PVAL[illegible]
nitrogen compound transpor	0,002531	0,03586581	-1,9317848	6,428923748	SNAP25;RAB3A;SLC35F3;STXBP1;EIF5A2;AQP6;SLC1A2;SLC7A14;CPLX1;RIMS2;RIMS1;[illegible]
acylglycerol metabolic proce:	0,001563	0,02639185	-1,753574	6,373715792	ACLY;DGAT2;ELOVL4;FASN;INSIG1;GPD1;ACSL6;PNPLA3;DGKK
alcohol metabolic process (G	0,002466	0,03582633	-1,9047864	6,341171264	CDS1;IDI1;ACHE;PCYT1B;DGAT2;MVK;HMGCS1;GOT1;INSIG1;CYP51A1;CHAT;MSMO1[illegible]
cellular divalent inorganic ca	0,002629	0,03695796	-1,9196437	6,330935457	SLC24A2;CHRM2;RYR2;CHRNA7;HTR2C;ATP2B2;TACR1;HTR2A;ATP1B1;MCHR1;GRIN[illegible]
response to alcohol (GO:009	0,002944	0,04051294	-1,9672746	6,307345949	CHRNB2;HMGCS1;CHRNA7;WNT7A;HMGCR;TACR1;HTR5A;SSTR3;GRIN2B;GRIN1;GRI[illegible]
regulation of metal ion trans	0,002844	0,03977571	-1,9549471	6,303724733	RYR2;CACNA1B;ATP2B2;HTR2A;THY1;CABP1;ATP1B1;MCHR1;JPH3;GAL;ADORA1;CAL[illegible]
neutral lipid metabolic proce	0,001684	0,02772926	-1,7527964	6,284243274	ACLY;DGAT2;ELOVL4;INSIG1;FASN;GPD1;ACSL6;PNPLA3;DGKK
calcium ion homeostasis (GO	0,002944	0,04051294	-1,9213935	6,160244841	SLC24A2;CHRM2;RYR2;CHRNA7;HTR2C;ATP2B2;TACR1;HTR2A;ATP1B1;MCHR1;GRIN[illegible]
rhythmic behavior (GO:0007(	0,002171	0,03263803	-1,7904841	6,127532879	CHRNB2;STAR;CHAT;ADORA1;HCRTR2
response to organophosphor	0,002389	0,03496552	-1,8188482	6,099312354	HCN4;SLC8A3;EGR3;DMTN;STAR;PTPRN;GPD1;PLA2G5;HCN2;HCN1
multi-organism behavior (GC	0,001788	0,02835147	-1,6975948	6,04866017	CHRNB2;SNCG;TACR1;MAPK8IP2;NPAS1;GRIN2B;GRIN1;SHANK1
cardiac muscle cell action po	0,001376	0,02353812	-1,6107739	6,039007449	RYR2;KCNE1L;FGF12;SCN4B;SCN1B
neuron projection developm	0,002272	0,03396347	-1,7577689	5,945600078	DAB1;MAP2;MCF2;CHAT;KLHL1;CCDC64;NEFL;NCDN;PAK3;NEFH;CDK5R1
cellular chemical homeostasi	0,003672	0,04948381	-1,9605446	5,893612229	SLC24A2;CHRM2;RYR2;DGAT2;SLC12A5;CHRNA7;ATP1A3;HTR2C;ATP2B2;TACR1;HTR[illegible]
visual learning (GO:0008542)	0,001646	0,02726357	-1,6328489	5,88185483	CHRNB2;GRIN2A;NETO1;ATP1A3;HMGCR;GRIN1
regulation of amine transpor	0,001748	0,02806615	-1,6375902	5,851422531	CHRNB2;RAB3B;HRH3;CHRNA7;ADORA1;SNCG;HTR2A
acyl-CoA metabolic process (	0,001748	0,02806615	-1,6225776	5,797779587	ACLY;ACOT7;MVK;DGAT2;ELOVL4;FASN;ACSL6
thioester metabolic process (	0,001748	0,02806615	-1,6192297	5,785816928	ACLY;DGAT2;ACOT7;MVK;ELOVL4;FASN;ACSL6
cardiac muscle cell action po	0,001612	0,02686974	-1,5969405	5,775741656	RYR2;KCNE1L;SCN4B;FGF12;SCN1B
organic hydroxy compound r	0,003527	0,04777881	-1,8789245	5,714134535	CDS1;IDI1;ACHE;MVK;INSIG1;CHRNA7;CHAT;MSMO1;PLA2G5;HMGCR;SDR16C5;GRI[illegible]
cofactor metabolic process ((	0,003455	0,04704374	-1,8551656	5,670643031	ACOT7;DGAT2;MVK;MDH1;ELOVL4;ACSL6;HMGCR;NMNAT2;ANK1;SPTB;QDPR;ACLY;[illegible]
plasma membrane organizat	0,002872	0,03994106	-1,7236375	5,550716749	EHD3;RAB26;EPB41L3;AHNAK2;ATP1B1;SPTB;ANK1;SYT7;GPR158;PACSIN1
regulation of cation channel	0,002118	0,03221034	-1,6114597	5,536117901	RYR2;JPH3;GAL;CALM3;KCNE1L;CABP1;FGF12
response to pain (GO:00482(	0,002171	0,03263803	-1,6141869	5,524194942	RET;SCN9A;CACNA1B;TACR1;GRIN2B
acid secretion (GO:0046717)	0,002326	0,03456706	-1,6325113	5,493162359	RIMS1;SNAP25;RAB3A;STXBP1;PLA2G5;CPLX1
innervation (GO:0060384)	0,000984	0,0178893	-1,3018726	5,238152374	RET;GABRB3;UNC13A;GABRA5
regulation of vesicle fusion ((	0,001257	0,02193768	-1,3461566	5,141712023	SYT4;SYT3;RAB3A;STXBP1
response to ammonium ion (	0,003035	0,04155229	-1,6106775	5,123247516	CHRNB2;GABRB3;SNCG;HTR2A;TACR1;GABRG2;GRIN1
long-chain fatty-acyl-CoA bio	0,001257	0,02193768	-1,3329725	5,091354587	ACLY;ELOVL4;FASN;ACSL6
synaptic transmission, cholin	0,001257	0,02193768	-1,2574679	4,802960924	CHRNB2;SLC5A7;ACHE;CHRNA7
regulation of synaptic vesicle	0,001579	0,02649366	-1,3110068	4,760068542	STX1B;NAPB;RAB3A;STXBP1
regulation of catecholamine	0,003694	0,04952284	-1,5580101	4,682320785	CHRNB2;HRH3;CHRNA7;SNCG;HTR2A
intermediate filament organi	0,001257	0,02193768	-1,2204302	4,661493656	SHH;NEFL;NEFM;NEFH
fatty-acyl-CoA biosynthetic p	0,001954	0,03007545	-1,2694833	4,448328034	ACLY;ELOVL4;FASN;ACSL6
circadian sleep/wake cycle p(	0,002387	0,03496552	-1,3112819	4,397243279	CHRNB2;STAR;ADORA1;HCRTR2
cerebellar granule cell differe	0,00013	0,00323186	-0,6185099	3,546968359	WNT7A;ATP2B2;KNDC1;CBLN1
regulation of short-term neu	0,00013	0,00323186	-0,6162249	3,533864403	RAB3A;UNC13A;SYP;PPFIA3
synaptic vesicle docking invo	4,56E-05	0,00139434	-0,2790215	1,834659097	SNAP25;STX1B;UNC13A;SNPH
synaptic vesicle maturation (	4,56E-05	0,00139434	-0,2665427	1,752606899	RAB3A;UNC13A;STXBP1;SYP
regulation of phospholipid bi	0,002537	0,03586581	0,81275828	-2,70483593	FABP3;HTR2C;HTR2A
cell migration in hindbrain (G	0,002537	0,03586581	0,87923928	-2,926082781	DAB1;CEND1;CTNNA2
neuromuscular process contr	0,002537	0,03586581	0,90823394	-3,022576172	GLRA1;ATP8A2;SCN1A
peripheral nervous system n(	0,002537	0,03586581	0,92857266	-3,090262812	ISL2;ONECUT2;HOXD10
regulation of synapse structu	0,001815	0,02835941	1,02368324	-3,647174997	FRMPD4;NCAN;CTNNA2
L-amino acid import (GO:004	0,001815	0,02835941	1,22682062	-4,370912152	SLC6A5;KCNJ10;SLC1A2
righting reflex (GO:0060013)	0,001237	0,02193768	1,31019225	-5,004344326	GLRA1;GLRB;SHANK1

Supplementary Table 5

	Neuron Specific Genes Enriched in DOWN Genes		
**Gene Symbol**	**Gene name**	**HGNC ID**	**Location**
NEFM	neurofilament medium	HGNC:7734	8p21.2
NEFL	neurofilament light	HGNC:7739	8p21.2
STMN2	stathmin 2	HGNC:10577	8q21.13
GABRA5	gamma-aminobutyric acid type A rece	HGNC:4079	15q12
SLC12A5	solute carrier family 12 member 5	HGNC:13818	20q13.12
GABRG2	gamma-aminobutyric acid type A rece	HGNC:4087	5q34
SNAP25	synaptosome associated protein 25	HGNC:11132	20p12.2
RBFOX3	RNA binding protein, fox-1 homolog 3	HGNC:27097	17q25.3
PCSK2	proprotein convertase subtilisin/kexin	HGNC:8744	20p12.1
SYT4	synaptotagmin 4	HGNC:11512	18q12.3
TMEM130	transmembrane protein 130	HGNC:25429	7q22.1
VSNL1	visinin like 1	HGNC:12722	2p24.2
SLC17A6	solute carrier family 17 member 6	HGNC:16703	11p14.3
HTR2C	5-hydroxytryptamine receptor 2C	HGNC:5295	Xq23
L1CAM	L1 cell adhesion molecule	HGNC:6470	Xq28
MYT1L	myelin transcription factor 1 like	HGNC:7623	2p25.3
SV2B	synaptic vesicle glycoprotein 2B	HGNC:16874	15q26.1
RGS4	regulator of G-protein signaling 4	HGNC:10000	1q23.3
CLSTN2	calsyntenin 2	HGNC:17448	3q23
NAPB	NSF attachment protein beta	HGNC:15751	20p11.21
KCNC2	potassium voltage-gated channel subf	HGNC:6234	12q21.1
CACNA1B	calcium voltage-gated channel subuni	HGNC:1389	9q34.3
CDH8	cadherin 8	HGNC:1767	16q21

Supplementary Table 6

Legend:

**GeneName**	Gene Name
**baseMean**	Mean expression in both groups
**baseMeanCtl**	Mean expression in control group
**baseMeanAls**	Mean expression in ALS group
**foldChange**	Fold change
**log2FoldChange**	Log2 fold change
**pval**	P value
**padj**	Correted P value

GeneName	baseMean	baseMeanCtl	baseMeanAls	foldChange	log2FoldChar	pval	padj
12S.rRNA	1032316,66	758737,941	1305895,38	1,72114153	0,78336573	0,02218235	0,2883706
16S.rRNA	2565064,02	2358930,92	2771197,13	1,17476824	0,23237617	0,24832406	0,64564254
ATPase8-6	925853,373	867908,472	983798,274	1,13352768	0,18081962	0,4421054	0,85020855
CO1	1284869,71	1304207,55	1265531,87	0,97034545	-0,0434296	0,82276645	0,89133032
CO2	950723,74	921553,429	979894,051	1,06330683	0,08855796	0,71940723	0,85020855
CO3	275268,382	297111,913	253424,851	0,85296092	-0,2294485	0,46938462	0,85020855
Cytb	622836,737	600451,628	645221,845	1,07456091	0,10374726	0,64227188	0,85020855
ND1	703389,9	815711,717	591068,083	0,72460413	-0,4647351	0,05850817	0,38030307
ND2	1080090,34	1125586,64	1034594,04	0,91915985	-0,1216123	0,63444419	0,85020855
ND3	324768,749	321990,358	327547,139	1,0172576	0,02468506	0,89480768	0,89480768
ND4L-4	1666869,9	1725820,51	1607919,29	0,93168396	-0,1020874	0,65797499	0,85020855
ND5	967118,247	835203,899	1099032,59	1,31588537	0,39603382	0,1634698	0,57537214
ND6	142917,01	102251,491	183582,529	1,79540198	0,84430689	0,17703758	0,57537214

Supplementary Table 7

Canonical Pathways For DOWN Genes

Ingenuity Canonical Pathways	-log(p-value)	Ratio	z-score	Molecules
Superpathway of Cholesterol Bi	7,57	0,40	NaN	HMGCS1,SQLE,IDI1,DHCR24,MVK,ACAT2,HMGCR,CYP51A1,MSMO1,DHCR7
GABA Receptor Signaling	6,83	0,23	NaN	SLC32A1,GAD1,GABRG2,DNM1,NSF,GABRB3,GABRA2,SLC6A11,KCNN1,KCNQ2,GABBR2,GAD2,GABRA5,GABRA3
Mevalonate Pathway I	4,58	0,50	NaN	HMGCS1,IDI1,MVK,ACAT2,HMGCR
Superpathway of Geranylgerany	4,11	0,42	NaN	HMGCS1,IDI1,MVK,ACAT2,HMGCR
Cholesterol Biosynthesis I	3,92	0,39	NaN	SQLE,DHCR24,CYP51A1,MSMO1,DHCR7
Cholesterol Biosynthesis II (via 2	3,92	0,39	NaN	SQLE,DHCR24,CYP51A1,MSMO1,DHCR7
Cholesterol Biosynthesis III (via	3,92	0,39	NaN	SQLE,DHCR24,CYP51A1,MSMO1,DHCR7
Glutamate Receptor Signaling	3,33	0,16	NaN	CALM1 (includes others),GRIN2A,GRM4,SLC17A6,SLC1A2,GRM5,GRIN1,GRIN2B,GRM3
Gαi Signaling	2,93	0,11	-1,941	OPRK1,RGS7,SSTR3,CHRM2,NPR3,GRM3,GABBR2,GRM4,HRH3,PRKAR1B,RGS4,PTGER3,ADORA1
Glutamate Dependent Acid Resi	2,76	1,00	NaN	GAD1,GAD2
Calcium Signaling	2,62	0,10	-3,162	ATP2B3,CALM1 (includes others),TRPC5,CHRNA7,CAMKK1,RCAN2,GRIN1,GRIN2B,ATP2B2,SLC8A3,GRIN2A,CHRNB2,PRKAR1B,RYR2
Remodeling of Epithelial Adhere	2,12	0,13	NaN	TUBB2A,CTNNA2,TUBB3,TUBA8,TUBA4A,DNM1,MAPRE3
Neuropathic Pain Signaling In D	2,06	0,10	-3,162	KCNN1,KCNQ2,TACR1,GRIN2A,GRM4,PRKAR1B,GRM5,GRIN1,GRIN2B,GRM3
Dopamine-DARPP32 Feedback	1,92	0,09	-1,508	PPP1R14C,CALM1 (includes others),GRIN2A,GUCY1A2,PRKAR1B,CAMKK1,PPP2R2C,KCNJ10,CALY,KCNJ16,GRIN1,GRIN2B
Glutamate Degradation III (via 4	1,80	0,40	NaN	GAD1,GAD2
Aspartate Degradation II	1,80	0,40	NaN	GOT1,MDH1
G-Protein Coupled Receptor Sig	1,79	0,07	NaN	OPRK1,RGS7,ADRA1D,SSTR3,CHRM2,NPR3,GRM3,GABBR2,GRM4,HRH3,PRKAR1B,HTR2C,GRM5,HTR5A,RGS4,PTGER3,ADORA1,HTR2A
Dopamine Receptor Signaling	1,67	0,10	NaN	PPP1R14C,NCS1,PRKAR1B,PPP2R2C,CALY,SLC18A3,QDPR
cAMP-mediated signaling	1,66	0,07	-2,324	OPRK1,CALM1 (includes others),RGS7,SSTR3,CHRM2,NPR3,GRM3,GABBR2,GRM4,HRH3,PRKAR1B,HTR5A,RGS4,PTGER3,ADORA1
Serotonin Receptor Signaling	1,65	0,13	NaN	HTR2C,HTR5A,SLC18A3,QDPR,HTR2A
Zymosterol Biosynthesis	1,63	0,33	NaN	CYP51A1,MSMO1
Synaptic Long Term Potentiatio	1,60	0,09	-2,333	PPP1R14C,CALM1 (includes others),GRIN2A,GRM4,PRKAR1B,GRM5,GRIN1,GRIN2B,GRM3
Amyotrophic Lateral Sclerosis S	1,60	0,09	NaN	NEFH,GRIN2A,NEFM,SLC1A2,PRPH,GRIN1,HECW1,NEFL,GRIN2B
Gluconeogenesis I	1,43	0,17	NaN	ENO2,ME1,MDH1
Stearate Biosynthesis I (Animals	1,41	0,13	NaN	DHCR24,FASN,ACOT7,ACSL6
Ketogenesis	1,39	0,25	NaN	HMGCS1,ACAT2
Acetyl-CoA Biosynthesis III (fror	1,38	1,00	NaN	ACLY
Palmitate Biosynthesis I (Animal	1,38	1,00	NaN	FASN
L-cysteine Degradation III	1,38	1,00	NaN	GOT1
Fatty Acid Biosynthesis Initiatior	1,38	1,00	NaN	FASN
Cellular Effects of Sildenafil (Via	1,35	0,08	NaN	CACNG2,KCNN1,KCNQ2,CACNG8,CALM1 (includes others),GUCY1A2,PRKAR1B,CACNG3

Canonical Pathways For UP Genes

Ingenuity Canonical Pathways	-log(p-value)	Ratio	z-score	Molecules
TREM1 Signaling	10,30	0,29	3,742	TLR5,TLR7,MYD88,TLR1,CASP1,TLR8,IL18,FCGR2B,TYROBP,CASP5,ITGA5,TLR2,CD86,NLRC4
Complement System	10,00	0,48	1,667	C1QC,C3,CFI,C5AR1,C1QB,CFD,C1R,SERPING1,C3AR1,C1QA
Role of Pattern Recognition Rec	9,10	0,17	3,606	TLR5,TLR7,C3,MYD88,TLR1,C1QB,CASP1,C3AR1,C1QA,PIK3CG,TLR8,C1QC,IL18,SYK,C5AR1,TLR2,CLEC7A,NLRC4
LXR/RXR Activation	8,95	0,20	-1,291	C3,TNFRSF1A,SERPINF1,CD14,TNFRSF1B,ABCA1,MSR1,IL1R2,RBP4,TNFRSF11B,IL18,LY96,IL1R1,IL18RAP,APOE,APOL1
Hepatic Fibrosis / Hepatic Stella	8,86	0,14	NaN	IGFBP4,COL4A1,TNFRSF1A,CD14,TNFRSF1B,CTGF,IGFBP5,IL1R2,LEPR,IGF1,TNFRSF11B,CCR5,LY96,IL1R1,IL18RAP,EDN1,IL4R,COL2A1,IGFBP3,TIMP1,HGF
Granulocyte Adhesion and Diap	7,97	0,16	NaN	TNFRSF1A,TNFRSF1B,CSF3R,FPR2,HRH1,IL1R2,FPR1,CDH5,TNFRSF11B,IL18,IL1R1,C5AR1,IL18RAP,CXCL16,ITGA5,PECAM1,HRH2
Acute Phase Response Signalir	7,61	0,14	3,742	C3,MYD88,TNFRSF1A,SERPINF1,CP,TNFRSF1B,SERPINA3,RBP4,C1R,PIK3CG,OSMR,TNFRSF11B,SOD2,IL18,IL1R1,CEBPB,SERPING1,HAMP
Dendritic Cell Maturation	7,61	0,14	4	MYD88,TNFRSF1A,FCGR1A,TNFRSF1B,FCER1G,LEPR,TREM2,FCGR2A,PIK3CG,TNFRSF11B,IL18,FCGR2B,CD1D,TYROBP,COL2A1,HLA-C,TLR2,CD86
Phagosome Formation	6,92	0,17	NaN	TLR5,TLR7,FCGR1A,TLR1,MSR1,FCER1G,FCGR2A,PIK3CG,TLR8,SYK,FCGR2B,ITGA5,TLR2,CLEC7A
Communication between Innate	5,91	0,20	NaN	TLR8,IL18,TLR5,TNFSF13B,TLR7,TLR1,HLA-C,FCER1G,TLR2,CD86
LPS/IL-1 Mediated Inhibition of I	5,77	0,11	1,732	MYD88,ABCC2,FABP4,TNFRSF1A,CD14,TNFRSF1B,ABCA1,IL1R2,SULT1B1,TNFRSF11B,IL18,LY96,IL1R1,ACSL5,IL18RAP,APOE,ABCC3
Role of Macrophages, Fibroblas	5,65	0,09	NaN	TLR5,TNFSF13B,CEBPD,TLR7,MYD88,TNFRSF1A,FCGR1A,TLR1,TNFRSF1B,IL1R2,IRAK3,PIK3CG,TNFRSF11B,TLR8,IL18,IL1R1,C5AR1,CEBPB,IL18RAP,SFRP2,TLR2,IL18R1
IL-10 Signaling	5,51	0,18	NaN	CCR5,IL18,FCGR2B,IL1R1,CCR1,CD14,IL18RAP,IL4R,IL1R2,FCGR2A
Inflammasome pathway	5,29	0,35	2,449	IL18,MYD88,CASP1,CASP5,NLRC4,PYCARD
NF-κB Signaling	5,24	0,11	3,5	TLR5,TNFSF13B,TLR7,MYD88,TNFRSF1A,TLR1,TNFRSF1B,IL1R2,FCER1G,IRAK3,PIK3CG,TNFRSF11B,TLR8,IL18,IL1R1,TLR2
Toll-like Receptor Signaling	4,89	0,16	2,828	TLR8,IL18,LY96,TLR5,TLR7,MYD88,TLR1,CD14,TLR2,IRAK3
T Helper Cell Differentiation	4,76	0,17	NaN	TNFRSF11B,IL18,TNFRSF1A,TNFRSF1B,IL4R,FCER1G,CD86,IL2RG,IL18R1
Hepatic Cholestasis	4,63	0,11	NaN	MYD88,ABCC2,TNFRSF1A,CD14,TNFRSF1B,IL1R2,IRAK3,TNFRSF11B,LY96,IL18,IL1R1,IL18RAP,ABCC3,ESR1
Altered T Cell and B Cell Signali	4,56	0,16	NaN	TLR8,IL18,TLR5,TNFSF13B,TLR7,TLR1,FCER1G,TLR2,CD86
Eicosanoid Signaling	4,16	0,16	2	ALOX5AP,FPR2,PLA2G7,PLB1,PTGIR,PTGDR,PTGS1,ALOX5
Systemic Lupus Erythematosus	4,04	0,10	NaN	TNFSF13B,TLR7,FCGR1A,BDKRB2,FCER1G,FCGR2A,PIK3CG,IL18,FCGR2B,HLA-C,PTPRC,CD86,LYN
Crosstalk between Dendritic Cel	4,03	0,16	NaN	IL18,TLR7,TNFRSF1B,TYROBP,HLA-C,TREM2,CD86,IL2RG
Th1 and Th2 Activation Pathway	3,29	0,09	NaN	CCR5,LGALS9,IL18,CCR1,IL4R,SPI1,CD86,IL2RG,HAVCR2,IKZF1,PIK3CG,IL18R1
Atherosclerosis Signaling	3,18	0,11	NaN	IL18,COL2A1,APOE,MSR1,PLA2G7,RBP4,PLB1,APOL1,ALOX5
B Cell Development	3,03	0,25	NaN	SPN,IL7R,PTPRC,CD86
Agranulocyte Adhesion and Diap	3,03	0,09	NaN	IL18,IL1R1,C5AR1,TNFRSF1A,CXCR1,CXCL16,HRH1,ITGA5,PECAM1,CDH5
Fcγ Receptor-mediated Phagoc	2,87	0,11	2,828	SYK,HCK,FCGR1A,FGR,RAC2,FCGR2A,PIK3CG,LYN
Production of Nitric Oxide and R	2,86	0,08	3,317	TNFRSF11B,TNFRSF1A,TNFRSF1B,APOE,SPI1,RBP4,CYBB,TLR2,CYBA,APOL1,PIK3CG,NCF2
IL-6 Signaling	2,82	0,09	3	TNFRSF11B,IL18,IL1R1,TNFRSF1A,CD14,TNFRSF1B,CEBPB,IL18RAP,IL1R2,PIK3CG
Primary Immunodeficiency Signa	2,73	0,21	NaN	IL7R,BTK,PTPRC,IL2RG
FXR/RXR Activation	2,72	0,10	NaN	IL18,C3,ABCC2,SERPINF1,FBP1,APOE,RBP4,APOL1
PPAR Signaling	2,72	0,10	-2,121	TNFRSF11B,IL18,IL1R1,TNFRSF1A,TNFRSF1B,IL18RAP,IL1R2,CITED2
Role of Osteoblasts, Osteoclasts	2,60	0,07	NaN	TNFRSF1A,TNFRSF1B,IL1R2,IGF1,PIK3CG,TNFRSF11B,ALPL,IL18,IL1R1,IL18RAP,SFRP2,ITGA5,IL18R1
Type I Diabetes Mellitus Signalir	2,49	0,09	2,449	TNFRSF11B,MYD88,IL1R1,TNFRSF1A,TNFRSF1B,HLA-C,FCER1G,CD86
IL-12 Signaling and Production i	2,41	0,08	NaN	IL18,MYD88,CEBPB,APOE,SPI1,RBP4,TLR2,APOL1,PIK3CG
Graft-versus-Host Disease Signa	2,22	0,15	NaN	IL18,HLA-C,FCER1G,CD86
Leukocyte Extravasation Signali	2,19	0,07	NaN	SPN,BTK,TIMP1,RAC2,CYBB,CYBA,ITGA5,PECAM1,CDH5,PIK3CG,NCF2
Citrulline-Nitric Oxide Cycle	2,11	0,40	NaN	ASS1,NOS3
PI3K Signaling in B Lymphocyte	2,04	0,08	2,121	SYK,FCGR2B,C3,BTK,IL4R,PTPRC,PIK3CG,LYN
FcγRIIB Signaling in B Lymphoc	2,00	0,11	2	SYK,FCGR2B,BTK,PIK3CG,LYN
B Cell Receptor Signaling	1,97	0,07	2,646	SYK,FCGR2B,BCL2A1,BTK,PTPRC,RAC2,POU2F2,FCGR2A,PIK3CG,LYN
Leukotriene Biosynthesis	1,94	0,33	NaN	GGT5,ALOX5
GM-CSF Signaling	1,94	0,09	1,633	HCK,BCL2A1,RUNX1,CISH,PIK3CG,LYN
Th2 Pathway	1,90	0,07	1,134	CCR5,CCR1,IL4R,SPI1,CD86,IL2RG,IKZF1,PIK3CG
Endothelin-1 Signaling	1,89	0,07	3,162	EDN1,CASP1,CASP5,NOS3,PLA2G7,PLB1,CASP4,PTGS1,CASP7,PIK3CG
Cytotoxic T Lymphocyte-mediat	1,73	0,15	NaN	HLA-C,FCER1G,CASP7
p38 MAPK Signaling	1,71	0,07	2,646	IL18,IL1R1,TNFRSF1A,TNFRSF1B,IL18RAP,IL1R2,IRAK3
IGF-1 Signaling	1,69	0,07	NaN	IGFBP4,CTGF,IGFBP3,IGFBP5,NOV,IGF1,PIK3CG
Th1 Pathway	1,69	0,07	2,236	CCR5,LGALS9,IL18,CD86,HAVCR2,PIK3CG,IL18R1
Pathogenesis of Multiple Scleros	1,69	0,25	NaN	CCR5,CCR1
Role of Hypercytokinemia/hyper	1,67	0,14	NaN	CCR5,IL18,CCR1
Role of NFAT in Regulation of th	1,65	0,06	3	SYK,FCGR2B,FCGR1A,BTK,FCER1G,CD86,FCGR2A,PIK3CG,LYN
iNOS Signaling	1,60	0,10	2	LY96,MYD88,CD14,IRAK3
Role of JAK1 and JAK3 in γc Cy	1,60	0,09	NaN	SYK,IL7R,IL4R,IL2RG,PIK3CG
Guanosine Nucleotides Degrada	1,59	0,22	NaN	AOX1,GDA
Coagulation System	1,56	0,13	NaN	SERPINA5,BDKRB2,F13A1
Antigen Presentation Pathway	1,56	0,13	NaN	CD74,HLA-C,MR1
Natural Killer Cell Signaling	1,56	0,08	NaN	SYK,TYROBP,FCER1G,RAC2,FCGR2A,PIK3CG
Reelin Signaling in Neurons	1,56	0,08	NaN	HCK,FGR,APOE,ITGA5,PIK3CG,LYN
Allograft Rejection Signaling	1,47	0,12	NaN	HLA-C,FCER1G,CD86
Autoimmune Thyroid Disease Si	1,47	0,12	NaN	HLA-C,FCER1G,CD86
MIF-mediated Glucocorticoid Re	1,47	0,12	NaN	LY96,CD74,CD14
Superpathway of Citrulline Meta	1,42	0,18	NaN	ASS1,NOS3

Supplementary Table 8

Diseases and Bio Functions For DOWN Genes

Categories	Functions	Diseases or F	p-Value	Molecules	# Molecules
Neurological D	schizophrenia	Schizophrenia	1,72E-22	SYT7,GRIN2A	48
Hereditary Dis	Huntington dis	Huntington's D	0,0000022	RBFOX1,GAB	23
Neurological D	sporadic amyo	sporadic amyo	0,0000465	PRPH,NEFL,S	5
Neurological D	movement dis	Movement Dis	0,0000895	RBFOX1,GAB	26
Neurological D	disorder of bas	disorder of bas	0,0000895	RBFOX1,GAB	26
Neurological D	neuromuscula	neuromuscula	0,000359	RBFOX1,GAB	26
Neurological D	pervasive deve	pervasive deve	0,00114	NEFL,CPLX2,	5
Neurological D	bipolar disorde	bipolar disorde	0,00138	ATP1A3,GRIN	7
Metabolic Dise	Alzheimer dise	Alzheimer dise	0,0039	GRIN1,STXBP	18
Psychological	major affective	major affective	0,0162	NRG1,ATP1A3	11
Cell Morpholog	axonogenesis	axonogenesis	0,037	WNT7A,ADGR	3

Diseases and Bio Functions For UP Genes

Categories	Functions	Diseases or F	p-Value	Molecules	# Molecules
Neurological D	tauopathy	tauopathy	0,000559	APOE,C3,PTG	16
Neurological D	chronic fatigue	chronic fatigue	0,00119	C3,SERPINF1	4
Metabolic Dise	Alzheimer dise	Alzheimer dise	0,00138	APOE,C3,PTG	15
Neurological D	movement dis	Movement Dis	0,00616	APOE,C3,VIM	16
Neurological D	disorder of bas	disorder of bas	0,00616	APOE,C3,VIM	16
Hereditary Dis	Huntington dis	Huntington's D	0,0066	APOE,CYP26I	12
Cellular Mover	cell movement	cell movement	0,00998	IGF1,HGF,SEI	3
Neurological D	neuromuscula	neuromuscula	0,0135	APOE,C3,HAV	16
Cell Death and	cell viability	cell viability of	0,0139	CCR1,APOE,I	3
Nervous Syste	quantity	quantity of neu	0,0213	IGF1,HGF,ESI	3
Cellular Growt	proliferation	proliferation of	0,0219	CCR1,APOE,I	6
Cell Death and	cell viability	cell viability	0,0272	CCR1,APOE,I	4
Cellular Devel	outgrowth	outgrowth of n	0,0369	APOE,HGF,ES	4
Cell Death and	cell viability	cell viability of	0,0496	APOE,IGF1,H	3

Supplementary Table 9

Canonical Pathways For Differentially Spiced Genes

Ingenuity Canonical Pathways	-log(p-value)	Ratio	Molecules
Actin Cytoskeleton Signaling	4,51	0,11	FGFR2,DIAPH1,ACTR2,VCL,MYH10,ABI2,PTK2,APC,PAK3,MYH14,FN1,ARHGEF7,GSN,MYLK,MYH11,CYFIP2,SSH2,RAC1,NCKAP1
Clathrin-mediated Endocytosis Signaling	3,67	0,10	FGFR2,SNAP91,ACTR2,CTTN,AAK1,DNM1,PPP3CC,AP1B1,DNM1L,PICALM,CLTA,SH3GLB1,APOE,CLTB,AP2M1,RAC1
Calcium Signaling	3,19	0,10	ATP2B4,CAMK2D,MYH10,ATP2B1,PPP3CC,CALR,TPM3,ATP2B2,MYH14,CAMK2G,MYH11,ITPR1,ASPH,GRIA4
Integrin Signaling	2,78	0,09	FGFR2,ACTR2,ITGAV,VCL,CTTN,ITGA10,PTK2,PAK3,ARHGEF7,MAP2K4,GSN,MYLK,RAPGEF1,RAC1,TNK2
Lipid Antigen Presentation by CD1	2,66	0,25	CALR,PSAP,AP2M1,AP1B1
GNRH Signaling	2,49	0,09	DNM1L,GNAS,MAP2K4,CAMK2G,CAMK2D,ADCY2,ITPR1,DNM1,PTK2,RAC1,PAK3
Calcium Transport I	2,47	0,33	ATP2B2,ATP2B4,ATP2B1
Trans, trans-farnesyl Diphosphate Biosynthesis	2,42	0,67	IDI1,FDPS
Protein Kinase A Signaling	2,36	0,07	FLNB,PHKA2,YWHAQ,ADD1,AKAP2,CAMK2D,MYH10,ADD2,PTK2,PPP3CC,BAD,GNAS,PTPRD,CAMK2G,MYLK,PTPRU,ADCY2,ITPR1,PTPRC,AKAP11
Paxillin Signaling	2,31	0,10	FGFR2,ARHGEF7,ITGAV,VCL,MAP2K4,ITGA10,PTK2,RAC1,PAK3
Cdc42 Signaling	2,25	0,10	DIAPH1,ACTR2,EXOC1,MAP2K4,MYLK,RASA1,TNK2,APC,PAK3
GABA Receptor Signaling	2,20	0,12	GNAS,ADCY2,UBQLN1,DNM1,AP2M1,GPHN,AP1B1
iCOS-iCOSL Signaling in T Helper Cells	2,13	0,09	FGFR2,CAMK2G,CAMK2D,ITPR1,PTPRC,RAC1,PLEKHA4,PPP3CC,BAD
Epithelial Adherens Junction Signaling	2,02	0,09	MYH14,ACTR2,AFDN,VCL,SORBS1,MYH11,MYH10,RAPGEF1,RAC1,APC
GDNF Family Ligand-Receptor Interactions	2,01	0,11	FGFR2,MAP2K4,PDLIM7,GFRA1,ITPR1,RASA1,RAC1
Virus Entry via Endocytic Pathways	1,99	0,10	FLNB,FGFR2,CLTA,CLTB,DNM1,AP2M1,RAC1,AP1B1
Tight Junction Signaling	1,98	0,08	MYH14,EPB41,AFDN,VCL,SNAP25,MYLK,MYH11,NAPB,MYH10,RAC1,SPTAN1
Rac Signaling	1,83	0,08	FGFR2,ACTR2,MAP2K4,ABI2,CYFIP2,PTK2,RAC1,PAK3,NCKAP1
Agrin Interactions at Neuromuscular Junction	1,71	0,10	ARHGEF7,CTTN,MAP2K4,PTK2,RAC1,PAK3
Cellular Effects of Sildenafil (Viagra)	1,66	0,08	MYH14,GNAS,MYLK,MYH11,ADCY2,ITPR1,MYH10,CACNA1D
Reelin Signaling in Neurons	1,59	0,09	FGFR2,ARHGEF11,HCK,MAP2K4,APP,APOE,APBB1
Glycolysis I	1,59	0,17	ENO4,PKM,PFKM
Gluconeogenesis I	1,59	0,17	ME3,ENO4,ME2
Leucine Degradation I	1,50	0,25	BCAT1,HMGCLL1
FAK Signaling	1,49	0,08	FGFR2,ARHGEF7,VCL,TNS1,PTK2,RAC1,PAK3
PAK Signaling	1,46	0,08	FGFR2,ARHGEF7,MAP2K4,MYLK,PTK2,RAC1,PAK3
Lipoate Biosynthesis and Incorporation II	1,44	1,00	LIAS
Acetyl-CoA Biosynthesis III (from Citrate)	1,44	1,00	ACLY
Netrin Signaling	1,43	0,11	UNC5C,ABLIM1,RAC1,PPP3CC
Pregnenolone Biosynthesis	1,40	0,22	CYP2E1,MICAL2
CTLA4 Signaling in Cytotoxic T Lymphocytes	1,36	0,08	FGFR2,CLTA,AP1S2,CLTB,AP2M1,AP1B1
Huntington's Disease Signaling	1,33	0,06	FGFR2,NCOR2,SNAP25,SDHA,DNM1,DCTN1,DNM1L,CLTA,MAP2K4,NAPB,ITPR1,CLTB,RASA1
Remodeling of Epithelial Adherens Junctions	1,31	0,09	DNM1L,ACTR2,VCL,DNM1,APC
Dermatan Sulfate Degradation (Metazoa)	1,31	0,20	CEMIP,IDS
Histidine Degradation VI	1,31	0,20	CYP2E1,MICAL2

Supplementary Table 10

Legend:

**Term**	GO term - Biological Process
**P-value**	P value computed using a standard statistical method: Fisher's exact test or the hypergeometric test.
**Adjusted P-v**	Adjusted p-value using the Benjamini-Hochberg method for correction for multiple hypotheses testing
**Z-score**	Score computed using a modification to Fisher's exact test for deviation from an expected rank.
**Combined Sc**	Is a combination of the p-value and z-score calculated by multiplying the two scores as follows: c = log(p) * z, where p is the p value and z the z-score
**Genes**	List of genes related to the GO term

Term	P-value	Adjusted P-v	Z-score	Combined Sc	Genes
axon guidanc	1,4014E-10	1,3967E-07	-2,4103009	38,0441621	MEG3;MYCBP2;CLTA;CACNA1D;ABLIM1;ALCAM;CHL1;MYH14;ITGAV;MYH11;NRCAM;NCAM1;RAC1;MYH10;SPTAN1;AP2M1;CLASP1;CLASP2;ARHGEF11;SEMA4D;SEMA4F;UNC5C;ANK2;GFRA1;ANK3;PTK2;CACNB1;MYL6;NFASC;TYROBP;PTPRC;KIAA1598;ITGA10;FEZ1;FEZ2
neuron proje	1,4014E-10	1,3967E-07	-2,4078531	38,0055251	MEG3;MYCBP2;CLTA;CACNA1D;ABLIM1;ALCAM;CHL1;MYH14;ITGAV;MYH11;NRCAM;NCAM1;RAC1;MYH10;SPTAN1;CLASP1;AP2M1;CLASP2;ARHGEF11;SEMA4D;SEMA4F;UNC5C;ANK2;GFRA1;ANK3;PTK2;CACNB1;MYL6;NFASC;TYROBP;PTPRC;KIAA1598;ITGA10;FEZ1;FEZ2
cell part mor	2,2016E-10	1,6457E-07	-2,3977166	37,4521422	APP;DOCK7;MYCBP2;MFF;COL4A3BP;SPAST;MAP2;KIF3A;EPB41L3;PACSIN2;MYH14;NRCAM;APBB1;RAC1;PAK3;DNM1L;FIS1;GSN;DST;MINK1;MYO9B;ANK3;DCLK1;PTK2;APC;PARD3;FGFR2;PICALM
actin filamen	6,4658E-11	1,3967E-07	-2,3306742	36,7873348	NCKAP1;CAMK2D;EPB41;LDB3;ADD1;ADD2;SIPA1L1;EPB41L1;EPB41L2;EPB41L3;PACSIN2;MYH14;DMD;FLNB;MYH11;RAC1;MYH10;PDLIM7;ARHGEF11;GSN;TPM3;MINK1;MYO5A;MYO9B;SSH2;MYL6;FMNL1;FMNL2;INF2;PARD3;CALR;LPIN1
microtubule-	4,1584E-09	2,0723E-06	-2,4223303	31,7007289	DYNC1I2;APP;RAB1A;TNKS;DCTN1;DOCK7;DNAH9;KLC1;TTL;CAMSAP2;UCHL1;SPAST;OPA1;MAP2;KIF3A;NTMT1;ZNF207;KIF21A;KIF1B;KIF1A;MAP4;CLASP1;CLASP2;SS18;ACTR2;DST;NEK7;PTK2;KTN1;EML4;KIF9;APC;KIF2A;PARD3;CCSER2;TACC1
cellular comp	4,1584E-09	2,0723E-06	-2,4160471	31,6185016	APP;RAB1A;DOCK7;MYCBP2;MFF;ADD1;RTN4;COL4A3BP;SPAST;MAP2;KIF3A;EPB41L3;PACSIN2;MYH14;ITGAV;NRCAM;APBB1;RAC1;DNM1L;PAK3;SS18;FIS1;GSN;DST;MINK1;FN1;MYO9B;ANK3;THOC5;PTK2;DCLK1;APC;ADAM15;PARD3;FGFR2;PICALM
regulation of	4,0562E-08	1,5977E-05	-2,3246515	25,6742312	SNAP25;PRKCSH;TTL;RTN4;PQBP1;ARHGAP4;DGUOK;SIPA1L1;SPP1;DMD;MBP;NRCAM;APOE;IL15RA;SEMA4D;FN1;COBL;SEMA4F;SSH2;PTK2;PTPRD;FEZ1;CAPRIN2;RAPGEF1;NSMF;DBN1
cortical actin	4,0701E-07	8,1131E-05	-2,717712	25,5993321	FMNL1;FMNL2;EPB41L1;EPB41;EPB41L2;EPB41L3;CALR
cell projectio	7,5682E-08	2,5143E-05	-2,3619784	25,0155312	APP;GSN;DST;DOCK7;MINK1;MYCBP2;MYO9B;ANK3;DCLK1;PTK2;SPAST;APC;MAP2;KIF3A;PARD3;EPB41L3;PACSIN2;NRCAM;APBB1;RAC1;PAK3;FGFR2;PICALM
axonogenesis	4,2749E-08	1,5977E-05	-2,2498921	24,8485631	APP;DST;MYCBP2;DOCK7;ANK3;PTK2;DCLK1;SPAST;APC;MAP2;PARD3;NRCAM;APBB1;PAK3;FGFR2;PICALM
regulation of	9,6255E-08	2,878E-05	-2,3694429	24,7744747	TTL;PALM2-AKAP2;RTN4;PQBP1;ARHGAP4;SIPA1L1;EPB41L3;SPP1;S100A13;MYH14;ANXA7;MBP;NRCAM;APOE;RAC1;MYH10;SEMA4D;FN1;SEMA4F;SSH2;PTK2;PTPRD;DIAPH1;HCK;FMNL1;FMNL2;RASA1;CAPRIN2;NSMF;CALR;ARHGEF7;BRWD3
regulation of	1,2027E-07	3,1582E-05	-2,3836116	24,701207	SNAP25;NCKAP1;PRKCSH;TTL;RTN4;PQBP1;ARHGAP4;DGUOK;SIPA1L1;SPP1;DMD;MBP;NRCAM;APOE;RAC1;IL15RA;SEMA4D;PIFO;FN1;COBL;SEMA4F;SSH2;PTK2;PTPRD;APC;FEZ1;CAPRIN2;RAPGEF1;NSMF;ARHGEF7;DBN1
cortical cytos	8,9574E-07	0,00014879	-2,7449009	24,1907047	FMNL1;FMNL2;EPB41L1;EPB41;EPB41L2;EPB41L3;CALR
actin cytoske	1,2675E-07	3,1582E-05	-2,3009937	23,8450431	ARHGEF11;NCKAP1;EPB41;MINK1;LDB3;SSH2;ADD1;ADD2;FMNL1;FMNL2;SIPA1L1;INF2;EPB41L1;EPB41L2;EPB41L3;PACSIN2;MYH14;FLNB;MYH11;CALR;RAC1;LPIN1;MYH10;PDLIM7
nucleoside m	2,6996E-07	6,2091E-05	-2,3632963	22,8930192	MACF1;AK2;DNAH9;IDE;ATP5O;UQCRH;DGUOK;PSMD6;RNF213;SPAST;GUK1;KIF3A;HLTF;MYH14;KIF21A;TK2;KIF1B;KIF1A;MYH10;SLC25A25;PMS1;ABCA1;BAD;ATP2B2;MYO9B;ABCA8;ACLY;MYL6;KIF9;KIF2A;DPYD;ACIN1
neuron proje	3,2952E-07	7,0377E-05	-2,2472831	21,4877249	APP;DST;MYCBP2;DOCK7;MINK1;ANK3;DCLK1;PTK2;SPAST;APC;MAP2;PARD3;EPB41L3;NRCAM;APBB1;RAC1;PAK3;FGFR2;PICALM
ATP metaboli	5,7694E-07	0,00010782	-2,3504642	21,4717053	MACF1;AK2;DNAH9;IDE;ATP5O;UQCRH;PSMD6;RNF213;SPAST;GUK1;KIF3A;HLTF;MYH14;KIF21A;KIF1B;KIF1A;MYH10;SLC25A25;PMS1;ABCA1;BAD;MYO9B;ATP2B2;ABCA8;ACLY;KIF9;MYL6;KIF2A;ACIN1
regulation of	8,156E-07	0,00014345	-2,3501515	20,7977422	SNAP25;APP;PRKCSH;TTL;RTN4;PQBP1;ARHGAP4;DGUOK;SIPA1L1;SPP1;MBP;DMD;NRCAM;APOE;IL15RA;SEMA4D;TCF12;FN1;COBL;SEMA4F;SSH2;PTK2;PTPRD;BIN1;FEZ1;CAPRIN2;RAPGEF1;NSMF;CALR;DBN1
ribonucleosic	1,4203E-06	0,00022351	-2,3520748	19,7717144	MACF1;AK2;DNAH9;IDE;ATP5O;UQCRH;PSMD6;RNF213;SPAST;GUK1;KIF3A;HLTF;MYH14;KIF21A;KIF1B;KIF1A;MYH10;SLC25A25;PMS1;ABCA1;BAD;MYO9B;ATP2B2;ABCA8;ACLY;MYL6;KIF9;KIF2A;DPYD;ACIN1
purine ribonu	2,1133E-06	0,00030174	-2,3534928	19,0773135	MACF1;AK2;DNAH9;IDE;ATP5O;UQCRH;PSMD6;RNF213;SPAST;GUK1;KIF3A;HLTF;MYH14;KIF21A;KIF1B;KIF1A;MYH10;SLC25A25;PMS1;ABCA1;BAD;MYO9B;ATP2B2;ABCA8;ACLY;MYL6;KIF9;KIF2A;ACIN1
purine nucle	2,2201E-06	0,00030174	-2,3525243	19,0694634	MACF1;AK2;DNAH9;IDE;ATP5O;UQCRH;PSMD6;RNF213;SPAST;GUK1;KIF3A;HLTF;MYH14;KIF21A;KIF1B;KIF1A;MYH10;SLC25A25;PMS1;ABCA1;BAD;MYO9B;ATP2B2;ABCA8;ACLY;MYL6;KIF9;KIF2A;ACIN1
regulation of	2,1882E-06	0,00030174	-2,1578271	17,4912559	SEMA4D;FN1;PALM2-AKAP2;PTK2;DIAPH1;HCK;FMNL1;RASA1;EPB41L3;S100A13;MYH14;ANXA7;MYH10;BRWD3
microtubule-	3,8454E-06	0,0004999	-2,2476963	17,0849572	APP;ACTR2;RAB1A;DYNC1I2;DST;DCTN1;DNAH9;KLC1;KTN1;KIF9;UCHL1;KIF2A;OPA1;KIF3A;KIF21A;KIF1B;KIF1A;MAP4
negative regu	1,8714E-05	0,00159869	-2,420172	15,5824478	PRKCSH;INSIG1;ADD1;RTN4;ADD2;ARHGAP4;TRIM9;DGUOK;OPA1;PACSIN2;SPP1;ZNF207;PPFIA1;MBP;APOE;RAC1;SPTAN1;AP2M1;CLASP1;CLASP2;IL15RA;GSN;SEMA4F;PTK2;EML4;TOM1L2;APC;TRIP12;FGFR2;PICALM
microtubule (	1,3779E-05	0,00148184	-2,3303289	15,1808586	SS18;DST;TNKS;NEK7;DOCK7;TTL;PTK2;CAMSAP2;SPAST;KIF2A;APC;MAP2;PARD3;NTMT1;ZNF207;CCSER2;TACC1;MAP4;CLASP1;CLASP2
ATP catabolic	1,1951E-05	0,00142937	-2,2892577	14,9958259	ABCA1;MACF1;ATP2B2;DNAH9;MYO9B;IDE;ABCA8;ATP5O;ACLY;MYL6;PSMD6;KIF9;RNF213;SPAST;KIF2A;KIF3A;HLTF;ACIN1;MYH14;KIF21A;KIF1B;KIF1A;MYH10;PMS1
regulation of	1,646E-05	0,00149142	-2,2965812	14,9462113	SEMA4D;FN1;SEMA4F;SSH2;TTL;PTK2;RTN4;PQBP1;ARHGAP4;PTPRD;SIPA1L1;CAPRIN2;SPP1;NRCAM;NSMF;MBP;RAC1;APOE;CALR;ARHGEF7
ribonucleosic	1,5364E-05	0,00148184	-2,2741731	14,8150336	ABCA1;MACF1;ATP2B2;DNAH9;MYO9B;ABCA8;IDE;ATP5O;ACLY;MYL6;KIF9;PSMD6;RNF213;SPAST;KIF2A;KIF3A;HLTF;MYH14;ACIN1;KIF21A;KIF1B;KIF1A;MYH10;PMS1
purine nucle	1,6144E-05	0,00149142	-2,27466	14,8035472	ABCA1;MACF1;ATP2B2;DNAH9;MYO9B;ABCA8;IDE;ATP5O;ACLY;MYL6;KIF9;PSMD6;RNF213;SPAST;KIF2A;KIF3A;HLTF;MYH14;ACIN1;KIF21A;KIF1B;KIF1A;MYH10;PMS1
purine ribonu	1,5364E-05	0,00148184	-2,2717222	14,7990675	ABCA1;MACF1;ATP2B2;DNAH9;MYO9B;ABCA8;IDE;ATP5O;ACLY;MYL6;KIF9;PSMD6;RNF213;SPAST;KIF2A;KIF3A;HLTF;MYH14;ACIN1;KIF21A;KIF1B;KIF1A;MYH10;PMS1
nucleoside m	1,7812E-05	0,00156643	-2,2847469	14,7570819	ABCA1;MACF1;MYO9B;ATP2B2;DNAH9;ABCA8;IDE;ATP5O;ACLY;MYL6;KIF9;PSMD6;RNF213;SPAST;KIF2A;KIF3A;HLTF;MYH14;ACIN1;KIF21A;KIF1B;KIF1A;MYH10;PMS1
regulation of	1,2774E-05	0,00146903	-2,216253	14,4569567	EML4;GSN;SPAST;ASPH;APC;SPTAN1;ADD1;CLASP1;ADD2;CLASP2
establishmen	1,5053E-05	0,00148184	-2,1937511	14,2911267	SNAP25;ACTR2;RAB1A;DCTN1;DENND1A;MYO5A;PTK2;TRIM9;RIMS1;KIF3A;FEZ1;KIF1B;MAP4;MYH10;FGFR2;CLASP1
regulation of	3,6204E-05	0,00292571	-2,410236	14,0618465	GEN1;NCKAP1;ACTR2;GSN;DST;MYCBP2;ARHGEF10L;SSH2;PTK2;ADD1;ADD2;EML4;HCK;SPAST;APC;ABI2;RASA1;PPFIA1;RAC1;PAK3;PAM;SPTAN1;CLASP1;CLASP2
organelle loc	1,164E-05	0,00142937	-2,1265879	13,9302543	RAB1A;ACTR2;DCTN1;DOCK7;MYO5A;PTK2;KIF3A;MYO1A;PARD3;S100A13;TACC1;DMD;KIF1B;MYH10
regulation of	1,3911E-05	0,00148184	-2,1321945	13,890118	SEMA4D;FN1;SEMA4F;SSH2;TTL;PTK2;RTN4;ARHGAP4;SIPA1L1;SPP1;NRCAM;MBP;APOE
plasma mem	2,6491E-05	0,00220024	-2,1239205	12,9966691	MACF1;SGIP1;AHNAK2;MYO5A;ATP1B3;ANK2;ANK3;GOLGA4;SCP2;EPB41L3;PACSIN2;RAC1;MYH10
single-organi	4,3598E-05	0,00325893	-2,2296231	12,7676148	MACF1;RAB1A;RPN2;DCTN1;RPLP0;MFF;GPHN;GOLGA4;SCP2;KIF3A;EPB41L3;S100A13;KIF1B;KPNA5;APOE;RAC1;FIS1;ACTR2;RPS9;MYO5A;ANK2;ATP1B3;ANK3;CACNB1;RPS25;TMCO6;PEX5L;PARD3;RPL27;RPS24
regulation of	4,7613E-05	0,00347229	-2,2204329	12,5741813	SGIP1;TNK2;ADD1;RUFY1;HCK;KIF3A;DNAJC6;BIN1;PPT1;PACSIN2;AAK1;ITGAV;RAC1;CALR;PICALM
actin filamen	3,8094E-05	0,00299737	-2,1120742	12,2711942	MYL6;CAMK2D;TPM3;PARD3;MYH14;MYO5A;MYO9B;DMD;MYH10
mitochondric	0,00012495	0,00762448	-2,4654937	12,0227121	FIS1;COL4A3BP;MYH14;MFF;DNM1L
regulation of	6,1809E-05	0,00440024	-2,191528	11,8914401	EML4;GSN;SPAST;ASPH;APC;SPTAN1;ADD1;CLASP1;ADD2;CLASP2
regulation of	9,5569E-05	0,00607981	-2,282806	11,6486611	ARHGEF11;ITSN2;DENND1C;PLEKHG4;SEMA4D;DOCK9;RALGAPA2;DENND1A;DOCK7;ARHGEF10L;MYO9B;PTK2;ARHGAP4;DOCK10;SIPA1L1;RASA1;RAPGEF1;GNAS;PKP4;DENND5A;ARHGEF7;DGKI;AGFG1
endocytosis (	8,3655E-05	0,00568476	-2,2491791	11,6281805	ITSN2;APP;RAB1A;SGIP1;TNK2;SYNRG;DENND1A;CLTA;DNM1;RUFY1;HCK;BIN1;PPT1;ENPP2;PACSIN2;AAK1;ITGAV;APOE;DNM1L;PDLIM7;AP2M1;PICALM
negative regu	7,5627E-05	0,00525869	-2,1873154	11,4787524	EML4;GSN;APC;SPTAN1;ADD1;CLASP1;ADD2;CLASP2
regulation of	4,35E-05	0,00325893	-1,9896076	11,3932007	SEMA4D;FN1;SPP1;NRCAM;SEMA4F;APOE;TTL;RTN4;ARHGAP4
cellular comp	9,9744E-05	0,00621325	-2,2394381	11,3787461	NAPB;RPS9;SMARCC2;GSN;RPLP0;FN1;MICAL2;MTIF2;KLC1;ADD1;PTK2;RPS25;KIF9;KIF2A;APC;ADAM15;DNAJC6;SPP1;ACIN1;RPL27;DNM1L;SPTAN1;RPS24
mitochondria	0,00016288	0,0093658	-2,4141017	11,2755216	FIS1;OPA1;MFF;LPIN1;DNM1L
muscle contr	9,252E-05	0,00601382	-2,1750133	11,1223539	ARHGEF11;DTNA;TPM3;ANK2;SORBS1;MYLK;MYL6;COL4A3BP;ASPH;CALD1;SLMAP;KCNMA1;MYH14;DMD;MYH11;VCL
establishmen	8,8168E-05	0,00585828	-2,1207924	10,9006598	ARHGEF11;NCKAP1;ACTR2;DST;PARD3;DOCK7;MYO9B;PTK2;CLASP1;SDCCAG8;CLASP2
regulation of	0,00022679	0,01189652	-2,3912953	10,5970486	FIS1;INF2;MFF;DNM1L
negative regu	0,00012977	0,00776013	-2,1661368	10,524731	EML4;GSN;APC;SPTAN1;ADD1;CLASP1;ADD2;CLASP2
positive regu	0,00016126	0,0093658	-2,2503604	10,510737	ARHGEF11;ITSN2;DENND1C;PLEKHG4;SEMA4D;DOCK9;RALGAPA2;DENND1A;DOCK7;ARHGEF10L;MYO9B;ARHGAP4;DOCK10;SIPA1L1;RASA1;RAPGEF1;GNAS;PKP4;DENND5A;ARHGEF7
nucleus local	0,00020902	0,01157347	-2,2032315	9,82429652	DOCK7;DMD;TACC1;MYH10;PTK2
generation o	0,00027312	0,01296232	-2,2207193	9,65059951	SNAP25;SLC25A3;FASTKD1;NDUFB5;AK2;ITPR1;CACNA1D;ADCY2;ATP5O;SDHA;PHKA2;ENO4;UQCRH;ACLY;PKM;OGDH;GNAS;NDUFS2;ME3;NDUFV3;GYG1;PFKM;SLC25A25
actin filamen	0,00024156	0,01204307	-2,1649679	9,56756839	ACTR2;GSN;MICAL2;COBL;SORBS2;SORBS1;ADD1;ADD2;MYLK;DIAPH1;ABI2;RAC1;DBN1
protein locali	0,00021881	0,01181331	-2,153617	9,5588892	MACF1;RPS9;RPN2;RPLP0;MYO5A;ATP1B3;ANK2;ANK3;GPHN;CACNB1;RPS25;GOLGA4;SCP2;PARD3;EPB41L3;RPL27;APOE;RAC1;RPS24
negative regu	0,00018474	0,01042203	-2,0650931	9,42474054	DGUOK;IL15RA;PRKCSH;SPP1;SEMA4F;MBP;APOE;PTK2;RTN4;ARHGAP4
regulation of	0,00040248	0,01671425	-2,3031952	9,42350831	SH3GLB1;NAPB;ACTR2;GSN;INSIG1;IDE;ADD1;ADD2;HCK;TRIM9;KIF9;APC;RASA1;RAC1;PAK3;DNM1L;SPTAN1
antigen proc	0,00025572	0,01233225	-2,1412412	9,41190547	DYNC1I2;DCTN1;AP1B1;CLTA;KLC1;SEC61A2;PSMD6;KIF2A;KIF3A;AP1S2;ITGAV;CALR;AP2M1;SEC31A
regulation of	0,00038792	0,01633625	-2,2806963	9,38362514	MACF1;GSN;SEMA4D;MINK1;ADAM22;TNC;ANK3;PTK2;EGFLAM;CYTH4;PTPRC;ADAM15;APC;RASA1;SPP1;DMD;ITGAV;PKP4;CALR;RAC1;ARHGEF7
muscle syste	0,00028714	0,0130601	-2,1626795	9,38212241	ARHGEF11;CAMK2D;DTNA;TPM3;ANK2;SORBS1;MYLK;MYL6;COL4A3BP;ASPH;CALD1;SLMAP;KCNMA1;MYH14;DMD;MYH11;VCL
positive regu	0,00035728	0,01570973	-2,2411954	9,30874959	DENND1C;ITSN2;DOCK9;DENND1A;DOCK7;ARHGEF10L;SIPA1L2;ARHGAP12;ARHGAP4;DOCK10;SIPA1L1;CYTH4;DENND5A;AGFG1;RGS7;ARHGEF11;PLEKHG4;SEMA4D;RALGAPA2;MYO9B;BIN1;RASA1;RAPGEF1;GNAS;PKP4;ARHGEF7;RAPGEF6
antigen proc	0,00028828	0,0130601	-2,1359428	9,26613358	DYNC1I2;DCTN1;AP1B1;CLTA;KLC1;SEC61A2;PSMD6;KIF2A;KIF3A;AP1S2;ITGAV;CALR;AP2M1;SEC31A
positive regu	0,00036899	0,01598969	-2,2404255	9,26597649	ITSN2;DENND1C;DOCK9;DENND1A;DOCK7;ARHGEF10L;SIPA1L2;ARHGAP12;ARHGAP4;DOCK10;SIPA1L1;CYTH4;DENND5A;AGFG1;RGS7;ARHGEF11;PLEKHG4;SEMA4D;RALGAPA2;MYO9B;BIN1;RASA1;RAPGEF1;GNAS;PKP4;ARHGEF7;RAPGEF6
antigen proc	0,00022125	0,01181331	-2,0814554	9,23859799	DYNC1I2;KIF2A;KIF3A;DCTN1;AP1S2;AP1B1;CLTA;KLC1;AP2M1;SEC31A
antigen proc	0,00024167	0,01204307	-2,0612584	9,10924859	DYNC1I2;KIF2A;KIF3A;DCTN1;AP1S2;AP1B1;CLTA;KLC1;AP2M1;SEC31A
regulation of	0,00052367	0,02007411	-2,3092815	9,02542131	CAMK2D;WNK2;ATP2B4;DMD;ATP1B3;ANK3
antigen proc	0,00028725	0,0130601	-2,0756416	9,00453517	DYNC1I2;KIF2A;DCTN1;KIF3A;AP1S2;AP1B1;CLTA;KLC1;AP2M1;SEC31A
cell division (	0,00024884	0,01219726	-2,0378987	8,98008973	ACTR2;CCND3;SENP5;MAEA;PARD3;TACC1;MAP4;THOC5;SEPT4;CDK14;FGFR2;CLASP1
protein locali	0,00024106	0,01204307	-2,0226684	8,9387091	MACF1;GOLGA4;SCP2;EPB41L3;MYO5A;ATP1B3;ANK2;ANK3;RAC1
lamellipodiur	0,00038689	0,01633625	-2,1172485	8,71114073	NCKAP1;ABLIM1;MYO9B;RAC1;ARHGEF7;VCL
regulation of	0,00049876	0,01962216	-2,19712	8,63708871	EML4;SPAST;APC;CLASP1;CLASP2
regulation of	0,00033426	0,01491706	-2,0110149	8,45682052	KIF3A;DNAJC6;SGIP1;TNK2;PPT1;AAK1;RAC1;PICALM
cell junction	0,00045234	0,01803326	-2,0725858	8,32254611	DST;FN1;ANK2;SORBS1;MLLT4;NFASC;APC;PARD3;EPB41L3;PKP4;ARHGEF7;VCL;TNS1
regulation of	0,0007249	0,02435323	-2,199039	8,16962975	NCKAP1;ACTR2;GSN;ANK2;ARHGEF10L;SSH2;ADD1;ADD2;HCK;ABI2;PARD3;RASA1;PPFIA1;RAC1;PAK3;PAM;SPTAN1
antigen proc	0,00056824	0,0212381	-2,0991113	8,08568964	DYNC1I2;DCTN1;AP1B1;CLTA;KLC1;SEC61A2;PSMD6;KIF2A;KIF3A;AP1S2;ITGAV;CALR;AP2M1;SEC31A
cytoskeleton	0,00072457	0,02435323	-2,1655137	8,04508016	ACTR2;APC;RASA1;ANK3;MYH10
negative regu	0,00082795	0,02633577	-2,195008	7,9828644	GSN;INSIG1;ADD1;ADD2;EML4;TRIM9;TOM1L2;OPA1;APC;ZNF207;PPFIA1;TRIP12;SPTAN1;FGFR2;CLASP1;CLASP2
regulation of	0,00078202	0,025695	-2,1796173	7,98057962	NAPB;C2CD5;SGIP1;INSIG1;TNK2;ADD1;RUFY1;HCK;TRIM9;DNAJC6;BIN1;KIF3A;PPT1;PACSIN2;AAK1;ITGAV;CALR;RAC1;PICALM
axon cargo tr	0,00069536	0,02435323	-2,1369162	7,93883797	APP;UCHL1;OPA1;DST;KIF1B;KIF1A
regulation of	0,00069399	0,02435323	-2,1359095	7,93509801	ARHGEF11;ABCA1;RALGAPA2;DENND1A;MYO9B;SIPA1L2;ARHGAP12;ARHGAP4;SIPA1L1;CYTH4;RASA1;RAPGEF1;APOE;RAC1;ARHGEF7;RAPGEF6;DGKI
macromolecu	0,00052135	0,02007411	-2,0233132	7,90776432	NAPB;RPS25;RPS9;SMARCC2;KIF2A;DNAJC6;RPLP0;MICAL2;MTIF2;RPL27;KLC1;RPS24
mitochondria	0,00043913	0,0177645	-1,9587762	7,89495129	FIS1;CHCHD3;OPA1;MFF
cell junction	0,00070229	0,02435323	-2,0715536	7,69600974	DST;FN1;ANK2;SORBS1;MLLT4;NFASC;APC;PARD3;EPB41L3;PKP4;RAC1;ARHGEF7;VCL;TNS1
regulation of	0,00043966	0,0177645	-1,9019025	7,66571889	FN1;NRCAM;SEMA4F;APOE;TTL;RTN4;ARHGAP4
negative regu	0,00071063	0,02435323	-2,0626015	7,66275193	APP;IL15RA;DGUOK;PRKCSH;SPP1;SEMA4F;MBP;APOE;CALR;PTK2;RTN4;ARHGAP4
negative regu	0,00063696	0,02322576	-2,0364442	7,66210727	IL15RA;DGUOK;PRKCSH;SPP1;SEMA4F;MBP;APOE;PTK2;RTN4;ARHGAP4
positive regu	0,00097839	0,02954935	-2,1424397	7,54501569	FIS1;C2CD5;ANK3;MFF
actin polyme	0,00079606	0,02575142	-2,0184093	7,3858952	DIAPH1;GSN;ABI2;MICAL2;COBL;RAC1
regulation of	0,00071227	0,02435323	-1,9734822	7,33166563	PTPRD;SIPA1L1;SEMA4D;CAPRIN2;COBL;NSMF;APOE;DBN1;PQBP1
cellular comp	0,00055857	0,02114082	-1,8899541	7,28870174	GSN;APC;ACIN1;DNM1L;SPTAN1;PTK2;ADD1
positive regu	0,00080096	0,02575142	-1,9831302	7,25679989	ARHGEF11;ITSN2;DOCK10;PLEKHG4;SEMA4D;DOCK9;DOCK7;PKP4;ARHGEF10L;MYO9B;ARHGEF7;ARHGAP4
maintenance	0,00095431	0,02954935	-2,0038508	7,05694821	BSCL2;PEX5L;EPB41L3;ZNF207;G3BP2;FLNB;APOE;CALR;ANK3;DBN1;CLASP1;CLASP2
positive regu	0,00058597	0,0216302	-1,8276	7,00640584	WNK2;DMD;ATP1B3;ANK3
regulation of	0,0010191	0,02987356	-1,9926499	6,99575856	HCK;AP1S2;AP1B1;RAC1;AP2M1
regulation of	0,00099472	0,029742	-1,9852195	6,97843405	ARHGEF11;ITSN2;PLEKHG4;SEMA4D;DOCK9;DOCK7;ARHGEF10L;MYO9B;PTK2;ARHGAP4;DOCK10;PKP4;ARHGEF7
synapse orga	0,0009135	0,02875121	-1,9640889	6,97069927	APP;NFASC;TNC;MYO5A;NRCAM;ATP2B2;ANK3;PAK3;FGFR2;GPHN
regulation of	0,00132263	0,03661739	-2,0729126	6,85560296	SH3GLB1;FIS1;INF2;PPP3CC;OPA1;YWHAQ;BAD;MFF;DNM1L
regulation of	0,00105153	0,03052502	-1,9600041	6,83886341	PGS1;SEMA4D;FN1;SPP1;NRCAM;SEMA4F;APOE;TTL;FGFR2;RTN4;ARHGAP4
establishmen	0,00114064	0,03217465	-1,98474	6,82071064	RIMS1;RAB1A;SNAP25;TRIM9;DCTN1;KIF3A;DENND1A;MYO5A;FGFR2
microtubule-	0,00114064	0,03217465	-1,9770706	6,79435416	APP;RAB1A;UCHL1;OPA1;DST;KIF3A;DCTN1;KIF1B;KIF1A
regulation of	0,001339	0,03673034	-2,0275813	6,69943712	CAMK2D;ASPH;WNK2;MINK1;MYO5A;CACNA1D;ATP1B3;DMD;RIPK1;ANK2;ANK3
positive regu	0,00131552	0,03661739	-2,0156475	6,66621402	ASPH;WNK2;ATP1B3;DMD;ANK2;ANK3
regulation of	0,00168804	0,04277317	-2,018124	6,36081267	CAMK2D;ASPH;WNK2;MINK1;MYO5A;CACNA1D;ATP1B3;DMD;RIPK1;ANK2;ANK3
regulation of	0,00192914	0,04614509	-2,0667065	6,35711629	HCK;ACTR2;GSN;INF2;RASA1;ATP2B2;RAC1;SSH2;PAK3;SPTAN1;ADD1;ADD2
myelination (	0,00107441	0,0308893	-1,8152296	6,31218037	NFASC;PARD3;ADAM22;MYO5A;MBP;SCN2A;ASPA
endoplasmic	0,00097839	0,02954935	-1,7883695	6,29808917	FIS1;APP;CAMK2D;ITPR1
cell volume h	0,00076455	0,02540001	-1,6993635	6,24177178	SLC12A2;KCNMA1;ANXA7;ADD1
positive regu	0,00174108	0,04338199	-1,984011	6,22525291	SH3GLB1;FIS1;PPP3CC;YWHAQ;BAD;MFF;DNM1L
negative regu	0,00175672	0,04340974	-1,9833889	6,22203265	EML4;GSN;APC;PPFIA1;SPTAN1;ADD1;CLASP1;ADD2;CLASP2
regulation of	0,00097839	0,02954935	-1,7601499	6,19870824	CAMK2D;ASPH;DMD;ANK2
positive regu	0,00224292	0,0489514	-2,0292524	6,12210673	SH3GLB1;FIS1;PTPRC;PPP3CC;YWHAQ;FBXW7;BAD;RIPK1;SEPT4;MFF;TPD52L1;DNM1L
regulation of	0,00187431	0,04519512	-1,9707406	6,1029226	HCK;ACTR2;GSN;RASA1;RAC1;SSH2;PAK3;SPTAN1;ADD1;ADD2
regulation of	0,00199172	0,04726387	-1,9659766	6,00017861	HCK;ACTR2;GSN;RASA1;RAC1;SSH2;PAK3;SPTAN1;ADD1;ADD2
regulation of	0,00215168	0,04765578	-1,9586179	5,96154587	HCK;ACTR2;GSN;RASA1;RAC1;PAK3;SPTAN1;ADD1;ADD2
cytokinesis ((	0,00138082	0,03753311	-1,8136459	5,95335013	ARHGEF11;ACTR2;APC;RASA1;NEK7;ANK3;MYH10;RAB11FIP3
cell projectio	0,00219595	0,04827854	-1,9637846	5,9517755	NCKAP1;ACTR2;RAB1A;MYLK;TTBK2;TSGA10;ABLIM1;MYO1A;KIF3A;DMD;RAC1;ARHGEF7;MYH10;VCL
axon ensheat	0,00144413	0,0385416	-1,8028126	5,86998869	NFASC;PARD3;ADAM22;MYO5A;MBP;SCN2A;ASPA
regulation of	0,00211499	0,04765578	-1,927849	5,86789273	EML4;GEN1;DIAPH1;MACF1;SPAST;APC;DST;TACC1;CLASP1;CLASP2
ensheathmer	0,00144413	0,0385416	-1,7997415	5,85998919	NFASC;PARD3;ADAM22;MYO5A;MBP;SCN2A;ASPA
cytoskeleton	0,00215168	0,04765578	-1,8886406	5,74855229	APP;RAB1A;UCHL1;OPA1;DST;KIF3A;DCTN1;KIF1B;KIF1A
lamellipodiur	0,00186231	0,04519512	-1,8502462	5,72977967	NCKAP1;ABLIM1;RAC1;ARHGEF7;VCL
maintenance	0,00201288	0,04738989	-1,8782442	5,72741698	EPB41L3;ZNF207;G3BP2;FLNB;APOE;ANK3;DBN1;CLASP1;CLASP2
dendrite mor	0,00213517	0,04765578	-1,867086	5,68294536	MAP2;MINK1;RAC1;DCLK1;PICALM
negative regu	0,00185035	0,04519512	-1,8237021	5,64757888	SPP1;SEMA4F;MBP;PTK2;RTN4;ARHGAP4
response to (	0,00174108	0,04338199	-1,7926533	5,62482791	SLC12A2;KCNMA1;ANXA7;RAC1;SLC12A6;SCN2A;MYLK
regulation of	0,00152598	0,0389972	-1,7022342	5,52249956	CAMK2D;ASPH;DMD;ANK2
establishmen	0,00206084	0,04765578	-1,8058289	5,4964941	ARHGEF11;PARD3;DOCK7;MYO9B;PTK2;SDCCAG8
regulation of	0,00209302	0,04765578	-0,2169679	0,6603962	CAMK2D;ASPH;ANK3
positive regu	0,00149526	0,0385416	-0,1748791	0,5694095	FIS1;MFF;DNM1L
barbed-end a	0,00149526	0,0385416	-0,0983967	0,32038131	GSN;ADD1;ADD2
peroxisome f	0,00209302	0,04765578	-0,0983837	0,29945539	FIS1;MFF;DNM1L
mitochondria	0,00149526	0,0385416	-0,0843019	0,27448839	FIS1;MFF;DNM1L
regulation of	0,00209302	0,04765578	0,00183062	-0,0055719	DNAJC6;TNK2;AAK1
deoxyribonu(	0,00149526	0,0385416	0,03121557	-0,1016384	DGUOK;GUK1;TK2
response to (	0,00101738	0,02987356	0,08965808	-0,3147699	SGIP1;APOE;SLC25A25

Term	P-value	Adjusted P-v	Z-score	Combined Sc	Genes
anchoring jur	3,6933E-10	7,3906E-08	-2,3745516	38,9912687	NCKAP1;RPLP0;DOCK7;TNC;MLLT4;ADD1;ALCAM;YWHAQ;EPB41L2;PPFIA1;PACSIN2;ANXA6;ITGAV;FLNB;RAC1;SPTAN1;PDLIM7;TNS1;ACTR2;RPS9;GSN;DST;MCAM;SORBS1;PTK2;HCK;NFASC;PTPRC;CTTN;APC;ABI2;PARD3;RPL27;PKP4;CALR;ARHGEF7;VCL
adherens jun	5,3684E-10	7,3906E-08	-2,3289731	38,2428481	NCKAP1;RPLP0;DOCK7;TNC;MLLT4;ADD1;ALCAM;YWHAQ;EPB41L2;PPFIA1;PACSIN2;ANXA6;ITGAV;FLNB;RAC1;SPTAN1;PDLIM7;TNS1;ACTR2;RPS9;GSN;DST;MCAM;SORBS1;PTK2;HCK;NFASC;PTPRC;CTTN;APC;ABI2;PARD3;RPL27;CALR;ARHGEF7;VCL
cytosol (GO:0	5,009E-10	7,3906E-08	-2,0785533	34,1308358	CYFIP2;APP;NCKAP1;SAT1;MYLK;COL4A3BP;PSMD6;AP1S2;KPNA5;GYG1;AP2M1;RGS7;CAST;MAP2K4;RPS9;TPM3;AP1B1;ANK2;PHKA2;ATG13;RBX1;ACLY;MYL6;PARD3;AKAP9;RPL27;VCL;IDI1;TNKS;AK2;KLC1;NDRG2;MTMR6;ARHGAP12;TTBK2;DGUOK;NDOR1;PCBP3;EPB41L1;CALD1;KIF3A;PACSIN2;S100A13;MYH11;RIPK1;HMGCLL1;SEC31A;FDPS;CIDEB;MYO5A;UPF3A;PTK2;RPS25;PEX5L;GNAS;PPT1;HNRNPD;CALR;EIF4G3;CDK14;EIF4G2;RPS24;DOCK9;RPLP0;CLTA;ARHGEF10L;IDE;PPCDC;ARHGAP4;UCHL1;PPP3CC;YWHAQ;GUK1;ANXA7;RAC1;LRRFIP1;DNM1L;SPTAN1;ARHGEF11;ACTR2;DST;ITPK1;MINK1;SREBF2;TIRAP;HCK;PKM;KIF2A;EEF1D;DNAJC6;RASA1;DPYD;RAPGEF1;BCAT1;ARHGEF7;SDCCAG8;DYNC1I2;CAMK2D;DCTN1;MLLT4;ADD1;NCALD;DMD;FLNB;CAMK2G;CLASP1;CLASP2;GSN;GK;BAD;CADPS;MYO9B;SORBS1;FMNL1;APC;ABI2;ACIN1;PFKM;TNRC6A;TNRC6B
cell-substrate	5,7141E-09	5,8998E-07	-2,3439371	33,6195009	NCKAP1;RPLP0;DOCK7;TNC;ADD1;ALCAM;YWHAQ;EPB41L2;PPFIA1;PACSIN2;ANXA6;ITGAV;FLNB;RAC1;SPTAN1;PDLIM7;TNS1;ACTR2;RPS9;GSN;DST;MCAM;SORBS1;PTK2;HCK;NFASC;PTPRC;CTTN;RPL27;CALR;ARHGEF7;VCL
cell-substrate	1,6084E-08	1,3285E-06	-2,307048	31,2176681	NCKAP1;RPLP0;DOCK7;TNC;ADD1;ALCAM;YWHAQ;EPB41L2;PPFIA1;ANXA6;PACSIN2;ITGAV;FLNB;RAC1;PDLIM7;TNS1;ACTR2;RPS9;GSN;DST;MCAM;SORBS1;PTK2;HCK;NFASC;PTPRC;CTTN;RPL27;CALR;ARHGEF7;VCL
focal adhesio	3,927E-08	2,7031E-06	-2,270939	29,1159463	NCKAP1;RPLP0;DOCK7;TNC;ADD1;ALCAM;YWHAQ;EPB41L2;PPFIA1;ANXA6;PACSIN2;ITGAV;FLNB;RAC1;PDLIM7;TNS1;ACTR2;RPS9;GSN;DST;MCAM;PTK2;HCK;NFASC;PTPRC;CTTN;RPL27;CALR;ARHGEF7;VCL
axon (GO:00	8,7758E-08	4,7921E-06	-2,099083	25,7107154	APP;SLC38A1;DTNA;DST;TNK2;MYCBP2;DOCK7;CPNE6;COBL;KLC1;UCHL1;ALCAM;BIN1;FEZ1;PPT1;MYH14;NRCAM;SCN2A;MYH10
actomyosin (	9,2825E-08	4,7921E-06	-2,0355774	24,9328642	ABLIM1;TPM3;MYH14;MYO5A;MYH11;FLNB;SORBS1;MYH10;PTK2;DBN1;PDLIM7;MYLK
stress fiber (	1,4135E-06	6,4864E-05	-2,0018254	19,3040231	ABLIM1;TPM3;MYH14;MYH11;FLNB;SORBS1;MYH10;PTK2;PDLIM7;MYLK
synapse (GO:	3,4726E-06	0,00011952	-2,0803529	18,789872	CYFIP2;APP;CAMK2D;DTNA;DENND1A;CADPS;ANK3;MFF;GPHN;EGFLAM;DNAJC6;NSMF;NRCAM;DMD;APBB1;ERC1;DNM1L;MYH10;FGFR2;GRIA4;PRIMA1
actin filamen	2,0191E-06	8,339E-05	-1,9891954	18,6824812	ABLIM1;TPM3;MYH14;MYH11;FLNB;SORBS1;MYH10;PTK2;PDLIM7;MYLK
growth cone	2,8799E-06	0,00010813	-2,0198178	18,4454187	SNAP25;TNK2;DOCK7;COBL;MYO5A;KLC1;ARHGAP4;KIAA1598;APC;PARD3;MYH14;APBB1;MYH10
T-tubule (GO	4,4589E-06	0,00013154	-2,0455168	18,2791833	CACNB1;CAMK2D;BIN1;AHNAK2;ATP2B4;ANK2;ANK3;SCN2A
site of polari	4,0419E-06	0,00012841	-2,0072151	17,9852268	SNAP25;TNK2;DOCK7;COBL;MYO5A;KLC1;ARHGAP4;KIAA1598;APC;PARD3;MYH14;APBB1;MYH10
perinuclear r	9,3184E-06	0,00024053	-2,0869186	17,3895787	CYFIP2;SNAP25;APP;ADD1;COL4A3BP;NDOR1;SPP1;S100A13;ANXA6;UBQLN1;DNM1L;HMGCLL1;SEC31A;ABCA1;TPD52;GSN;COBL;ANK2;MYO9B;TPD52L1;TPD52L2;INF2;GNAS;PKP4;CALR;PAM;DGKI;PICALM
microtubule	2,0255E-05	0,00041827	-2,0447281	15,9067388	DYNC1I2;MACF1;DCTN1;DNAH9;KLC1;SPAST;MAP2;KIF3A;ZNF207;KIF21A;KIF1B;MAP4;KIF1A;DNM1L;CLASP1;CLASP2;SS18;GABARAPL1;NEK7;DNM1;EML4;KIF9;KIF2A;APC;FEZ1
cytoplasmic v	2,3271E-05	0,00044986	-2,0177006	15,5495761	APP;C2CD5;CAMK2D;DENND1A;CLTB;ITPR1;CLTA;NCALD;YWHAQ;AP1S2;ANXA7;KIF1B;APOE;CAMK2G;AP2M1;GRIA4;SEC31A;SGIP1;SYNRG;AHNAK2;FN1;AP1B1;CALR;PAM;PICALM
axon part (GO	1,6534E-05	0,00035939	-1,8624349	14,7711581	CAMK2D;COBL;KCNAB2;ANK3;NFASC;KIAA1598;APC;BIN1;PARD3;EPB41L3;AAK1;NRCAM;MBP;SCN2A
dendrite (GO	2,728E-05	0,00046944	-1,8849046	14,4458516	TNK2;DENND1A;CPNE6;COBL;ADCY2;ANK3;TRIM9;PEX5L;OPA1;ABI2;CHL1;FEZ1;GNAS;PPT1;NSMF;APOE;DBN1;GRIA4;CNTNAP4
coated pit (G	1,3834E-05	0,00033608	-1,7704227	14,160151	APP;SGIP1;TNK2;AP1S2;AAK1;DNM1L;SNAP91;AP2M1;PICALM
extracellular	2,723E-05	0,00046944	-1,8215957	13,9606536	CYFIP2;ITSN2;APP;NCKAP1;FAM49B;FRMPD1;CLSTN1;CPNE6;MYLK;DOCK10;PCMT1;PSMD6;CHCHD3;ALCAM;SCP2;GYG1;AP2M1;SH3GLB1;GEN1;RPS9;SLC38A1;TPM3;APLP2;ATP1B3;ACLY;TOM1L2;MYL6;ADAM15;RPL27;VCL;RTN3;AK2;LTBP3;NDRG2;ATP5O;RTN4;SPAST;PCBP3;KIF3A;EPB41L2;PSAP;PACSIN2;S100A13;MYH14;MYH11;ARSF;APOE;MAP4;MYH10;CLCA4;FN1;MYO5A;HSPA12A;PTPRD;IGSF11;RPS25;PTPRC;GNAS;PPT1;HNRNPD;CALR;PAM;SLC25A3;RPLP0;YBX1;PTBP1;UCHL1;PPP3CC;YWHAQ;CHL1;ANXA6;ANXA7;ITGAV;RAC1;SPTAN1;BCAS1;ACTR2;DST;VPS13C;VPS13D;MINK1;GFRA1;DNM1;PKM;CTTN;PPAP2A;KCNMA1;SFT2D2;ASPA;ITIH4;RAB1A;NAPB;NCALD;SH3BGRL;SPP1;FLNB;NCAM1;SLC25A25;CLASP1;SLC12A2;GSN;GK;ATP2B1;GATM;FMNL1;NFASC;SH3D21;PFKM
membrane c	1,6145E-05	0,00035939	-1,7144386	13,5973845	AP1S2;CLTB;AP1B1;CLTA;SNAP91;DNM1;NCALD;SEC31A;PICALM
cell cortex pa	2,8993E-05	0,00047897	-1,7523748	13,3949439	ACTR2;MAEA;ASPH;MYO1A;EPB41;CALD1;EPB41L2;NSMF;SEPT4;SPTAN1;CLASP1;EXOC1
Z disc (GO:00	2,3963E-05	0,00044986	-1,7216018	13,2676661	DST;BIN1;AHNAK2;ATP2B4;CACNA1D;LDB3;DMD;SORBS2;ANK2;ANK3;SPTAN1
cell-cell junct	5,1396E-05	0,00073195	-1,7972175	12,9755445	APP;PTPRU;CAMK2D;ADCYAP1R1;MAGI3;ANK2;ANK3;SORBS1;MLLT4;MYLK;NFASC;APC;ABI2;PARD3;EPB41L3;NHS;PACSIN2;PKP4;SCN2A;SPTAN1;DBN1;VCL;SDCCAG8
neuronal cell	5,5329E-05	0,00076169	-1,7162122	12,3223519	CAMK2D;TNK2;DENND1A;MYO5A;COBL;ATP2B2;KLC1;RTN4;UCHL1;ALCAM;MAP2;PARD3;PPT1;MBP;APOE;KIF1A;ARHGEF7;MYH10;GTF2I;PICALM
lamellipodiur	5,0544E-05	0,00073195	-1,666671	12,0330251	NCKAP1;ABLIM1;GSN;APC;CTTN;ABI2;MYO9B;APBB1;RAC1;ARHGEF7;PTK2;MYLK
ruffle membr	4,8686E-05	0,00073195	-1,5548282	11,2255429	MACF1;DIAPH1;C2CD5;APC;ITGAV;RAC1;TIRAP;CLASP2
extrinsic com	7,1631E-05	0,00095431	-1,5572589	10,8299962	HCK;DTNA;EPB41L1;EPB41;EPB41L2;EPB41L3;PACSIN2;AAK1;GFRA1;WDFY3;APOE;RAC1
cell body (GC	0,00016229	0,00203109	-1,6594273	10,2870942	CAMK2D;GABARAPL1;TNK2;DENND1A;MYO5A;COBL;ATP2B2;KLC1;RTN4;UCHL1;ALCAM;MAP2;PARD3;PPT1;MBP;APOE;KIF1A;ARHGEF7;MYH10;GTF2I;PICALM
synapse part	0,00023283	0,00267107	-1,7050776	10,1030556	SNAP25;MINK1;CLSTN1;ITPR1;SEMA4F;ANK2;ANK3;ADD1;GPHN;RIMS1;TRIM9;SIPA1L1;BIN1;KIF3A;DMXL2;PPFIA1;PPT1;AAK1;DMD;NSMF;ERC1;PICALM;GRIA4;CNTNAP4
contractile fil	0,00019996	0,00242894	-1,6091532	9,68758735	DST;TPM3;AHNAK2;ATP2B4;CACNA1D;LDB3;SORBS2;ANK2;ANK3;BIN1;MYH11;DMD;SPTAN1;VCL
actin cytoske	0,00027494	0,00291153	-1,5416105	9,00158154	ACTR2;GSN;DST;MYO9B;SORBS2;ABLIM1;MAEA;BIN1;CALD1;DMD;FLNB;PPP1R12B;DBN1;PDLIM7;VCL
leading edge	0,00026364	0,00286531	-1,4525053	8,50453405	DIAPH1;MACF1;C2CD5;GABARAPL1;APC;EPB41L3;ITGAV;RAC1;TIRAP;CLASP2
microtubule	0,00040228	0,00395576	-1,4029156	7,76174765	DYNC1I2;KIF9;KIF2A;KIF3A;DCTN1;MAP2;DNAH9;KIF21A;KIF1B;KIF1A;MAP4;KLC1
cytoplasmic v	0,00057221	0,00513741	-1,4343009	7,56049491	C2CD5;CAMK2D;DENND1A;AHNAK2;ITPR1;AP1B1;CLTA;YWHAQ;AP1S2;ANXA7;KIF1B;PAM;CAMK2G;AP2M1;GRIA4;SEC31A
intracellular	0,00092673	0,00765482	-1,4924602	7,27189306	ZNF573;HKR1;PRKCSH;ZNF43;ADCY2;SAT1;RTN4;NCALD;ARHGAP4;SENP7;UCHL1;FSD1L;TRIM4;ZNF823;G3BP2;BCO2;ZNF620;ARHGEF11;GABARAPL1;ZNF283;ITPK1;CIDEB;MYO9B;ANK2;PARP8;TOM1L2;NFASC;RNF207;RASA1;RAPGEF1;ZNF559;CALR;RAPGEF6;DBN1;ZNF211
cell cortex (G	0,00042528	0,00408464	-1,2648892	6,95755067	C2CD5;CTTN;PARD3;COBL;MYO9B;MYH10;CLASP1;FGFR2;CLASP2
microtubule	0,00044365	0,00416424	-1,2642581	6,92967957	THAP6;SPAST;DST;KIF3A;FEZ1;MYCBP2;CCSER2;TACC1;MAP4;DRAM2;SPTAN1
axon initial s	5,9495E-06	0,00016381	-0,7867784	6,85819658	CAMK2D;NFASC;BIN1;NRCAM;ANK3
cell-cell adhe	0,00026232	0,00286531	-1,1237267	6,57950903	ABI2;APC;PARD3;SORBS1;SPTAN1;MLLT4;VCL
myosin comp	0,00033426	0,00336708	-1,1483791	6,53853812	MYL6;MYO15B;MYO1A;MYH14;MYO5A;MYH11;MYO9B;MYH10
vesicle mem	0,00139047	0,01066327	-1,3273091	6,0272444	C2CD5;CAMK2D;DENND1A;AHNAK2;ITPR1;AP1B1;CLTA;YWHAQ;AP1S2;ANXA7;KIF1B;PAM;CAMK2G;AP2M1;GRIA4;SEC31A
membrane ra	0,00121585	0,00965669	-1,2860601	5,96745332	ABCA1;APP;ADCYAP1R1;ATP2B4;ATP1B3;ADCY2;ANK2;SORBS1;HCK;KCNMA1;PPT1;PACSIN2;DMD;RIPK1
synaptic mem	0,00153803	0,01114399	-1,2310699	5,53594315	SNAP25;MINK1;CLSTN1;SEMA4F;ANK2;ANK3;GPHN;RIMS1;SIPA1L1;DMD;NSMF;ERC1;PICALM;GRIA4;CNTNAP4
mitochondria	0,00178578	0,01250043	-1,2491665	5,47383803	SH3GLB1;SLC25A3;GK;BAD;NDUFB5;MGST1;AK2;ABCA8;PPOX;MFF;ATP5O;SDHA;UQCRH;PGS1;GATM;CHCHD3;OPA1;CSDE1;OGDH;NDUFS2;TK2;NDUFV3;DNM1L;SLC25A25
kinesin comp	0,00055857	0,00512643	-0,9880963	5,2105729	KIF9;KIF2A;KIF3A;KIF21A;KIF1B;KIF1A;KLC1
Golgi appara	0,00270251	0,01829732	-1,1750254	4,70127754	RTN3;APP;MACF1;TNKS;CLSTN1;NDRG2;COL4A3BP;GOLGA4;CCDC91;PLAGL1;DYM;DENND5A;APOE;ERC1;DRAM2;DNM1L;CLASP1;CLASP2;OSBPL9;ABCA1;SH3GLB1;IL15RA;GABARAPL1;FBXW7;SYNRG;MINK1;MYO5A;PDE4DIP;PPHLN1;ANK3;HCK;TRAPPC6B;CSDE1;AKAP9;PPT1;CALR;FGFR2;PICALM
trans-Golgi n	0,00165625	0,01179367	-1,007989	4,47566515	SNAP25;GOLGA4;CYTH4;SLC9A7;PIFO;CLTB;FAM109A;RAC1;PAM;CLASP2
mitochondrio	0,00354185	0,0235933	-1,1628293	4,35687993	SLC25A3;FASTKD1;IDE;TMEM143;COL4A3BP;CHCHD3;OPA1;SCP2;C14ORF2;DBT;KIF1B;ME3;ME2;DNM1L;AP2M1;SEPT4;SDHA;CA5BP1;ACLY;PKM;NDUFS2;MICU3;CYP2E1;BCAT1;IDI1;NDUFB5;MGST1;MTIF2;ATP5O;LIAS;UQCRH;TRAK2;PGS1;APOO;DGUOK;MOBP;PSAP;SH3BP5;TDH;RIPK1;NDUFV3;BCO2;SLC25A25;FIS1;FDPS;BAD;OXR1;GATM;CAPRIN2;OGDH;OCIAD1
clathrin coat	5,0228E-05	0,00073195	-0,5720576	4,13013955	CLTB;CLTA;SNAP91;NCALD;PICALM
cytoplasmic r	0,00455995	0,02942596	-1,160221	4,09079786	ABCA1;RAB1A;APP;PIGT;DST;SGIP1;TNK2;DENND1A;MYO5A;CLTA;FAM109A;TIRAP;ADD2;TRIM9;FMNL1;BIN1;KIF3A;DMXL2;PPT1;AAK1;ITGAV;CALR;RAC1;SEC31A
organelle inn	0,00487484	0,0300494	-1,0889202	3,81657015	SLC25A3;NDUFB5;MGST1;AK2;ITPR1;ABCA8;ATP5O;SDHA;UQCRH;PGS1;GATM;CHCHD3;OPA1;CSDE1;NDUFS2;TK2;MATR3;NDUFV3;SLC25A25
intrinsic com	0,00107441	0,00870062	-0,7992459	3,79191101	RASA1;GNAS;FLT3LG;CYP2E1;PPOX;SCN2A;PRIMA1
AP-type mem	0,00060481	0,00531458	-0,6347724	3,32449404	SGIP1;SYNRG;AP1S2;AP1B1;AP2M1;PICALM
sarcoplasmic	0,00069536	0,005983	-0,6116443	3,13090498	CACNB1;CAMK2D;ITPR1;ANK3;CALR;CAMK2G
costamere (G	0,00020902	0,00246643	-0,5147281	3,09093441	AHNAK2;ANK2;DMD;ANK3;VCL
ruffle (GO:00	0,00247551	0,01703973	-0,724795	2,95151563	GSN;CTTN;RASA1;MYO5A;COBL;MYO9B;PDLIM7
cell projectio	0,00766913	0,04280204	-0,8850126	2,78882489	DIAPH1;MACF1;C2CD5;GABARAPL1;APC;EPB41L3;CLTB;DMD;ITGAV;RAC1;TIRAP;CLASP2
microbody (G	0,006563	0,03826674	-0,8053705	2,62806408	IDI1;FIS1;PEX5L;SCP2;MGST1;MYO5A;IDE;MFF;DNM1L
coated vesicl	0,0043081	0,02824196	-0,7250843	2,58633705	APP;SGIP1;DENND1A;TNK2;AAK1;FAM109A;SEC31A
caveola (GO:	0,00485765	0,0300494	-0,4809476	1,68567938	HCK;ADCYAP1R1;KCNMA1;PACSIN2;ATP2B4;ATP1B3
plasma mem	0,00485765	0,0300494	-0,4524408	1,58576553	HCK;ADCYAP1R1;KCNMA1;PACSIN2;ATP2B4;ATP1B3
clathrin adap	0,00139423	0,01066327	-0,0500579	0,22731031	SGIP1;SYNRG;AP1B1;AP2M1;PICALM
presynaptic r	0,0066712	0,03826674	0,0517145	-0,1687534	RIMS1;SNAP25;ERC1;CNTNAP4;PICALM
cell-cell cont	0,0066712	0,03826674	0,08565169	-0,2794964	CAMK2D;PKP4;ANK2;ANK3;SCN2A
lateral plasm	0,00805555	0,04435922	0,15691116	-0,4888465	APC;MYO1A;DMD;ANK3;SPTAN1
cell division s	0,00581735	0,03533186	0,80153672	-2,6795133	MAEA;MYH10;RAB11FIP3;MYLK
mitotic spind	0,00664316	0,03826674	0,83101637	-2,7117511	EML4;DIAPH1;PKP4;MAP4
node of Ranv	0,00076455	0,00644406	0,95586022	-4,8219299	NFASC;BIN1;ANK3;SCN2A
axonal growt	0,00032055	0,00330963	1,29799883	-7,4127644	KIAA1598;APC;PARD3;COBL
paranode reg	0,00015452	0,00199423	1,2253393	-7,6185434	NFASC;EPB41L3;ANK3;SCN2A
microtubule	0,00717071	0,04056852	3,54129797	-11,34902	DST;APC;MYO5A
clathrin vesic	0,00149526	0,01102754	5,23792486	-23,609212	CLTB;CLTA;NCALD
spectrin (GO:	0,00149526	0,01102754	5,88460255	-26,524021	EPB41;EPB41L2;SPTAN1

Term	P-value	Adjusted P-v	Z-score	Combined Sc	Genes
actin binding	2,1787E-14	1,3334E-11	-2,3955872	59,9872638	MACF1;TAGLN;EPB41;ADD1;NCALD;ADD2;MYLK;SNTG2;ABLIM1;MAEA;EPB41L1;CALD1;EPB41L2;EPB41L3;MYH14;PLS3;PHACTR2;DMD;FLNB;MYH11;MYH10;SPTAN1;TNS1;ACTR2;GSN;DST;TPM3;MYO5A;MICAL2;COBL;MYO9B;SORBS1;SSH2;PTK2;DIAPH1;FMNL1;FMNL2;INF2;MYO1A;KCNMA1;DBN1;VCL
GTPase bindi	1,415E-11	4,3298E-09	-2,470635	47,5788634	NCKAP1;ADCYAP1R1;DOCK9;DOCK7;IPO4;DOCK10;RIMS1;GOLGA4;MOBP;DENND5A;ERC1;RAC1;PAK3;RAB11FIP3;ABCA1;PIFO;MYO5A;DIAPH1;FMNL1;PEX5L;FMNL2;INF2;ABI2;BIN1;RASA1;DMXL2;RAPGEF6;DGKI
small GTPase	6,0956E-10	1,2435E-07	-2,4434734	38,8516431	NCKAP1;ADCYAP1R1;DOCK9;DOCK7;IPO4;DOCK10;RIMS1;MOBP;DENND5A;ERC1;RAC1;PAK3;RAB11FIP3;ABCA1;PIFO;MYO5A;DIAPH1;PEX5L;FMNL1;FMNL2;INF2;ABI2;DMXL2;RAPGEF6;DGKI
calmodulin bi	8,1323E-09	1,2442E-06	-2,3710847	32,2396094	CAMK2D;ARPP21;EPB41;ATP2B4;MYO5A;ATP2B2;MYO9B;ATP2B1;PHKA2;ADD1;ADD2;MYLK;PPP3CC;MYO1A;MAP2;CALD1;MYH14;MYH11;CAMK2G;SPTAN1;MYH10
Ras GTPase b	1,6057E-08	1,9654E-06	-2,3668996	31,1005977	NCKAP1;DOCK9;DOCK7;PIFO;MYO5A;IPO4;DOCK10;DIAPH1;RIMS1;FMNL1;FMNL2;INF2;MOBP;ABI2;DMXL2;DENND5A;ERC1;RAC1;PAK3;RAPGEF6;RAB11FIP3;DGKI
motor activit	2,0087E-08	2,0488E-06	-2,2248459	29,1415483	DYNC1I2;DCTN1;MYO5A;DNAH9;MYO9B;KLC1;MYL6;KIF9;KIF2A;MYO15B;KIF3A;MYO1A;MYH14;MYH11;KIF21A;KIF1B;KIF1A;MYH10
tubulin bindi	2,0785E-07	1,8172E-05	-2,3543546	25,6992083	MACF1;GABARAPL1;DST;SGIP1;PIFO;KLC1;NCALD;CAMSAP2;KIF9;SPAST;KIF2A;APC;KIF3A;MAP2;FEZ1;ZNF207;CCSER2;KIF1B;KIF21A;KIF1A;MAP4;CLASP1;CLASP2
spectrin bind	8,9574E-07	6,8524E-05	-2,5241164	24,2020535	EPB41;KIF3A;ANK2;ANK3;SPTAN1;ADD1;ADD2
microtubule	1,0587E-06	7,1994E-05	-2,3107648	22,0422274	MACF1;DST;SGIP1;CAMSAP2;KIF9;SPAST;KIF2A;APC;KIF3A;MAP2;ZNF207;CCSER2;KIF21A;KIF1B;KIF1A;MAP4;CLASP1;CLASP2
ATPase activi	1,2649E-05	0,00071071	-2,4609216	17,8398306	MACF1;DNAH9;IDE;ATP5O;PSMD6;RNF213;SPAST;KIF3A;HLTF;MYH14;KIF21A;KIF1B;KIF1A;MYH10;PMS1;ABCA1;ATP2B4;ATP11C;ATP1B3;ATP2B2;MYO9B;ABCA8;ATP2B1;MYL6;KIF9;KIF2A;ACIN1
kinase bindin	3,1091E-05	0,00146366	-2,4096157	15,7271214	PRKCSH;LDB3;CCND3;UBQLN1;ITGAV;RAC1;SLC12A6;GTF2I;MAP2K4;BAD;PIFO;CADPS;AP1B1;ANK2;SORBS1;ATG13;SNAP91;DNM1;TIRAP;PTK2;TOM1L2;PTPRC;KIAA1598;APC;ABI2;FEZ1;PARD3;ARHGEF7;PAM;PFKM
ATP binding (	2,0453E-05	0,00104311	-2,2620097	15,5299433	IDE;GPHN;MYLK;GUK1;TK2;KIF21A;KIF1B;KIF1A;CDKL3;CSNK1G3;ACTR2;MAP2K4;CERS5;MAGI3;ITPK1;MINK1;ATP11C;ACLY;HCK;KIF9;PKM;KIF2A;NAV3;DGKI;CAMK2D;TARSL2;AK2;DNAH9;ADCY2;TTL;TTBK2;PGS1;DGUOK;SPAST;KIF3A;HLTF;MYH14;MYH11;RIPK1;PAK3;CAMK2G;MYH10;MARK3;PMS1;MAP4K4;ABCA1;GK;TNK2;NEK7;ATP2B4;DCLK2;MYO5A;MYO9B;ATP2B2;ABCA8;ATP2B1;HSPA12A;PTK2;DCLK1;HIPK3;MYO15B;MYO1A;WNK1;WNK2;AAK1;CDK14;PFKM;FGFR2
Rho GTPase b	1,2774E-05	0,00071071	-2,0358085	14,7580804	DOCK10;NCKAP1;DIAPH1;FMNL1;FMNL2;INF2;ABI2;DOCK9;DOCK7;PAK3
phospholipid	6,7754E-05	0,00296184	-2,3605426	13,7429487	ABCA1;C2CD5;MCTP1;EPB41;SGIP1;BAD;CPNE6;ITPR1;PLEKHA4;SNAP91;TIRAP;COL4A3BP;SH3PXD2A;PARD3;SNX14;ANXA6;PACSIN2;ANXA7;WDFY3;APOE;PICALM
protein kinas	0,00018076	0,0065075	-2,3456957	11,8101085	PRKCSH;LDB3;CCND3;ITGAV;RAC1;SLC12A6;GTF2I;MAP2K4;BAD;PIFO;CADPS;AP1B1;ANK2;SORBS1;ATG13;SNAP91;TIRAP;DNM1;PTK2;TOM1L2;PTPRC;APC;PARD3;FEZ1;ARHGEF7;PAM
calcium ion b	0,0002034	0,00673262	-2,1616799	10,8101092	ITSN2;MACF1;C2CD5;MCTP1;PRKCSH;CLSTN1;CPNE6;EFCAB14;LTBP3;NCALD;PGS1;MMP28;ANXA6;S100A13;ENPP2;ANXA7;PLS3;DMD;RAB11FIP3;SPTAN1;SLC25A25;EDEM3;TPD52;CGREF1;GSN;DTNA;DST;SYNRG;MYO5A;ATP2B2;MYL6;ASPH;MICU3;EFHB;CALR;PAM
1-phosphatid	0,00020902	0,00673262	-2,1366511	10,6849453	EPB41;WDFY3;PLEKHA4;SNAP91;PICALM
microtubule	0,00015452	0,00591026	-1,9006158	9,75218343	DST;APC;CLASP1;CLASP2
microtubule	0,00026604	0,00814071	-1,9917977	9,58229615	DYNC1I2;KIF9;KIF2A;KIF3A;KIF21A;KIF1B;DNAH9;KIF1A;KLC1
actin filamen	0,00033981	0,00990306	-2,007754	9,26560657	MACF1;ACTR2;FMNL1;MYH14;MYO5A;MYH11;MYO9B;MYH10;ADD1;ADD2
Rab GTPase b	0,00046502	0,01293613	-1,9610734	8,52622042	RIMS1;MOBP;PIFO;DMXL2;MYO5A;DENND5A;ERC1;RAC1;RAB11FIP3
Ras guanyl-n	0,00062761	0,01669977	-2,0435686	8,36301912	ITSN2;ARHGEF11;DOCK10;DENND1C;PLEKHG4;DOCK9;DENND1A;RAPGEF1;ARHGEF10L;DENND5A;ARHGEF7
clathrin bindi	0,0010191	0,023988	-2,2412254	8,36022278	TOM1L2;CLTA;SNAP91;NCALD;PICALM
guanyl-nucle	0,00100133	0,023988	-2,1951124	8,18821192	ARHGEF11;ITSN2;DENND1C;PLEKHG4;DOCK9;DENND1A;DOCK7;ARHGEF10L;DOCK10;CYTH4;RAPGEF1;DENND5A;ARHGEF7;RAPGEF6
protein serin	0,0014132	0,02865718	-2,1761416	7,73041958	CDKL3;MAP2K4;CSNK1G3;CAMK2D;CERS5;TNK2;NEK7;MINK1;DCLK2;PHKA2;HIPK3;DCLK1;TTBK2;MYLK;CCND3;WNK1;WNK2;AAK1;RIPK1;PAK3;CAMK2G;CDK14;MARK3;MAP4K4
actin-depend	0,00015452	0,00591026	-1,4990008	7,69147068	MYL6;MYH14;MYO9B;MYH10
protein bindi	0,00111811	0,02534384	-1,9120115	7,02706175	ITSN2;SH3BGRL;ABI2;FBXW7;SORBS2;ANK2;SORBS1;ANK3;TIRAP;ARHGAP4;PICALM
protein homo	0,00207616	0,03732878	-2,0663981	6,79429766	CAMK2D;HM13;PLEKHB1;MYCBP2;MGST1;FAM109A;IDE;MFF;ADD1;ADD2;TRIM9;ANXA6;S100A13;APOE;LRRFIP1;DNM1L;RAB11FIP3;CAMK2G;SH3GLB1;TPD52;DST;FLT3LG;MYO9B;TPD52L1;TPD52L2;SUMF2;TIRAP;SLC9A7;DPYD;PFKM;FGFR2
ion channel b	0,00149841	0,02865718	-1,8671281	6,63269474	RIMS1;DIAPH1;CAMK2D;ASPH;PRKCSH;AKAP9;ANK2;ANK3
protein kinas	0,00131552	0,02865718	-1,8267449	6,48923957	PRKCSH;PARD3;FEZ1;LDB3;ITGAV;TIRAP
cell adhesion	0,00224292	0,03732878	-1,9112936	6,28431539	PTPRD;DST;APC;ADAM15;ADAM23;FN1;ADAM22;ANXA7;ANK3;CALR;MLLT4;VCL
ion channel i	0,00213517	0,03732878	-1,8752923	6,16594331	CAMK2D;WNK1;RASA1;ENSA;ITPR1
binding, brid	0,00222534	0,03732878	-1,8358909	6,03639193	ITSN2;SH3BGRL;ABI2;FBXW7;SORBS2;ANK2;SORBS1;ANK3;TIRAP;ARHGAP4;PICALM
channel inhib	0,00243604	0,03923313	-1,8120493	5,86783939	CAMK2D;WNK1;RASA1;ENSA;ITPR1
coenzyme bi	0,00556083	0,07914485	-1,8578711	4,71244449	NDOR1;SCP2;DPYD;OGDH;MICAL2;TDH;NDUFS2;ME3;ME2;FMO4;PPOX;SDHA
microfilamen	0,00225681	0,03732878	-1,412186	4,64325437	MYH14;MYO5A;MYO9B;MYH10
Rho guanyl-n	0,00498334	0,07557254	-1,7625374	4,55203885	ITSN2;DOCK10;ARHGEF11;PLEKHG4;DOCK9;ARHGEF10L;ARHGEF7
SH3 domain b	0,00530331	0,0772768	-1,7384638	4,45109596	SH3BGRL;ADAM15;ABI2;DNAJC6;SGIP1;DENND1A;RAPGEF1;SH3BP5;PAK3
structural co	0,00492031	0,07557254	-1,6440037	4,24590638	MYL6;ASPH;MYH11;DMD;SORBS2
channel regu	0,00675631	0,08988824	-1,7543969	4,22667209	CAMK2D;WNK1;RASA1;ENSA;ATP2B4;ITPR1;ANK2;KCNAB2
structural co	0,00600823	0,08356904	-1,7027583	4,22638596	ACTR2;EPB41;EPB41L3;DMD;SORBS2;ANK2;ANK3;SPTAN1
nucleoside-tr	0,00942164	0,11078678	-1,8943546	4,16786021	ARHGEF11;TNK2;RALGAPA2;MINK1;ARHGEF10L;ATP1B3;MYO9B;SIPA1L2;ARHGAP12;ARHGAP4;RIMS1;SIPA1L1;RASA1;DGKI;AGFG1;MAP4K4;RGS7
glycoprotein	0,00656883	0,08933607	-1,6644595	4,02025211	PTPRC;MAP2;RASA1;DMD;IDE;CALR;VCL
calmodulin-d	0,00506287	0,07557254	-1,5275359	3,94510945	CAMK2D;CAMK2G;PHKA2;MYLK
GTPase regul	0,01136553	0,12238086	-1,8393509	3,86377216	ARHGEF11;TNK2;RALGAPA2;MINK1;ARHGEF10L;MYO9B;SIPA1L2;ARHGAP12;ARHGAP4;RIMS1;SIPA1L1;RASA1;DGKI;AGFG1;MAP4K4;RGS7
phosphatidyl	0,0097753	0,11078678	-1,7231698	3,79122831	COL4A3BP;SH3PXD2A;EPB41;SNX14;PARD3;ITPR1;WDFY3;PLEKHA4;SNAP91;TIRAP;PICALM
cation-transp	0,0084562	0,10223399	-1,5113105	3,44653007	ATP2B4;ATP11C;ATP1B3;ATP2B2;ATP2B1;ATP5O
beta-amyloid	0,00754301	0,09617337	-1,4177335	3,3197686	CLSTN1;IDE;APOE;APBB1
ATPase activi	0,00976435	0,11078678	-1,476834	3,24925317	ATP2B4;ATP11C;ATP1B3;ATP2B2;ATP2B1;ATP5O
kinesin bindi	0,00754301	0,09617337	-1,3637055	3,19325651	PIFO;CLSTN1;KIF1B;KTN1
ATPase activi	0,0085195	0,10223399	-1,3683263	3,12045578	ATP2B4;ATP1B3;ATP2B2;ATP2B1
Rac GTPase b	0,0085195	0,10223399	-1,2497686	2,85008603	NCKAP1;FMNL1;ABI2;DOCK7
nucleobase-c	0,01139822	0,12238086	-1,3539034	2,84403292	DGUOK;GUK1;MAGI3;AK2;TK2
peptide bindi	0,01973045	0,18576981	-1,6003613	2,69380329	PEX5L;ADCYAP1R1;CLSTN1;MGST1;CLTB;CLTA;APOE;APBB1;IDE;CALR
flavin adenin	0,01547552	0,15785032	-1,4156832	2,61350414	NDOR1;DPYD;MICAL2;PPOX;FMO4;SDHA
amide bindin	0,02352738	0,19836213	-1,5193871	2,45785318	PEX5L;ADCYAP1R1;CLSTN1;MGST1;CLTB;CLTA;APOE;APBB1;IDE;CALR
calcium ion t	0,02306997	0,19836213	-1,4335376	2,31897782	CACNB1;ATP2B4;ITPR1;ATP2B2;CACNA1D;DENND5A;ITGAV;ATP2B1
integrin bindi	0,02152988	0,19666097	-1,3978175	2,27323432	DST;ADAM15;ADAM23;FN1;ADAM22;ANXA7;CALR
ATPase activi	0,02366084	0,19836213	-1,3551085	2,19210622	ATP2B4;ATP11C;ATP1B3;ATP2B2;ATP2B1;ABCA8;ATP5O
ATPase activi	0,02477892	0,20492834	-1,3553701	2,14839027	ATP2B4;ATP11C;ATP1B3;ATP2B2;ATP2B1;ABCA8;ATP5O
cofactor bind	0,03116513	0,23841322	-1,4736134	2,11279308	MICAL2;FMO4;PPOX;SDHA;ACLY;NDOR1;SCP2;DPYD;OGDH;TDH;NDUFS2;ME3;ME2
hydrolase act	0,02834913	0,22353099	-1,3565698	2,03241992	ATP2B4;ATP11C;ATP1B3;ATP2B2;ATP2B1;ABCA8;ATP5O
calcium-depe	0,01611641	0,16169247	-1,0982833	2,00113699	C2CD5;MCTP1;ANXA6;ANXA7
calcium-depe	0,01800766	0,17775303	-1,130236	1,95232463	SNAP25;ANXA6;ANXA7;NSMF;SEC31A
P-P-bond-hyc	0,03225247	0,24071353	-1,308333	1,86325941	ATP2B4;ATP11C;ATP1B3;ATP2B2;ATP2B1;ABCA8;ATP5O
primary activ	0,03225247	0,24071353	-1,2938469	1,84262908	ATP2B4;ATP11C;ATP1B3;ATP2B2;ATP2B1;ABCA8;ATP5O
enzyme inhib	0,04299732	0,2829501	-1,3670916	1,7259322	CAST;PPP1R36;APP;ITIH4;TNK2;APLP2;ATP2B4;WNK1;ENSA;SH3BP5;SSPO;PHACTR2;PKIG;RECK;DGKI
phosphatase	0,03290102	0,24259549	-1,1760557	1,66571805	PPP1R36;CHCHD3;AKAP11;WNK1;BAD;PARD3;ENSA;ATP2B4
potassium ch	0,02297378	0,19836213	-1,0167764	1,64479952	RASA1;ENSA;ANK2;KCNAB2
oxidoreducta	0,02297378	0,19836213	-0,9796021	1,58466405	NDOR1;MICAL2;CYP2E1;FMO4
metal ion tra	0,04813325	0,29755098	-1,2732952	1,54344982	SLC38A1;ATP2B4;ITPR1;ATP1B3;CACNA1D;ATP2B2;KCNAB2;ATP2B1;MTMR6;CACNB1;SLC9A7;KCNMA1;SCN9A;ITGAV;DENND5A;SCN2A;SLC12A6
phosphoprot	0,04639414	0,29601774	-1,16955	1,42373523	PGS1;PTPRD;PTPRU;PTPRC;PPP3CC;DNAJC6;SSH2;CAMK2G;MTMR6
protein meth	0,04653504	0,29601774	-0,940446	1,14483872	PCMT1;NTMT1;KMT2C;WHSC1;PCMTD1
SH3/SH2 ada	0,04692253	0,29601774	-0,5516707	0,67156854	ITSN2;SH3BGRL;SORBS1;ARHGAP4
nucleotide ki	0,02108393	0,19550549	-0,3752998	0,6125518	GUK1;MAGI3;AK2
carbon-carbo	0,04986389	0,30516698	-0,5154236	0,61175436	ME3;ME2;HMGCLL1;PPCDC
transmembra	0,01210511	0,12556489	-0,2709868	0,56227936	PTPRD;PTPRU;PTPRC
transmembra	0,01210511	0,12556489	-0,2288193	0,47478455	PTPRD;PTPRU;PTPRC
plus-end-dire	0,01029276	0,11453039	-0,1632867	0,35382839	KIF3A;KIF1B;KIF1A
Rab guanyl-n	0,03980147	0,27161534	-0,0402477	0,05245759	DENND1C;DENND1A;DENND5A
cadherin binc	0,03624879	0,25209386	-0,023075	0,03179623	APC;ANK3;VCL
calcium-trans	0,00149526	0,02865718	0,48569077	-1,7253442	ATP2B4;ATP2B2;ATP2B1
protein bindi	0,00149526	0,02865718	0,58307518	-2,0712879	NFASC;ITGA10;NRCAM
profilin bindi	0,00101738	0,023988	0,57893102	-2,1595294	DIAPH1;FMNL1;DBN1
alkali metal i	0,04740153	0,29601774	3,52973668	-4,2968752	PKM;SCN9A
GABA recept	0,04740153	0,29601774	3,73400079	-4,5455332	GABARAPL1;TRAK2
nitric-oxide s	0,04083106	0,27161534	3,73218605	-4,8644134	ATP2B4;DMD
sterol transp	0,04083106	0,27161534	3,85496245	-5,0244363	ABCA1;APOE
nucleoside ki	0,04083106	0,27161534	4,50843553	-5,8761525	DGUOK;TK2
tau protein b	0,02885449	0,22353099	3,95776466	-5,9295436	BIN1;APOE
adenylate cyc	0,03463994	0,24367408	4,23737339	-5,9828478	GNAS;ADCY2
opsonin bind	0,03463994	0,24367408	4,28637851	-6,0520393	ITGAV;CALR
cholesterol tr	0,03463994	0,24367408	4,35387824	-6,1473437	ABCA1;APOE
high voltage-	0,02885449	0,22353099	4,1886928	-6,2755214	CACNB1;CACNA1D
phosphatidat	0,03463994	0,24367408	4,48833423	-6,3371853	PPAP2A;LPIN1
adenylate cyc	0,02885449	0,22353099	4,45539067	-6,6750895	ADCYAP1R1;ADCY2
guanylate kin	0,02885449	0,22353099	4,48308167	-6,7165763	GUK1;MAGI3
potassium ch	0,0235022	0,19836213	5,22970488	-8,4598896	RASA1;ENSA
cation:chloric	0,01861177	0,17797501	5,27824482	-9,1108425	SLC12A2;SLC12A6
CDP-alcohol	0,01861177	0,17797501	5,98417207	-10,329352	PGS1;CHPT1

Supplementary Table 11

	Pathway	Padj
**1**	PI3K-Akt signaling pathway (hsa04151)	2,60E-27
**2**	Neurotrophin signaling pathway (hsa04722)	1,39E-19
**3**	Axon guidance (hsa04360)	1,39E-19
**4**	TGF-beta signaling pathway (hsa04350)	3,04E-18
**5**	Focal adhesion (hsa04510)	3,13E-18
**6**	Insulin signaling pathway (hsa04910)	5,61E-18
**7**	Transcriptional misregulation in cancer (hsa05202)	4,20E-16
**8**	MAPK signaling pathway (hsa04010)	1,59E-15
**9**	Pathways in cancer (hsa05200)	1,59E-15
**10**	Regulation of actin cytoskeleton (hsa04810)	1,92E-15
**11**	mRNA surveillance pathway (hsa03015)	5,06E-15
**12**	Prostate cancer (hsa05215)	6,78E-15
**13**	Long-term potentiation (hsa04720)	2,29E-14
**14**	Wnt signaling pathway (hsa04310)	9,22E-12
**15**	Small cell lung cancer (hsa05222)	5,84E-11
**16**	Dopaminergic synapse (hsa04728)	7,64E-10
**17**	T cell receptor signaling pathway (hsa04660)	1,17E-09
**18**	mTOR signaling pathway (hsa04150)	1,65E-09
**19**	Chronic myeloid leukemia (hsa05220)	3,53E-09
**20**	Gap junction (hsa04540)	4,16E-09
**21**	Ubiquitin mediated proteolysis (hsa04120)	1,12E-08
**22**	Arrhythmogenic right ventricular cardiomyopathy (ARVC) (hsa05412)	1,36E-08
**23**	Endocytosis (hsa04144)	1,70E-08
**24**	Renal cell carcinoma (hsa05211)	2,30E-08
**25**	B cell receptor signaling pathway (hsa04662)	3,55E-08
**26**	Glutamatergic synapse (hsa04724)	6,62E-08
**27**	Non-small cell lung cancer (hsa05223)	3,60E-07
**28**	Melanoma (hsa05218)	3,60E-07
**29**	Hedgehog signaling pathway (hsa04340)	3,64E-07
**30**	Dorso-ventral axis formation (hsa04320)	5,30E-07
**31**	RNA degradation (hsa03018)	5,30E-07
**32**	Hepatitis B (hsa05161)	9,37E-07
**33**	p53 signaling pathway (hsa04115)	1,25E-06
**34**	Shigellosis (hsa05131)	2,01E-06
**35**	Acute myeloid leukemia (hsa05221)	3,30E-06
**36**	Hepatitis C (hsa05160)	3,93E-06
**37**	Circadian rhythm (hsa04710)	3,96E-06
**38**	Bacterial invasion of epithelial cells (hsa05100)	3,96E-06
**39**	Adherens junction (hsa04520)	3,96E-06
**40**	Fc gamma R-mediated phagocytosis (hsa04666)	4,19E-06
**41**	Adipocytokine signaling pathway (hsa04920)	5,47E-06

**42**	Gastric acid secretion (hsa04971)	7,57E-06
**43**	ErbB signaling pathway (hsa04012)	8,72E-06
**44**	Inositol phosphate metabolism (hsa00562)	1,11E-05
**45**	Endometrial cancer (hsa05213)	1,78E-05
**46**	Herpes simplex infection (hsa05168)	2,78E-05
**47**	Tight junction (hsa04530)	4,15E-05
**48**	Vasopressin-regulated water reabsorption (hsa04962)	4,48E-05
**49**	Colorectal cancer (hsa05210)	5,36E-05
**50**	HTLV-I infection (hsa05166)	6,31E-05
**51**	Phosphatidylinositol signaling system (hsa04070)	9,55E-05
**52**	VEGF signaling pathway (hsa04370)	0,00019505
**53**	Long-term depression (hsa04730)	0,000207185
**54**	Regulation of autophagy (hsa04140)	0,000299943
**55**	Hypertrophic cardiomyopathy (HCM) (hsa05410)	0,000636027
**56**	RNA transport (hsa03013)	0,000770108
**57**	Melanogenesis (hsa04916)	0,000792673
**58**	ABC transporters (hsa02010)	0,000939598
**59**	Endocrine and other factor-regulated calcium reabsorption (hsa04961)	0,001109559
**60**	Basal cell carcinoma (hsa05217)	0,001408197
**61**	Prion diseases (hsa05020)	0,002224552
**62**	Retrograde endocannabinoid signaling (hsa04723)	0,002414814
**63**	Osteoclast differentiation (hsa04380)	0,002995736
**64**	Dilated cardiomyopathy (hsa05414)	0,003054503
**65**	Jak-STAT signaling pathway (hsa04630)	0,00345273
**66**	Pentose phosphate pathway (hsa00030)	0,003884526
**67**	Fc epsilon RI signaling pathway (hsa04664)	0,004642571
**68**	Thyroid cancer (hsa05216)	0,006803136
**69**	Amoebiasis (hsa05146)	0,008682158
**70**	Pancreatic cancer (hsa05212)	0,01068502
**71**	Protein processing in endoplasmic reticulum (hsa04141)	0,01115217
**72**	Folate biosynthesis (hsa00790)	0,01221622
**73**	Valine, leucine and isoleucine biosynthesis (hsa00290)	0,01387064
**74**	Aldosterone-regulated sodium reabsorption (hsa04960)	0,01740304
**75**	Epstein-Barr virus infection (hsa05169)	0,02594112
**76**	Chemokine signaling pathway (hsa04062)	0,02624899
**77**	Cholinergic synapse (hsa04725)	0,02800947
**78**	Epithelial cell signaling in Helicobacter pylori infection (hsa05120)	0,02807072

**79**	Notch signaling pathway (hsa04330)	0,03224311
**80**	RIG-I-like receptor signaling pathway (hsa04622)	0,03314291
**81**	Vibrio cholerae infection (hsa05110)	0,03382052
**82**	Calcium signaling pathway (hsa04020)	0,04417211
**83**	HIF-1 signaling pathway (hsa04066)	0,0454352
**84**	Pathogenic Escherichia coli infection (hsa05130)	0,04886979
**85**	Glioma (hsa05214)	0,04886979

Supplementary Table 12

Legend:

**RNAseq_Run**	SRA accession number for RNAseq experiment in GTEx Project
**WGS_Run**	SRA accession number for WGS experiment in GTEx Project
**Tissue**	Tissue name
**BodySite**	Body Site Name
**Abrreviated_BodySite**	Abbreviated Body Site Name
**n.As**	Number of As at GRIA2 Q/R site
**n.Gs**	Number of Gs at GRIA2 Q/R site
**rCov**	RNAseq coverage at GRIA2 Q/R site
**EditingFreq**	RNA editng frequency at GRIA2 Q/R site
**gCov**	WGS coverage at GRIA2 Q/R site

RNAseq_Run	WGS_Run	Tissue	BodySite	Abrreviated_B	n.As	n.Gs	rCov	EditingFreq	gCov
SRR1085015	SRR2167793	Brain	Brain - Amygdala	AM	8	21	29	0,72	38
SRR1095865	SRR2166954	Brain	Brain - Amygdala	AM	3	19	22	0,86	24
SRR598124	SRR2167175	Brain	Brain - Amygdala	AM	9	10	19	0,53	40
SRR598671	SRR2165704	Brain	Brain - Amygdala	AM	0	25	25	1	43
SRR599448	SRR2157426	Brain	Brain - Amygdala	AM	3	23	26	0,88	41
SRR600445	SRR2166846	Brain	Brain - Amygdala	AM	1	141	142	0,99	35
SRR603068	SRR2165027	Brain	Brain - Amygdala	AM	1	37	38	0,97	42
SRR603534	SRR2170759	Brain	Brain - Amygdala	AM	4	43	47	0,91	35
SRR607445	SRR2165757	Brain	Brain - Amygdala	AM	2	9	11	0,82	53
SRR607478	SRR2167268	Brain	Brain - Amygdala	AM	2	26	28	0,93	31
SRR614647	SRR2167626	Brain	Brain - Amygdala	AM	1	89	90	0,99	30
SRR655435	SRR2165627	Brain	Brain - Amygdala	AM	3	59	62	0,95	30
SRR656516	SRR2164775	Brain	Brain - Amygdala	AM	3	11	14	0,79	24
SRR657151	SRR2167534	Brain	Brain - Amygdala	AM	4	40	44	0,91	36
SRR657891	SRR2165893	Brain	Brain - Amygdala	AM	1	43	44	0,98	28
SRR659625	SRR2156397	Brain	Brain - Amygdala	AM	2	24	26	0,92	25
SRR660330	SRR2165734	Brain	Brain - Amygdala	AM	2	51	53	0,96	27
SRR661818	SRR2157265	Brain	Brain - Amygdala	AM	1	21	22	0,95	47
SRR662978	SRR2165707	Brain	Brain - Amygdala	AM	3	49	52	0,94	40
SRR818418	SRR2165852	Brain	Brain - Amygdala	AM	2	120	122	0,98	39
SRR821549	SRR2165974	Brain	Brain - Amygdala	AM	4	138	142	0,97	25
SRR1073143	SRR2167793	Brain	Brain - Anterior cingulate cortex (BA2	ACC	1	106	107	0,99	38
SRR1085825	SRR2166510	Brain	Brain - Anterior cingulate cortex (BA2	ACC	2	76	78	0,97	19
SRR1085850	SRR2166954	Brain	Brain - Anterior cingulate cortex (BA2	ACC	0	70	70	1	24
SRR1323106	SRR2165027	Brain	Brain - Anterior cingulate cortex (BA2	ACC	0	241	241	1	42
SRR1349056	SRR2165627	Brain	Brain - Anterior cingulate cortex (BA2	ACC	0	190	190	1	30
SRR1440058	SRR2157265	Brain	Brain - Anterior cingulate cortex (BA2	ACC	1	47	48	0,98	47
SRR600385	SRR2165270	Brain	Brain - Anterior cingulate cortex (BA2	ACC	5	66	71	0,93	44
SRR600608	SRR2157426	Brain	Brain - Anterior cingulate cortex (BA2	ACC	5	144	149	0,97	41
SRR601755	SRR2165757	Brain	Brain - Anterior cingulate cortex (BA2	ACC	2	14	16	0,88	53
SRR602365	SRR2165704	Brain	Brain - Anterior cingulate cortex (BA2	ACC	4	14	18	0,78	43
SRR602728	SRR2166846	Brain	Brain - Anterior cingulate cortex (BA2	ACC	1	180	181	0,99	35
SRR607091	SRR2167175	Brain	Brain - Anterior cingulate cortex (BA2	ACC	6	25	31	0,81	40
SRR607775	SRR2165027	Brain	Brain - Anterior cingulate cortex (BA2	ACC	0	65	65	1	42
SRR614742	SRR2165627	Brain	Brain - Anterior cingulate cortex (BA2	ACC	0	25	25	1	30
SRR657683	SRR2167534	Brain	Brain - Anterior cingulate cortex (BA2	ACC	0	92	92	1	36
SRR658129	SRR2170255	Brain	Brain - Anterior cingulate cortex (BA2	ACC	5	619	624	0,99	49
SRR659235	SRR2166936	Brain	Brain - Anterior cingulate cortex (BA2	ACC	4	242	246	0,98	15
SRR660945	SRR2156397	Brain	Brain - Anterior cingulate cortex (BA2	ACC	0	42	42	1	25
SRR661133	SRR2157265	Brain	Brain - Anterior cingulate cortex (BA2	ACC	0	5	5	1	47
SRR814989	SRR2167595	Brain	Brain - Anterior cingulate cortex (BA2	ACC	0	196	196	1	36
SRR820623	SRR2165027	Brain	Brain - Anterior cingulate cortex (BA2	ACC	1	572	573	1	42
SRR821626	SRR2165974	Brain	Brain - Anterior cingulate cortex (BA2	ACC	3	298	301	0,99	25
SRR1075433	SRR2166510	Brain	Brain - Caudate (basal ganglia)	CA	1	18	19	0,95	19
SRR1082328	SRR2167793	Brain	Brain - Caudate (basal ganglia)	CA	1	19	20	0,95	38
SRR1086561	SRR2166954	Brain	Brain - Caudate (basal ganglia)	CA	7	26	33	0,79	24
SRR598428	SRR2166846	Brain	Brain - Caudate (basal ganglia)	CA	3	156	159	0,98	35
SRR598638	SRR2157426	Brain	Brain - Caudate (basal ganglia)	CA	9	31	40	0,78	41
SRR599192	SRR2165704	Brain	Brain - Caudate (basal ganglia)	CA	3	62	65	0,95	43
SRR602622	SRR2170759	Brain	Brain - Caudate (basal ganglia)	CA	1	42	43	0,98	35
SRR602839	SRR2157433	Brain	Brain - Caudate (basal ganglia)	CA	2	20	22	0,91	19
SRR604366	SRR2167175	Brain	Brain - Caudate (basal ganglia)	CA	6	26	32	0,81	40
SRR607406	SRR2167268	Brain	Brain - Caudate (basal ganglia)	CA	0	14	14	1	31
SRR607611	SRR2165757	Brain	Brain - Caudate (basal ganglia)	CA	5	17	22	0,77	53
SRR608174	SRR2165027	Brain	Brain - Caudate (basal ganglia)	CA	10	103	113	0,91	42
SRR612899	SRR2167626	Brain	Brain - Caudate (basal ganglia)	CA	3	37	40	0,93	30
SRR613090	SRR2165627	Brain	Brain - Caudate (basal ganglia)	CA	0	154	154	1	30
SRR614479	SRR2167752	Brain	Brain - Caudate (basal ganglia)	CA	0	27	27	1	33
SRR655555	SRR2170255	Brain	Brain - Caudate (basal ganglia)	CA	27	172	199	0,86	49
SRR656552	SRR2156397	Brain	Brain - Caudate (basal ganglia)	CA	0	17	17	1	25
SRR657587	SRR2164775	Brain	Brain - Caudate (basal ganglia)	CA	0	139	139	1	24
SRR661325	SRR2166936	Brain	Brain - Caudate (basal ganglia)	CA	0	289	289	1	15
SRR662689	SRR2157265	Brain	Brain - Caudate (basal ganglia)	CA	0	81	81	1	47
SRR662725	SRR2165734	Brain	Brain - Caudate (basal ganglia)	CA	6	25	31	0,81	27
SRR665298	SRR2165893	Brain	Brain - Caudate (basal ganglia)	CA	0	24	24	1	28
SRR665393	SRR2165707	Brain	Brain - Caudate (basal ganglia)	CA	0	64	64	1	40
SRR816721	SRR2165974	Brain	Brain - Caudate (basal ganglia)	CA	4	85	89	0,96	25
SRR817927	SRR2170659	Brain	Brain - Caudate (basal ganglia)	CA	18	68	86	0,79	32
SRR818057	SRR2170047	Brain	Brain - Caudate (basal ganglia)	CA	0	13	13	1	28
SRR818396	SRR2165852	Brain	Brain - Caudate (basal ganglia)	CA	2	74	76	0,97	39

SRR819946	SRR2167268	Brain	Brain - Caudate (basal ganglia)	CA	4	21	25	0,84	31
SRR820292	SRR2167411	Brain	Brain - Caudate (basal ganglia)	CA	0	7	7	1	56
SRR1074164	SRR2166510	Brain	Brain - Cerebellar Hemisphere	CH	1	48	49	0,98	19
SRR1080927	SRR2166954	Brain	Brain - Cerebellar Hemisphere	CH	1	80	81	0,99	24
SRR1097392	SRR2165974	Brain	Brain - Cerebellar Hemisphere	CH	0	83	83	1	25
SRR1097752	SRR2170047	Brain	Brain - Cerebellar Hemisphere	CH	0	68	68	1	28
SRR1098519	SRR2167793	Brain	Brain - Cerebellar Hemisphere	CH	0	69	69	1	38
SRR1475737	SRR2167752	Brain	Brain - Cerebellar Hemisphere	CH	2	73	75	0,97	33
SRR2166176	SRR2165707	Brain	Brain - Cerebellar Hemisphere	CH	3	535	538	0,99	40
SRR2167642	SRR2164775	Brain	Brain - Cerebellar Hemisphere	CH	6	457	463	0,99	24
SRR598533	SRR2165757	Brain	Brain - Cerebellar Hemisphere	CH	0	24	24	1	53
SRR600754	SRR2165704	Brain	Brain - Cerebellar Hemisphere	CH	1	49	50	0,98	43
SRR600957	SRR2167268	Brain	Brain - Cerebellar Hemisphere	CH	1	51	52	0,98	31
SRR603596	SRR2157426	Brain	Brain - Cerebellar Hemisphere	CH	1	63	64	0,98	41
SRR604098	SRR2165027	Brain	Brain - Cerebellar Hemisphere	CH	0	48	48	1	42
SRR607887	SRR2170759	Brain	Brain - Cerebellar Hemisphere	CH	0	26	26	1	35
SRR608718	SRR2166846	Brain	Brain - Cerebellar Hemisphere	CH	1	110	111	0,99	35
SRR613498	SRR2167626	Brain	Brain - Cerebellar Hemisphere	CH	1	109	110	0,99	30
SRR615599	SRR2167175	Brain	Brain - Cerebellar Hemisphere	CH	0	55	55	1	40
SRR615766	SRR2167752	Brain	Brain - Cerebellar Hemisphere	CH	1	31	32	0,97	33
SRR655375	SRR2165734	Brain	Brain - Cerebellar Hemisphere	CH	0	108	108	1	27
SRR655684	SRR2165707	Brain	Brain - Cerebellar Hemisphere	CH	0	95	95	1	40
SRR656179	SRR2166936	Brain	Brain - Cerebellar Hemisphere	CH	4	153	157	0,97	15
SRR660498	SRR2165893	Brain	Brain - Cerebellar Hemisphere	CH	0	16	16	1	28
SRR660812	SRR2164775	Brain	Brain - Cerebellar Hemisphere	CH	2	152	154	0,99	24
SRR662570	SRR2157265	Brain	Brain - Cerebellar Hemisphere	CH	2	81	83	0,98	47
SRR662799	SRR2156397	Brain	Brain - Cerebellar Hemisphere	CH	0	110	110	1	25
SRR818188	SRR2167595	Brain	Brain - Cerebellar Hemisphere	CH	1	67	68	0,99	36
SRR819999	SRR2165852	Brain	Brain - Cerebellar Hemisphere	CH	0	135	135	1	39
SRR821468	SRR2170659	Brain	Brain - Cerebellar Hemisphere	CH	0	73	73	1	32
SRR1093527	SRR2166510	Brain	Brain - Cerebellum	CE	1	66	67	0,99	19
SRR1398839	SRR2170047	Brain	Brain - Cerebellum	CE	0	50	50	1	28
SRR598009	SRR2165270	Brain	Brain - Cerebellum	CE	1	63	64	0,98	44
SRR600876	SRR2166846	Brain	Brain - Cerebellum	CE	0	41	41	1	35
SRR601098	SRR2167268	Brain	Brain - Cerebellum	CE	2	40	42	0,95	31
SRR604146	SRR2167175	Brain	Brain - Cerebellum	CE	0	45	45	1	40
SRR607311	SRR2165027	Brain	Brain - Cerebellum	CE	1	60	61	0,98	42
SRR607967	SRR2165704	Brain	Brain - Cerebellum	CE	2	90	92	0,98	43
SRR612419	SRR2165627	Brain	Brain - Cerebellum	CE	0	84	84	1	30
SRR613747	SRR2167752	Brain	Brain - Cerebellum	CE	0	48	48	1	33
SRR614383	SRR2167626	Brain	Brain - Cerebellum	CE	1	27	28	0,96	30
SRR615249	SRR2165704	Brain	Brain - Cerebellum	CE	0	74	74	1	43
SRR627299	SRR2165704	Brain	Brain - Cerebellum	CE	0	24	24	1	43
SRR627429	SRR2165704	Brain	Brain - Cerebellum	CE	0	118	118	1	43
SRR627433	SRR2165027	Brain	Brain - Cerebellum	CE	1	18	19	0,95	42
SRR627451	SRR2165027	Brain	Brain - Cerebellum	CE	4	81	85	0,95	42
SRR627453	SRR2167175	Brain	Brain - Cerebellum	CE	0	15	15	1	40
SRR627462	SRR2167175	Brain	Brain - Cerebellum	CE	1	60	61	0,98	40
SRR657915	SRR2165707	Brain	Brain - Cerebellum	CE	2	160	162	0,99	40
SRR657997	SRR2167534	Brain	Brain - Cerebellum	CE	1	108	109	0,99	36
SRR658813	SRR2165734	Brain	Brain - Cerebellum	CE	3	149	152	0,98	27
SRR659331	SRR2157265	Brain	Brain - Cerebellum	CE	6	89	95	0,94	47
SRR659412	SRR2165893	Brain	Brain - Cerebellum	CE	0	78	78	1	28
SRR662871	SRR2170255	Brain	Brain - Cerebellum	CE	2	242	244	0,99	49
SRR663453	SRR2166936	Brain	Brain - Cerebellum	CE	0	145	145	1	15
SRR663681	SRR2164775	Brain	Brain - Cerebellum	CE	2	46	48	0,96	24
SRR807657	SRR2165852	Brain	Brain - Cerebellum	CE	0	97	97	1	39
SRR815232	SRR2170659	Brain	Brain - Cerebellum	CE	2	84	86	0,98	32
SRR816292	SRR2165974	Brain	Brain - Cerebellum	CE	3	109	112	0,97	25
SRR817190	SRR2167595	Brain	Brain - Cerebellum	CE	0	78	78	1	36
SRR817258	SRR2166954	Brain	Brain - Cerebellum	CE	1	176	177	0,99	24
SRR820468	SRR2156397	Brain	Brain - Cerebellum	CE	2	123	125	0,98	25
SRR1081741	SRR2167793	Brain	Brain - Cortex	CO	1	74	75	0,99	38
SRR1083632	SRR2166510	Brain	Brain - Cortex	CO	3	102	105	0,97	19
SRR1382732	SRR2165704	Brain	Brain - Cortex	CO	4	26	30	0,87	43
SRR1475168	SRR2167752	Brain	Brain - Cortex	CO	4	73	77	0,95	33
SRR2038083	SRR2038099	Brain	Brain - Cortex	CO	7	772	779	0,99	19
SRR2038084	SRR2038097	Brain	Brain - Cortex	CO	8	489	497	0,98	60
SRR2038090	SRR2038095	Brain	Brain - Cortex	CO	11	1297	1308	0,99	24
SRR601006	SRR2165270	Brain	Brain - Cortex	CO	0	15	15	1	44
SRR601669	SRR2167175	Brain	Brain - Cortex	CO	10	41	51	0,8	40

SRR602927	SRR2166846	Brain	Brain - Cortex	CO	0	43	43	1	35
SRR603333	SRR2157433	Brain	Brain - Cortex	CO	2	40	42	0,95	19
SRR604026	SRR2167268	Brain	Brain - Cortex	CO	3	24	27	0,89	31
SRR608662	SRR2165757	Brain	Brain - Cortex	CO	3	21	24	0,88	53
SRR614310	SRR2165627	Brain	Brain - Cortex	CO	0	85	85	1	30
SRR627421	SRR2167175	Brain	Brain - Cortex	CO	1	17	18	0,94	40
SRR627425	SRR2165027	Brain	Brain - Cortex	CO	3	8	11	0,73	42
SRR627449	SRR2167175	Brain	Brain - Cortex	CO	14	185	199	0,93	40
SRR627455	SRR2165027	Brain	Brain - Cortex	CO	6	145	151	0,96	42
SRR654874	SRR2167534	Brain	Brain - Cortex	CO	1	25	26	0,96	36
SRR656745	SRR2156397	Brain	Brain - Cortex	CO	16	196	212	0,92	25
SRR660626	SRR2166936	Brain	Brain - Cortex	CO	10	165	175	0,94	15
SRR660933	SRR2164775	Brain	Brain - Cortex	CO	5	104	109	0,95	24
SRR663320	SRR2165734	Brain	Brain - Cortex	CO	6	191	197	0,97	27
SRR663753	SRR2170255	Brain	Brain - Cortex	CO	13	358	371	0,96	49
SRR808614	SRR2166954	Brain	Brain - Cortex	CO	3	119	122	0,98	24
SRR810319	SRR2170659	Brain	Brain - Cortex	CO	2	118	120	0,98	32
SRR810877	SRR2165852	Brain	Brain - Cortex	CO	1	95	96	0,99	39
SRR812012	SRR2165707	Brain	Brain - Cortex	CO	2	93	95	0,98	40
SRR812436	SRR2167595	Brain	Brain - Cortex	CO	4	139	143	0,97	36
SRR820078	SRR2165974	Brain	Brain - Cortex	CO	3	175	178	0,98	25
SRR1077405	SRR2165573	Brain	Brain - Frontal Cortex (BA9)	FC	2	3	5	0,6	45
SRR1084649	SRR2170047	Brain	Brain - Frontal Cortex (BA9)	FC	0	23	23	1	28
SRR1084842	SRR2165974	Brain	Brain - Frontal Cortex (BA9)	FC	0	329	329	1	25
SRR1085495	SRR2166954	Brain	Brain - Frontal Cortex (BA9)	FC	0	117	117	1	24
SRR1102055	SRR2167793	Brain	Brain - Frontal Cortex (BA9)	FC	0	170	170	1	38
SRR1335446	SRR2165757	Brain	Brain - Frontal Cortex (BA9)	FC	2	12	14	0,86	53
SRR2167030	SRR2164775	Brain	Brain - Frontal Cortex (BA9)	FC	0	1305	1305	1	24
SRR595926	SRR2165027	Brain	Brain - Frontal Cortex (BA9)	FC	1	135	136	0,99	42
SRR600361	SRR2165704	Brain	Brain - Frontal Cortex (BA9)	FC	1	62	63	0,98	43
SRR602314	SRR2167268	Brain	Brain - Frontal Cortex (BA9)	FC	3	131	134	0,98	31
SRR602516	SRR2157426	Brain	Brain - Frontal Cortex (BA9)	FC	1	224	225	1	41
SRR602704	SRR2166846	Brain	Brain - Frontal Cortex (BA9)	FC	1	311	312	1	35
SRR604262	SRR2164888	Brain	Brain - Frontal Cortex (BA9)	FC	5	41	46	0,89	38
SRR604456	SRR2170759	Brain	Brain - Frontal Cortex (BA9)	FC	1	41	42	0,98	35
SRR607337	SRR2157433	Brain	Brain - Frontal Cortex (BA9)	FC	2	103	105	0,98	19
SRR612563	SRR2167752	Brain	Brain - Frontal Cortex (BA9)	FC	1	12	13	0,92	33
SRR613627	SRR2167175	Brain	Brain - Frontal Cortex (BA9)	FC	3	144	147	0,98	40
SRR657127	SRR2156397	Brain	Brain - Frontal Cortex (BA9)	FC	11	23	34	0,68	25
SRR657635	SRR2165734	Brain	Brain - Frontal Cortex (BA9)	FC	0	168	168	1	27
SRR657777	SRR2157265	Brain	Brain - Frontal Cortex (BA9)	FC	2	35	37	0,95	47
SRR658307	SRR2167534	Brain	Brain - Frontal Cortex (BA9)	FC	5	79	84	0,94	36
SRR661349	SRR2165893	Brain	Brain - Frontal Cortex (BA9)	FC	1	215	216	1	28
SRR662233	SRR2164775	Brain	Brain - Frontal Cortex (BA9)	FC	0	313	313	1	24
SRR662534	SRR2165707	Brain	Brain - Frontal Cortex (BA9)	FC	0	260	260	1	40
SRR814967	SRR2165852	Brain	Brain - Frontal Cortex (BA9)	FC	7	352	359	0,98	39
SRR818033	SRR2170659	Brain	Brain - Frontal Cortex (BA9)	FC	4	192	196	0,98	32
SRR818210	SRR2167595	Brain	Brain - Frontal Cortex (BA9)	FC	0	327	327	1	36
SRR821602	SRR2165757	Brain	Brain - Frontal Cortex (BA9)	FC	10	8	18	0,44	53
SRR1088389	SRR2167793	Brain	Brain - Hippocampus	HI	0	57	57	1	38
SRR1096851	SRR2166510	Brain	Brain - Hippocampus	HI	2	43	45	0,96	19
SRR1097978	SRR2157426	Brain	Brain - Hippocampus	HI	3	7	10	0,7	41
SRR1378155	SRR2165852	Brain	Brain - Hippocampus	HI	0	23	23	1	39
SRR1458134	SRR2165627	Brain	Brain - Hippocampus	HI	1	117	118	0,99	30
SRR1472770	SRR2167175	Brain	Brain - Hippocampus	HI	6	14	20	0,7	40
SRR1498616	SRR2165704	Brain	Brain - Hippocampus	HI	1	24	25	0,96	43
SRR602169	SRR2166846	Brain	Brain - Hippocampus	HI	6	225	231	0,97	35
SRR603658	SRR2165757	Brain	Brain - Hippocampus	HI	3	9	12	0,75	53
SRR607935	SRR2165270	Brain	Brain - Hippocampus	HI	0	14	14	1	44
SRR608456	SRR2165027	Brain	Brain - Hippocampus	HI	0	76	76	1	42
SRR613474	SRR2165627	Brain	Brain - Hippocampus	HI	0	16	16	1	30
SRR614814	SRR2167626	Brain	Brain - Hippocampus	HI	0	91	91	1	30
SRR656564	SRR2165734	Brain	Brain - Hippocampus	HI	0	23	23	1	27
SRR658977	SRR2165893	Brain	Brain - Hippocampus	HI	0	27	27	1	28
SRR660103	SRR2156397	Brain	Brain - Hippocampus	HI	2	34	36	0,94	25
SRR660969	SRR2164775	Brain	Brain - Hippocampus	HI	3	155	158	0,98	24
SRR661255	SRR2167534	Brain	Brain - Hippocampus	HI	8	25	33	0,76	36
SRR661779	SRR2170255	Brain	Brain - Hippocampus	HI	0	72	72	1	49
SRR662007	SRR2165707	Brain	Brain - Hippocampus	HI	0	66	66	1	40
SRR817751	SRR2170659	Brain	Brain - Hippocampus	HI	2	214	216	0,99	32
SRR817758	SRR2167268	Brain	Brain - Hippocampus	HI	4	35	39	0,9	31

SRR818086	SRR2157265	Brain	Brain - Hippocampus	HI	9	26	35	0,74	47
SRR819134	SRR2167595	Brain	Brain - Hippocampus	HI	5	89	94	0,95	36
SRR819370	SRR2165974	Brain	Brain - Hippocampus	HI	2	61	63	0,97	25
SRR821690	SRR2167175	Brain	Brain - Hippocampus	HI	12	31	43	0,72	40
SRR1071880	SRR2170047	Brain	Brain - Hypothalamus	HY	1	33	34	0,97	28
SRR1072504	SRR2165974	Brain	Brain - Hypothalamus	HY	1	109	110	0,99	25
SRR1072797	SRR2167793	Brain	Brain - Hypothalamus	HY	0	110	110	1	38
SRR1080835	SRR2166954	Brain	Brain - Hypothalamus	HY	2	70	72	0,97	24
SRR1083100	SRR2166510	Brain	Brain - Hypothalamus	HY	4	40	44	0,91	19
SRR1389059	SRR2157426	Brain	Brain - Hypothalamus	HY	9	22	31	0,71	41
SRR598862	SRR2166846	Brain	Brain - Hypothalamus	HY	6	39	45	0,87	35
SRR601169	SRR2165270	Brain	Brain - Hypothalamus	HY	4	68	72	0,94	44
SRR602193	SRR2170759	Brain	Brain - Hypothalamus	HY	1	43	44	0,98	35
SRR604318	SRR2167175	Brain	Brain - Hypothalamus	HY	2	179	181	0,99	40
SRR608230	SRR2165704	Brain	Brain - Hypothalamus	HY	3	14	17	0,82	43
SRR608574	SRR2167268	Brain	Brain - Hypothalamus	HY	1	52	53	0,98	31
SRR613354	SRR2167752	Brain	Brain - Hypothalamus	HY	0	16	16	1	33
SRR614083	SRR2167626	Brain	Brain - Hypothalamus	HY	9	51	60	0,85	30
SRR614443	SRR2165027	Brain	Brain - Hypothalamus	HY	6	31	37	0,84	42
SRR659013	SRR2165734	Brain	Brain - Hypothalamus	HY	1	26	27	0,96	27
SRR660091	SRR2165707	Brain	Brain - Hypothalamus	HY	0	292	292	1	40
SRR661179	SRR2165893	Brain	Brain - Hypothalamus	HY	1	57	58	0,98	28
SRR661445	SRR2157265	Brain	Brain - Hypothalamus	HY	5	50	55	0,91	47
SRR661995	SRR2167534	Brain	Brain - Hypothalamus	HY	0	60	60	1	36
SRR665502	SRR2164775	Brain	Brain - Hypothalamus	HY	0	46	46	1	24
SRR818270	SRR2165757	Brain	Brain - Hypothalamus	HY	30	4	34	0,12	53
SRR820256	SRR2170659	Brain	Brain - Hypothalamus	HY	9	34	43	0,79	32
SRR820379	SRR2165852	Brain	Brain - Hypothalamus	HY	2	7	9	0,78	39
SRR1070986	SRR2165974	Brain	Brain - Nucleus accumbens (basal gar	NAC	1	136	137	0,99	25
SRR1076072	SRR2166954	Brain	Brain - Nucleus accumbens (basal gar	NAC	3	62	65	0,95	24
SRR1079281	SRR2166510	Brain	Brain - Nucleus accumbens (basal gar	NAC	3	17	20	0,85	19
SRR1082759	SRR2170047	Brain	Brain - Nucleus accumbens (basal gar	NAC	1	12	13	0,92	28
SRR1087031	SRR2167793	Brain	Brain - Nucleus accumbens (basal gar	NAC	0	42	42	1	38
SRR1383831	SRR2165707	Brain	Brain - Nucleus accumbens (basal gar	NAC	0	79	79	1	40
SRR597927	SRR2157426	Brain	Brain - Nucleus accumbens (basal gar	NAC	4	259	263	0,98	41
SRR598196	SRR2165704	Brain	Brain - Nucleus accumbens (basal gar	NAC	4	112	116	0,97	43
SRR598253	SRR2166846	Brain	Brain - Nucleus accumbens (basal gar	NAC	0	221	221	1	35
SRR600348	SRR2167268	Brain	Brain - Nucleus accumbens (basal gar	NAC	5	114	119	0,96	31
SRR600560	SRR2165027	Brain	Brain - Nucleus accumbens (basal gar	NAC	0	223	223	1	42
SRR601122	SRR2170759	Brain	Brain - Nucleus accumbens (basal gar	NAC	5	68	73	0,93	35
SRR601201	SRR2165757	Brain	Brain - Nucleus accumbens (basal gar	NAC	1	8	9	0,89	53
SRR602808	SRR2165270	Brain	Brain - Nucleus accumbens (basal gar	NAC	3	12	15	0,8	44
SRR607141	SRR2167175	Brain	Brain - Nucleus accumbens (basal gar	NAC	0	189	189	1	40
SRR612731	SRR2167752	Brain	Brain - Nucleus accumbens (basal gar	NAC	0	6	6	1	33
SRR613438	SRR2167626	Brain	Brain - Nucleus accumbens (basal gar	NAC	2	83	85	0,98	30
SRR654886	SRR2165893	Brain	Brain - Nucleus accumbens (basal gar	NAC	4	48	52	0,92	28
SRR655134	SRR2164775	Brain	Brain - Nucleus accumbens (basal gar	NAC	5	205	210	0,98	24
SRR655923	SRR2157265	Brain	Brain - Nucleus accumbens (basal gar	NAC	0	15	15	1	47
SRR656167	SRR2167534	Brain	Brain - Nucleus accumbens (basal gar	NAC	2	4	6	0,67	36
SRR657348	SRR2166936	Brain	Brain - Nucleus accumbens (basal gar	NAC	1	160	161	0,99	15
SRR659187	SRR2165734	Brain	Brain - Nucleus accumbens (basal gar	NAC	0	115	115	1	27
SRR664842	SRR2156397	Brain	Brain - Nucleus accumbens (basal gar	NAC	7	66	73	0,9	25
SRR817905	SRR2165852	Brain	Brain - Nucleus accumbens (basal gar	NAC	4	73	77	0,95	39
SRR819793	SRR2167411	Brain	Brain - Nucleus accumbens (basal gar	NAC	1	10	11	0,91	56
SRR820157	SRR2170659	Brain	Brain - Nucleus accumbens (basal gar	NAC	0	193	193	1	32
SRR1075898	SRR2165974	Brain	Brain - Putamen (basal ganglia)	PU	3	47	50	0,94	25
SRR1085662	SRR2166510	Brain	Brain - Putamen (basal ganglia)	PU	5	31	36	0,86	19
SRR1098336	SRR2166954	Brain	Brain - Putamen (basal ganglia)	PU	7	22	29	0,76	24
SRR1099023	SRR2170047	Brain	Brain - Putamen (basal ganglia)	PU	1	9	10	0,9	28
SRR598276	SRR2165027	Brain	Brain - Putamen (basal ganglia)	PU	2	26	28	0,93	42
SRR600533	SRR2157426	Brain	Brain - Putamen (basal ganglia)	PU	2	22	24	0,92	41
SRR601287	SRR2165704	Brain	Brain - Putamen (basal ganglia)	PU	1	30	31	0,97	43
SRR602540	SRR2166846	Brain	Brain - Putamen (basal ganglia)	PU	13	46	59	0,78	35
SRR607538	SRR2167175	Brain	Brain - Putamen (basal ganglia)	PU	2	19	21	0,9	40
SRR614155	SRR2167626	Brain	Brain - Putamen (basal ganglia)	PU	1	28	29	0,97	30
SRR614455	SRR2165627	Brain	Brain - Putamen (basal ganglia)	PU	5	50	55	0,91	30
SRR614515	SRR2167752	Brain	Brain - Putamen (basal ganglia)	PU	1	7	8	0,88	33
SRR658247	SRR2165893	Brain	Brain - Putamen (basal ganglia)	PU	0	31	31	1	28
SRR659121	SRR2165707	Brain	Brain - Putamen (basal ganglia)	PU	8	70	78	0,9	40
SRR659780	SRR2165734	Brain	Brain - Putamen (basal ganglia)	PU	0	26	26	1	27
SRR659899	SRR2164775	Brain	Brain - Putamen (basal ganglia)	PU	1	122	123	0,99	24

SRR817629	SRR2167595	Brain	Brain - Putamen (basal ganglia)	PU	4	40	44	0,91	36
SRR817775	SRR2165852	Brain	Brain - Putamen (basal ganglia)	PU	3	23	26	0,88	39
SRR818971	SRR2170659	Brain	Brain - Putamen (basal ganglia)	PU	5	39	44	0,89	32
SRR1073755	SRR2165757	Brain	Brain - Spinal cord (cervical c-1)	SC	3	3	6	0,5	53
SRR1441701	SRR2164888	Brain	Brain - Spinal cord (cervical c-1)	SC	9	7	16	0,44	38
SRR602598	SRR2166846	Brain	Brain - Spinal cord (cervical c-1)	SC	3	5	8	0,62	35
SRR602871	SRR2170759	Brain	Brain - Spinal cord (cervical c-1)	SC	7	12	19	0,63	35
SRR612311	SRR2165757	Brain	Brain - Spinal cord (cervical c-1)	SC	6	2	8	0,25	53
SRR612407	SRR2165627	Brain	Brain - Spinal cord (cervical c-1)	SC	0	24	24	1	30
SRR613807	SRR2167626	Brain	Brain - Spinal cord (cervical c-1)	SC	3	11	14	0,79	30
SRR614071	SRR2167175	Brain	Brain - Spinal cord (cervical c-1)	SC	2	25	27	0,93	40
SRR615020	SRR2167752	Brain	Brain - Spinal cord (cervical c-1)	SC	1	4	5	0,8	33
SRR655146	SRR2165734	Brain	Brain - Spinal cord (cervical c-1)	SC	9	4	13	0,31	27
SRR660661	SRR2167534	Brain	Brain - Spinal cord (cervical c-1)	SC	0	7	7	1	36
SRR660895	SRR2165707	Brain	Brain - Spinal cord (cervical c-1)	SC	12	24	36	0,67	40
SRR661723	SRR2164775	Brain	Brain - Spinal cord (cervical c-1)	SC	0	23	23	1	24
SRR817880	SRR2167595	Brain	Brain - Spinal cord (cervical c-1)	SC	23	46	69	0,67	36
SRR819534	SRR2164888	Brain	Brain - Spinal cord (cervical c-1)	SC	22	7	29	0,24	38
SRR1078879	SRR2167793	Brain	Brain - Substantia nigra	SN	3	13	16	0,81	38
SRR597977	SRR2170759	Brain	Brain - Substantia nigra	SN	4	7	11	0,64	35
SRR599486	SRR2165704	Brain	Brain - Substantia nigra	SN	2	12	14	0,86	43
SRR601787	SRR2157426	Brain	Brain - Substantia nigra	SN	2	17	19	0,89	41
SRR603397	SRR2167268	Brain	Brain - Substantia nigra	SN	9	11	20	0,55	31
SRR604050	SRR2165027	Brain	Brain - Substantia nigra	SN	3	36	39	0,92	42
SRR604074	SRR2166846	Brain	Brain - Substantia nigra	SN	8	36	44	0,82	35
SRR612455	SRR2167752	Brain	Brain - Substantia nigra	SN	0	5	5	1	33
SRR615467	SRR2167175	Brain	Brain - Substantia nigra	SN	1	7	8	0,88	40
SRR615934	SRR2165270	Brain	Brain - Substantia nigra	SN	5	3	8	0,38	44
SRR657973	SRR2164775	Brain	Brain - Substantia nigra	SN	0	38	38	1	24
SRR661925	SRR2156397	Brain	Brain - Substantia nigra	SN	7	24	31	0,77	25
SRR662138	SRR2165893	Brain	Brain - Substantia nigra	SN	5	43	48	0,9	28
SRR663693	SRR2165707	Brain	Brain - Substantia nigra	SN	12	31	43	0,72	40
SRR665670	SRR2166936	Brain	Brain - Substantia nigra	SN	13	37	50	0,74	15
SRR807591	SRR2165852	Brain	Brain - Substantia nigra	SN	2	174	176	0,99	39
SRR817797	SRR2167595	Brain	Brain - Substantia nigra	SN	2	84	86	0,98	36
SRR818469	SRR2164888	Brain	Brain - Substantia nigra	SN	0	8	8	1	38

Supplementary Table 13

Legend:

**Position (hg19)**	Genomic coordinates (hg19 assembly)
**Name**	miRNA name
**PMID**	PubMed of Publication descriding the editited miRNA
**In pre**	Editing position in pre-miRNA
**In mat**	Editing position in mature miRNA
**Type**	RNA editing type
**CTRL**	Number of edited control donors
**ALS**	Number of edited ALS donors
**Mean-Ctrl**	Mean editing level in controls
**Mean-Als**	Mean editing level in ALS samples
**Median-Ctrl**	Median editing level in controls
**Median-Als**	Median editing level in ALS samples
**Pval**	Mann-Whitney P-value
**Pval_corr**	Corrected P-value (BH correction)

Position (hg19)	Name	PMID	In pre	In mat	Type	CTRL	ALS	Mean-Ctrl	Mean-Als	Median-Ctrl	Median-Als	Pval	Pval_corr
chr14:101489681	hsa-mir-411	22499667	20	5	AG	5	6	0,310	0,287	0,310	0,280	0,1144	0,5000
chr14:101512308	hsa-mir-381	22499667	52	4	AG	5	6	0,082	0,075	0,090	0,080	0,2580	0,5000
chr7:5535483	hsa-mir-589	22499667	66	6	AG	5	5	0,646	0,562	0,670	0,570	0,3376	0,5000
chr21:17911421	hsa-mir-99a	22499667	13	1	AG	5	6	0,016	0,012	0,010	0,010	0,2073	0,5000
chr8:141742704	hsa-mir-151a	22499667	49	3	AG	5	6	0,016	0,010	0,020	0,010	0,0226	0,2708
chr14:101507127	hsa-mir-376a-	22499667	9	3	AG	4	2	0,028	0,100	0,025	0,100	0,1738	0,5000
chrX:73438243	hsa-mir-421	22499667	54	7	AG	5	6	0,010	0,012	0,010	0,010	0,4461	0,5000
chr2:25551539	hsa-mir-1301	-	52	5	AG	4	5	0,128	0,110	0,130	0,100	0,3106	0,5000
chr12:97885696	hsa-mir-1251	-	10	6	AG	5	4	0,192	0,123	0,170	0,120	0,2684	0,5000
chr14:101514299	hsa-mir-889	-	62	14	AG	2	5	0,040	0,048	0,040	0,030	0,4225	0,5000
chr1:1102544	hsa-mir-200b	22499667	61	5	AG	1	3	0,120	0,150	0,120	0,080	0,5000	0,5000
chr11:59976571	hsa-mir-6503	25521855	59	7	AG	1	5	1,000	0,900	1,000	1,000	0,5000	0,5000
